# Using Triazabutadienes as a Protected Source of Diazonium Cations to Facilitate Electrografting to a Variety of Conductive Surfaces

**DOI:** 10.1021/acs.langmuir.4c04848

**Published:** 2025-03-12

**Authors:** Nicholas D. J. Yates, Lucy Hudson, Oscar Schwabe, Alison Parkin

**Affiliations:** Department of Chemistry, University of York, Heslington, York YO10 5DD, U.K.

## Abstract

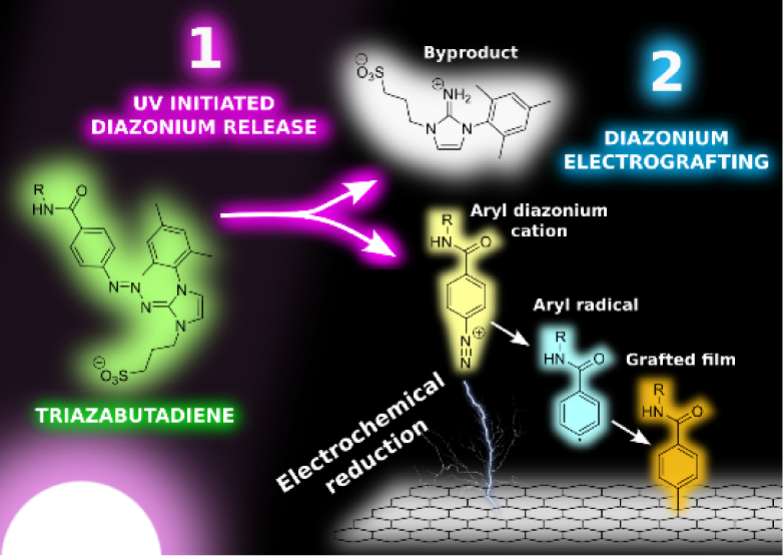

Aryl diazonium electrografting is a versatile methodology for the functionalization of electrode surfaces, yet its usage has been hampered by both the short lifespan of aryl diazonium cations in aqueous solution and the harsh conditions required to generate them *in situ*. This can make accessing complicated aryl diazonium cations and derivatized surfaces thereof difficult. The usage of triazabutadienes has the potential to address many of these issues as triazabutadienes are stable enough to endure multiple-step chemical syntheses and can persist for several hours in aqueous solution, yet upon UV exposure rapidly release aryl diazonium cations under mild conditions (i.e., 0 °C, pH 7 aqueous solution). Herein is described the synthesis and utilization of a versatile, highly water-soluble triazabutadiene scaffold which can be used to access complicated aryl diazonium cations (via photochemical decaging with a commercially available 365 nm UV irradiation source), which themselves can be used to directly derivatize electrode surfaces with desired organic moieties.

## Introduction

**Scheme 1:**
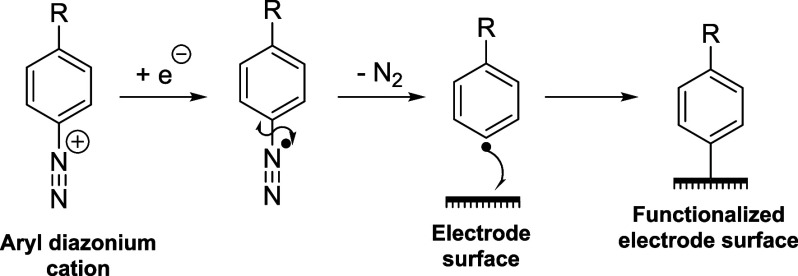
Functionalization of Electrode Surfaces Via the Electrochemical Reduction of Aryl Diazonium Salts

Aryl diazonium cations are highly popular reagents for the functionalization of electrode surfaces, as the electrochemical reduction of aryl diazonium cations produces aryl radicals, which rapidly form covalent bonds to essentially any conductive substrate, including carbon allotropes,^1−3^ ITO,^4^ silicon,^5,6^ and a range of metals (Scheme 1).^7^

However, while aryl diazonium cations have proven themselves to be valuable reagents in the functionalization of electrode surfaces, in practice, their utility is restricted by (i) their poor stability, which can both complicate the synthesis of complex aryl diazonium cations and result in a limited shelf life, and (ii) the harsh conditions typically required to generate diazonium cations from precursors, such as amine-reactive diazotizing agents, low pH, or strong methylating agents.^8−13^ To overcome these limitations, most diazonium electrografting-based strategies only use diazonium electrografting to introduce a simple “root” moiety onto the electrode surface (such as a carboxylic acid, an amine/amine precursor, or an azide/alkyne), and then subsequently install complex moieties on the electrode surface by reacting these “root” moieties with suitable probes.^1,13−16^ However, these reactions are often far more lethargic than their solution-phase analogues (most likely due to steric constraints), and thus, generating a functionalized surface via this strategy is often very time-consuming. Characterization of surfaces is also highly challenging, which can also make it difficult to validate the success or failure of these stepwise installation strategies. It would, therefore, be prudent to devise a strategy whereby desired organic moieties can be synthesized and fully characterized in solution and then directly and rapidly installed onto electrode surfaces “whole.” Furthermore, as aryl diazonium cations react with electron-rich motifs even under mild conditions if given enough time (a property that makes them valuable reagents for bioconjugation reactions to motifs such as tyrosine or histidine),^10,17,18^ it would be highly advantageous to unveil aryl diazonium functionalities immediately before electrografting in order to minimize the time available for unwanted side reactions.

**Figure 1:**
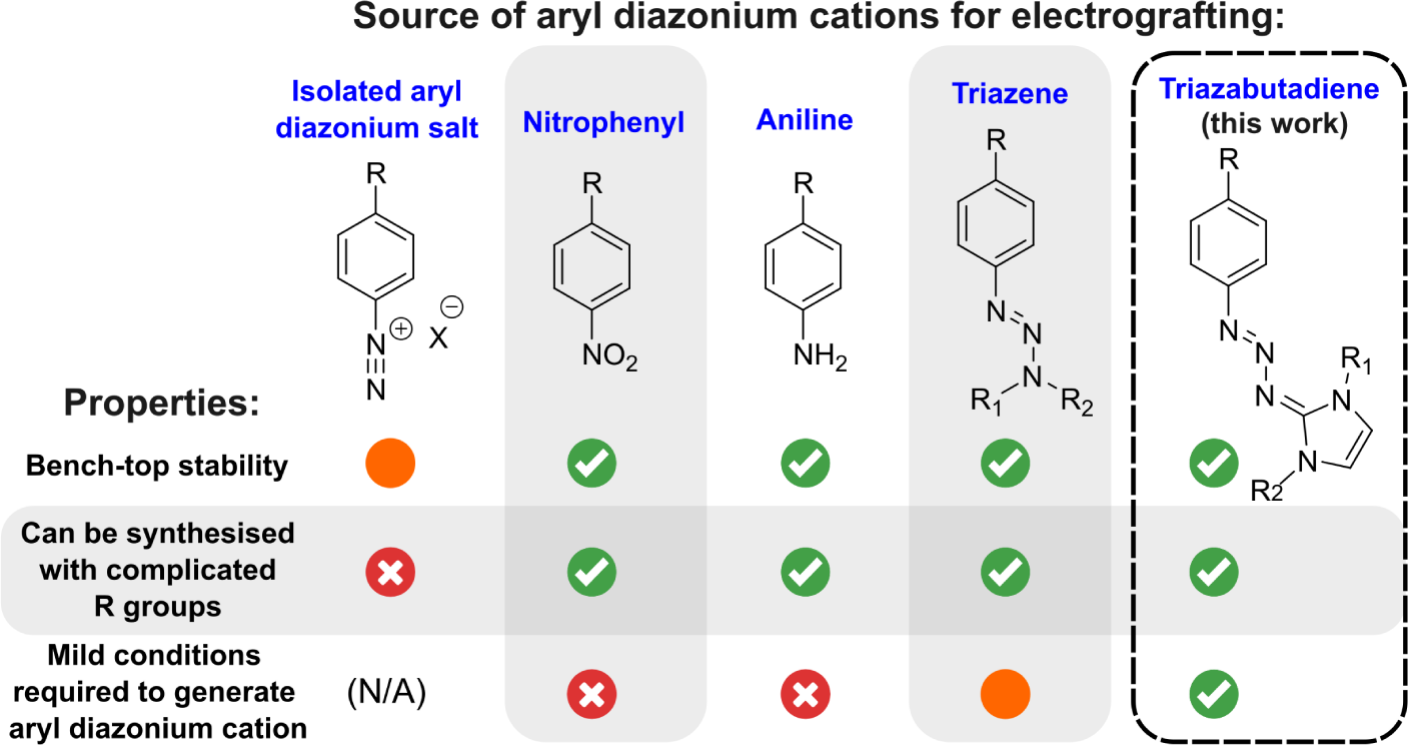
Aryl diazonium cation sources/precursors that can be utilized for electrografting.

**Figure 2:**
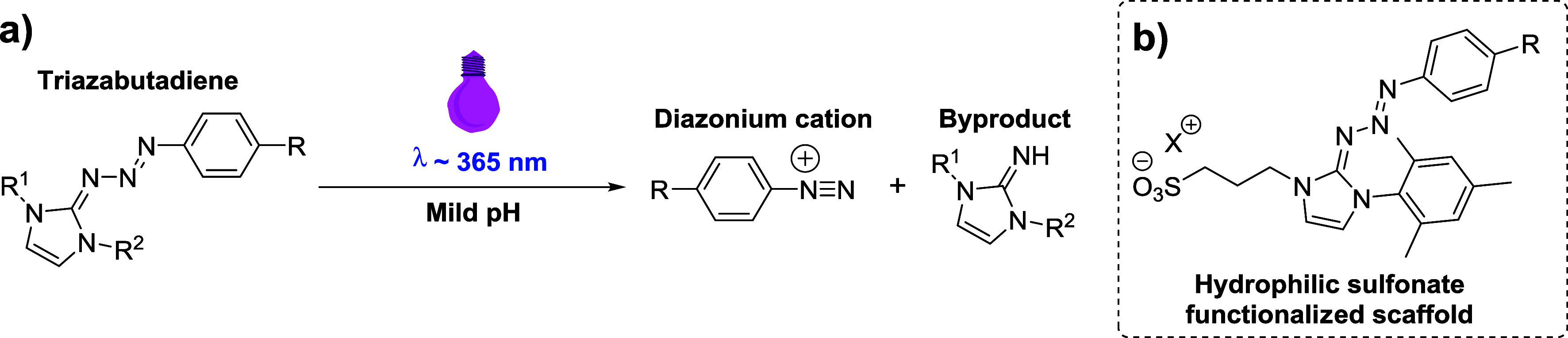
(a) Use of triazabutadienes as photocaged sources of aryl diazonium cations and the general structure of triazabutadiene scaffolds. (b) The general structure of the monomesityl monosulfonate-functionalized water-soluble triazabutadiene scaffold.

The triazabutadienes developed by the Jewett group^19−28^ have the potential to address many of these issues (Figure 1). These species rapidly break down upon exposure to UV irradiation to release diazonium cations (Figure 2a), even under biologically relevant mild conditions,^19−28^ yet are stable enough to endure multiple-step chemical syntheses, can be stored, and can persist for several hours in aqueous solution (when in darkness).^24,25^ The majority of the triazabutadienes developed to date have been used to install bio-orthogonal functionality or fluorophores onto proteins via azo-bond formation between triazabutadiene-derived aryl diazonium cations and electron-rich aromatic residues.^17−20,22^ In our previous work, we explored using this strategy as a method of neoglycoprotein fabrication,^17^ and having thus explored the stability and UV-responsive behavior of triazabutadienes, we hypothesized that triazabutadiene scaffolds could be amenable for usage in diazonium electrografting. We present herein the synthesis and application of a versatile, highly water-soluble, bench-stable triazabutadiene scaffold (Figure 2b) which we use to perform UV-initiated diazonium electrografting.

## Experimental Section

### Synthesis

**Scheme 2:**
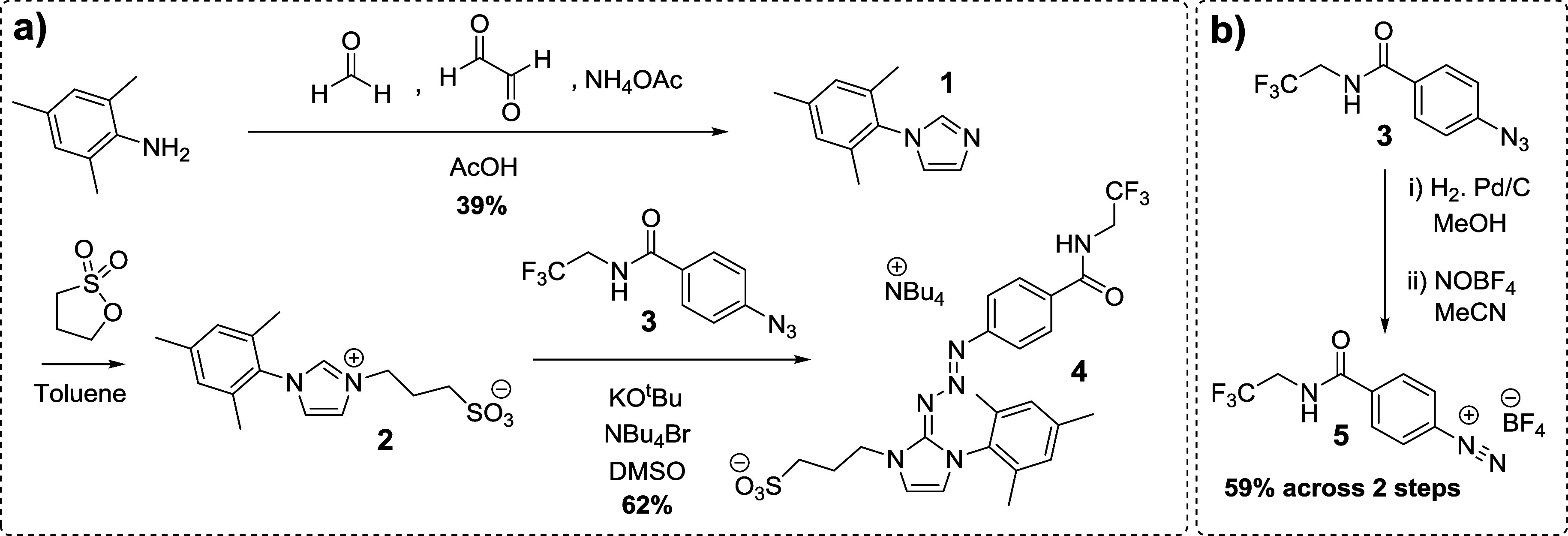
Syntheses of a) Protype Triazabutadiene **4** and b) Isolated Aryl Diazonium Salt **5**

**Scheme 3:**
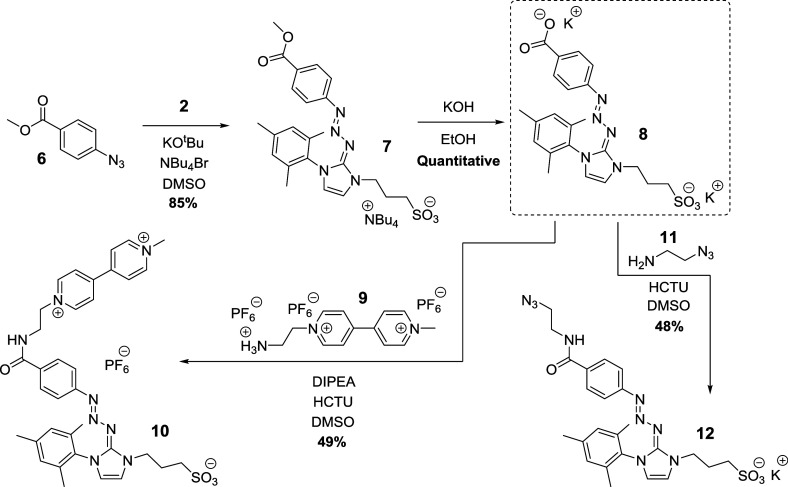
Synthesis of Carboxylate-Functionalized Triazabutadiene **8**, and the Use of This Species to Install Exotic Functionalities via Amide Bond Formation

The synthetic route leading to compounds **1** → **5** is shown in Scheme 2, and the synthetic route leading to compounds **6** → **12** is shown in Scheme 3. Full methods and characterization data can be found in the Supporting Information.

### Buffer Preparation

The buffers used during all experiments had compositions of 50 mM sodium phosphate and 150 mM NaCl. These buffers were prepared by first combining appropriate volumes of 1 M monobasic sodium phosphate and 2 M NaCl, and by then adjusting these solutions to the required pH and volume using NaOH and Milli-Q water.

### UV-Irradiation Sources and Exposure Methods

UV irradiation of solutions of **4** or **10** was performed using commercially available UV nail curing products that emit UV irradiation at 365 nm. UV irradiation of solutions of **4** was performed using a NailStar 36-W Professional UV Nail Lamp (Model: NS-01-UK and EU). Samples of **4** were introduced to quartz cuvettes, which were stood in a shallow dish of icy water inside the NailStar, and UV irradiation was then supplied for the desired durations. UV irradiation of solutions of **10** was performed using a 12 W UV LED Helios Nail Lamp (Item Code: HGPK25) for 30 s at 0 °C.

### UV–Vis Measurement of UV-Triggered Diazonium Release from 4

50 μM **4** were prepared by delivering 100 μL of a 500 μM stock solution of **4** in DMSO to 900 μL of buffer (pH 6.0, 7.0, or 8.0) in a quartz cuvette (1 mL volume and 1 cm path length). Initial UV–vis spectra were then recorded using a DeNovix DS-11 FX+ spectrophotometer. Samples were then exposed to 15 s periods of UV irradiation, with UV–vis spectra being recorded after each irradiation period.

### Half-Life Measurements of 4

In order to measure the half-life of triazabutadiene **4** in aqueous solution, the absorbance at the λ_max_ of the triazabutadiene signal (∼395 nm) was monitored at 24 °C as a function of time for 30 μM solutions of **4** in buffer (pH 7.0, 8.0, or 8.5). Quartz cuvettes (1 mL volume and 1 cm path length) were used, and data were recorded using a UV-1800 Shimadzu Spectrophotometer.

It was assumed that, in buffered aqueous solution (i.e., where [H^+^] is effectively constant) in darkness, the rate at which dilute solutions of triazabutadienes break down due to protonation-triggered aryl diazonium release would follow first-order kinetics, as described by eq S1. After fitting the data to eq S1, it was possible to calculate half-lives using eq S2.

### Electrochemistry

Experiments were conducted in a glovebox (in-house design and construction) under a N_2_ atmosphere (O_2_ ≤ 40 ppm). A PalmSens4 potentiostat (PalmSens) with PSTrace 5.9 for Windows software was used for all electrochemical experiments. Electrochemical experiments were conducted using a variety of electrodes, but all experiments were conducted using a three-electrode setup. Full experimental details of each electrochemical experiment, as well as diagrammatic depictions of the electrodes and electrochemical cells utilized, can be found in the Supporting Information.

Potentials are reported versus those of the standard hydrogen electrode (SHE) reference electrode. These potentials were calculated by recording cyclic voltammograms in a standard ferricyanide solution for each reference electrode and then comparing the midpoint potential to the literature value^29^ (see equation S3).

### XPS

XPS data for the gold-coated silicon wafer slice samples electrografted with solutions of **4**/**5** (see Supporting Information) was acquired using a Kratos Axis SUPRA using monochromated Al kα (1486.69 eV) X-rays at 15 mA emission and 12 kV HT (180 W) and a spot size/analysis area of 700 × 300 μm. The instrument was calibrated to the binding energy (BE) of the gold metal Au 4f_7/2_ line (83.95 eV)^30,31^ and dispersion adjusted to give a BE of 932.6 eV for the Cu 2p_3/2_ line of metallic copper. The Ag 3d_5/2_ line full width at half-maximum (fwhm) at 10 eV pass energy was 0.544 eV. The source resolution for monochromatic Al kα X-rays was ∼0.3 eV. The instrumental resolution was determined to be 0.29 at 10 eV pass energy using the Fermi edge of the valence band for metallic silver. Resolution with the charge compensation system was <1.33 eV fwhm on polytetrafluoroethylene (PTFE). High-resolution spectra were obtained using a pass energy of 20 eV, step size of 0.1 eV, and sweep time of 60 s, resulting in a line width of 0.696 eV for Au 4f_7/2_. Survey spectra were obtained using a pass energy of 160 eV. Samples were grounded onto the instrument via conductive clips. The data were recorded at a base pressure of below 9 × 10^–9^ Torr, a room temperature of 294 K, and an angle of 30°. The data were analyzed using CasaXPS v2.3.19PR1.0.^32^ Data were fit with a Shirley background prior to analysis and were calibrated by setting the Au 4f_7/2_ peak to be equal to the literature value of 83.95 eV.^30,31^ Regions associated with particular elements were assigned based on binding energies reported in the literature.^33−39^

## Results and Discussion

From a purely practical standpoint regarding electrochemistry, the use of aqueous reference electrodes and aqueous electrolytes is often more convenient and cheaper than using organic electrochemical systems. In our previous forays into the use of triazabutadienes,^17,40^ we found the previously reported dimesityl triazabutadiene scaffold (i.e., R^1^ = R^2^ = mesityl in Figure 2a) to be highly hydrophobic, and we thus hypothesized that such triazabutadienes (and the byproduct released upon their exposure to UV light) would be likely to adsorb to electrode surfaces in high quantities. We anticipated that this could complicate efforts to achieve diazonium electrografting from aqueous solutions, and we therefore opted to incorporate a hydrophilic sulfonate group into the triazabutadiene scaffold (Figure 2b); similar scaffolds had previously been studied by the Jewett group^24,25^ and we hypothesized that the negatively charged sulfonate motif would both improve water solubility and reduce the chance of nonspecific adsorption to negatively poised electrode surfaces.

**Figure 3:**
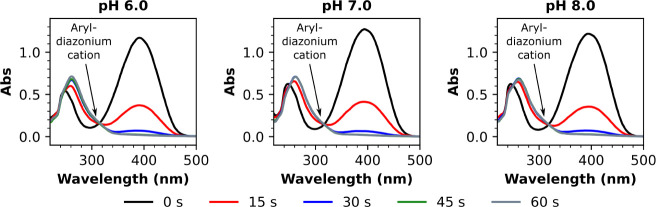
UV–vis analysis shows that **4** breaks down rapidly in response to 365 nm UV irradiation in a buffered aqueous solution to release aryl-diazonium cations. The concentration of compound **4** was 50 μM in each solution.

Prototype water-soluble triazabutadiene **4** was thereafter synthesized (Scheme 2a), and it was found to be both highly water-soluble and bench-stable. Encouragingly, UV–vis analysis proved that **4** was capable of releasing aryl diazonium cations rapidly upon exposure to 365 nm UV irradiation at 0 °C in aqueous solution at mild pH. Figure 3 shows that the triazabutadiene absorption feature at ∼400 nm is rapidly lost, whereas a new band attributable to intact aryl diazonium cations appears at ∼310 nm.^41^

**Figure 4:**
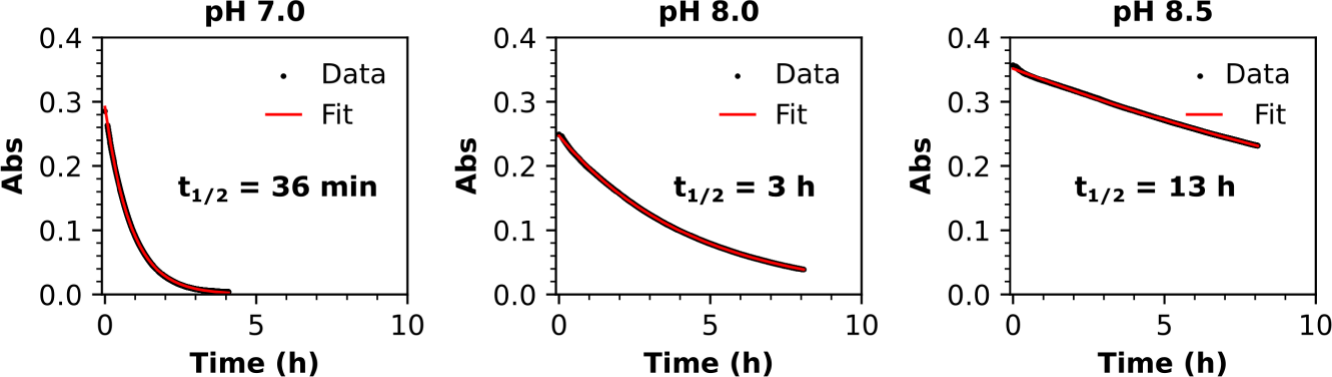
UV–vis analysis showing Abs_395 nm_ as a function of time for phosphate-buffered aqueous solutions of **4**, showing that the half-life of triazabutadiene **4** in aqueous solution increases as a function of pH.

The lifetime of **4** in buffered aqueous solutions in darkness was also evaluated (Figure 4). As would be expected of an acid-sensitive species,^24,25^ the half-life (∼) of **4** increased in aqueous solution over the pH range of 7 to 8.5. Encouragingly, the ∼ of **4** in aqueous solution could be increased from 36 min to 3 h (by a factor of 5) simply by raising the pH from 7.0 to 8.0. The measured value for ∼ at pH 8.5 (13 h) was also far larger than that observed at pH 8.0. We note that these results are in good agreement with studies of similar monomesityl monosulfonate functionalized triazabutadienes conducted by the Jewett group.^24,25^

**Figure 5:**
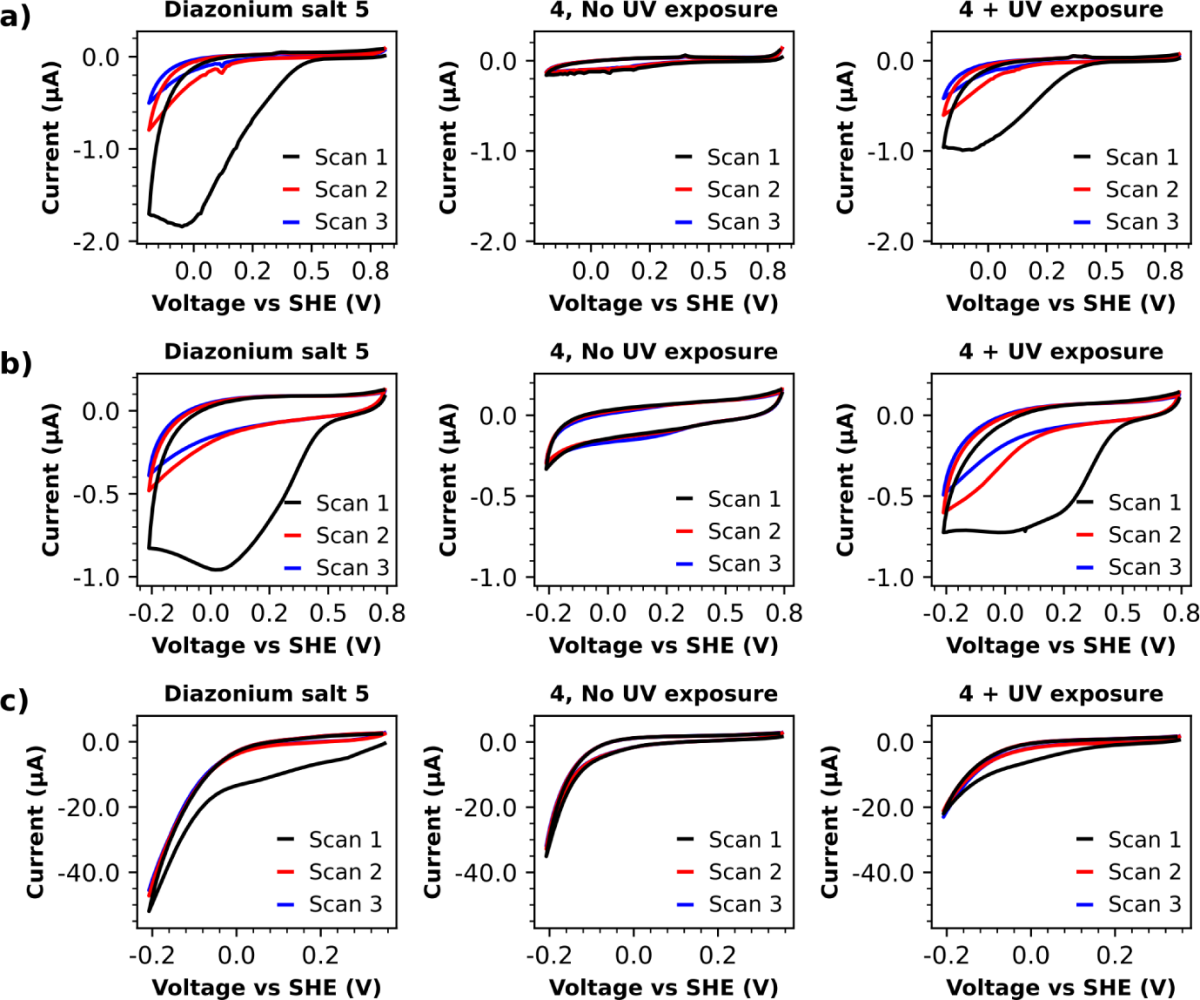
Use of isolated diazonium salt **5**, and comparable triazabutadiene **4** in electrografting experiments on a) carbon screen-printed electrodes, b) glassy carbon disk electrodes, and c) gold-coated silicon wafer slices. CVs were recorded in a pH 7 buffer at a scan rate of 100 mV s^–1^. Achieving significant degrees of aryl diazonium electrografting was clearly dependent on UV-exposure when using **4**.

Buffered aqueous solutions of **4** which had been briefly subjected to UV irradiation were then used to perform diazonium electrografting to carbon screen-printed electrodes (Figure 5a), glassy carbon disk electrodes (Figure 5b), and gold-coated silicon wafer slices (Figure 5c). In all cases, the characteristic clear broad reductive peaks associated with diazonium electrografting were clearly in evidence (Figure 5). The shapes of these diazonium electrografting features were akin to those obtained when the comparable isolated aryl diazonium salt **5** was used instead of **4** (Figure 5), thereby validating that a sufficient portion of the aryl diazonium cations released from **4** survive the brief UV exposure and are able to partake in electrografting. Aryl diazonium electrografting was not observed for samples of **4** that had not been subjected to UV exposure; this is consistent with the data displayed in Figure 4, which suggest that there should be minimal aryl diazonium cation release over a short time interval in the absence of UV irradiation. We observed that the intensities of the reductive electrografting features obtained when **4** was used were often moderately attenuated relative to those obtained using **5** (Figure 5). We did not consider this to be of concern, especially since thick multilayer formation is often regarded as an aggravation when performing diazonium electrografting.^12−16,42−46^ We were, however, intrigued and further probed the potential origins of this phenomenon in Supporting Information (see Figures S123–S125).

**Figure 6:**
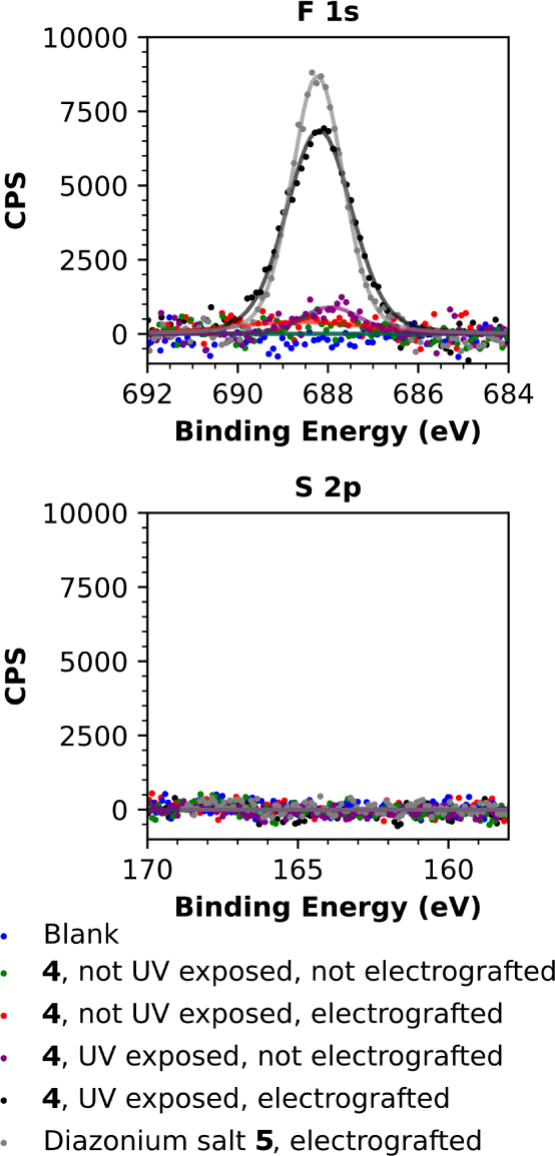
XPS measurements of gold-coated silicon wafer slices treated with samples of either **5** or **4** show significant levels of fluorine to be present only when samples of **5**, or UV-treated samples of **4**, were subjected to electrografting. Sulfur levels were below the limit of detection in all instances.

A CF_3_ group had been incorporated into the design of both triazabutadiene **4** and comparable diazonium salt **5** in order to facilitate the analysis of electrografted surfaces via XPS; detection of fluorine, but not sulfur, would be evidence of successful electrografting of diazonium cations onto a surface (rather than simple adsorption of **4** to the surface). XPS analysis of gold surfaces subjected to the treatments tabulated in Table 1 shows that the only gold surfaces to become fluorine-functionalized above the detection limit were those subjected to electrografting using either isolated diazonium salt **5** or UV-treated samples of **4** (Figures 6 and S127). Sulfur is notably absent (or at least falls below the detection limit), which is indicative that neither **4** or the byproduct released upon the UV-initiated breakdown of **4** irreversibly grafts/strongly adsorbs to the gold electrode surface.

**Table 1:** Treatments to which Gold-Coated Silicon Wafer XPS Samples Were Subjected

Sample name/treatments	UV-treatment (i.e., should aryl diazonium cations have been released?)	Electrochemical treatment (i.e., should aryl diazonium cations have grafted to the electrode via the radical mechanism)
Blank electrode	N/A	N/A
**4**, not UV exposed, not electrografted	No	No
**4**, not UV exposed, electrografted	No	Yes
**4**, UV exposed, not electrografted	Yes	No
**4**, UV exposed, electrografted	Yes	Yes
Diazonium salt **5**, electrografted	N/A	Yes

Having validated that the sulfonate-functionalized triazabutadiene scaffold of **4** was appropriate for facilitating UV-initiated diazonium electrografting in buffered aqueous media, water-soluble carboxylate-functionalized triazabutadiene **8** was synthesized (Scheme 3). The carboxylate functionality of **8** could be readily reacted with amine species to introduce interesting functionalities into the triazabutadiene scaffold, thereby allowing access to diazonium cations that would be challenging to synthesize and isolate using conventional methods.

**Scheme 4:**
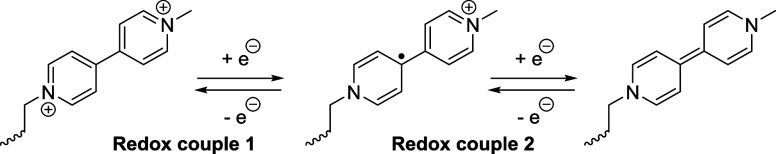
Redox Behavior of Viologen Motifs

The coupling of **8** to amine-functionalized viologen **9** yielded triazabutadiene **10** (Scheme 3). The viologen motif of **10**, being redox-active (Scheme 4), was intended to serve as a redox marker—detection of intense viologen-derived redox signals would be evidence of successful diazonium electrografting from **10**.

**Figure 7:**
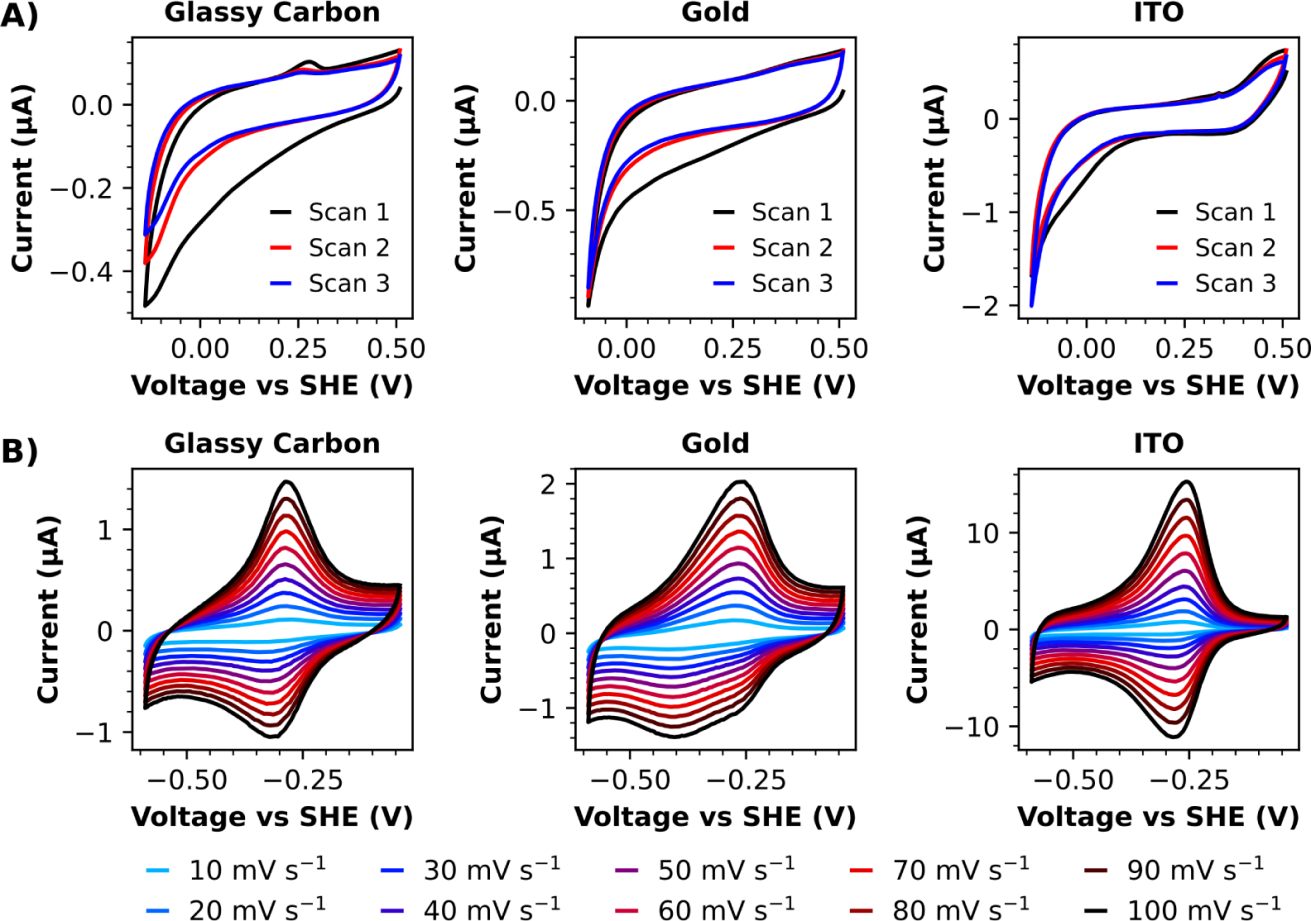
A) Cyclic voltammetry (scan rate = 20 mV s^–1^) of UV-treated solutions of **10** using glassy carbon, gold, and ITO working electrodes. A broad reductive wave, characteristic of multilayer aryl diazonium electrografting, can be observed in scan 1 for each working electrode. B) Cyclic voltammetry of electrode surfaces derivatized via electrografting of UV-treated solutions of **10** revealed redox signals attributable to surface-confined viologen motifs.

UV-treated 1 mM solutions of **10** in pH 8 buffer were subjected to cyclic voltammetry over a mild potential window using glassy carbon, gold, and indium tin oxide (ITO) electrodes. Broad reductive waves were observed in scan 1 in all cases, as is characteristic of diazonium electrografting (Figure 7A). After successively washing and sonicating the derivatized electrode surfaces in both water and DMSO, cyclic voltammograms were recorded in fresh pH 8 buffer over a potential window suitable for observing redox couple 1 (Scheme 4) of the viologen motifs. These redox signals were clearly in evidence for each electrode material trialed (Figure 7B), and the surface-confined nature of these redox couples was further verified by the linear relationship between extracted peak currents and the scan rate (Figure S121).

**Figure 8:**
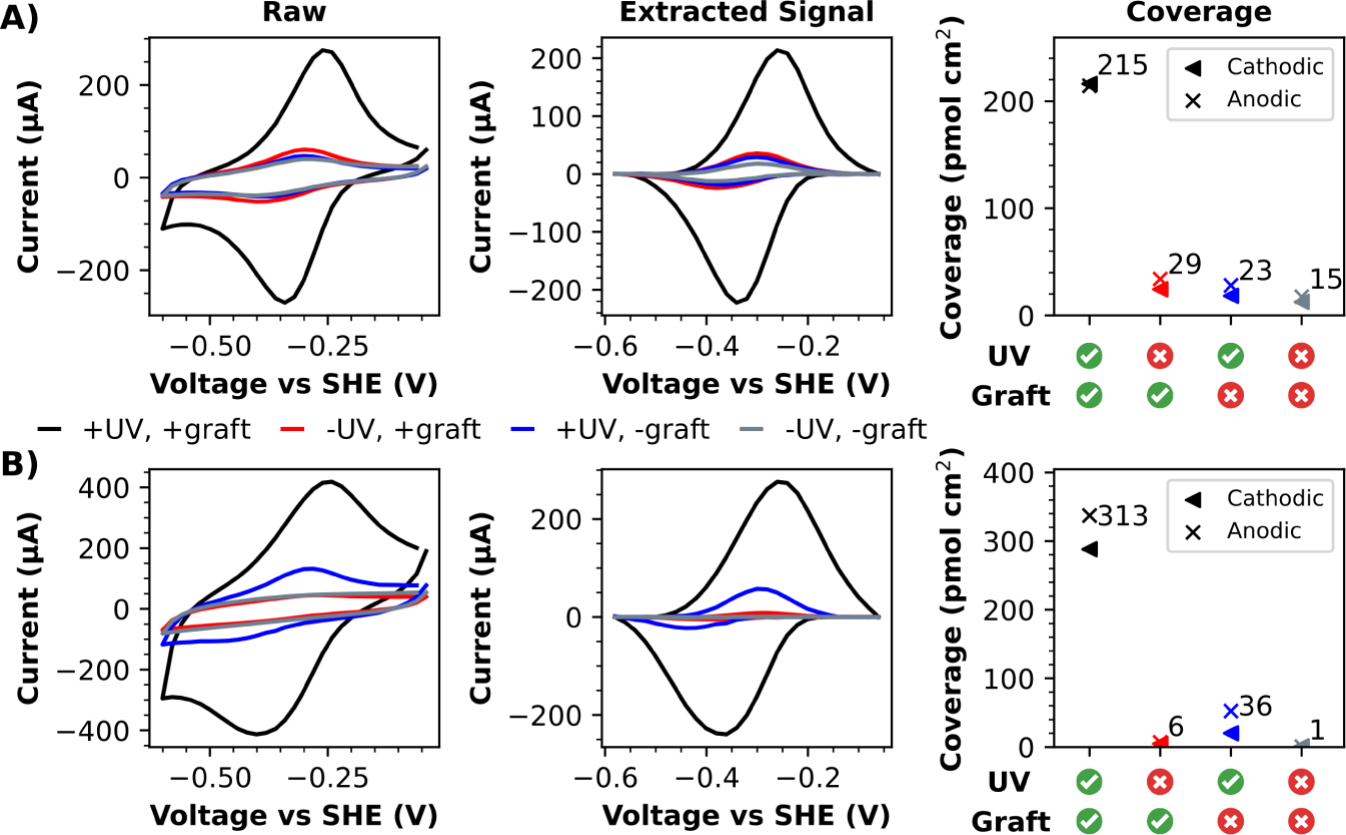
CVs of A) glassy carbon and B) gold electrode surfaces derivatized by using **10** reveal redox signals attributable to immobilized viologen motifs. An approximate extraction of these Faradaic signals was conducted via baseline subtraction, and the approximate coverage of electroactive viologen motifs was then calculated via integration (as described by eq S4). CVs were recorded in pH 8 buffer at a scan rate of 24 V s^–1^.

Having validated that UV-exposed samples of **10** can be used to electrograft viologen motifs onto electrode surfaces, gold and glassy carbon disk electrodes were used to perform experiments with **10** covering all permutations of UV exposure and electrografting (Figure 8). These experiments clearly show that viologen-derived redox signals are by far the most intense when solutions of **10** are subjected to both UV exposure and electrografting, and are least intense when solutions of **10** were not subjected to either UV exposure or diazonium electrografting (Figure 8). The presence of viologen-derived redox signals on electrodes subjected to electrografting with non-UV-treated samples of **10** is likely attributable to the presence of trace amounts of diazonium cations being released from **10** via ambient triazabutadiene degradation. The appearance of viologen-derived redox signals on electrodes treated with UV-exposed samples of **10**, but not subjected to electrografting, can likely be attributed to the spontaneous grafting of diazonium salts, which has been well-documented previously.^47,48^ Regardless, our findings strongly support the hypothesis that UV-treated samples of **10** install viologen functionality onto electrode surfaces via the grafting of diazonium salts.

**Scheme 5:**
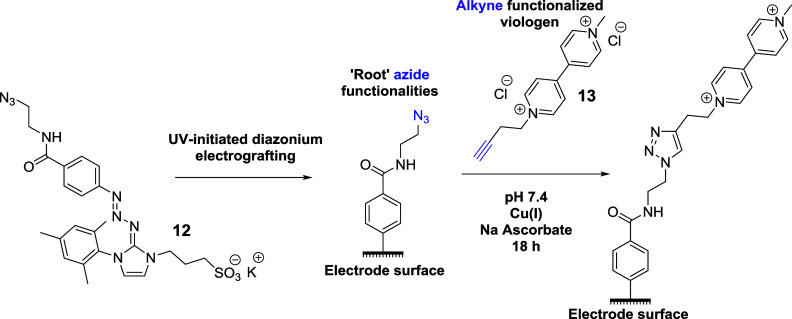
Introduction of Viologen Motifs onto Electrode Surfaces via a Two-Part Strategy whereby Azide Functionalities Are First Introduced onto the Electrode Surface via UV-Initiated Diazonium Electrografting and Are then Coupled to an Alkyne Functionalized Viologen Probe via the Use of the Facile CuAAC Reaction

In order to demonstrate the added value of being able to directly address desired organic motifs onto electrode surfaces in their entirety, for comparative purposes, we also decided to immobilize viologen motifs onto the electrode surface using a more classical two-part coupling approach. To achieve this, we synthesized triazabutadiene **12** and used UV-initiated diazonium electrografting to install azides onto glassy carbon and gold electrode surfaces (Figure S120). We then coupled a terminal alkyne-functionalized viologen probe to this simple “root” moiety via copper-catalyzed azide–alkyne cycloaddition (CuAAC), allowing the coupling reaction to take place over an 18 h period (Scheme 5). We chose to use CuAAC as our coupling strategy instead of amide bond formation to derivatize electrode surfaces, as this reaction is famed for being robust and rapid and is thus representative of a near “best-case” example of coupling to derivatized electrode surfaces.

**Figure 9:**
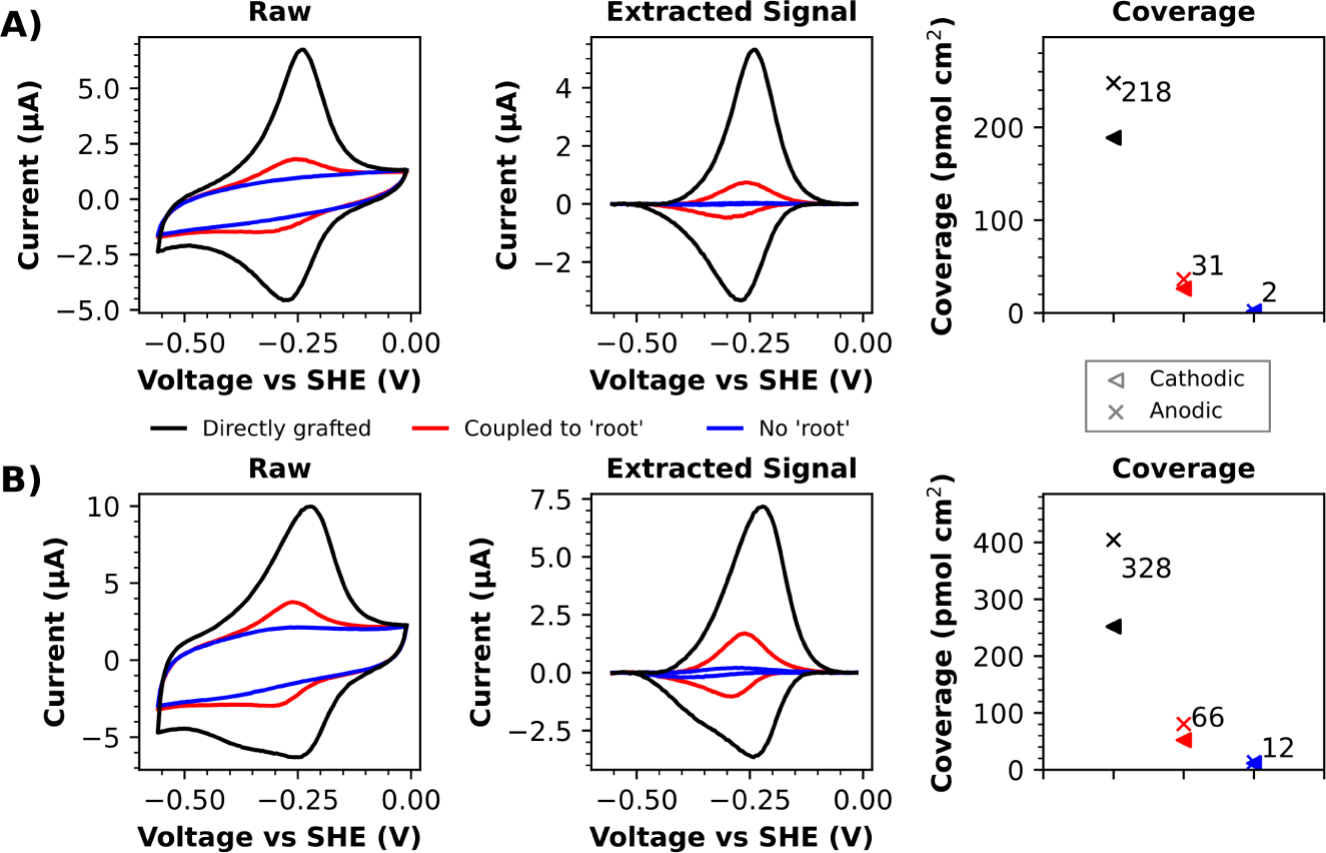
CVs of A) glassy carbon and B) gold electrode surfaces derivatized with viologen motifs either via UV-initiated diazonium electrografting of **10** (directly grafted) or by using UV-initiated diazonium electrografting of **12** to first install “root” azide functionalities to which alkyne-functionalized viologen **13** were subsequently coupled via CuAAC (coupled to “root”). An appropriate control experiment was also conducted in which **12** was not used to install azide functionalities onto the electrode surface prior to exposure to a CuAAC reaction solution of **13** (No “root”). An approximate extraction of these Faradaic signals was conducted via baseline subtraction, and the approximate coverage of electroactive viologen motifs was then calculated via integration (as described by eq S4). CVs were recorded in a pH 8 buffer at a scan rate of 0.4 V s^–1^.

As is evident in Figure 9, directly addressing viologen motifs on electrode surfaces via diazonium electrografting results in greater electroactive coverage than that obtained via the two-part coupling strategy. The electrografting of aryl diazonium cations commonly results in the formation of multilayers;^12−16,42−46^ therefore, electrografting using **10** will have probably resulted in a multilayer coverage of immobilized viologen motifs. In the case of the two-part strategy, we speculate that the lower coverage is due to **13** only being able to couple to a subpopulation of azide motifs within/on the surface of the multilayer derived from **12** (i.e., azide motifs that were relatively sterically unhindered). It could also be speculated that the negative poise applied to the working electrode during electrografting may help facilitate the approach of positively charged species (i.e., viologen-functionalized aryl diazonium cations), even as positively charged viologen motifs become immobilized on the electrode surface. Conversely, during the two-part coupling strategy, any accumulation of positive charge (from immobilized viologen motifs) on the electrode surface would be uncompensated for, and thus the progression of the coupling reaction may become hindered by electrostatic repulsions between the derivatized electrode surface and **13**. Regardless, it is certainly more time-efficient to directly address the viologen motifs onto the electrode surface via UV-initiated diazonium electrografting of **10**; the electrografting cyclic voltammetry method used took approximately 3 min, yet a comparable coverage of viologen motifs could not be achieved via the two-part coupling strategy, even after 18 h.

## Conclusion

In summary, we have presented a methodology for the functionalization of electrode surfaces via diazonium electrografting from UV-activatable bench-stable triazabutadiene precursors. We show that triazabutadienes are practical, efficient, and conveniently UV-light activatable motifs that allow access to aryl diazonium cations which would be expected to be either difficult to synthesize or difficult to store, and we show that aryl diazonium cations released from triazabutadienes are suitable for diazonium electrografting. We also demonstrate that being able to directly address desired organic motifs onto the electrode surface, instead of having to build motifs onto the surface via successive coupling reactions as part of a multistep approach, can result in a greater coverage of these motifs on the electrode surface. While we have not attempted to limit the formation of multilayers during the electrografting experiments reported in this paper, many techniques already exist for controlling grafted film thickness—i.e., the use of bulky/aromatic protecting groups,^12,13,15,42−45,49^ cross-redox reactions,^50^ electrochemically generated acids,^51^ or the careful optimization of grafting conditions via modulation of aryl diazonium cation concentration^46^ and the total reductive current passed during electrografting.^2,52^ We anticipate that any of these techniques for modulating electrografting could readily be used in conjunction with our triazabutadiene-based approach.

The mild, light-activated nature of aryl diazonium cation release, coupled with the biocompatible conditions employed during this study, means we foresee an exciting future for this technology, such as in the development of procedures via which triazabutadienes could be used to facilitate the immobilization of biomolecules.

## Supporting Information Available

Detailed experimental/analytical procedures, characterization data (i.e., XPS, NMR, ESI MS, FTIR), supplementary figures, equations, and associated references^53−60^ (PDF)

## Abbreviations

CV: cyclic voltammogram
CuAAC: copper-catalyzed azide–alkyne cycloaddition
UV: ultraviolet
XPS: X-ray photoelectron spectroscopy
DMSO: dimethyl sulfoxide
