# Supplementary material for: Using Triazabutadienes as a Protected Source of Diazonium Cations to Facilitate Electrografting to a Variety of Conductive Surfaces
Source: Langmuir. 2025 Mar 12;41(11):7386–95. doi: 10.1021/acs.langmuir.4c04848 (PMC11948462; doi:10.1021/acs.langmuir.4c04848)
Supplement: Supplementary file 1 — la4c04848_si_001.pdf [file la4c04848_si_001.pdf]

Supporting Information:

Using Triazabutadienes as a Protected Source of Diazonium Cations to Facilitate Electrografting to a Variety of Conductive Surfaces

Nicholas D. J. Yates,[1] Lucy Hudson,[1] Oscar Schwabe,[1] Alison Parkin*[1]

[1]Department of Chemistry, University of York, Heslington, York, YO10 5DD, U.K.

*Email: alison.parkin@york.ac.uk

Contents

Synthesis and characterisation of small molecules S4

General considerations S4

Compounds S5

1-mesityl-1H-imidazole **1** S5

3-(1-mesityl-1H-imidazol-3-ium-3-yl)propane-1-sulfonate **2** S10

4-azido-N-(2,2,2-trifluoroethyl)benzamide **3** S15

Tetrabutylammonium 3-((Z)-3-mesityl-2-((E)-(4-((2,2,2-trifluoroethyl)carbamoyl)phenyl)triaz-2-en-1-ylidene)-2,3-dihydro-1H-imidazol-1-yl)propane-1-sulfonate **4** S21

4-amino-N-(2,2,2-trifluoroethyl)benzamide **S1** S30

4-((2,2,2-trifluoroethyl)carbamoyl)benzenediazonium tetrafluoroborate **5** S36

Methyl 4-azidobenzoate **6** S42

Tetrabutylammonium 3-((Z)-3-mesityl-2-((E)-(4-(methoxycarbonyl)phenyl)triaz-2-en-1-ylidene)-2,3-dihydro-1H-imidazol-1-yl)propane-1-sulfonate **7** S42

Potassium 4-((E)-((E)-1-mesityl-3-(3-sulfonatopropyl)-1,3-dihydro-2H-imidazol-2-ylidene)triaz-1-en-1-yl)benzoate **8** S48

1-methyl-[4,4'-bipyridin]-1-ium iodide **S2** S53

1-methyl-[4,4'-bipyridin]-1-ium hexafluorophosphate **S3** S58

1-(2-ammonioethyl)-1'-methyl-[4,4'-bipyridine]-1,1'-diium hexafluorophosphate **9** S65

3-((Z)-3-mesityl-2-((E)-(4-((2-(1'-methyl-[4,4'-bipyridin]-1,1'-diium-1-yl)ethyl)carbamoyl)phenyl)triaz-2-en-1-ylidene)-2,3-dihydro-1H-imidazol-1-yl)propane-1-sulfonate hexafluorophosphate **10** S73

2-azidoethan-1-aminium chloride **11** S81

Preparation of potassium tert-butoxide treated silica **S4** S86

Potassium 3-((Z)-2-((E)-(4-((2-azidoethyl)carbamoyl)phenyl)triaz-2-en-1-ylidene)-3-mesityl-2,3-dihydro-1H-imidazol-1-yl)propane-1-sulfonate **12** S87

1-(but-3-yn-1-yl)-[4,4'-bipyridin]-1-ium hexafluorophosphate **S5** S93

1-(but-3-yn-1-yl)-1'-methyl-[4,4'-bipyridine]-1,1'-diium hexafluorophosphate **S6** S100

1-(but-3-yn-1-yl)-1'-methyl-[4,4'-bipyridine]-1,1'-diium chloride **13** S107

3-(2-iminio-3-mesityl-2,3-dihydro-1H-imidazol-1-yl)propane-1-sulfonate **S7** S113

UV-vis analysis of 4 ..... S118

General buffers: S118

Half-life measurements of **4** S118

UV-triggered aryl diazonium release from **4** S118

Electrochemistry S119

General considerations S119

General buffers: S119

Electrodes and electrode preparation steps S120

3 mm Disk Working Electrodes: S120

Screen Printed Electrodes: S120

Gold-Coated Silicon Wafers: S121

ITO-Coated Glass Slides: S121

Ag/AgCl (3 M NaCl) Reference Electrode S121

Platinum Wire Counter Electrode S121

Experimental procedures S122

Grafting **4**/**5** onto screen-printed electrodes (SPEs): S122

Grafting **4**/**5** onto 3 mm disk glassy carbon electrodes: S123

Grafting **4**/**5** onto gold-coated silicon wafer slices: S124

Aliquoting of **10**: S126

Exploring the grafting behaviour of **10** using 3 mm disk working electrodes: S126

Exploring the grafting behaviour of **10** using ITO-coated glass slides: S127

Grafting **12** onto a 3 disk working electrodes: S130

Isolation and analysis of viologen signals S132

Determination of surface-confined nature of redox couple S133

Calculation of surface coverage S133

Investigating potential origins of the attenuated diazonium electrografting behaviour of 4 relative to 5 ..... S134

Experimental S135

Preparing various solutions of **4**/**5** of **S7** S135

Grafting various solutions of **4**/**5** and **S7** onto 3 mm disk glassy carbon electrodes S136

UV-vis analysis of solutions of **4**/**5** and **S7** S136

NMR analysis of solution **5** + **S7** S136
Results S136
X-ray Photoelectron Spectroscopy (XPS) S140
Acknowledgments S140
Experimental S140
Analysis S140
XPS data S141

Synthesis and characterisation of small molecules

General considerations

Reagents were purchased from Sigma-Aldrich and used as supplied. Anhydrous solvents were dried over a PureSolv MD 7 Solvent Purification System. Anhydrous solvents were used in reactions unless otherwise stated, or where aqueous/organic cosolvent mixtures were employed. GPR-grade solvents were used for flash chromatography purposes. Solution-phase synthetic reactions were carried out using oven-dried glassware. All concentrations were performed *in vacuo* unless otherwise stated. Thin layer chromatography was carried out on Merck silica gel 60 F254 precoated aluminium foil sheets and these were visualized using UV light (254 nm) and/or PPh_3 (10% in DCM) and/or ninhydrin (1.5% ninhydrin, 3% AcOH in n-butanol). Unless otherwise indicated, flash column chromatography was performed on Supelco® silica gel (particle size 35–75 µm, pore diameter 60 Å, 220-440 mesh) and the solvent system used is recorded in parentheses.

Proton and carbon nuclear magnetic resonance (^{1}H- and ^{13}C-NMR respectively) spectra were recorded on either a Jeol ECX-400 (400 MHz) or a Bruker AVIIIHD500 (500 Mz) spectrometer. Assignments of NMR spectra were conducted on MestReNova, using additional NMR experiments including ^{1}H-^{1}H-COSY, DEPT and ^{1}H-^{13}C-HMQC spectra when necessary. All chemical shifts are quoted on the δ scale in ppm using residual solvent as the internal standard (^{1}H-NMR: $CDCl_3$ = 7.26; methanol-d_4 = 3.31; D_2O = 4.69; DMSO-d_6 = 2.50 and ^{13}C-NMR: $CDCl_3$ = 77.16, methanol-d_4 = 49.00, DMSO-d_6 = 39.52). Coupling constants (*J*) are reported in Hz with the following splitting abbreviations: s = singlet, d = doublet, t = triplet, q = quartet, m = multiplet, app = apparent, br = broad. $m^{AA'BB}$ refers to the multiplet pattern observed for 1,4-disubsituted aryl systems.

Small-molecule high resolution mass spectrometry (HRMS) data were obtained at RT on a Bruker Daltonics microTOF mass spectrometer coupled to an Agilent 1200 series LC system at The University York Centre of Excellence in Mass Spectrometry (CoEMS). The running solvent was a 1:1 (v/v) mixture of HPLC grade water and HPLC grade methanol, and samples were dissolved in an appropriate solvent prior to injection. No column was used. Nominal and exact m/z values are reported in Daltons.

Compounds

1-mesityl-1H-imidazole 1

This synthesis was adapted from refs[53, 54].

A stirred solution of 37% aqueous glyoxal (4.6 mL) and 37% aqueous formaldehyde (3 mL) in glacial acetic acid (10 mL) was heated to 70 °C, whereupon a solution of 2,4,6-trimethylaniline (5.39 g, 40.0 mmol), water (2 mL), and ammonium acetate (3.08 g, 40.0 mmol) in glacial acetic acid (10 mL) was added dropwise. The resultant solution was stirred at 70 °C for 18 h, after which time the solution was cooled to room temperature and slowly added to a stirred solution of $NaHCO_3$ (29.4 g) in water (300 mL). The product was then removed by filtration, dried *in vacuo* and purified by flash column chromatography (hexane → EtOAc) to yield **1** as a light brown microcrystalline solid (2.88 g, 39%). This product has a distinctively earthly aroma.

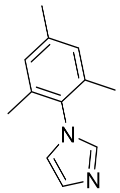

Figure S 1. The structure of **1**.

^{1}H-NMR (400 MHz, $CDCl_3$): δ_H 7.43 (dd, *J* = 1.00, 1.00 Hz, 1H), 7.22 (dd, *J* = 1.20, 1.00 Hz, 1H), 6.96 (s, 2H), 6.88 (dd, *J* = 1.20, 1.00 Hz, 1H), 2.33 (s, 3H), 1.98 (s, 6H).

^{13}C-NMR (101 MHz, $CDCl_3$): δ_c 138.9, 137.6, 135.5, 133.5, 129.7, 129.1, 120.2, 21.1, 17.4.

FT-IR (ATR) ($\upsilon max/cm^{-1}$): 3096 (C-H stretch), 2926 (C-H stretch), 1499, 816 (C-H bend, aromatic).

(ESI)HRMS: Found 187.1232, $C_{12}H_{15}N_2$ requires 187.1230.

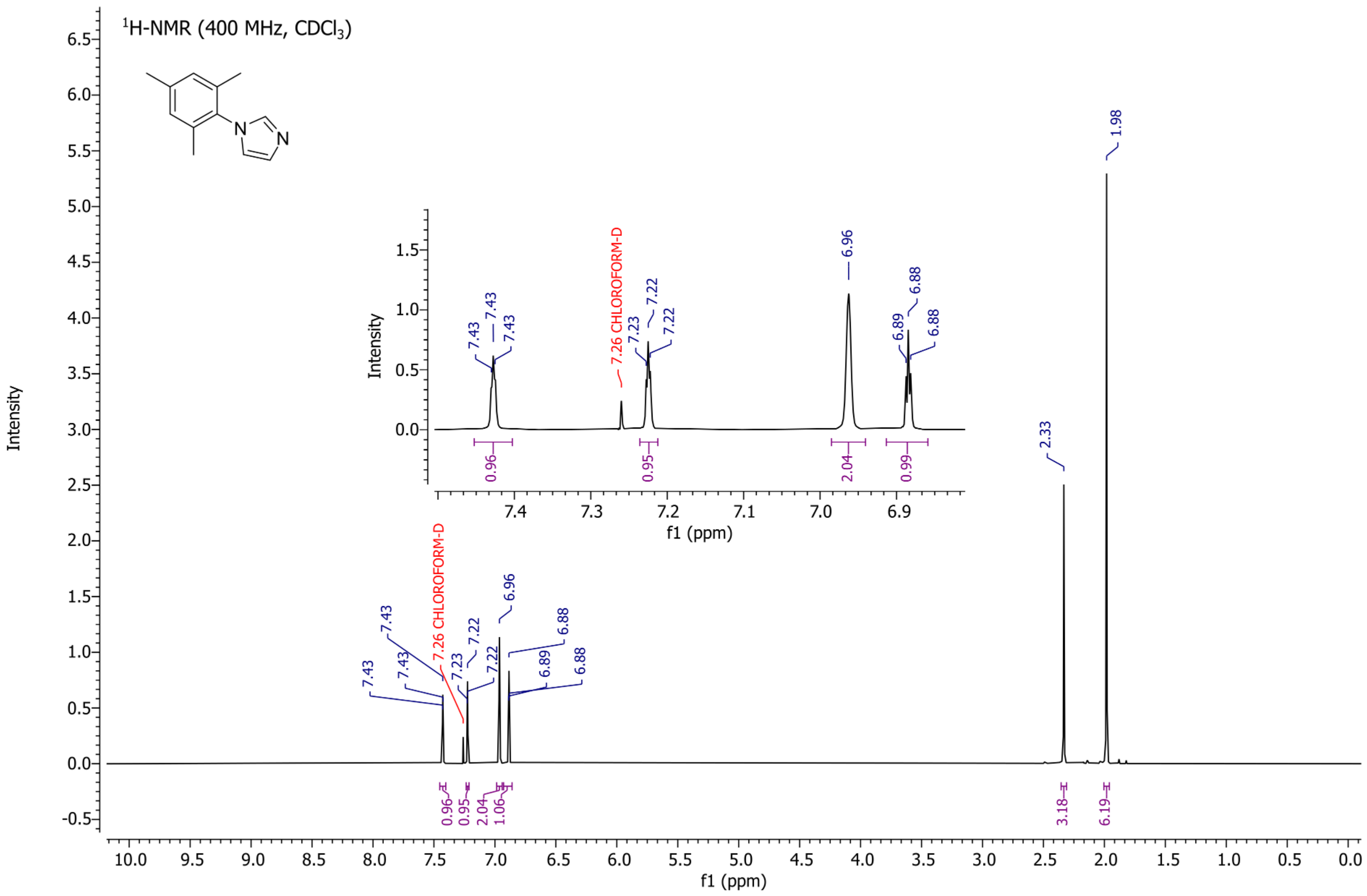

Figure S 2. ^{1}H-NMR spectrum of 1-mesityl-1H-imidazole **1**.

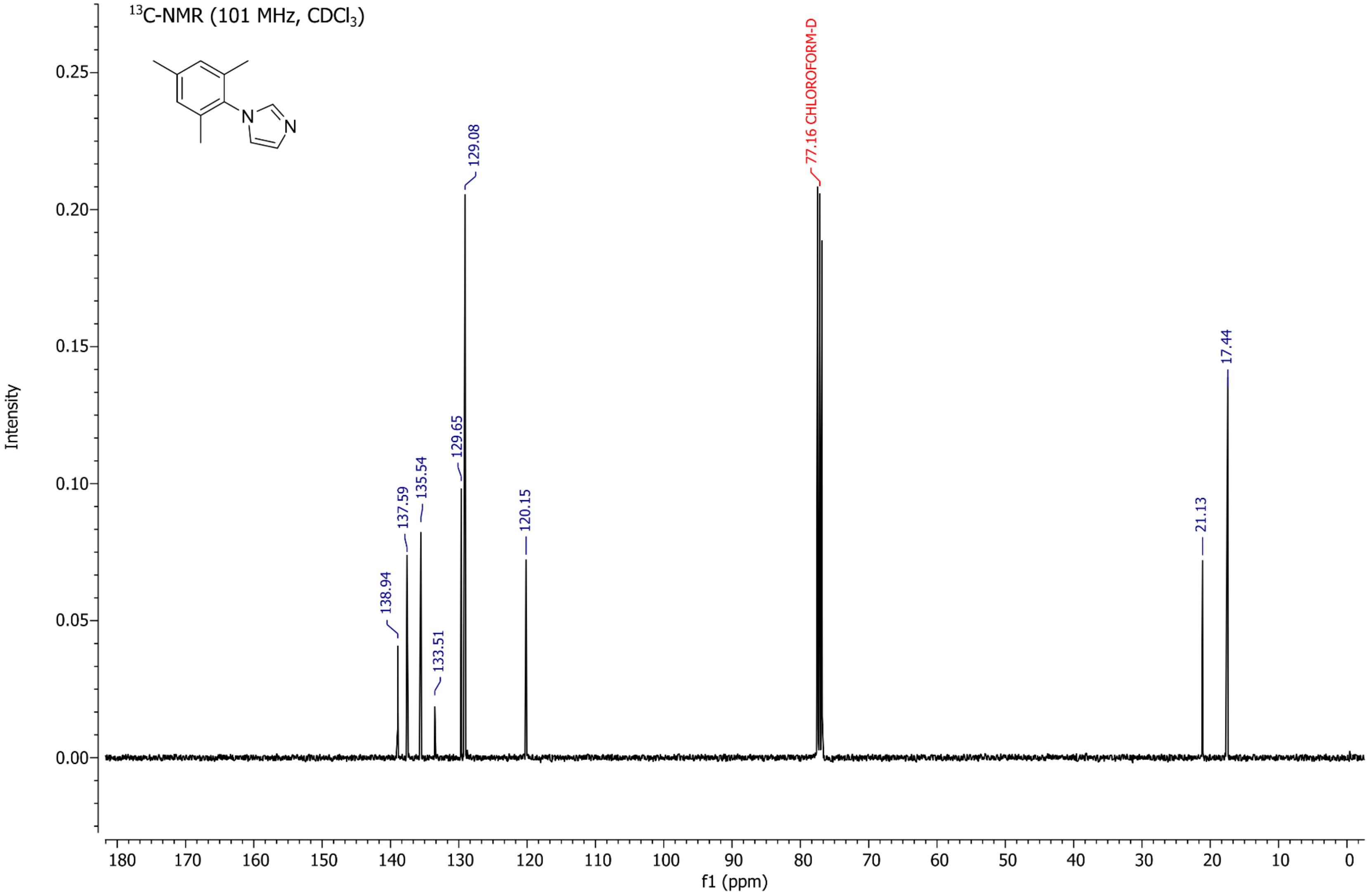

Figure S 3. ^{13}C-NMR spectrum of 1-mesityl-1H-imidazole **1**.

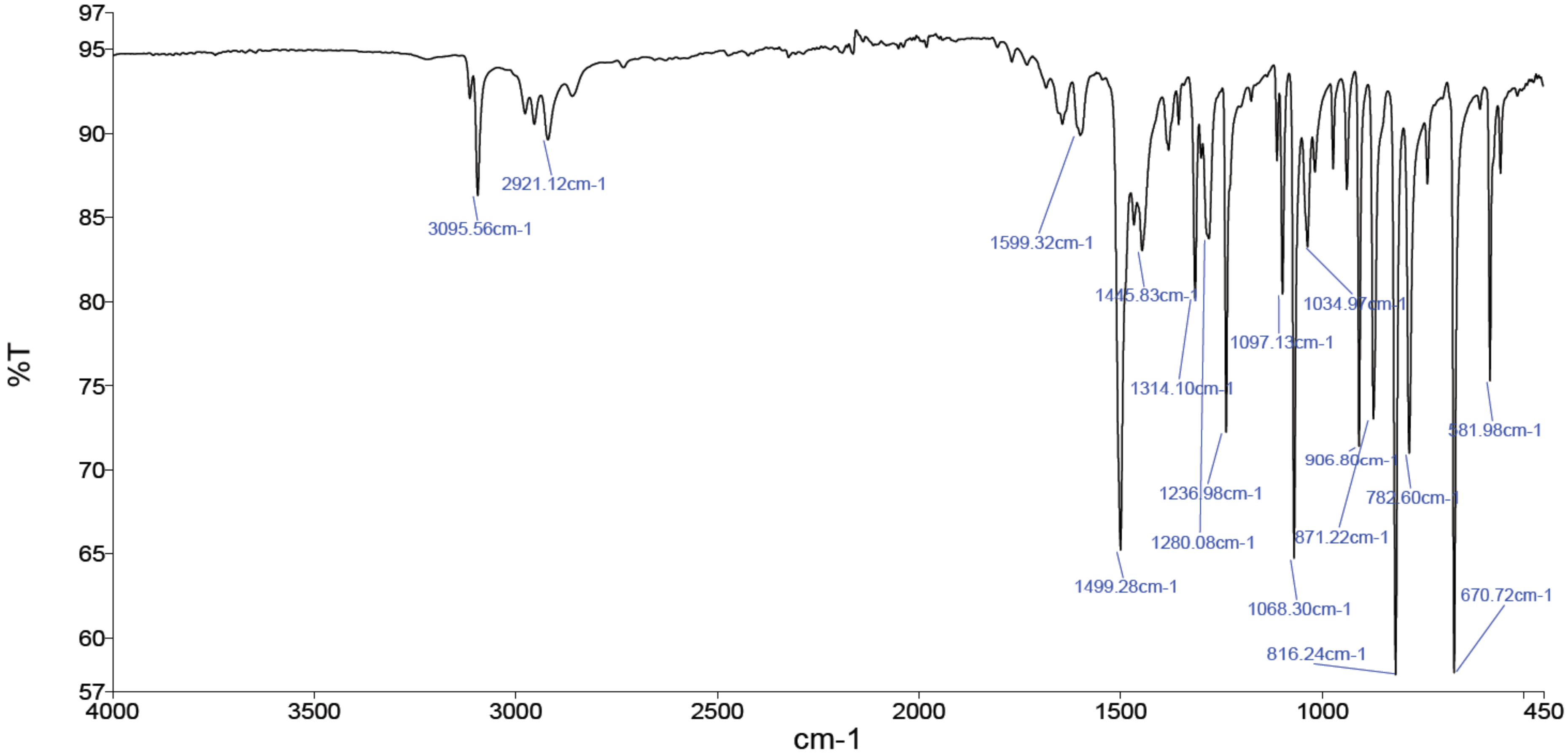

Figure S 4. FT-IR (ATR) spectrum of 1-mesityl-1H-imidazole **1**.

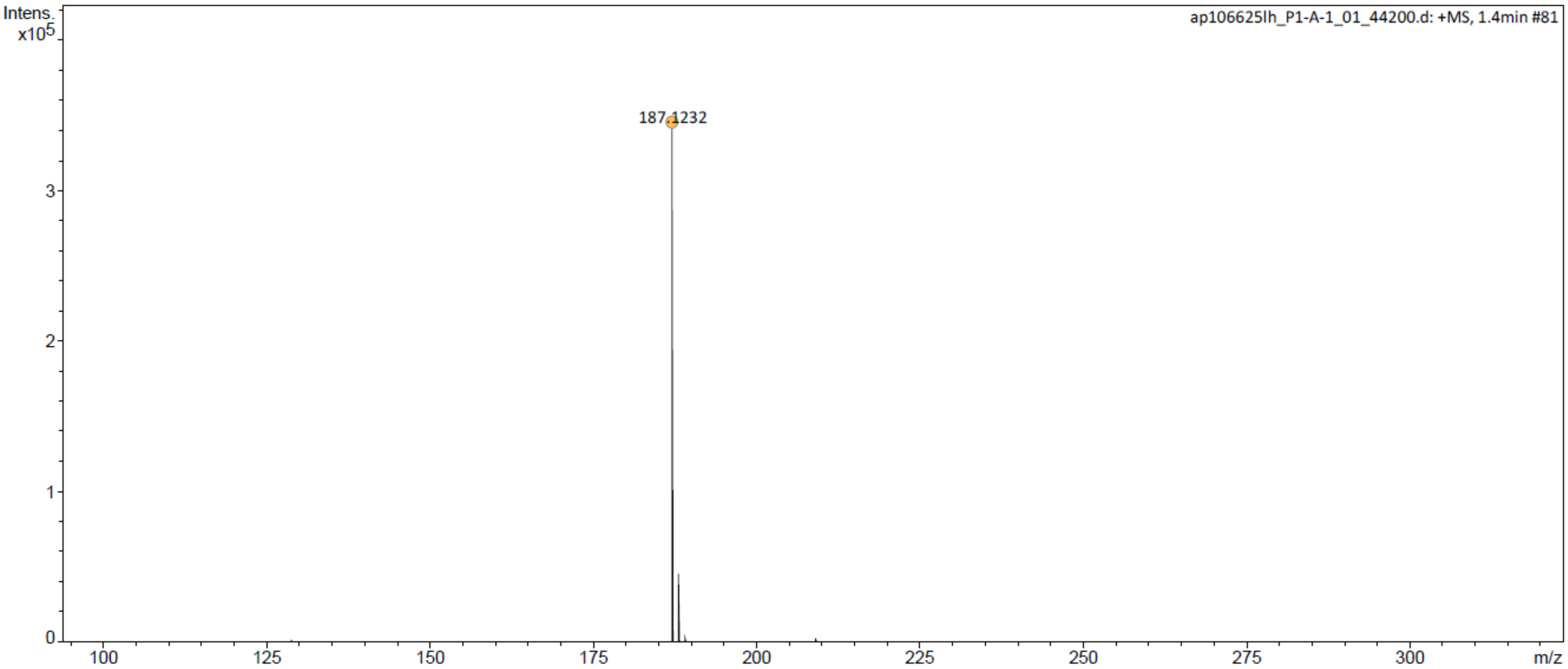

Meas. m/z	#	Ion Formula	m/z	err [ppm]	err [mDa]	mSigma	Mean err [ppm]
187.1232	1	C12H15N2	187.1230	-1.4	-0.3	3.1	-1.9

Figure S 5. (ESI)HRMS of 1-mesityl-1H-imidazole **1**.

3-(1-mesityl-1H-imidazol-3-ium-3-yl)propane-1-sulfonate 2

To a stirred solution of **1** (1.00 g, 5.4 mmol) in dry toluene (30 mL) was added 1,3-propanesultone (1.00 g, 8.2 mmol, 1.5 equiv). The resultant solution was refluxed overnight, after which time the solution was allowed to cool to room temperature. The product was then collected by filtration and washed with diethyl ether and cold acetone before being dried *in vacuo*. This yielded **2** as a light grey powder (1.62 g, 98%). Characterisations matched those of the literature.[55,56]

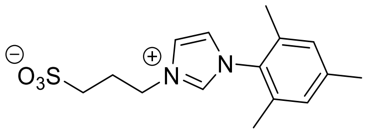

Figure S 6. The structure of **2**.

^{1}H-NMR (400 MHz, D_2O): δ_H 8.98 (dd, *J* = 1.80 Hz, 1H), 7.80 (dd, *J* = 1.80, 1.80 Hz, 1H), 7.55 (dd, *J* = 1.80 Hz, 1H), 7.12 (s, 2H), 4.49 (t, *J* = 7.10 Hz, 2H), 2.93 (t, *J* = 7.70 Hz, 2H), 2.40 (tt, *J* = 7.70, 7.10 Hz, 2H), 2.30 (s, 3H), 2.01 (s, 6H).

^{13}C-NMR (101 MHz, D_2O): δ_c 141.5, 136.6, 134.7, 130.8, 129.3, 124.4, 123.1, 48.2, 47.2, 25.1, 20.2, 16.3.

FT-IR (ATR) (υmax/cm^{-1}): 3121 (C-H stretch), 2979 (C-H stretch), 1610 (C=C stretch, aromatic), 1203 (S=O stretch), 1185 (S=O stretch).

(ESI)HRMS: Found 309.1258, $C_{15}H_{21}N_2O_3S^+$ requires 309.1267; found 331.1077, $C_{15}H_{21}N_2NaO_3S^+$ requires 331.1087.

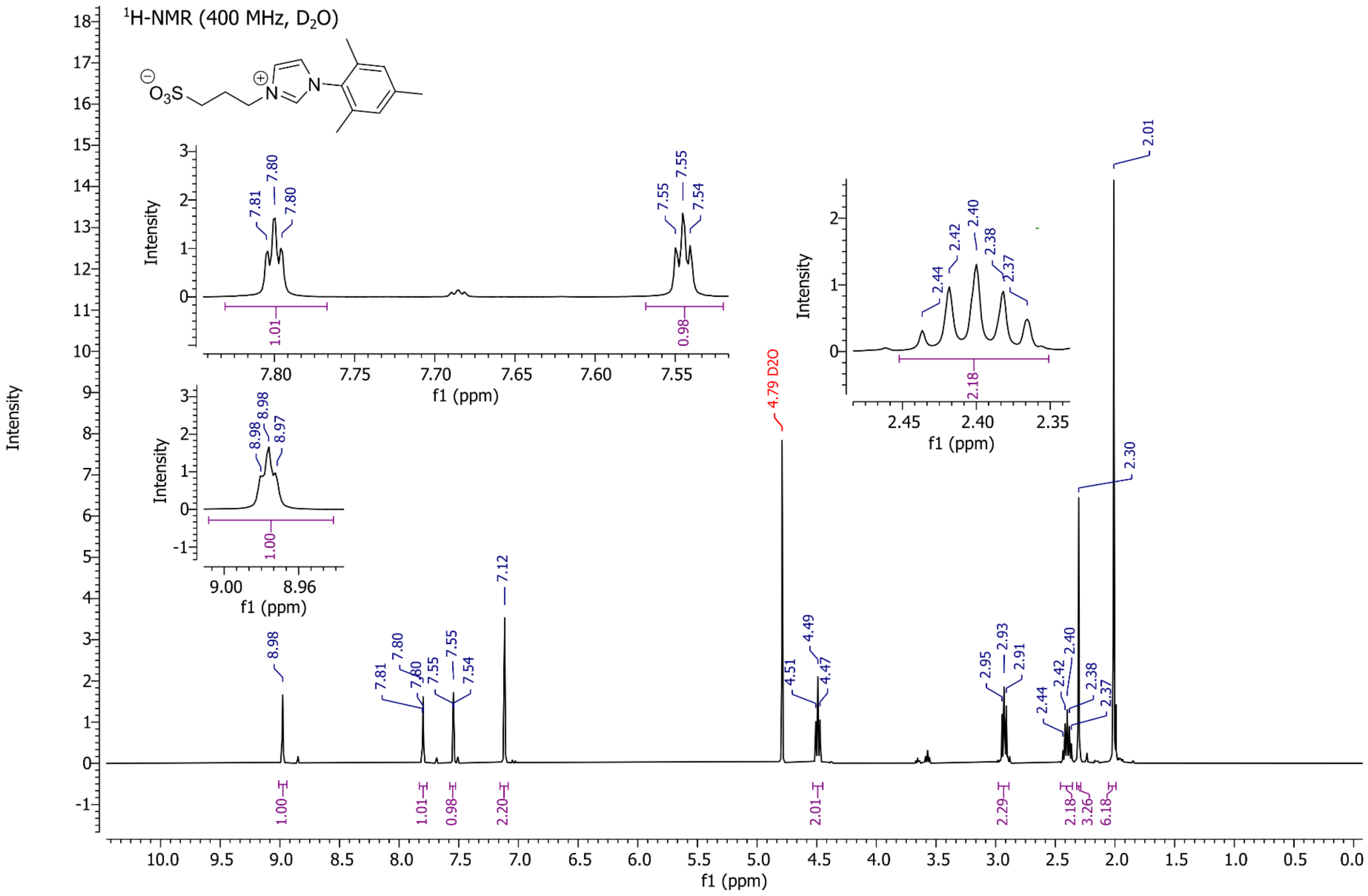

Figure S 7. ^{1}H-NMR spectrum of **2**.

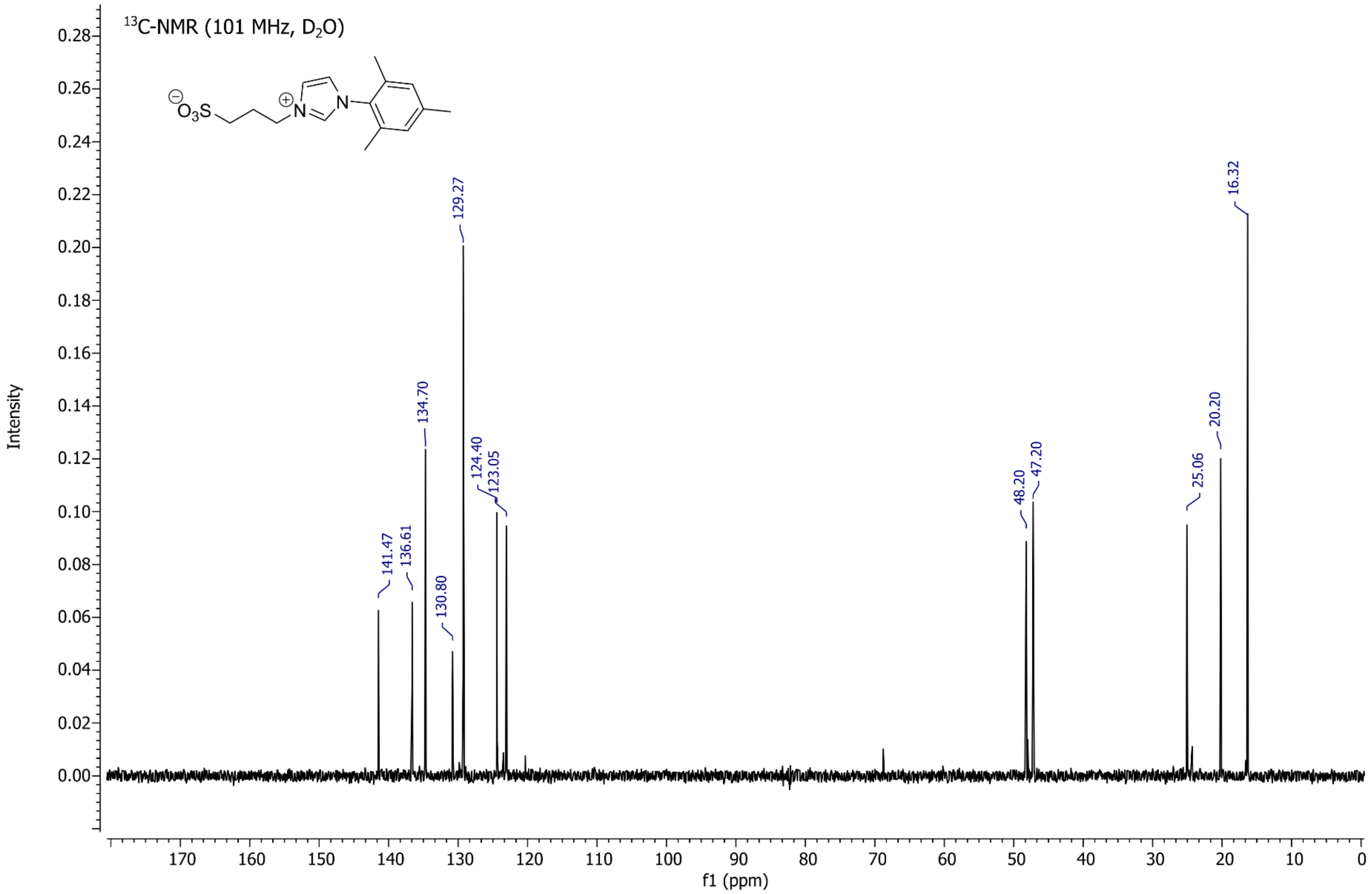

Figure S 8. ^{13}C-NMR spectrum of **2**.

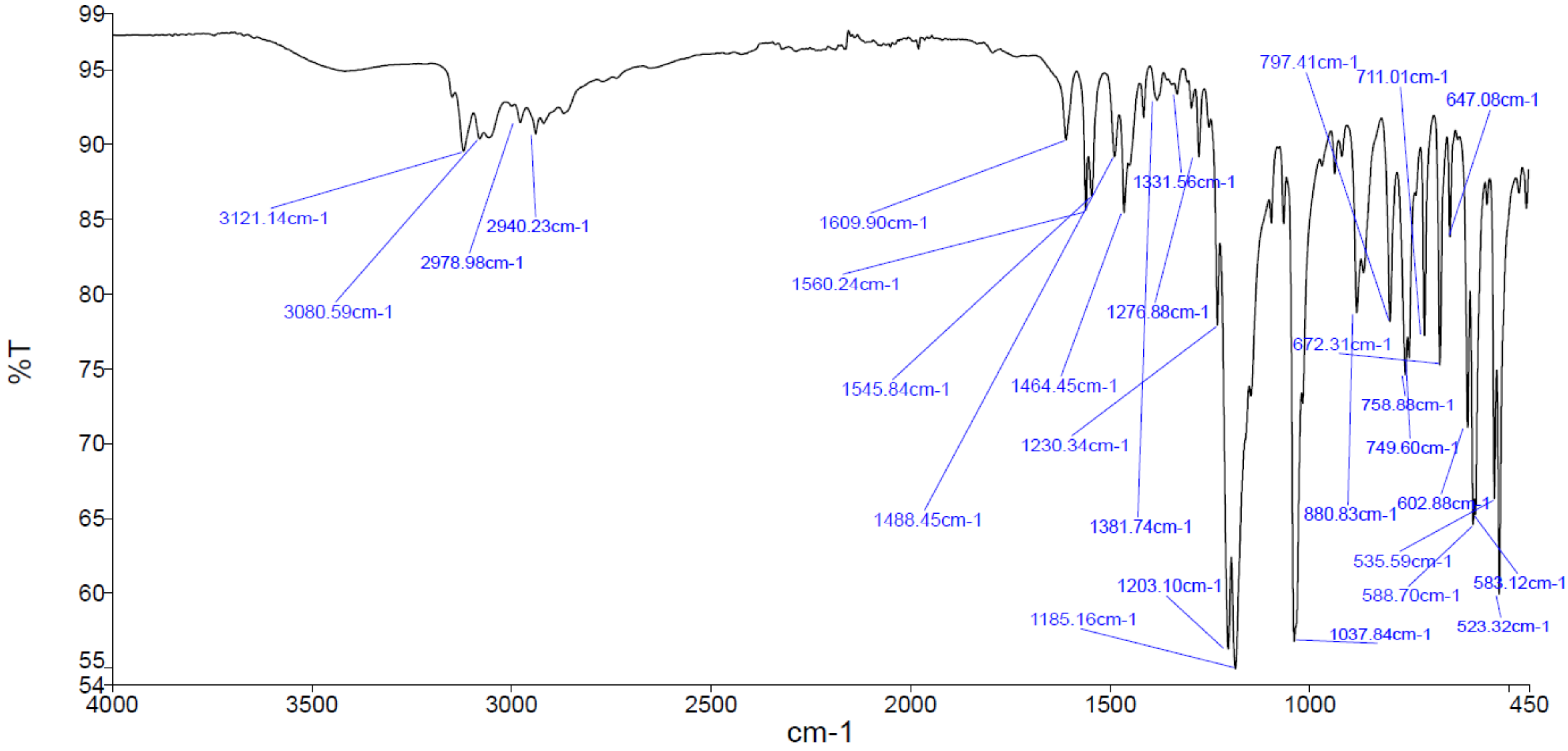

Figure S 9. FT-IR (ATR) spectrum of **2**.

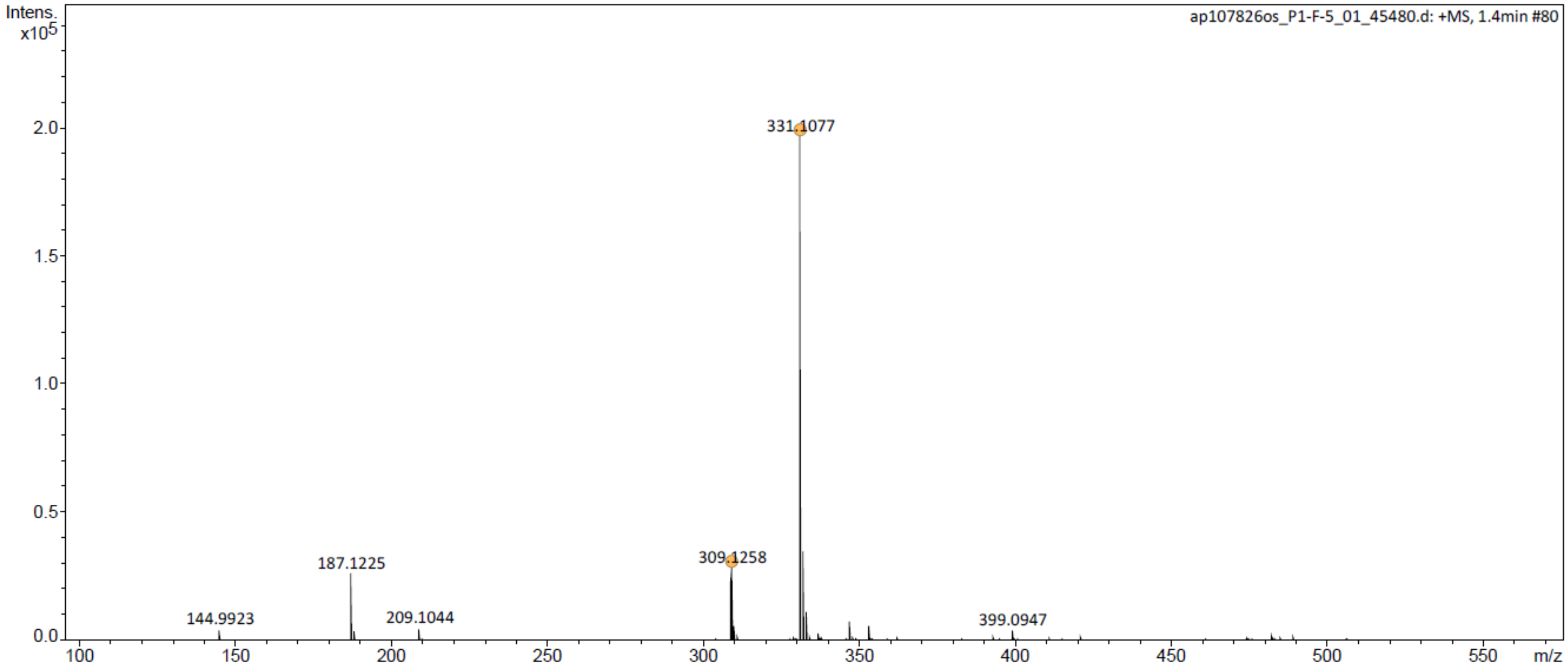

Meas. m/z	#	Ion Formula	m/z	err [ppm]	err [mDa]	mSigma	Mean err [ppm]
309.1258	1	C15H21N2O3S	309.1267	3.1	1.0	12.9	5.0
331.1077	1	C15H20N2NaO3S	331.1087	2.9	1.0	4.8	2.9

Figure S 10. (ESI)HRMS of **2**.

4-azido-N-(2,2,2-trifluoroethyl)benzamide 3

To a suspension of 4-azidobenzoic acid (0.88 g, 5.39 mmol) in DCM (6 mL) under a nitrogen atmosphere was added oxalyl chloride (1 mL, 11.7 mmol), followed by DMF (1 drop). The mixture was then refluxed for 2 h under nitrogen. After this time the solution was allowed to cool to rt and the small amount of solid material present was removed by filtration. The eluate was then concentrated *in vacuo* to yield a pale orange solid which was immediately re-dissolved in DMF (6 mL) before being added dropwise to a solution of 2,2,2-trifluoroethylamine (0.45 mL, 5.73 mmol) and triethylamine (1.86 mL, 13.3 mmol) in DMF (10 mL) at 0°C. The mixture was stirred overnight at rt and was then concentrated *in vacuo*. The resultant residue was purified by silica column chromatography (DCM → 10% MeOH in DCM) to yield 4-azido-N-(2,2,2-trifluoroethyl)benzamide (0.746 g, 57%).

^{1}H-NMR (400 MHz, $CDCl_3$): δ_H 7.83-7.75 (m, 2H), 7.13-7.02 (m, 2H), 6.58 (br t, *J* = 6.45 Hz, 1H), 4.09 (qd, *J* = 9.10, 6.45 Hz, 2H).

^{13}C-NMR (101 MHz, $CDCl_3$): δ_c 166.9, 144.3, 129.6, 129.1, [128.4, 125.6, 122.9, 120.1][*1], 119.2, [41.7, 41.4, 41.0, 40.7][*2].

^{19}F-NMR (376 MHz, $CDCl_3$): δ_F -72.2 (t, $J_{H\text{-}F}$ = 9.10 Hz).

FT-IR (ATR) (υmax/cm^{-1}): 3306 (N-H stretch), 2114 (N=N=N stretch, azide), 1648 (C=O stretch), 1605 (C=C stretch, aromatic).

(APCI)HRMS: Found 245.0627, $C_9H_8F_3N_4O^+$ requires 245.064472.

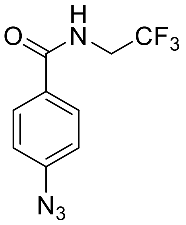

Figure S 11. The structure of **3**.

[*1] These four peaks correspond to a 1:3:3:1 quartet formed due to the ^{13}C-^{19}F coupling of the <u>C</u>F$_3$ environment. Here $J_{C\text{-}F}$ can be measured as 278.5 Hz.

[*2] These four peaks correspond to a 1:3:3:1 quartet formed due to the ^{13}C-^{19}F coupling of the <u>C</u>H$_2$CF$_3$ environment. Here $J_{C\text{-}F}$ can be measured as 34.8 Hz

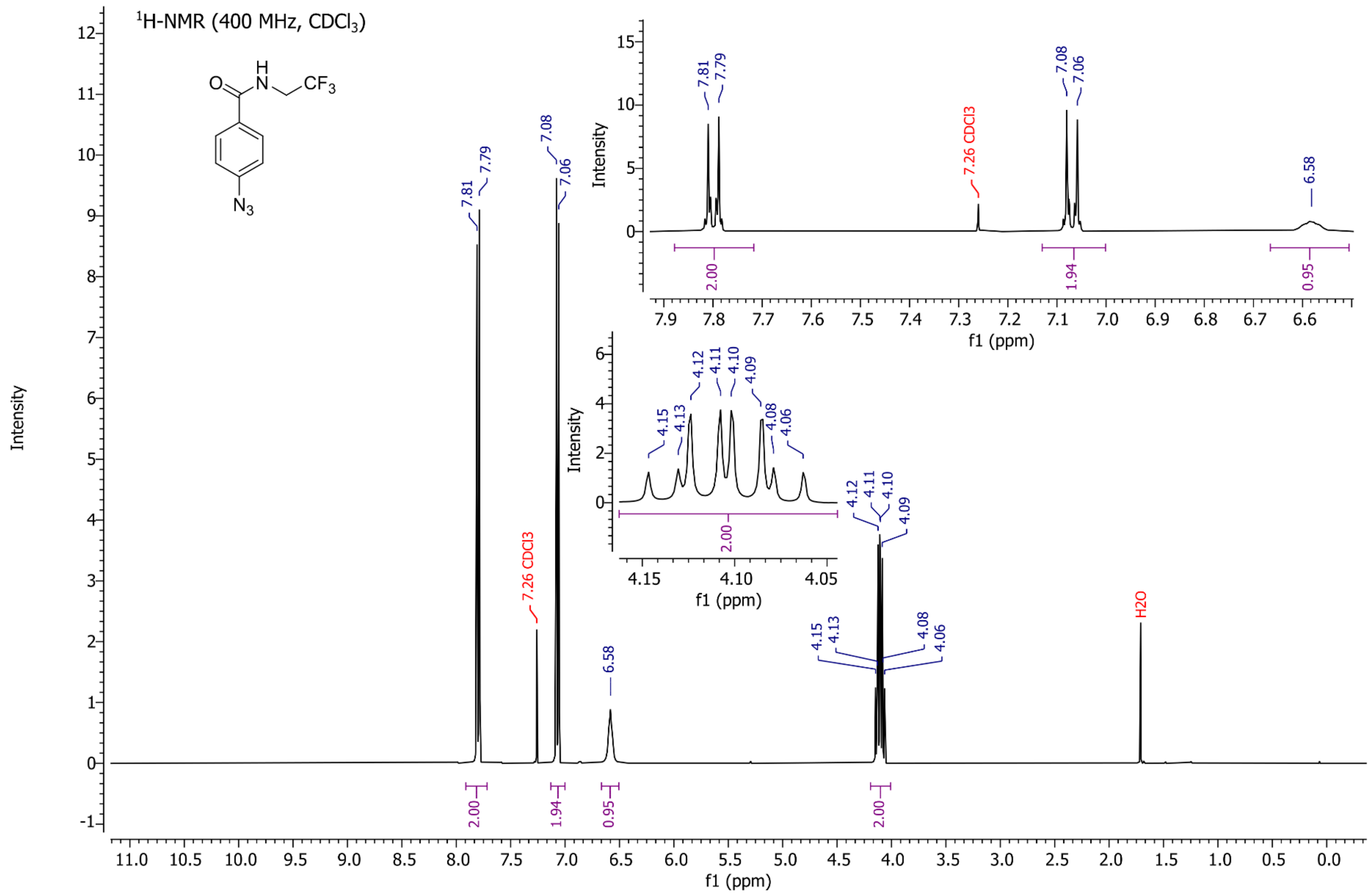

Figure S 12. ^{1}H-NMR spectrum of **3**.

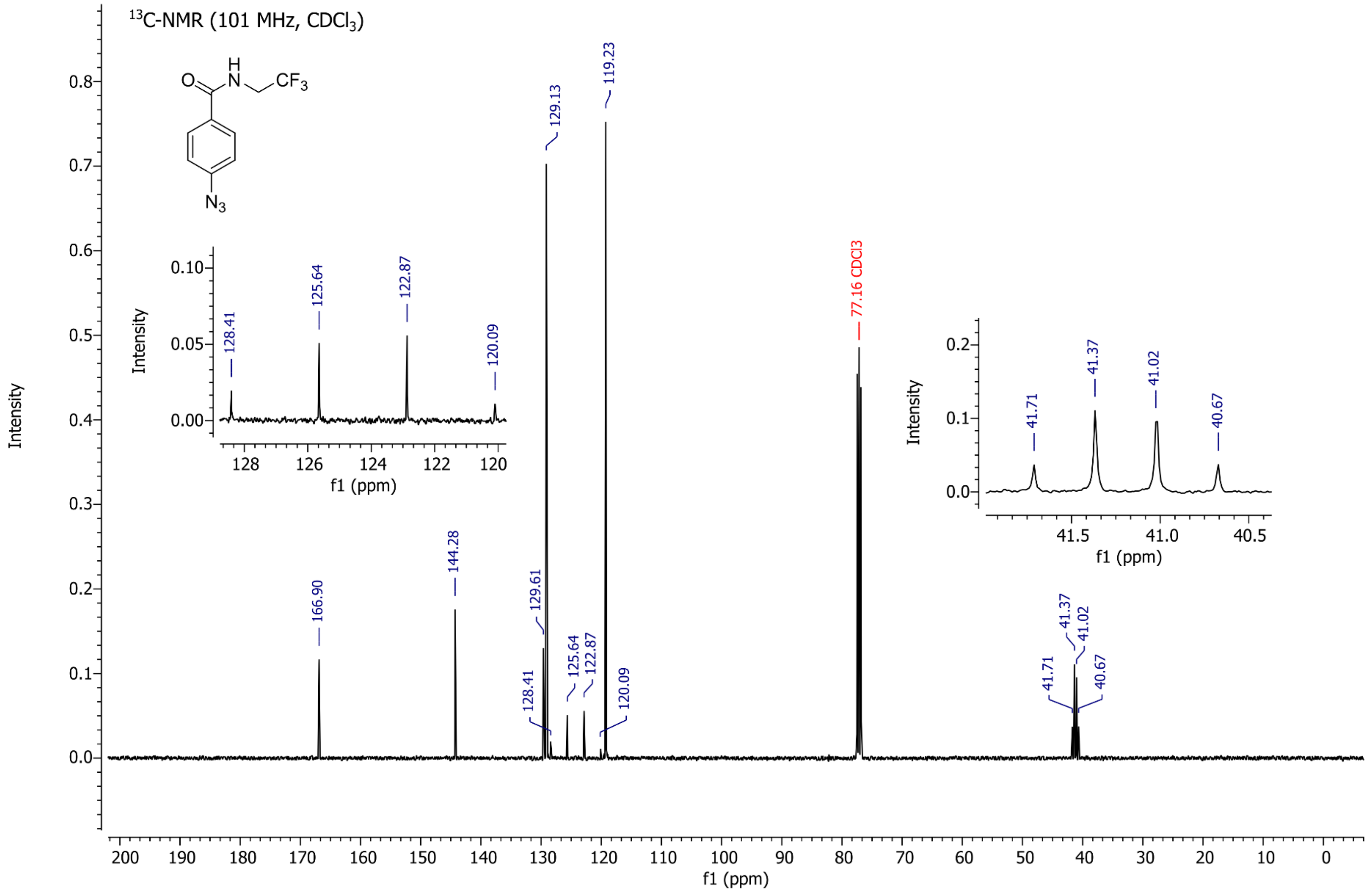

Figure S 13. ^{13}C-NMR spectrum of **3**.

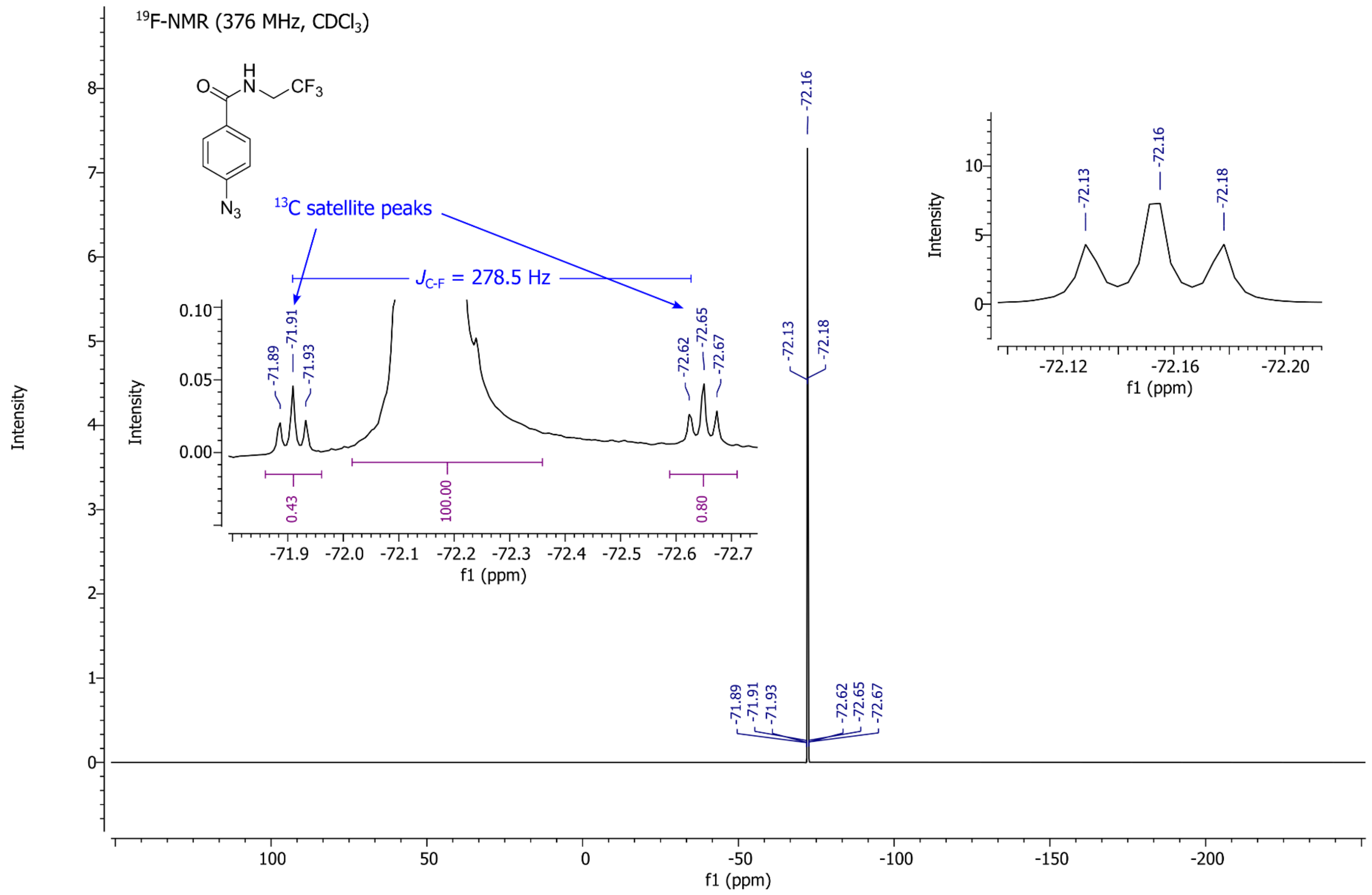

Figure S 14. ^{19}F-NMR spectrum of **3**.

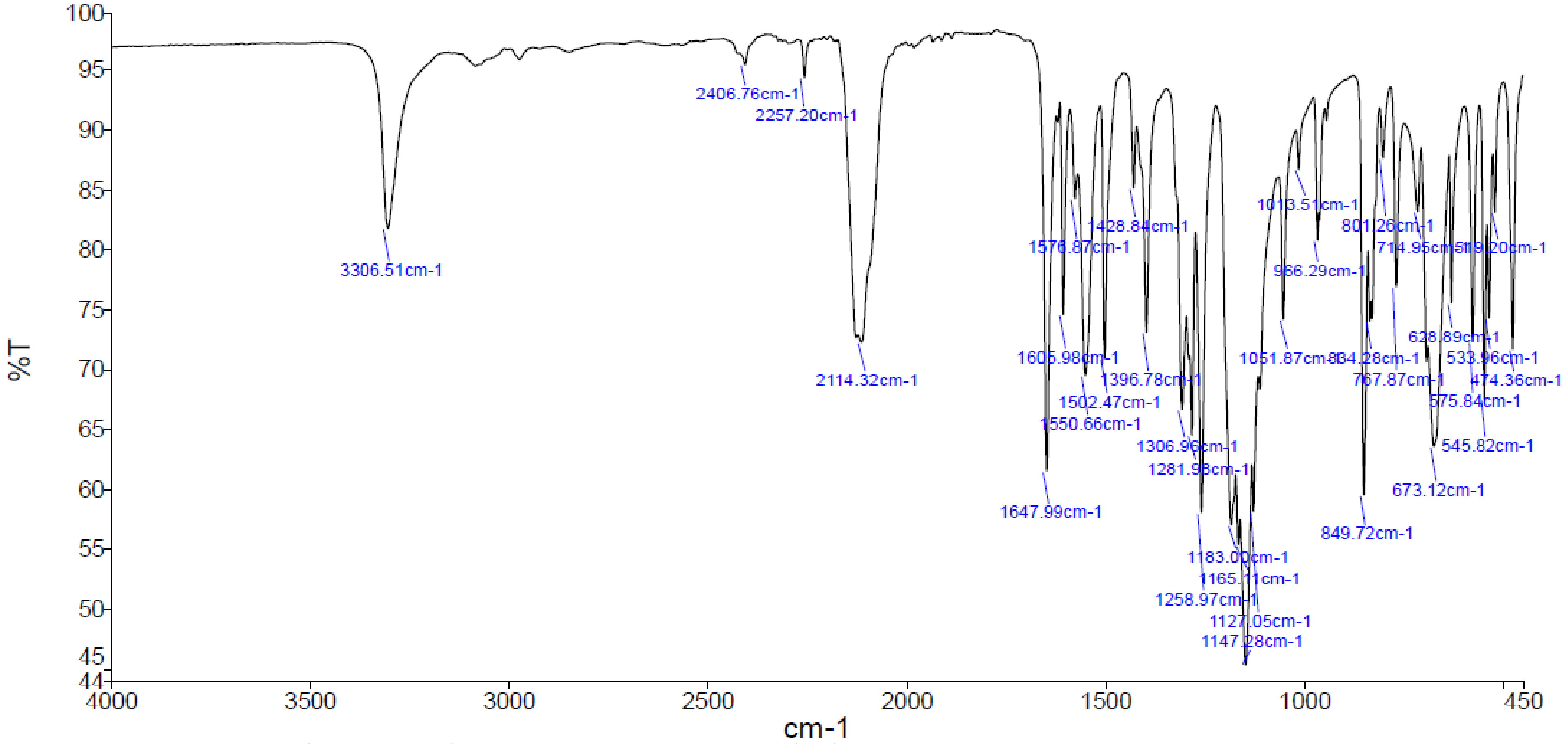

Figure S 15. FT-IR (ATR) spectrum of **3**.

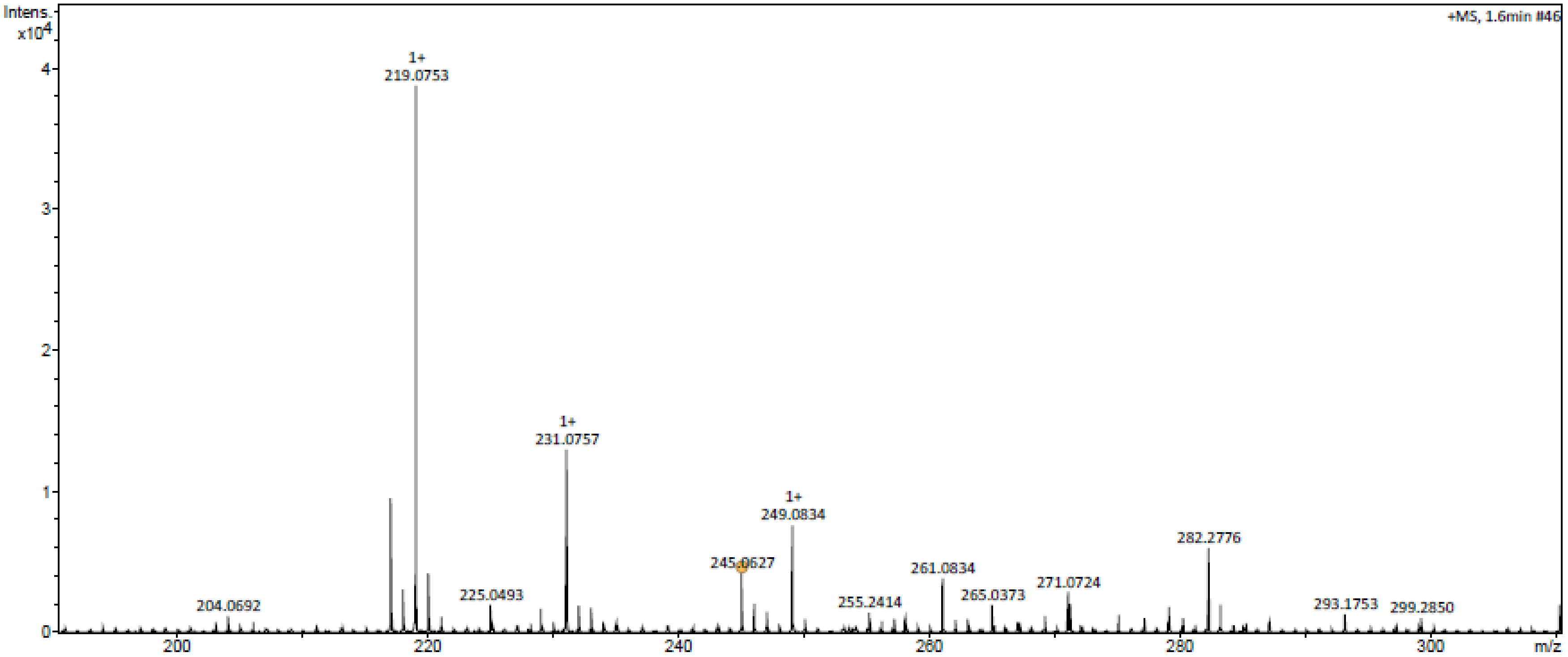

Meas. m/z	#	Ion Formula	m/z	err [ppm]	err [mDa]	mSigma	Mean err [ppm]
245.062708	1	C9H8F3N4O	245.064472	-7.2	-1.8	205.6	2.1

Figure S 16. Positive mode (APCI)HRMS of **3**.

Tetrabutylammonium 3-((Z)-3-mesityl-2-((E)-(4-((2,2,2-trifluoroethyl)carbamoyl)phenyl)triaz-2-en-1-ylidene)-2,3-dihydro-1H-imidazol-1-yl)propane-1-sulfonate 4

To a mixture of **2** (0.759 g, 2.46 mmol) and **3** (0.500 g, 2.05 mmol) in 20 mL of anhydrous DMSO was added potassium tert-butoxide (0.276 g, 2.05 mmol). The resultant solution was then placed under N_2 and stirred at room temperature overnight in darkness. Tetrabutylammonium bromide (0.658 g, 2.05 mmol) was then added to the resultant solution. The solution was stirred for 5 min, then DCM (200 mL) was added. The resultant solution was transferred to a separating funnel and was washed with water (2 × 200 mL). Upon the second wash with water an emulsion was formed, which was broken via washing with brine (200 mL). The organic layer was then dried over $MgSO_4$ and the filtrate concentrated *in vacuo* to yield **4** as a bright orange foam (1.05 g, 62%).

^{1}H-NMR$^{*see\ note}$ (400 MHz, Methanol-d$_4$): δ_H 7.62-7.56 (m, 2H), 7.31 (d, *J* = 2.50 Hz, 1H), 7.05 (s, 2H), 6.79 (d, *J* = 2.50 Hz, 1H), 6.65-6.59 (m, 2H), 4.30 (t, *J* = 7.00 Hz, 2H), 4.06 (q, J_{H-F} = 9.40 Hz, 2H), 3.27-3.19 (m, 8H), 2.89 (t, *J* = 7.50 Hz, 2H), 2.40 (s, 3H), 2.40-2.31 (m, 2H), 2.03 (s, 6H), 1.71-1.59 (m, 8H), 1.41 (qt, *J* = 7.63, 7.63 Hz, 8H), 1.02 (t, *J* = 7.63 Hz, 12H).

^{13}C-NMR$^{*see\ note}$ (101 MHz, Methanol-d$_4$): δ_c 170.0, 155.6, 152.0, 140.1, 136.7, 135.6, 130.6, 130.4, 129.0, [127.4, 124.7]^{*2}, 121.38, 119.1, 118.6, 59.5 (t, $J_{13C-14N}$ = 2.80 Hz), 49.0 (located using HMQC), [42.2, 41.8, 41.5, 41.1]^{*3}, 40.5, 26.5, 24.8, 21.2, 20.7 (t, $J_{13C-14N}$ = 1.50 Hz), 18.0, 14.0.

^{19}F-NMR (376 MHz, Methanol-d$_4$): δ_F -73.5 (t, J_{H-F} = 9.40 Hz).

FT-IR (ATR) (υmax/cm^{-1}): 3304 (N-H stretch), 2961 (C-H stretch), 2875 (C-H stretch), 1652 (C=O stretch), 1600 (C=C stretch, aromatic), 1536, 1143 (S=O stretch).

(ESI)HRMS: Positive mode found 242.2846, $C_{16}H_{36}N^+$ requires 242.2842; negative mode found 551.1676, $C_{24}H_{26}F_3N_6O_4S^-$ requires 551.1694.

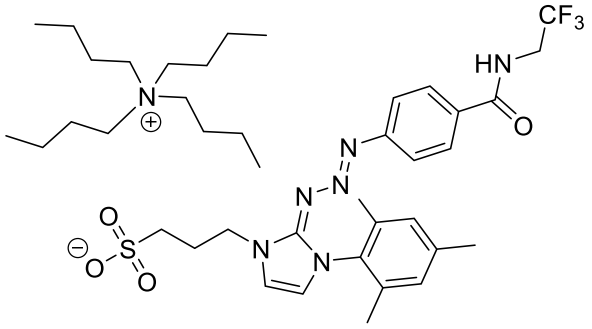

Figure S 17. The structure of **4**.

* **Note:** The highly symmetric environment of N atoms in quaternary ammonium compounds allows for the detection of the quadrupolar coupling of ^{14}N to other spin-active nuclei.[57] ^{13}C-^{14}N coupling is thus observed in some of the peaks corresponding the tetrabutylammonium cation, and ^{1}H-^{14}N coupling causes some tetrabutylammonium derived signals to appear as multiplets.

^{*2} These two peaks correspond to the most intense part of a 1:3:3:1 quartet formed due to the ^{13}C-^{19}F coupling of the C̲F$_3$ environment. Here J_{C-F} can be measured as 278.5 Hz.

^{*3} These four peaks correspond to a 1:3:3:1 quartet formed due to the ^{13}C-^{19}F coupling of the C̲H$_2$CF$_3$ environment. Here J_{C-F} can be measured as 34.8 Hz.

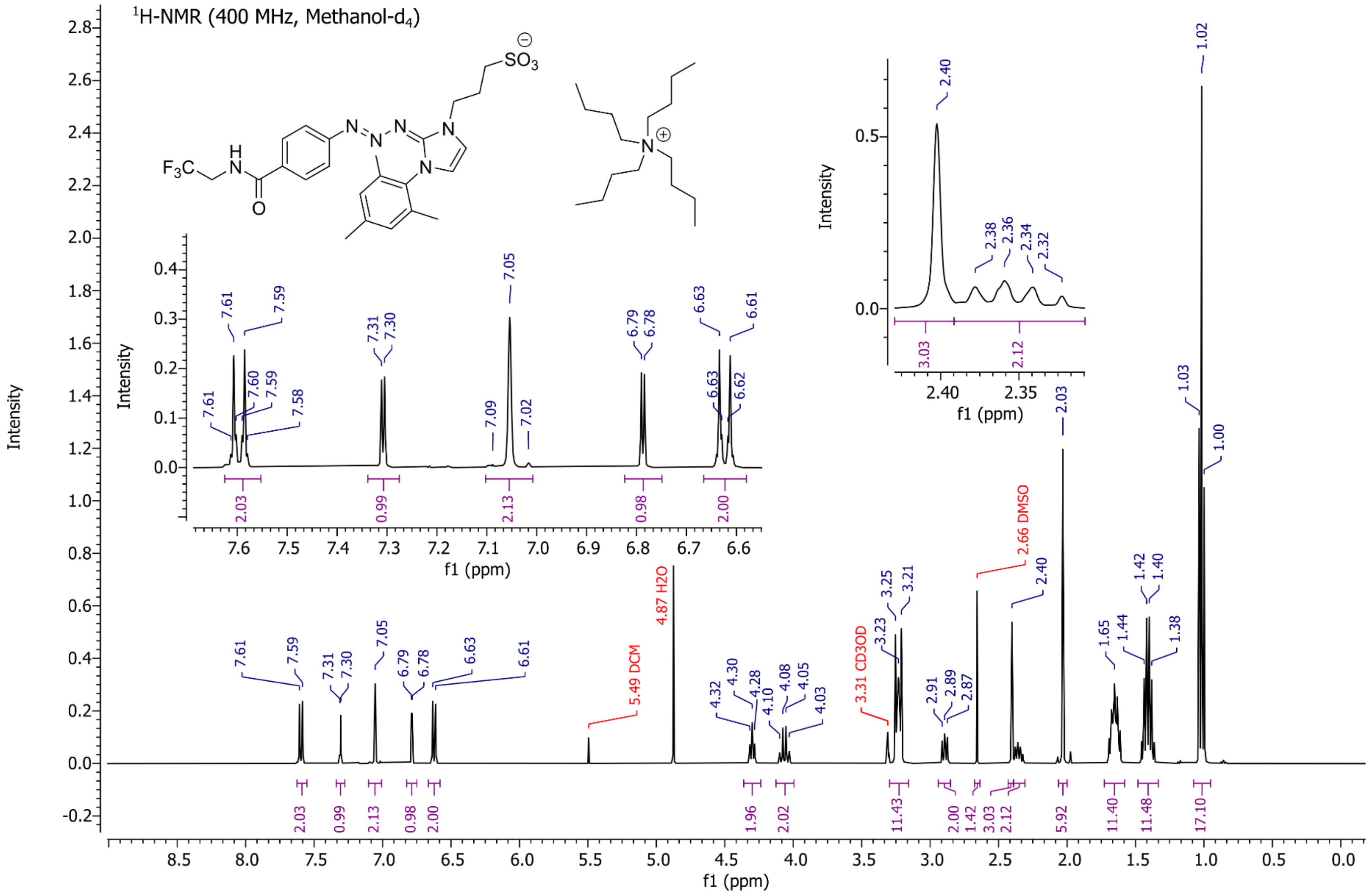

Figure S 18. ^{1}H-NMR spectrum of **4**. Note that we believe that the integrals corresponding to environments found on the tetrabutylammonium cation are measured to be larger than expected due to the effects of non-fully resolved ^{14}N-^{1}H quadrupolar couplings.

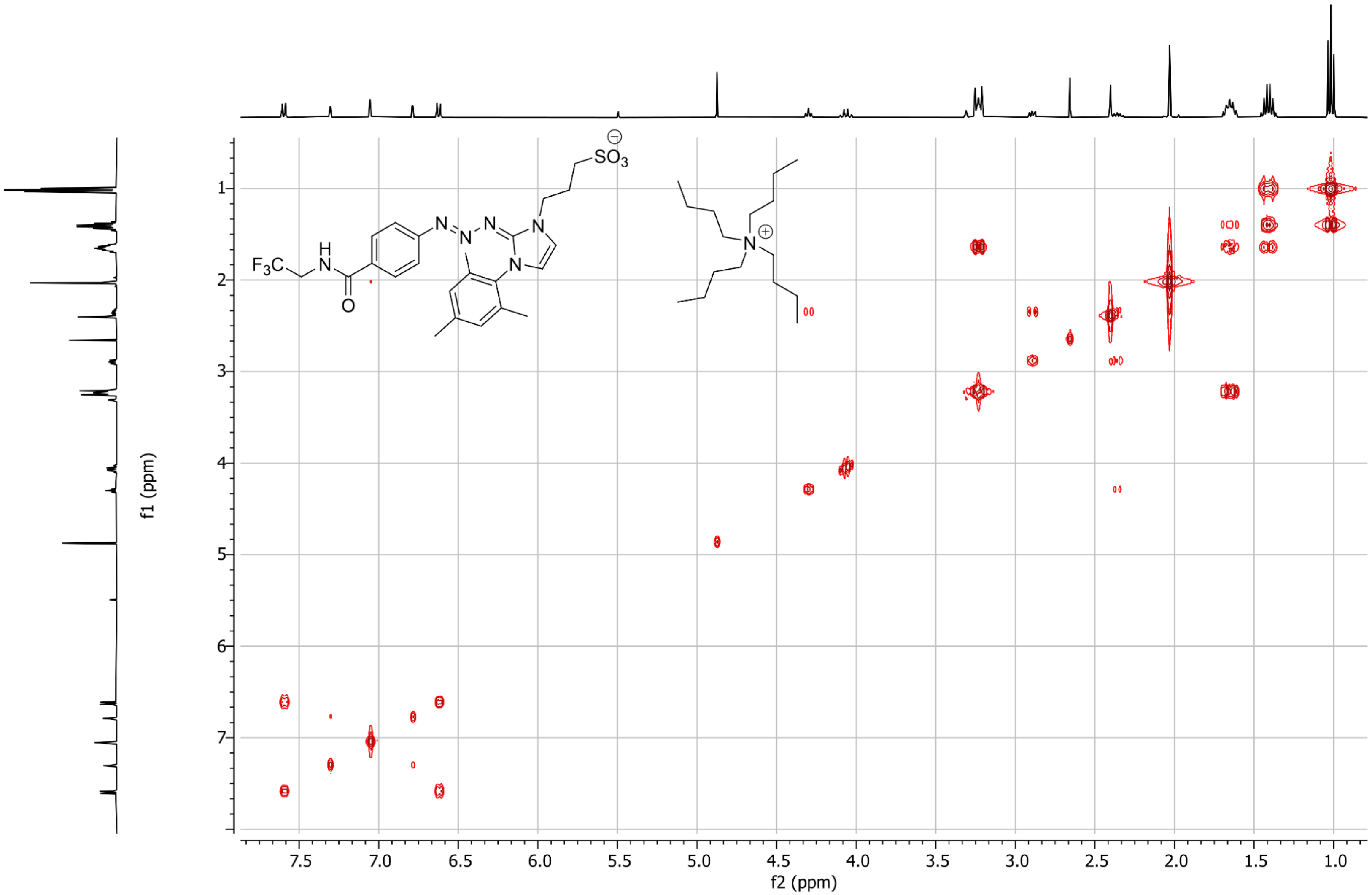

Figure S 19. COSY-NMR spectrum of **4**.

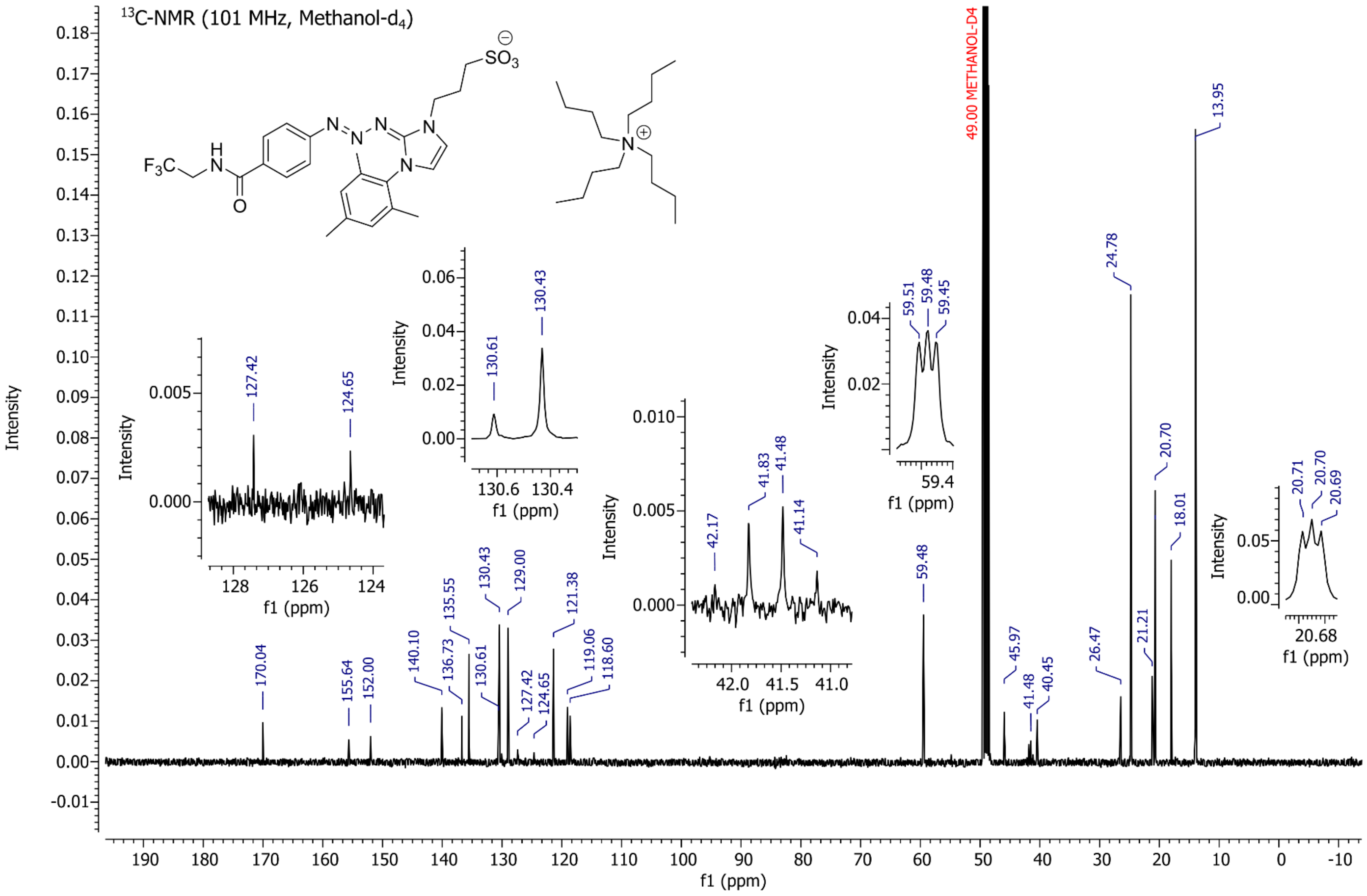

Figure S 20. ^{13}C-NMR spectrum of **4**.

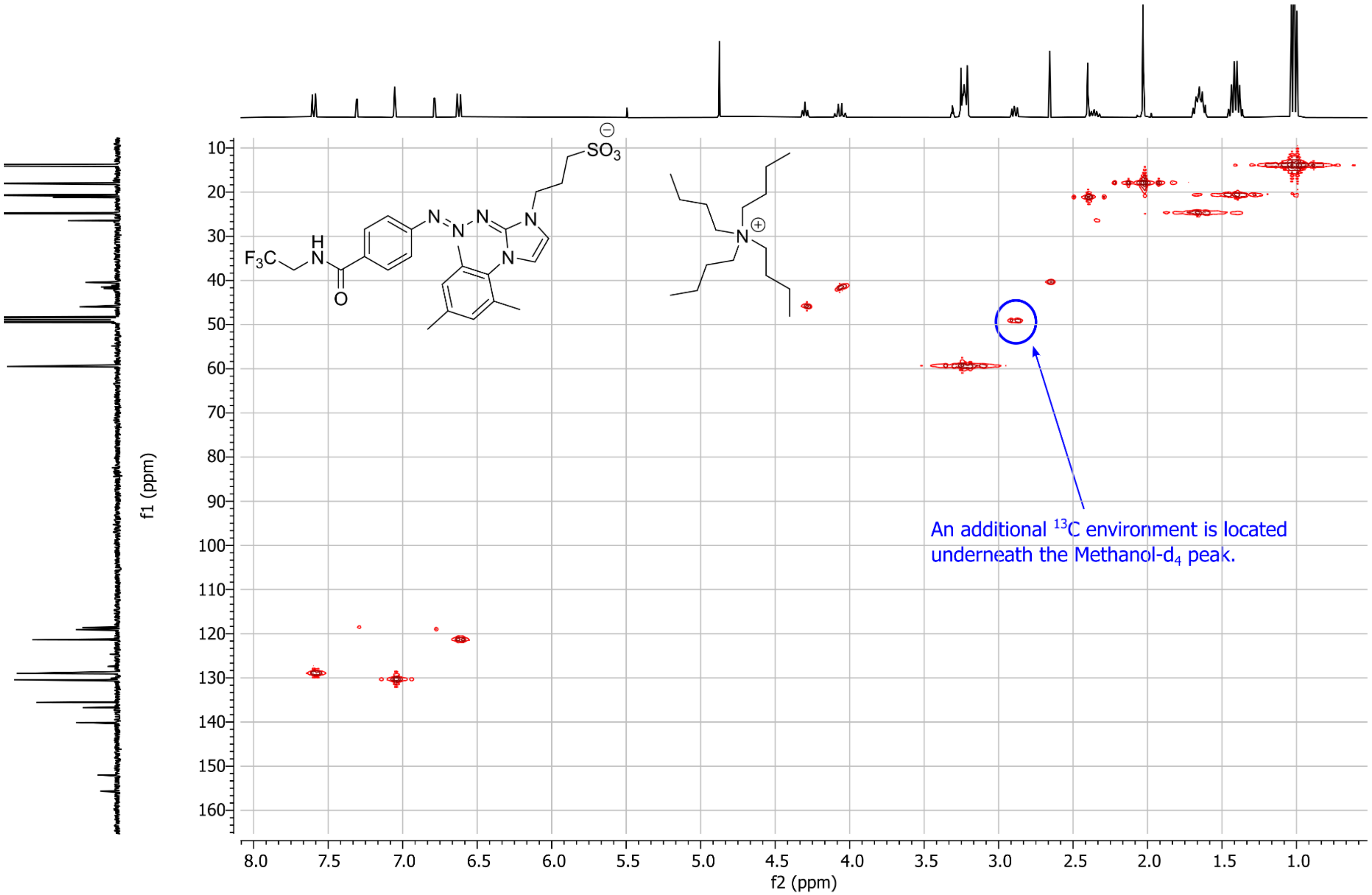

Figure S 21. ^{13}C-HMQC NMR spectrum of **4**.

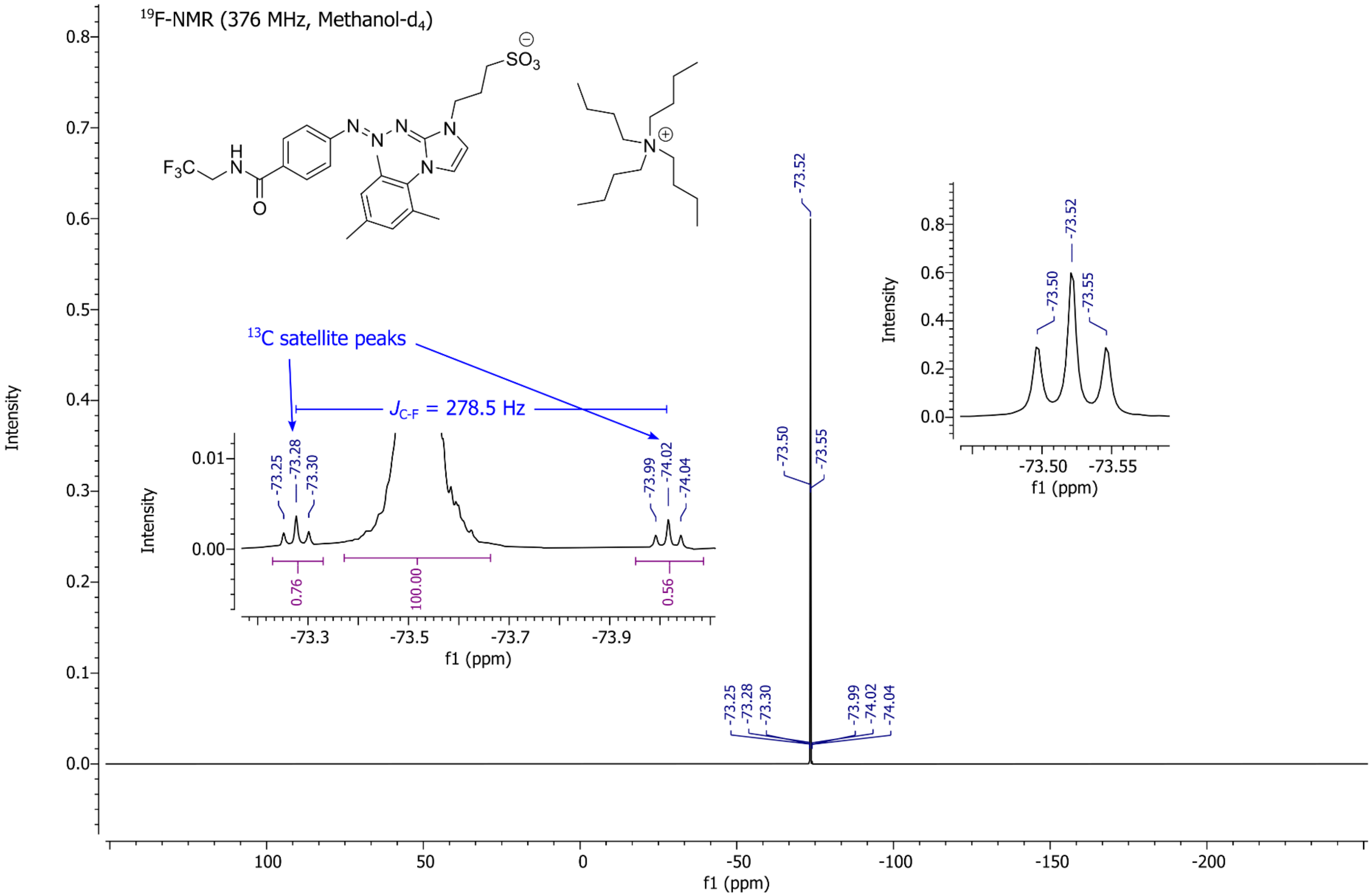

Figure S 22. ^{19}F-NMR spectrum of **4**. Note that the ^{13}C-^{19}F coupling constant of the <u>C</u>F$_3$ environment can be measured using the ^{13}C satellite peaks.

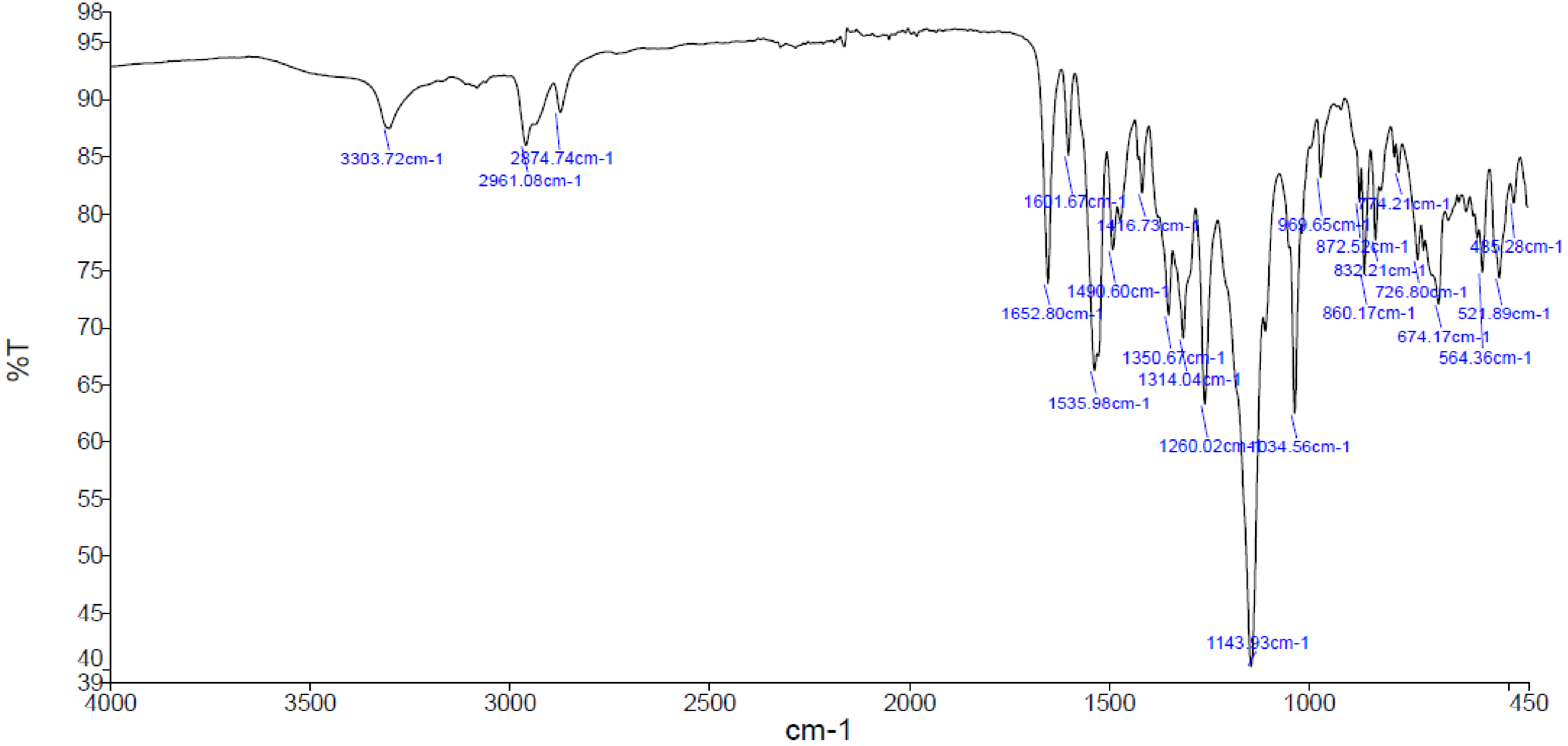

Figure S 23. FT-IR (ATR) spectrum of **4**.

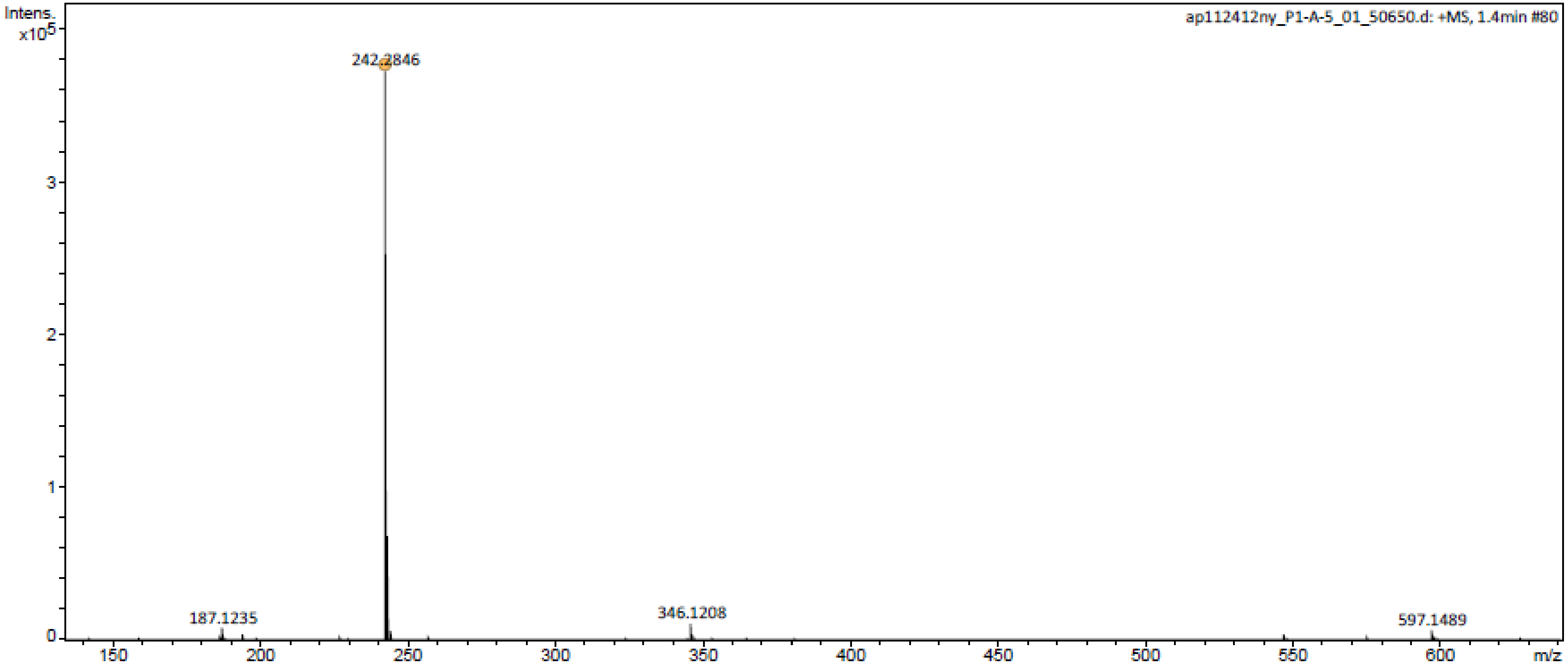

Meas. m/z	#	Ion Formula	m/z	err [ppm]	err [mDa]	mSigma	Mean err [ppm]
242.2846	1	C16H36N	242.2842	-1.7	-0.4	1.0	-2.0

Figure S 24. Positive mode (ESI)HRMS of **4**.

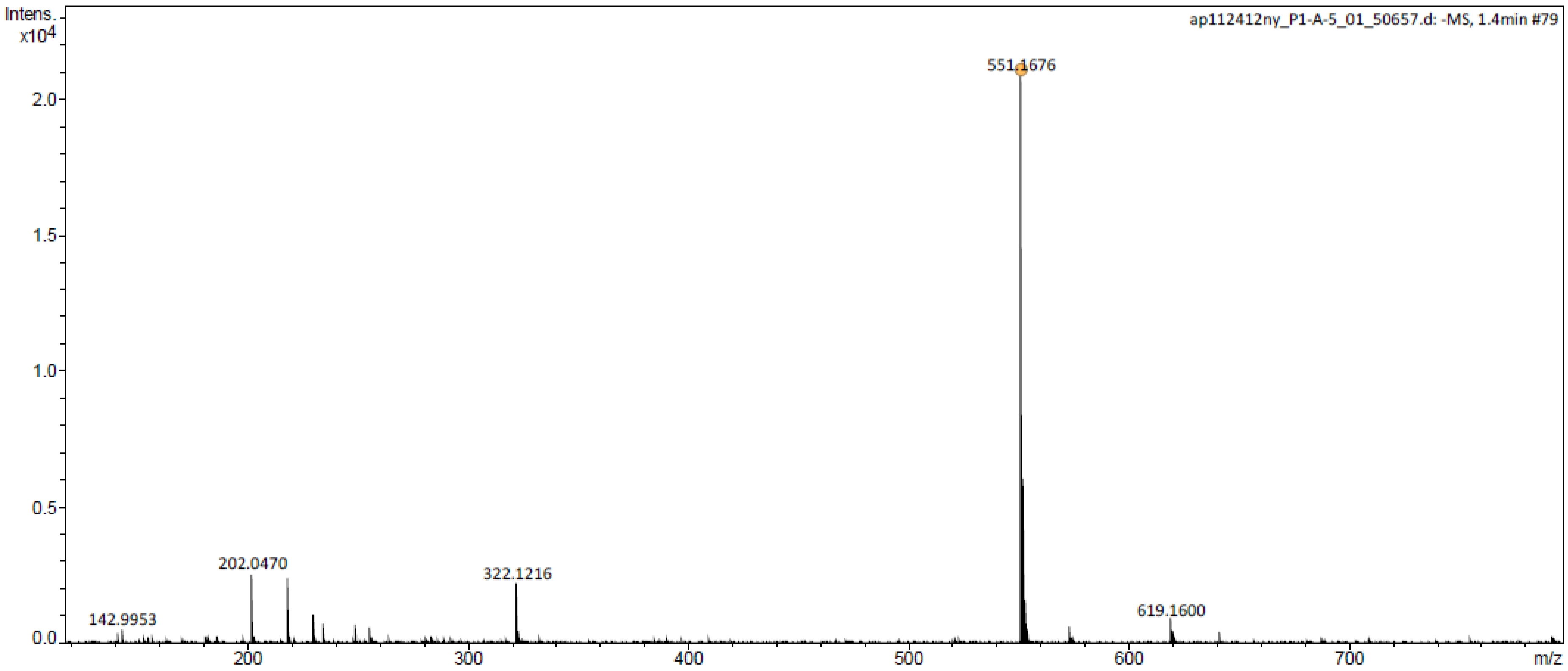

Meas. m/z	#	Ion Formula	m/z	err [ppm]	err [mDa]	mSigma	Mean err [ppm]
551.1676	1	C24H26F3N6O4S	551.1694	3.2	1.7	11.0	1.3

Figure S 25. Negative mode (ESI)HRMS of **4**.

4-amino-N-(2,2,2-trifluoroethyl)benzamide S1

To a solution of **3** (0.167 g, 0.68 mmol) in methanol (6 mL) was added a spatula-end of 10% Pd/C. The resultant mixture was then placed under a nitrogen atmosphere. The nitrogen atmosphere was then replaced with a hydrogen atmosphere (balloon), and the reaction mixture was stirred overnight at rt. The Pd/C was then removed via filtration and the eluate concentrated *in vacuo* to yield a sample of 4-amino-N-(2,2,2-trifluoroethyl)benzamide **S1** (0.116 g, 78%) which was used without further purification.

^{1}H-NMR (400 MHz, Methanol-d$_4$): δ_H 7.72-7.52 (m, 2H), 6.74-6.58 (m, 2H), 6.58 (br t, *J* = 6.45 Hz, 1H), 4.01 (q, *J* = 9.10, 2H).

^{13}C-NMR (101 MHz, Methanol-d$_4$): δ_c 170.5, 153.7, 130.3, [127.5, 124.7][*1], 121.9, 114.6, [42.1, 41.7, 41.4, 41.0][*2].

^{19}F-NMR (376 MHz, Methanol-d$_4$): δ_F -72.2 (t, $J_{H\text{-}F}$ = 9.10 Hz).

FT-IR (ATR) (υmax/cm^{-1}): 3343 (N-H stretch), 1626 (C=O stretch), 1604 (C=C stretch, aromatic).

(ESI)HRMS: Positive mode found 241.0559, $C_9H_9F_3N_2NaO^+$ requires 241.0559.

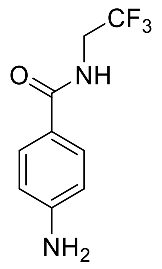

Figure S 26. The structure of **S1**.

[*1] These two peaks correspond to the most intense signals from a 1:3:3:1 quartet formed due to the ^{13}C-^{19}F coupling of the C̲F$_3$ environment. Here $J_{C\text{-}F}$ can be measured as 278.5 Hz.

[*2] These four peaks correspond to a 1:3:3:1 quartet formed due to the ^{13}C-^{19}F coupling of the C̲H$_2$CF$_3$ environment. Here $J_{C\text{-}F}$ can be measured as 34.5 Hz

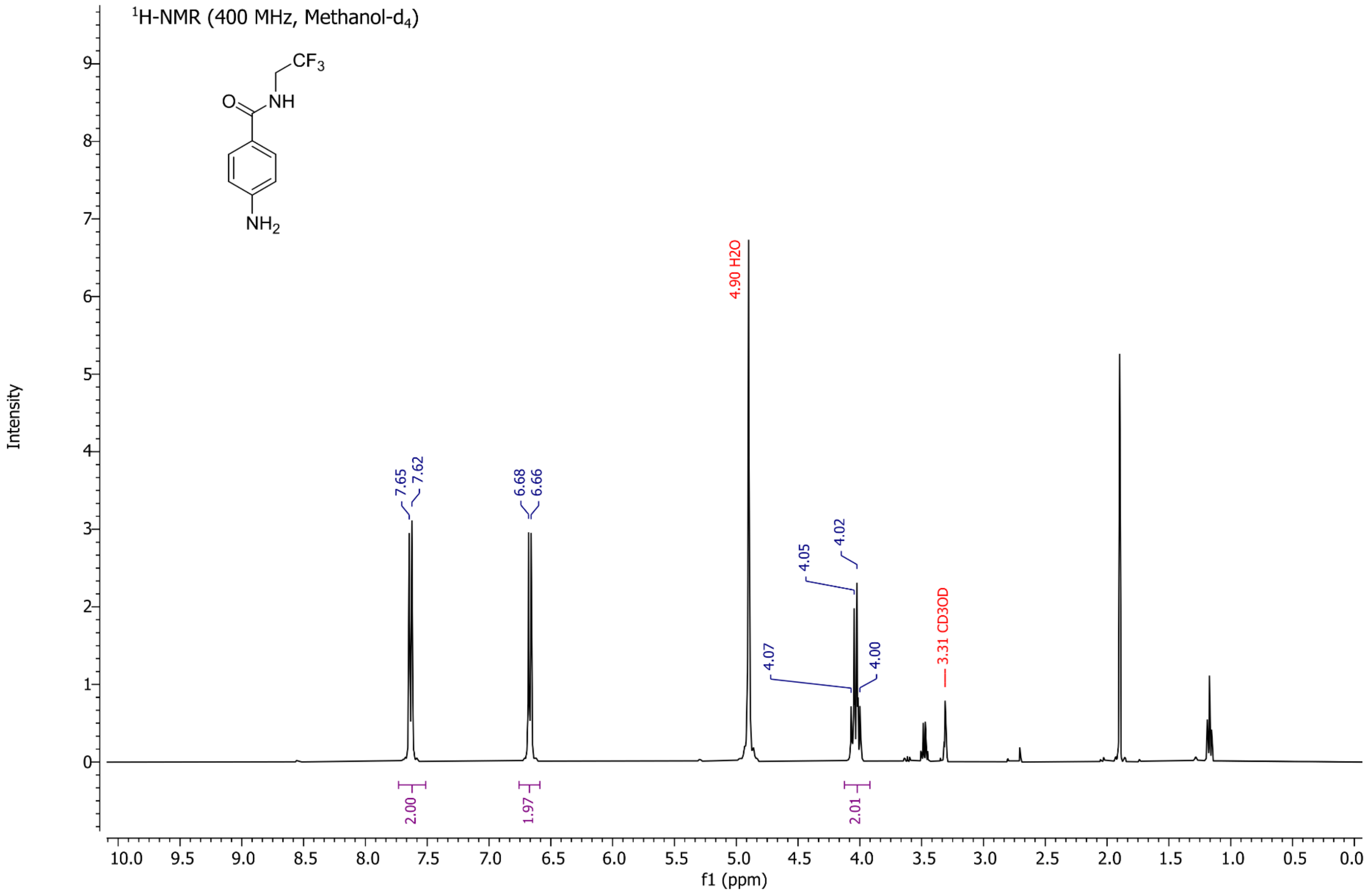

Figure S 27. ^{1}H-NMR spectrum of **S1**.

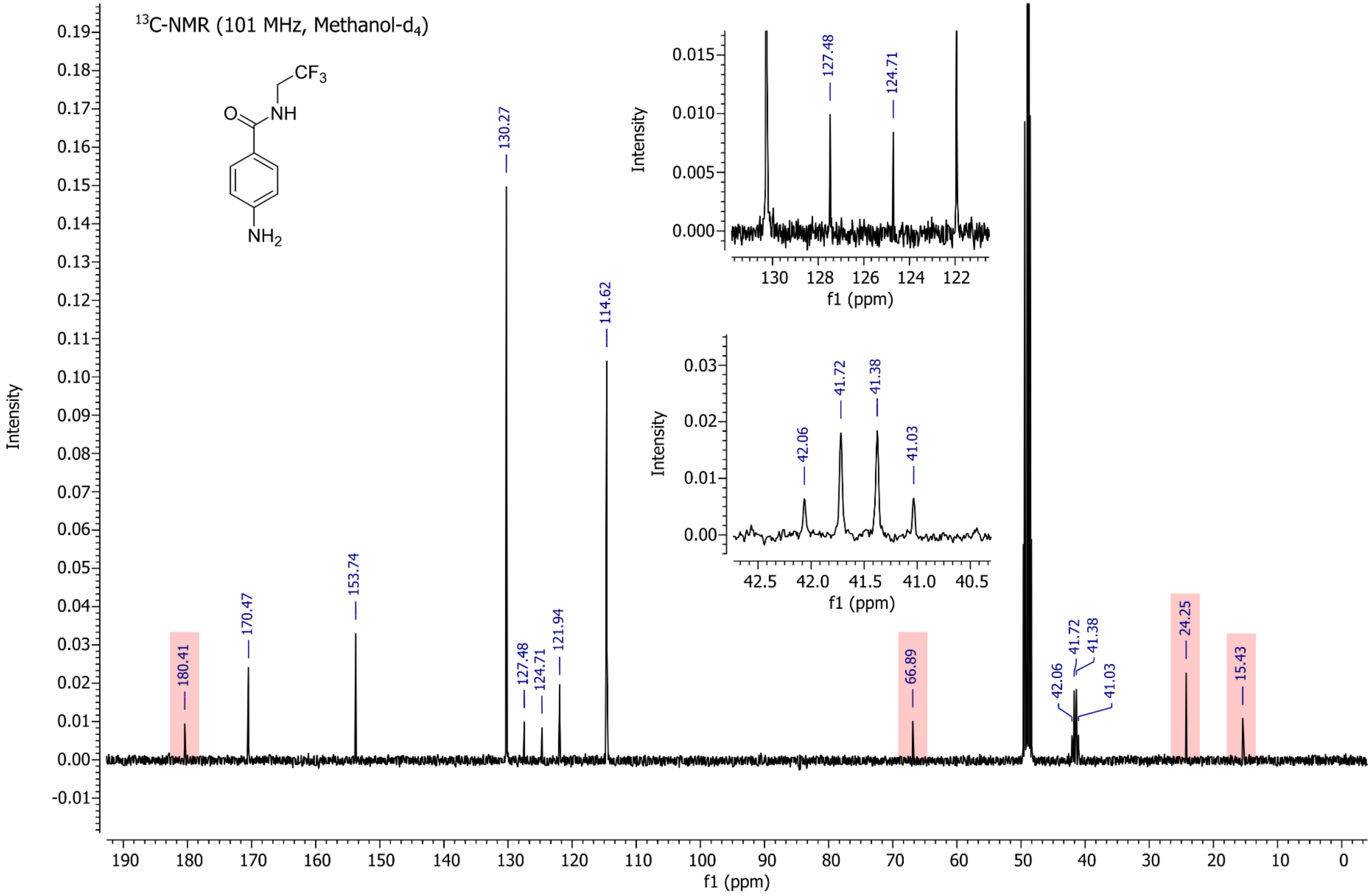

Figure S 28. ^{13}C-NMR spectrum of **S1**.

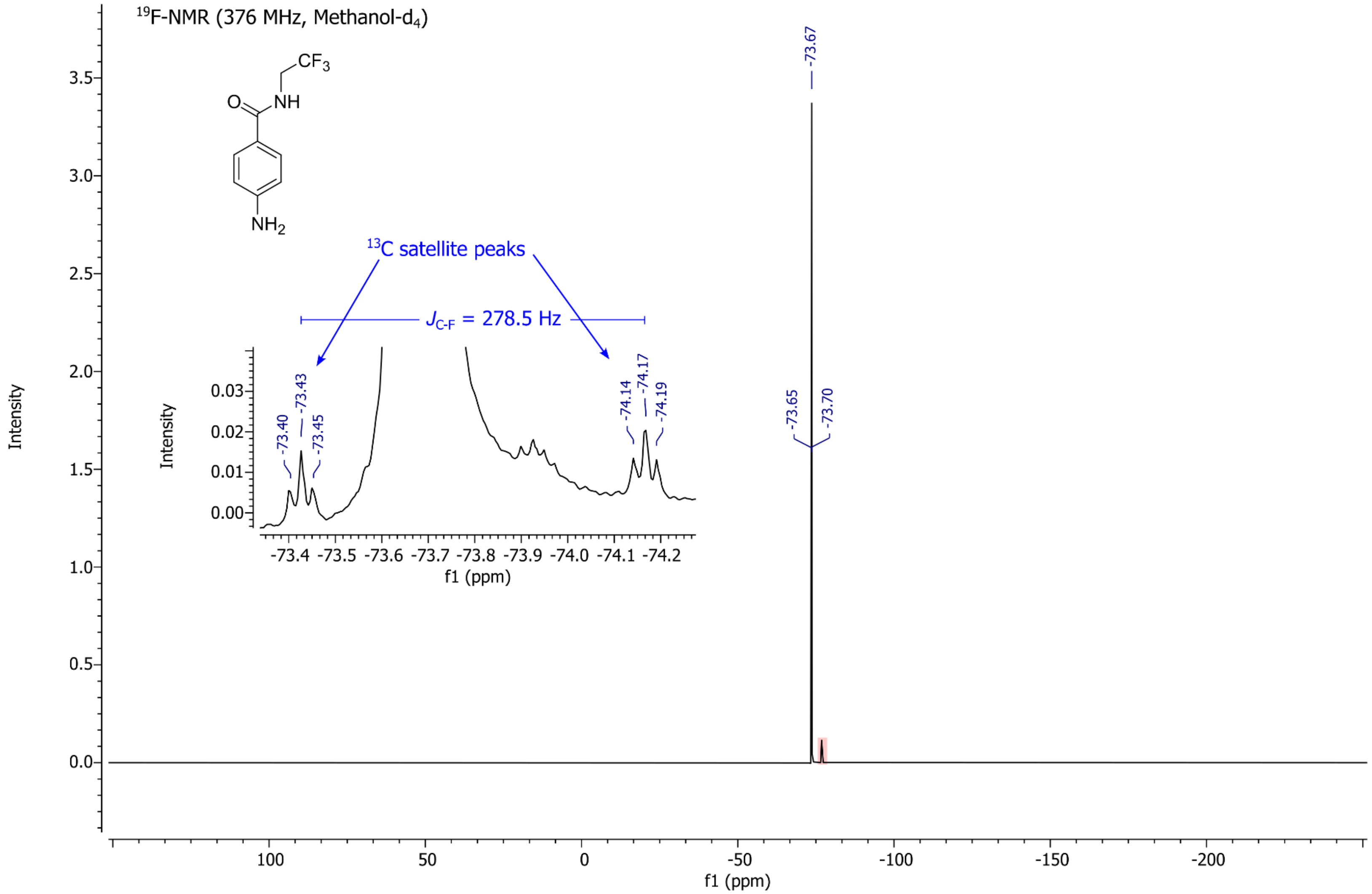

Figure S 29. ^{19}F-NMR spectrum of **S1**.

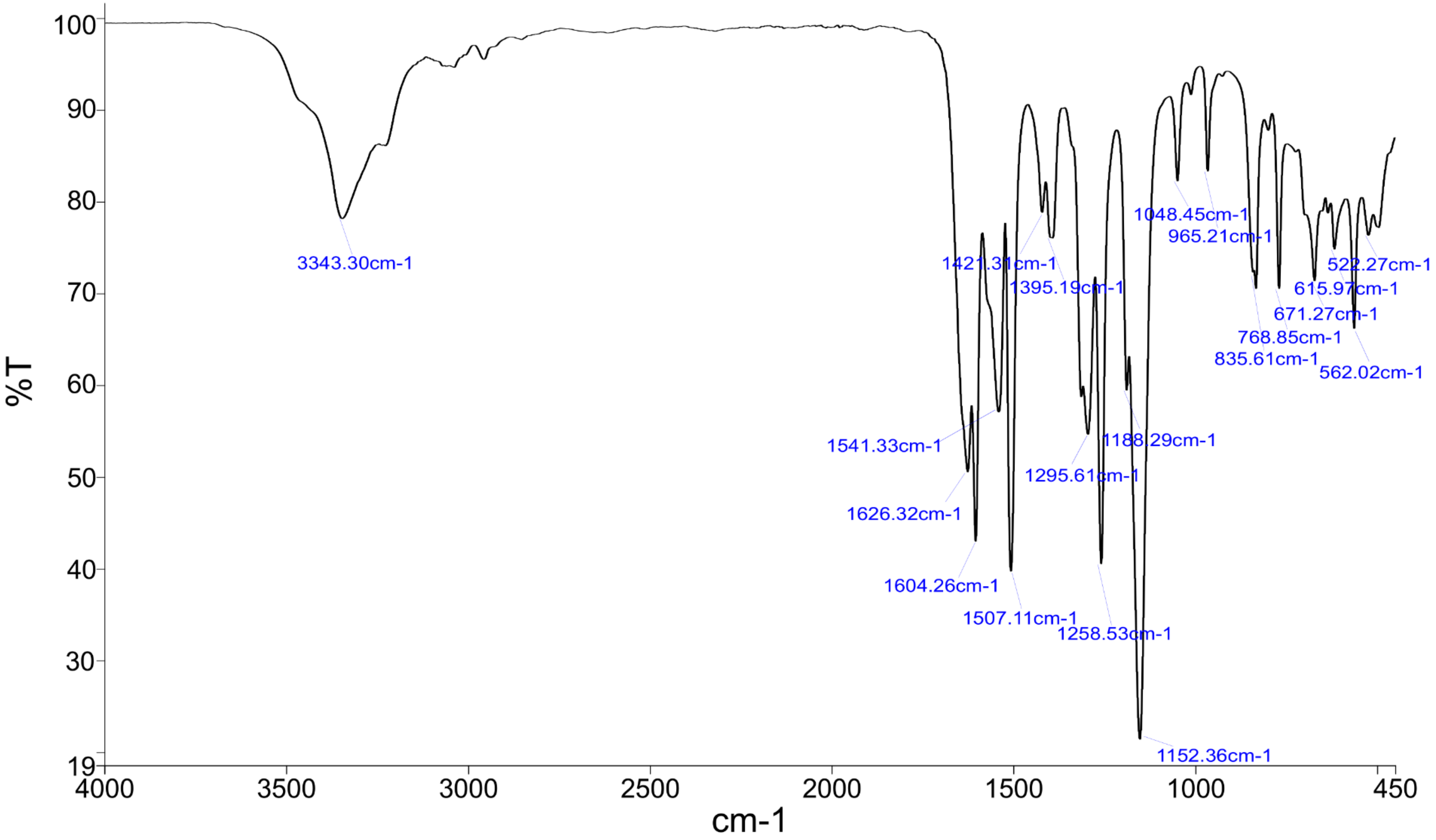

Figure S 30. FT-IR (ATR) spectrum of **S1**.

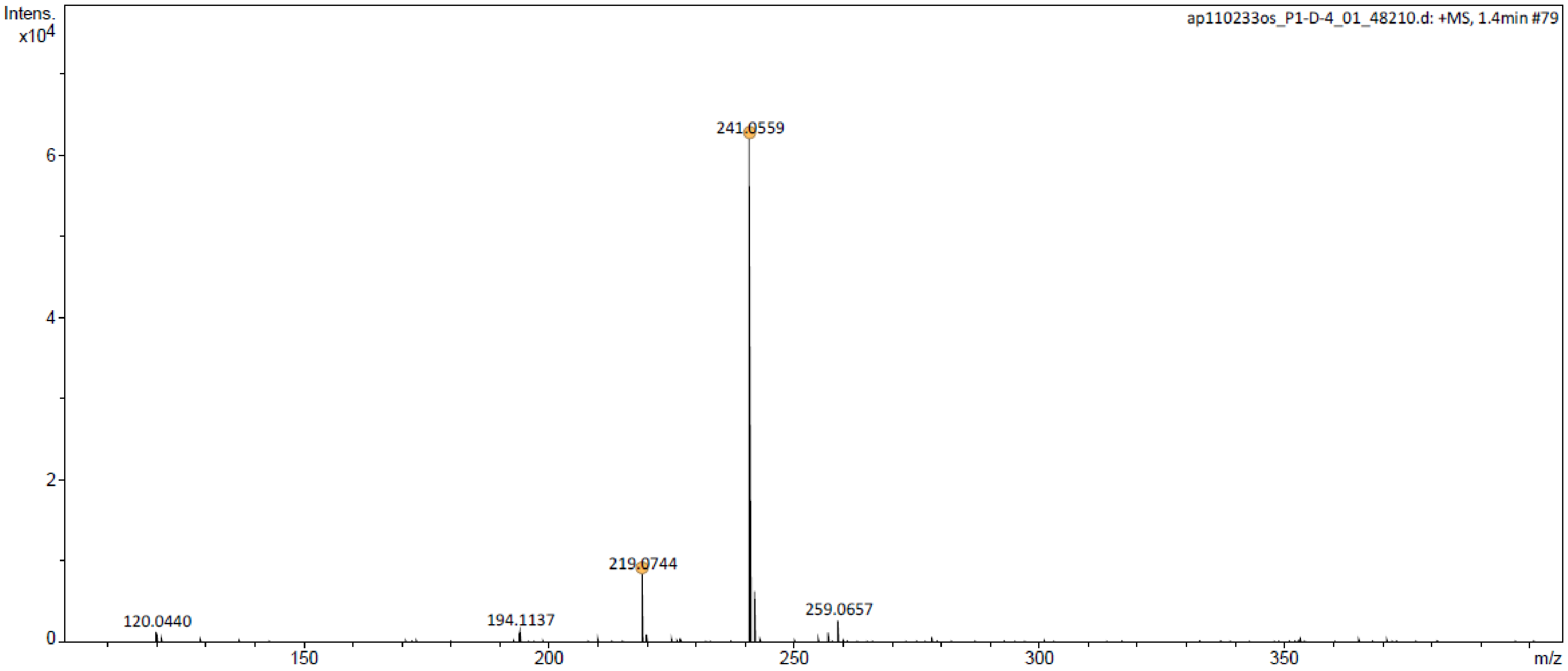

Meas. m/z	#	Ion Formula	m/z	err [ppm]	err [mDa]	mSigma	Mean err [ppm]
219.0744	1	C9H10F3N2O	219.0740	-1.7	-0.4	11.7	-0.9
241.0559	1	C9H9F3N2NaO	241.0559	0.2	0.0	2.3	-4.0

Figure S 31. Positive mode (ESI)HRMS of **S1**.

4-((2,2,2-trifluoroethyl)carbamoyl)benzenediazonium tetrafluoroborate 5

$NOBF_4$ (57 mg, 0.49 mmol) was dissolved in anhydrous acetonitrile (1.2 mL) under a nitrogen atmosphere at -30°C. A solution of **S1** (88 mg, 0.40 mmol) in anhydrous acetonitrile (0.4 mL) was then added dropwise, and the resultant solution stirred for 30 minutes at -30°C. After this time the cold bath was removed and the reaction solution stirred for a further 30 minutes as it slowly warmed. The reaction solution was then diluted with 3.2 mL of diethyl ether and further stirred until a copious quantity of precipitate had formed. This precipitate was collected by filtration and rinsed with additional diethyl ether, yielding 4-((2,2,2-trifluoroethyl)carbamoyl)benzenediazonium tetrafluoroborate **5** as a white powder (96 mg, 75%).

^{1}H-NMR (400 MHz, DMSO-d$_6$): 9.69 (t, J = 6.30 Hz, 1H), 8.82-7.50 (m, 2H), 8.35-8.27 (m, 2H), 4.16 (qd, J = 9.50, 6.30 Hz, 2H).

^{13}C-NMR (101 MHz, DMSO-d$_6$): δ_C 164.9, 143.2, 133.1, 129.9, [128.8 + 126.0 + 123.3 + 120.5]*1, 118.9, [41.0 + 40.6 + 40.3]*2.

^{19}F-NMR (376 MHz, DMSO-d$_6$): δ_F -70.2 (t, J_{H-F} = 9.50 Hz, C$\underline{F}_3$), -148.1 (s, ^{10}B$\underline{F}_4$), -148.1 (s, ^{11}B$\underline{F}_4$).

FT-IR (ATR) (υmax/cm^{-1}): 3326 (N-H stretch), 2314 (N≡N stetch, diazonium)[58], 1656 (C=O stretch), 1555 (C=C stretch, aromatic), 1031.

(ESI)HRMS: Found 230.0534, $C_9H_7F_3N_3O^+$ requires 230.0534.

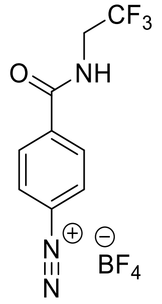

Figure S 32. The structure of **5**.

*1 These four peaks correspond to the most intense signals from a 1:3:3:1 quartet formed due to the ^{13}C-^{19}F coupling of the $\underline{C}F_3$ environment. Here J_{C-F} can be measured as 278.5 Hz.

*2 These three peaks correspond to a 1:3:3:1 quartet formed due to the ^{13}C-^{19}F coupling of the $\underline{C}H_2CF_3$ environment. Here J_{C-F} can be measured as 33.7 Hz

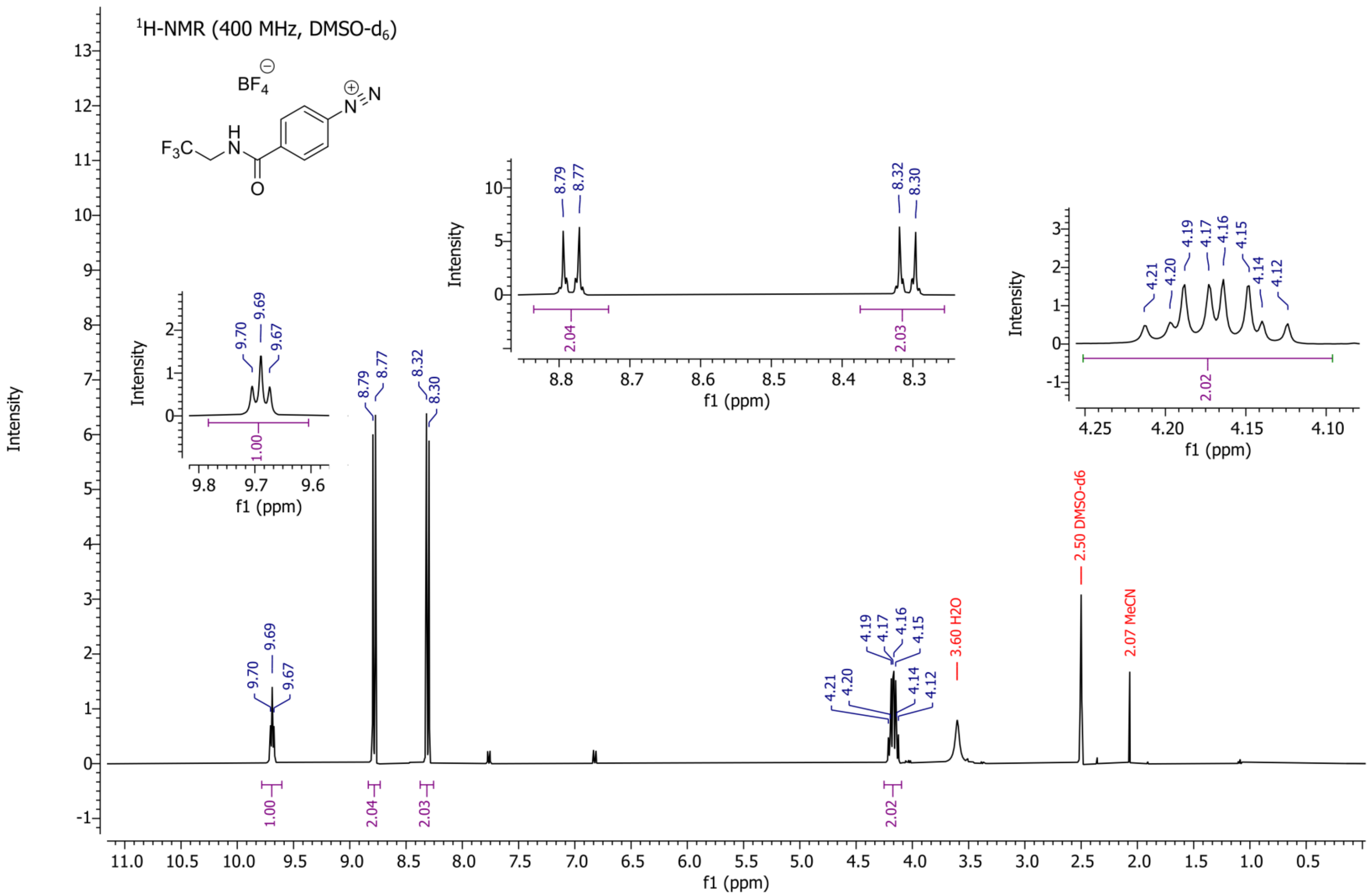

Figure S 33. ^{1}H-NMR spectrum of **5**.

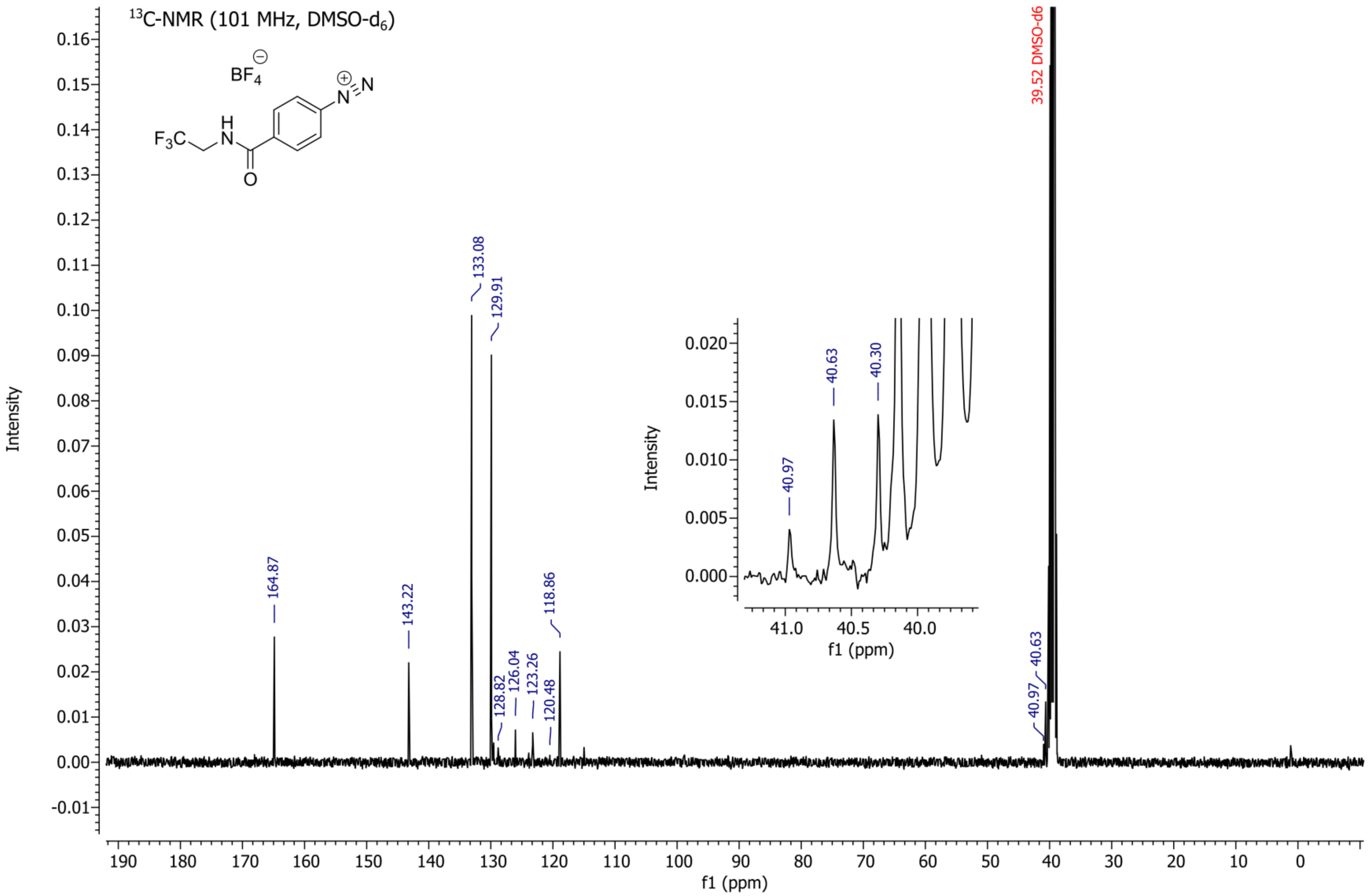

Figure S 34. ^{13}C-NMR spectrum of **5**.

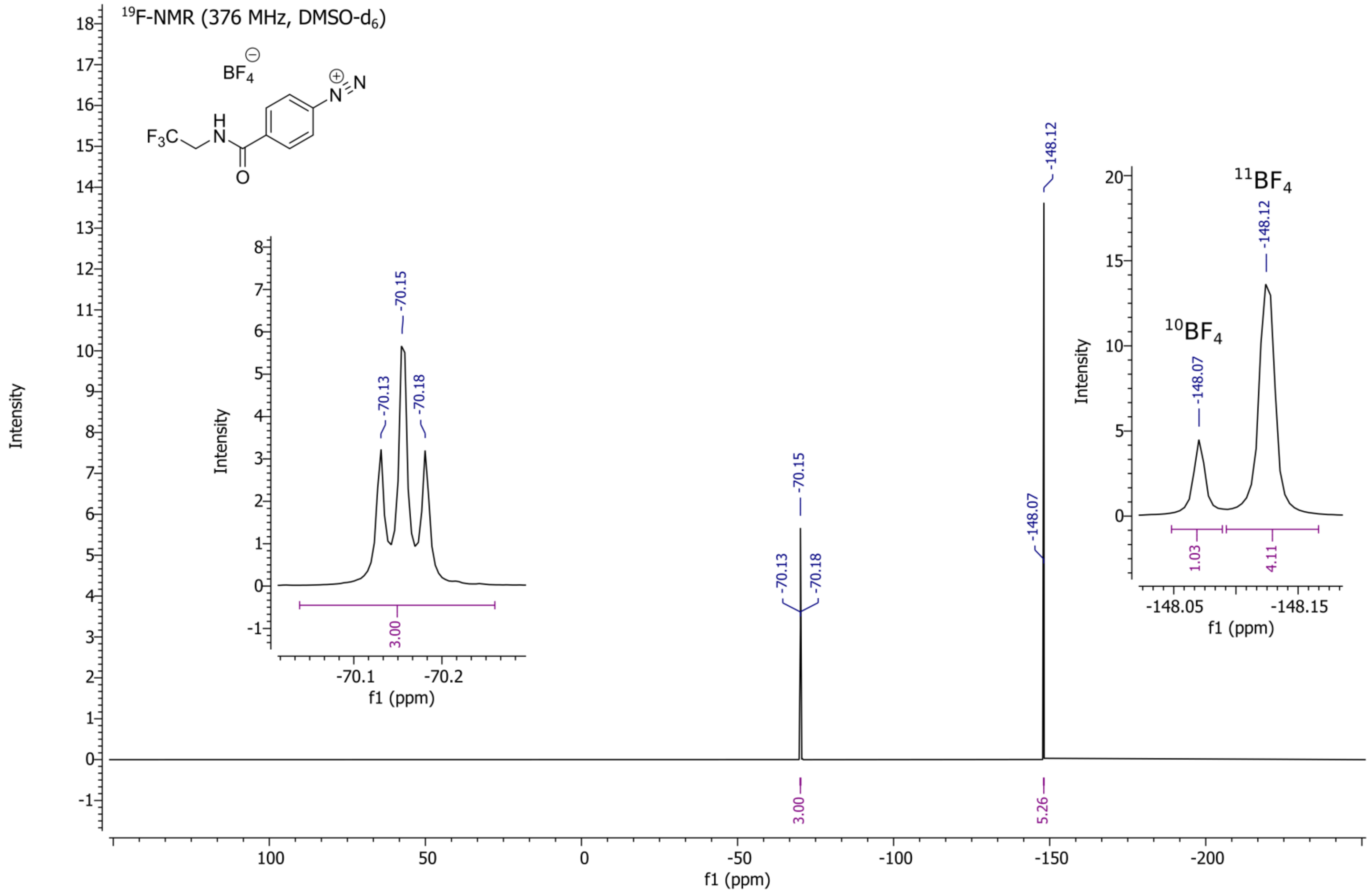

Figure S 35. ^{19}F-NMR spectrum of **5**.

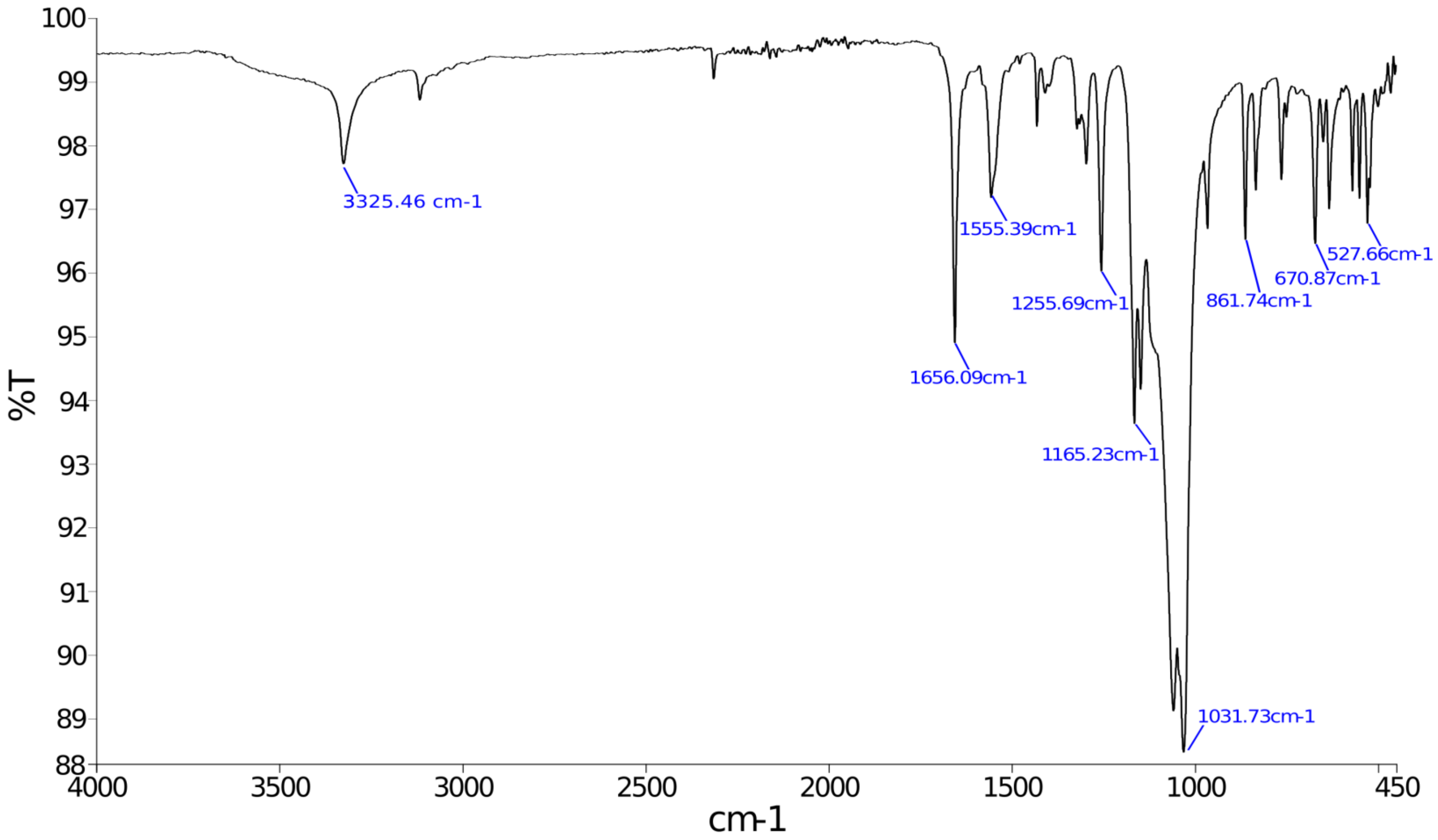

Figure S 36. FT-IR (ATR) spectrum of **5**.

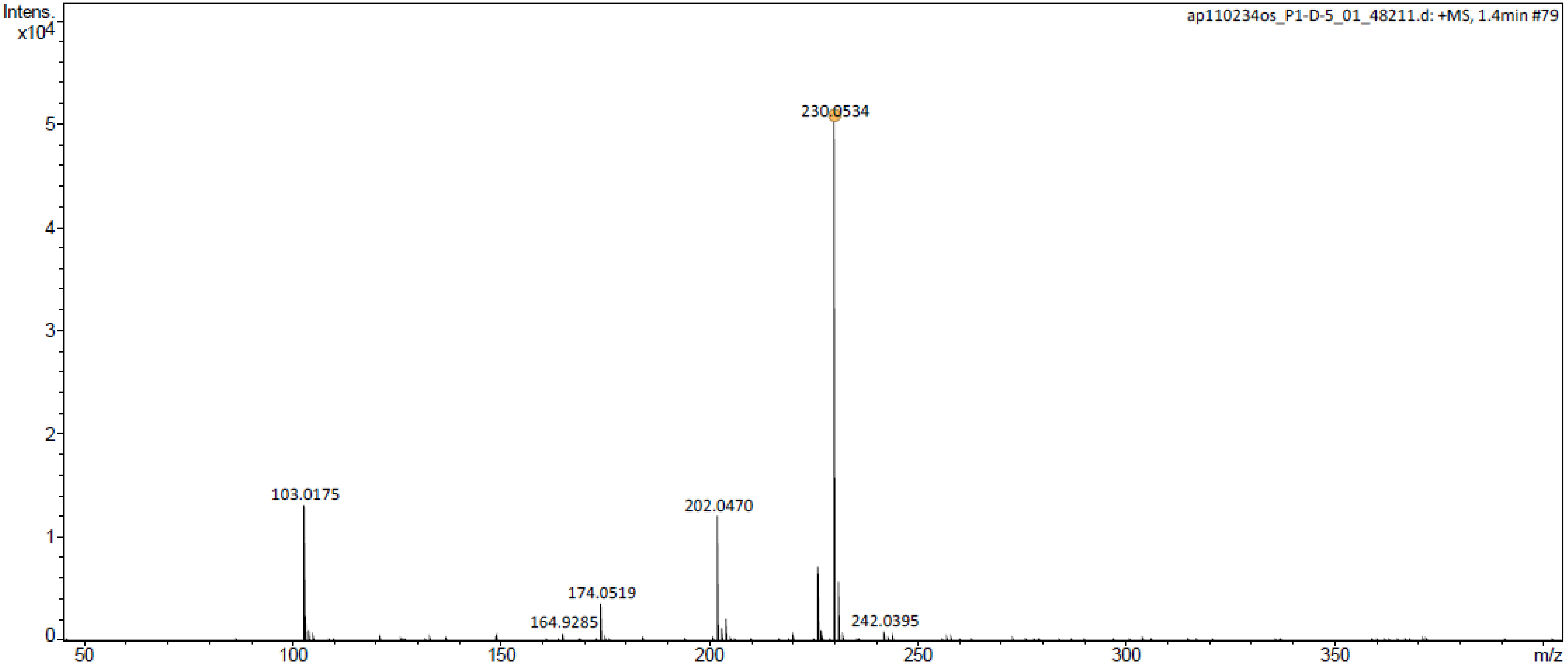

Meas. m/z	#	Ion Formula	m/z	err [ppm]	err [mDa]	mSigma	Mean err [ppm]
230.0534	1	C9H7F3N3O	230.0536	0.5	0.1	2.5	0.9

Figure S 37. Positive mode (ESI)HRMS of **5**.

Methyl 4-azidobenzoate 6

Methyl 4-azidobenzoate **6** was synthesised according to our previously published method.[40]

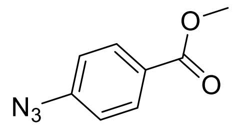

Figure S 38. The structure of **6**.

Tetrabutylammonium 3-((Z)-3-mesityl-2-((E)-(4-(methoxycarbonyl)phenyl)triaz-2-en-1-ylidene)-2,3-dihydro-1H-imidazol-1-yl)propane-1-sulfonate 7

To a mixture of **2** (1.14 g, 3.6 mmol) and **6** (0.65 g, 3.6 mmol) was added 20 mL of anhydrous DMSO, followed by potassium tert-butoxide (0.504 g, 4.4 mmol). The resultant solution was then placed under N_2 and stirred at room temperature overnight in darkness. To the resultant solution, tetrabutylammonium bromide (1.18 g, 3.6 mmol) was added. The solution was stirred for 5 min, then DCM (200 mL) was added. The resultant solution was transferred to a separating funnel and was washed with water (3 × 100 mL). The organic layer was then dried over $MgSO_4$ and the filtrate concentrated *in vacuo* to yield **7** as an orange foam (2.23 g, 85%).

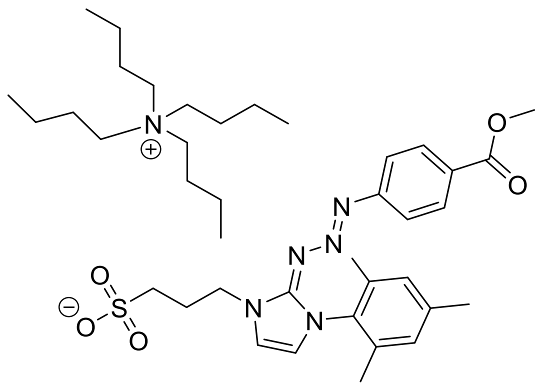

Figure S 39. The structure of **7**.

^{1}H-NMR (400 MHz, $CDCl_3$): δ_H 7.72-7.66 (m, 2H), 7.19 (d, *J* = 3.05 Hz, 1H), 6.96 (s, 2H), 6.63-6.57 (m, 2H), 6.32 (d, *J* = 3.05 Hz, 1H), 4.38 (t, *J* = 6.87 Hz, 2H), 3.84 (s, 3H), 3.33-3.23 (m, 8H), 2.86 (t, *J* = 6.87 Hz, 2H), 2.44-2.34 (m, 5H), 1.98 (s, 6H), 1.70-1.58 (m, 8H), 1.43 (qt, *J* = 7.63, 7.63 Hz, 8H), 0.99 (t, *J* = 7.63 Hz, 12H).

^{13}C-NMR (100 MHz, $CDCl_3$): δ_c 167.1, 153.2, 149.7, 138.8, 135.2, 134.2, 130.0, 129.4, 126.2, 119.9, 118.5, 117.2, 58.8, 51.9, 47.5, 45.0, 41.0, 25.5, 24.0, 21.1, 19.7, 17.9, 13.7.

FT-IR (ATR) (υmax/cm^{-1}): 2961 (C-H stretch), 1711 (C=O stretch, ester), 1600 (C=C stretch, aromatic), 1536, 1488, 1347, 1182, 1146.

(ESI)HRMS: Positive mode found 242.2835, $C_{16}H_{36}N^+$ requires 242.2842; negative mode found 484.1652, $C_{23}H_{26}N_5O_5S^-$ requires 484.1660.

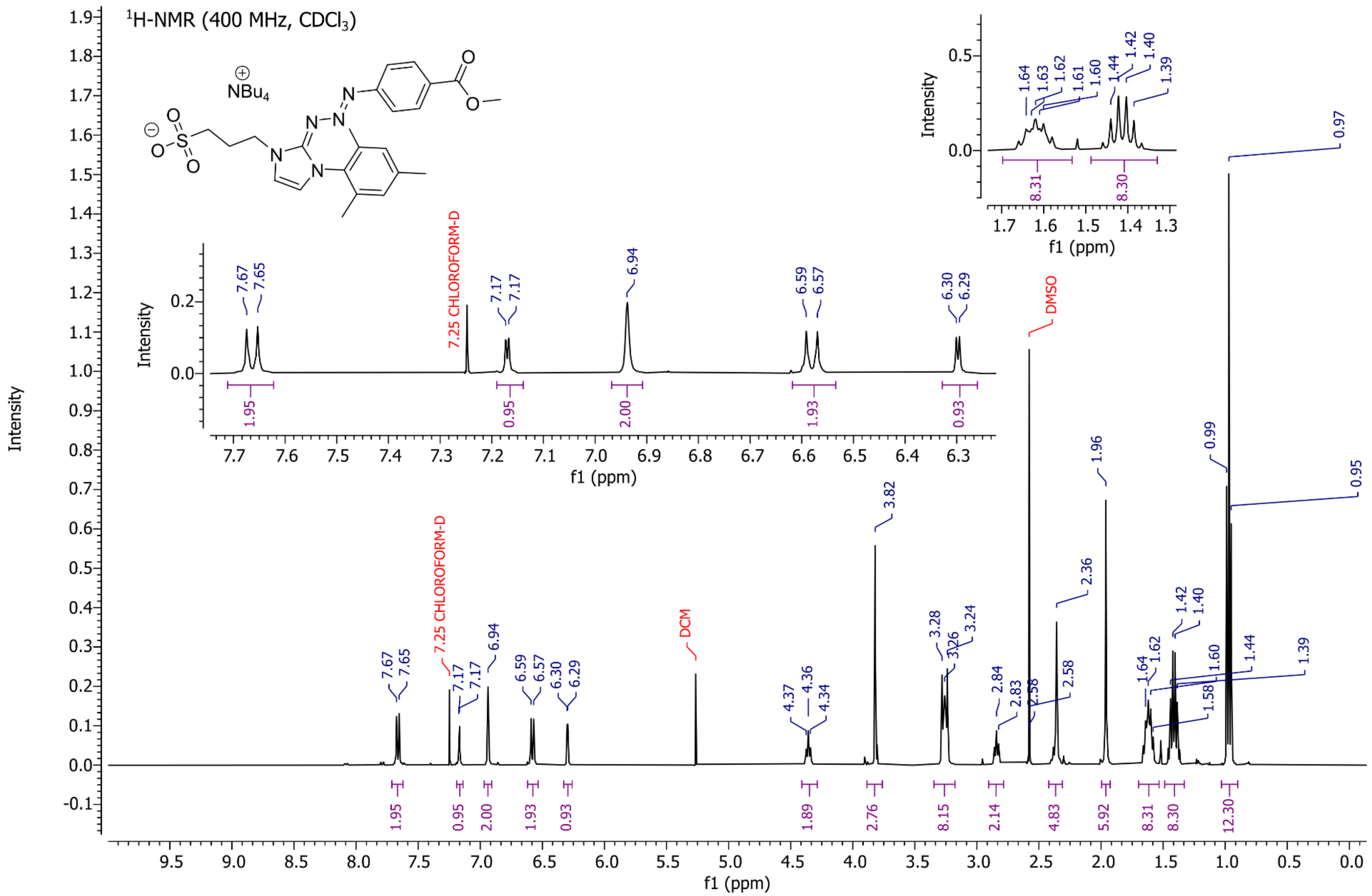

Figure S 40. ^{1}H-NMR spectrum of **7**.

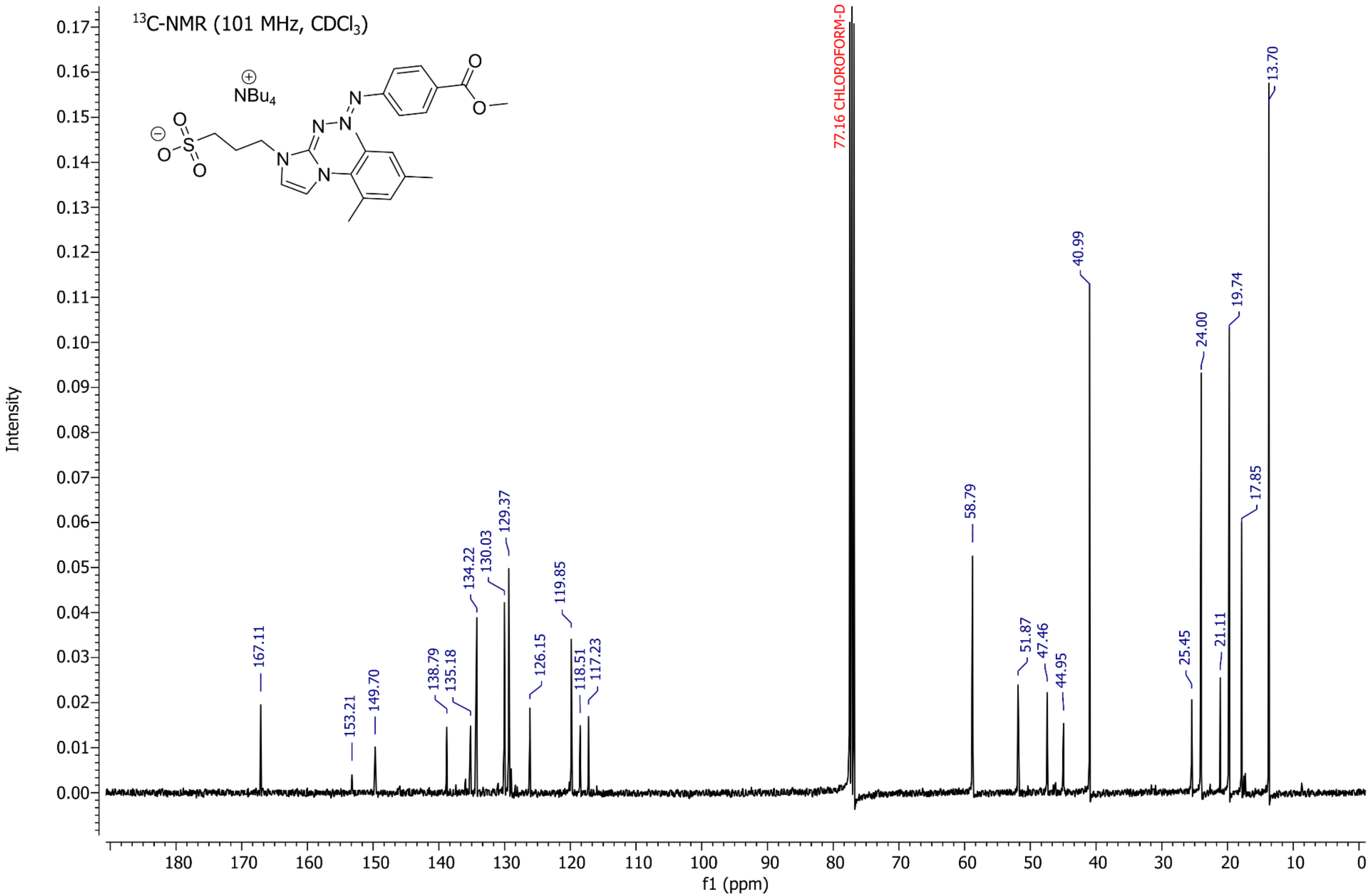

Figure S 41. ^{13}C-NMR spectrum of **7**.

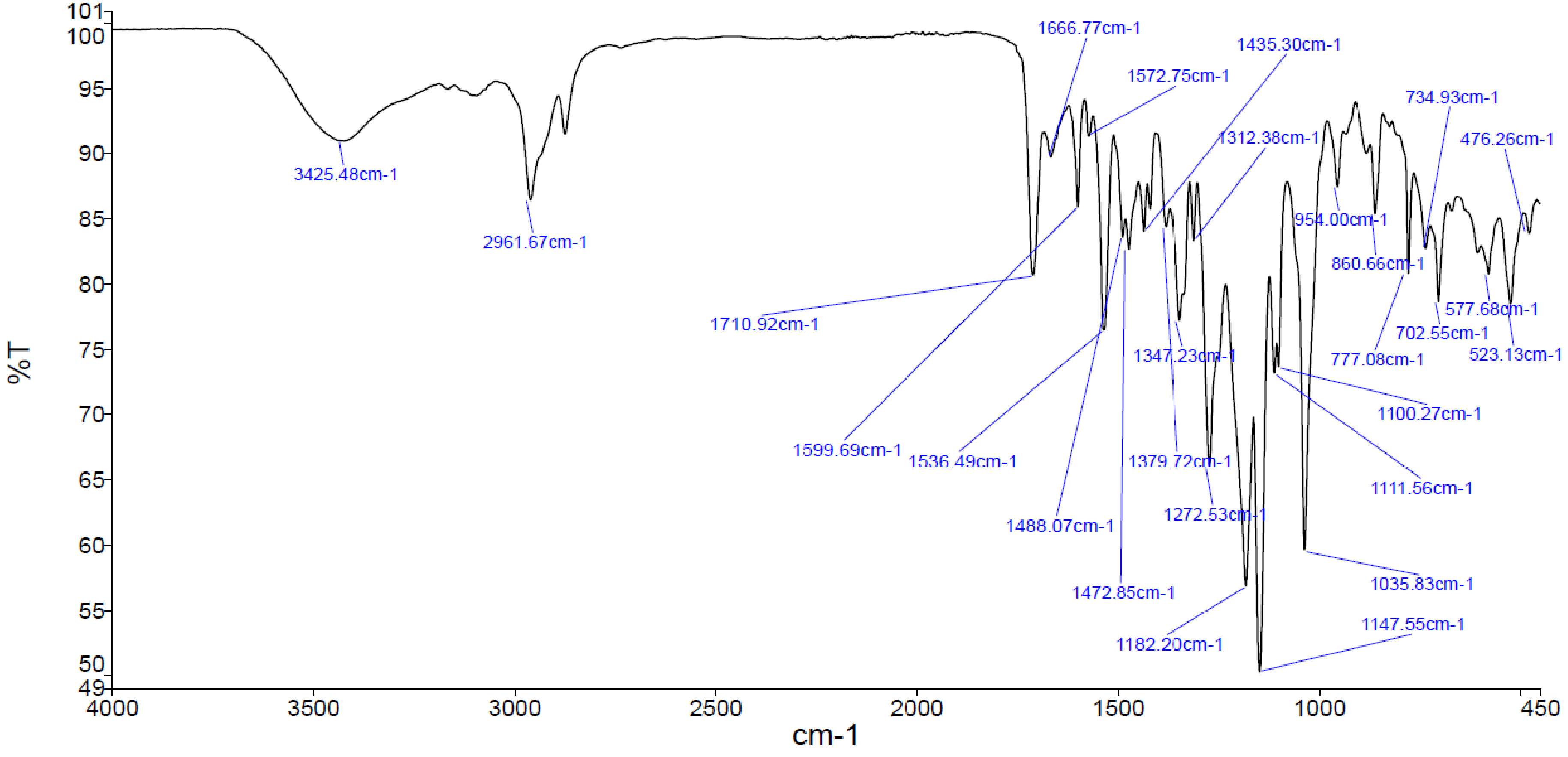

Figure S 42. FT-IR (ATR) spectrum of **7**.

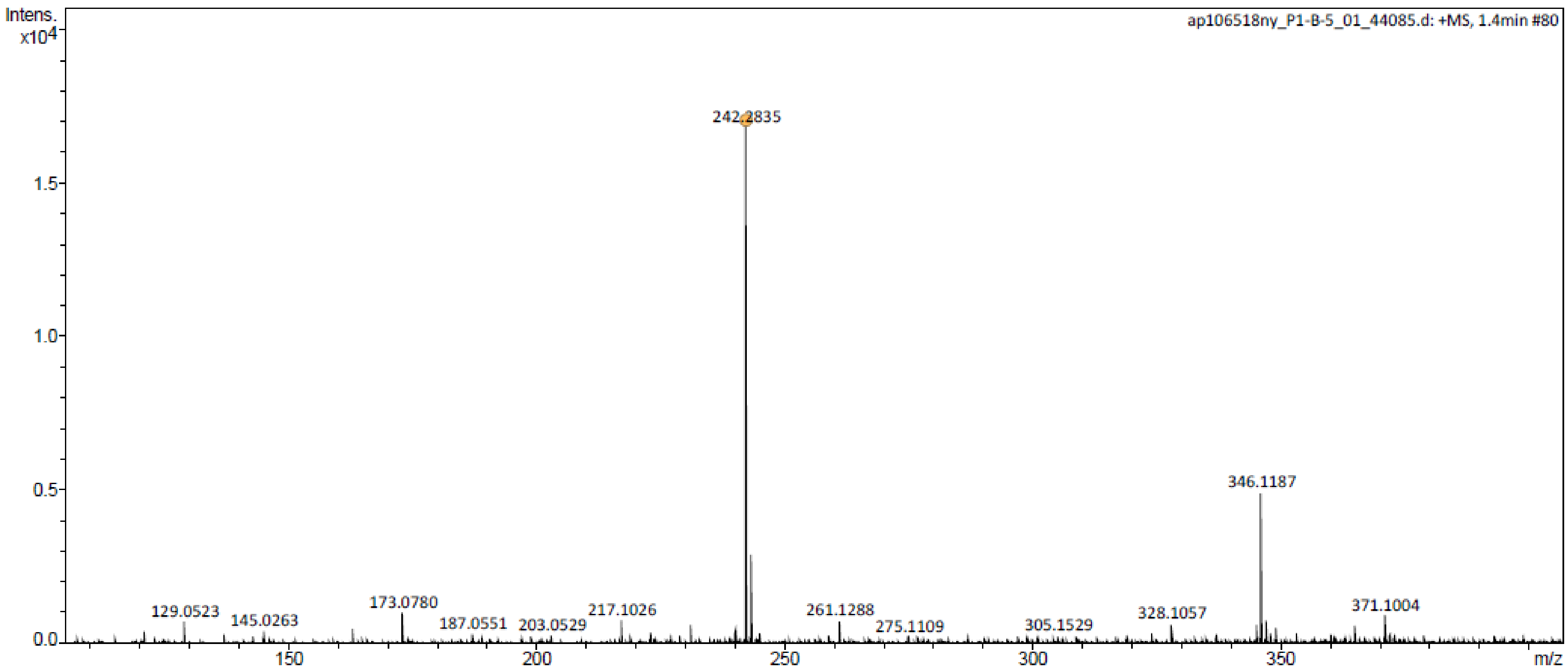

Meas. m/z	#	Ion Formula	m/z	err [ppm]	err [mDa]	mSigma	Mean err [ppm]
242.2835	1	C16H36N	242.2842	3.0	0.7	10.4	3.0

Figure S 43. Positive mode (ESI)HRMS of **7**.

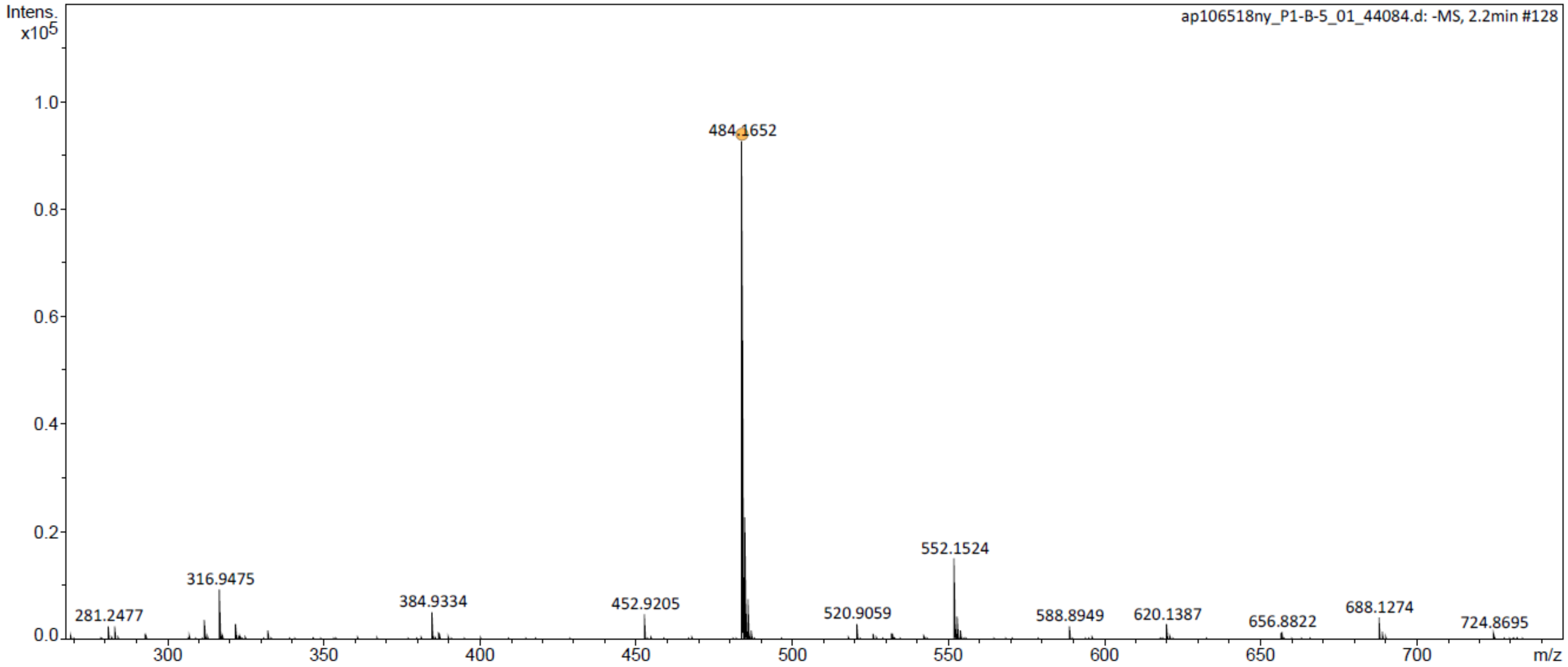

Meas. m/z	#	Ion Formula	m/z	err [ppm]	err [mDa]	mSigma	Mean err [ppm]
484.1652	1	C23H26N5O5S	484.1660	1.8	0.9	23.1	1.1

Figure S 44. Negative mode (ESI)HRMS of **7**.

Potassium 4-((E)-((E)-1-mesityl-3-(3-sulfonatopropyl)-1,3-dihydro-2H-imidazol-2-ylidene)triaz-1-en-1-yl)benzoate 8

To **7** (147 mg, 0.2 mmol) was added an ethanoic solution of KOH (56 mg, 1 mmol in 2.5 mL EtOH). The resulting solution was stirred at 50°C for 6 h, over which time a precipitate of **8** formed. The precipitate was isolated via filtration, and rinsed with EtOH. Drying *in vacuo* yielded **8** as a yellow solid in quantitative yield.

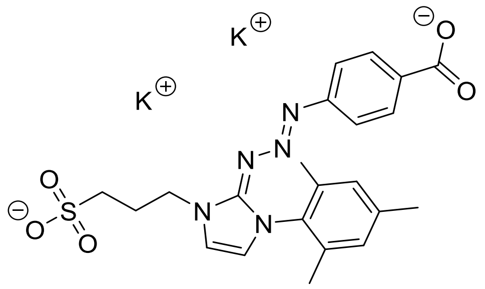

Figure S 45. The structure of **8**.

^{1}H-NMR (400 MHz, D_2O): δ_H 7.67-7.62 (m, 2H), 7.19 (d, *J* = 2.50 Hz, 1H), 7.03 (s, 2H), 6.71 (d, *J* = 2.50 Hz, 1H), 6.53-6.49 (m, 2H), 4.20 (t, *J* = 7.00 Hz, 2H), 3.02-2.95 (m, 2H), 2.34 (s, 3H), 2.34-2.28 (m, 2H), 1.94 (s, 6H).

^{13}C-NMR (101 MHz, D_2O): δ_c 175.1, 152.6, 149.8, 139.5, 134.9, 134.4, 133.4, 129.9, 129.2, 119.8, 118.4, 117.2, 47.9, 44.4, 24.4, 20.3, 16.9.

FT-IR (ATR) (υmax/cm^{-1}): 2968 (C-H stretch), 1601 (C=C stretch, aromatic), 1590 (C=C stretch, aromatic), 1536, 1347, 1177 (S=O stretch).

(ESI)HRMS: Found 470.1483, $C_{22}H_{24}N_5O_5S^-$ requires 470.1504.

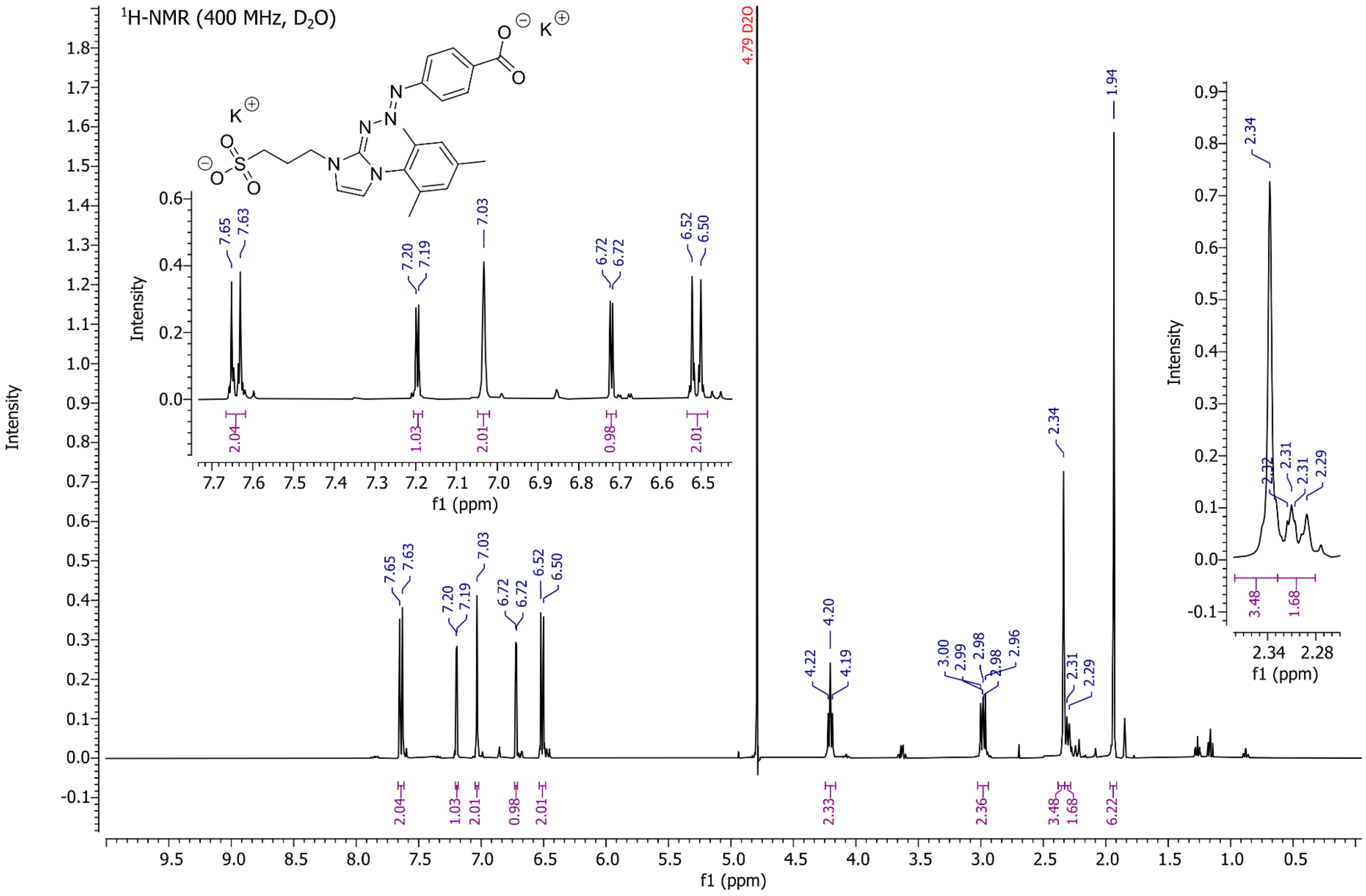

Figure S 46. ^{1}H-NMR spectrum of **8**.

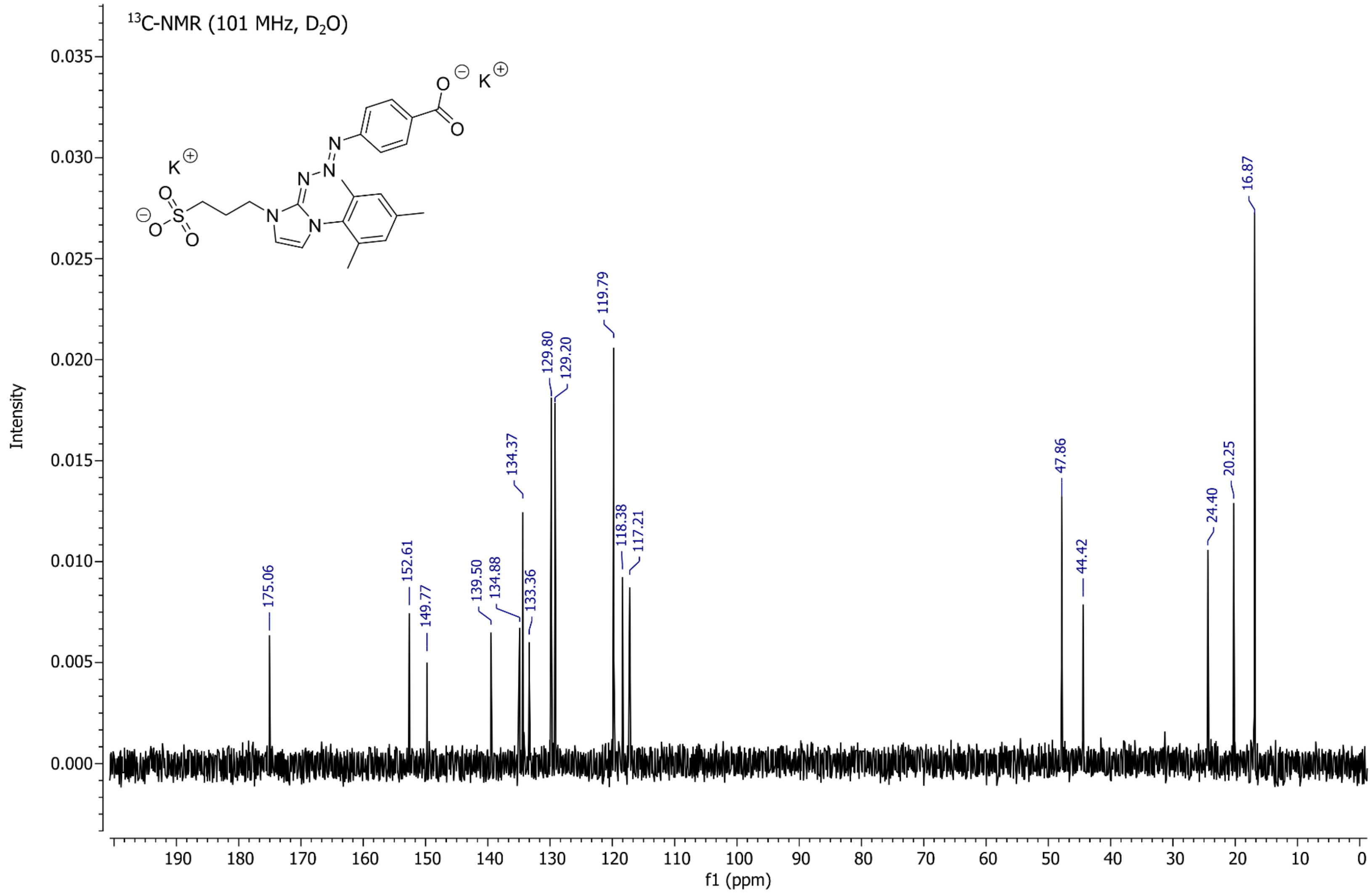

Figure S 47. ^{13}C-NMR spectrum of **8**.

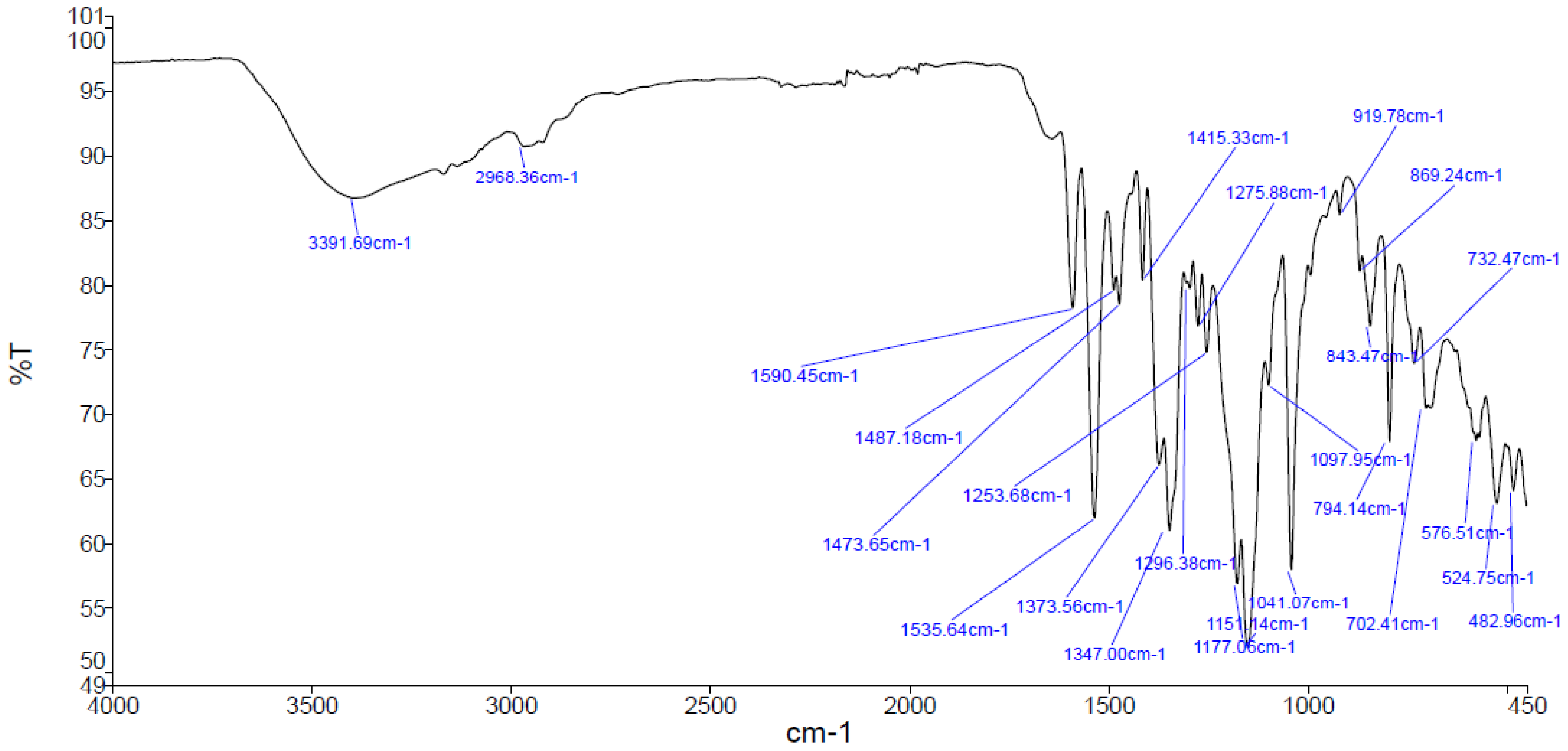

Figure S 48. FT-IR (ATR) spectrum of **8**.

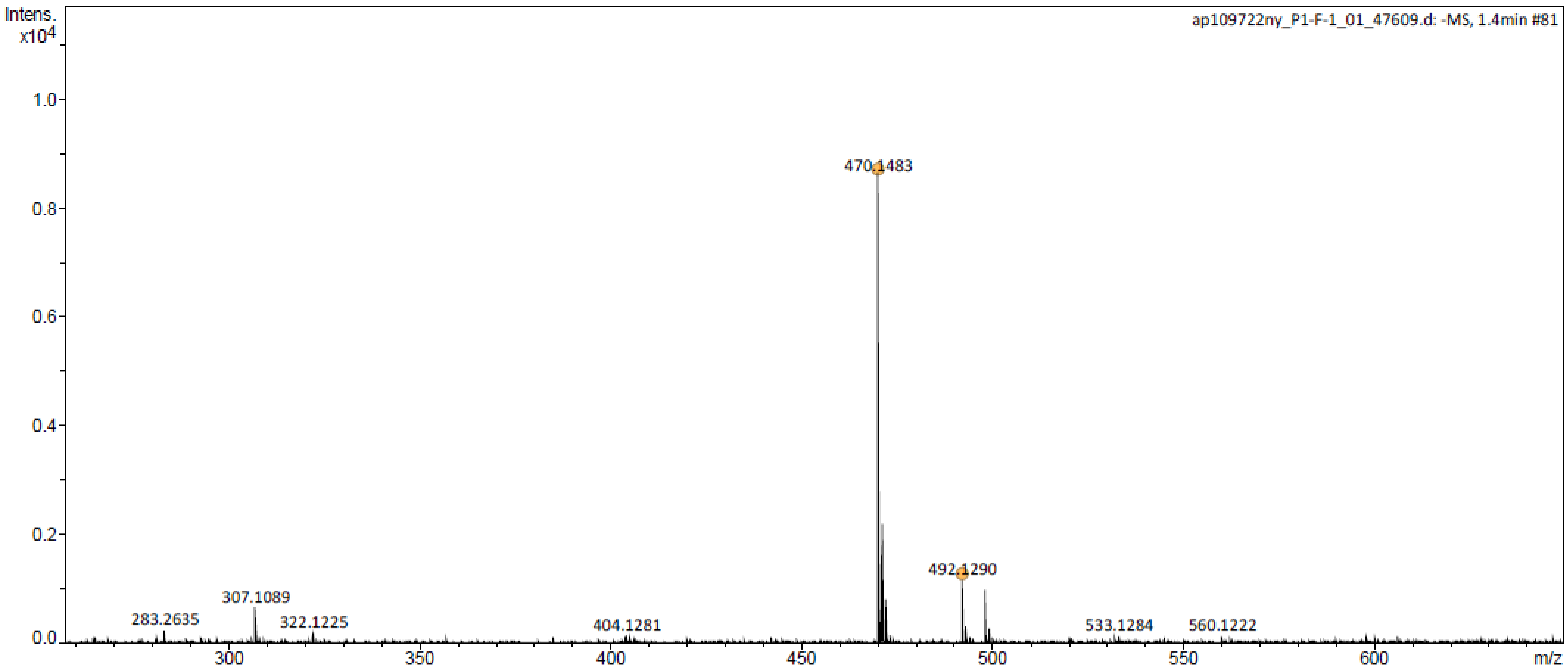

Meas. m/z	#	Ion Formula	m/z	err [ppm]	err [mDa]	mSigma	Mean err [ppm]
470.1483	1	C22H24N5O5S	470.1504	4.5	2.1	11.2	1.7
492.1290	1	C22H23N5NaO5S	492.1323	6.8	3.3	n.a.	3.0

Figure S 49. Negative mode (ESI)HRMS of **8**.

1-methyl-[4,4'-bipyridin]-1-ium iodide S2

To a stirred solution of 4,4'-bipyridine (3.00 g, 19.2 mmol) in DCM (50 mL) was added iodomethane (1.20 mL, 19.2 mmol) dropwise at rt. The resultant solution was then stirred overnight at 30°C. The reaction solution was then cooled to rt, and the precipitated product isolated by filtration and rinsed with diethyl either. This yielded a bright yellow powder which was re-dissolved in DMF and re-crystallised via the addition of diethyl ether as an anti-solvent. The solid obtained was isolated by filtration and dried *in vacuo*. This yielded 1-methyl-[4,4'-bipyridin]-1-ium iodide **S2** as bright yellow microcrystals. (4.07 g, 71%).

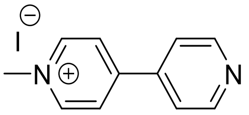

Figure S 50. The structure of **S2**.

^{1}H-NMR (400 MHz, D_2O): δ_H 8.92-8.86 (m, 2H), 8.75-8.69 (m, 2H), 8.38-8.32 (m, 2H), 7.98-7.85 (m, 2H), 4.44 (s, 3H).

^{13}C-NMR (101 MHz, D_2O): δ_C 153.4, 150.0, 145.7, 142.5, 125.8, 122.5, 48.0.

FT-IR (ATR) (υmax/cm^{-1}): 3022 (C-H stretch), 1651 (C=C stretch, aromatic), 810 (C-H bending, aromatic).

(ESI)HRMS: Found 171.0919, $C_{11}H_{11}N_2^{2+}$ requires 171.0917.

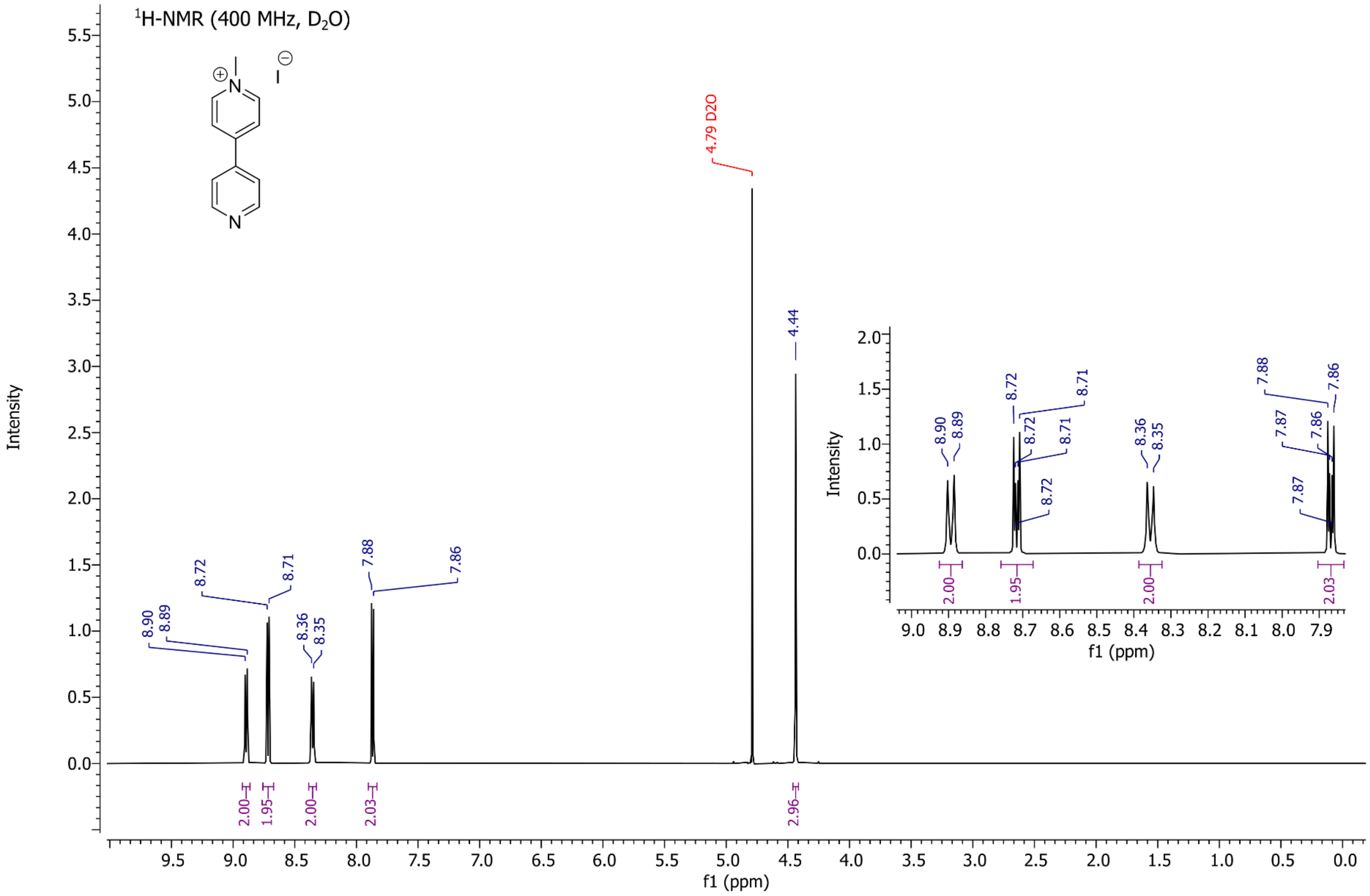

Figure S 51. ^{1}H-NMR spectrum of **S2**.

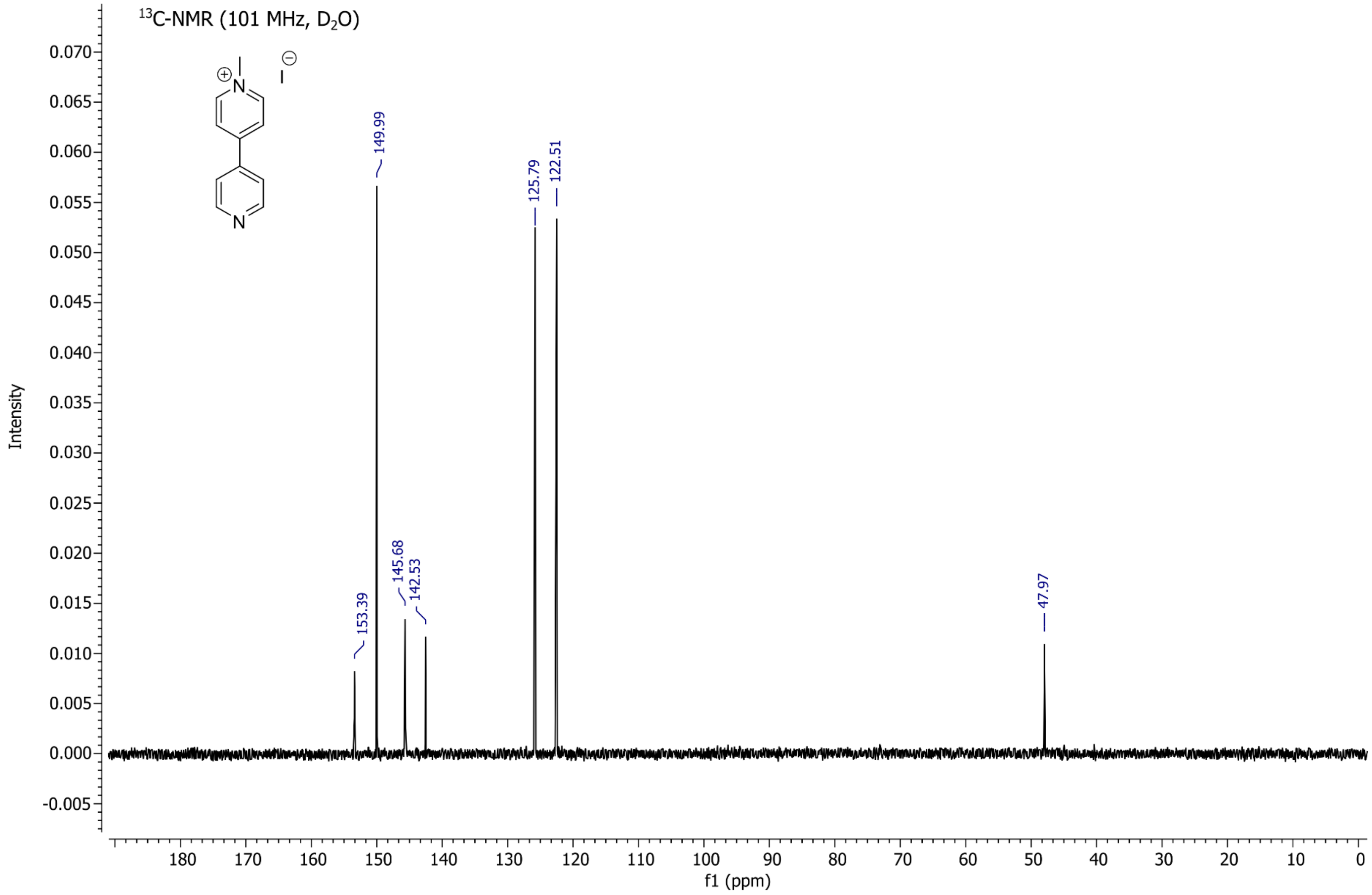

Figure S 52. ^{13}C-NMR spectrum of **S2**.

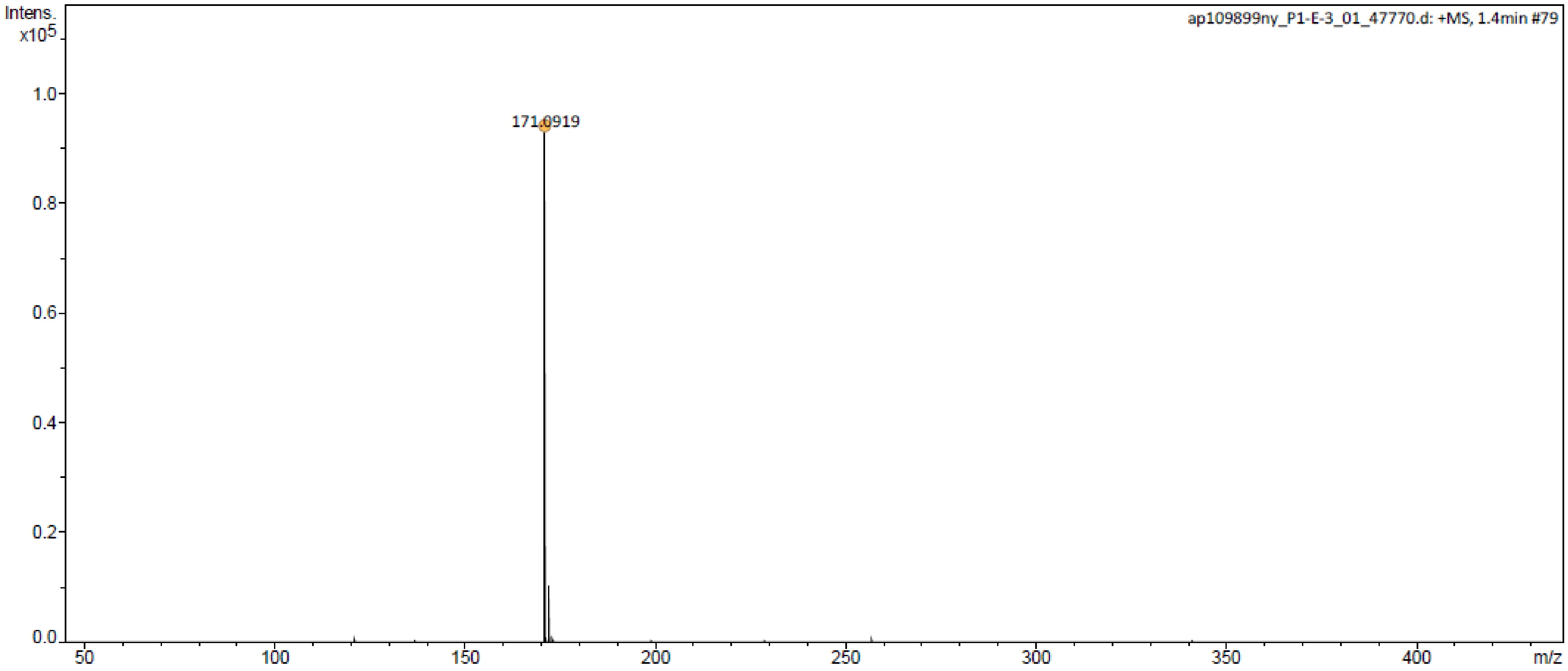

Meas. m/z	#	Ion Formula	m/z	err [ppm]	err [mDa]	mSigma	Mean err [ppm]
171.0919	1	C11H11N2	171.0917	-1.3	-0.2	9.9	-0.5

Figure S 53. Positive mode (ESI)HRMS of **S2**.

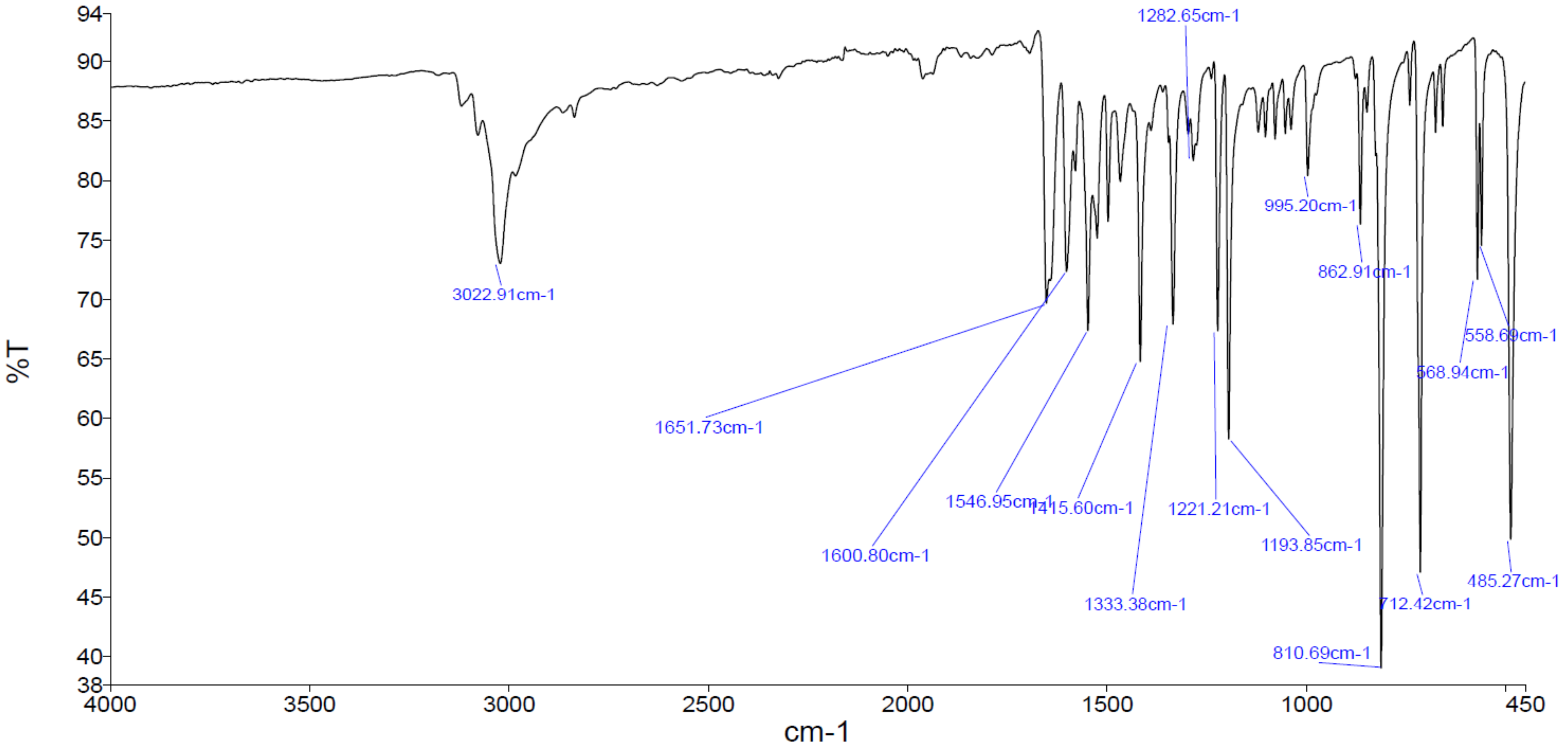

Figure S 54. FT-IR (ATR) spectrum of **S2**.

1-methyl-[4,4'-bipyridin]-1-ium hexafluorophosphate S3

S2 (6.00 g, 20.0 mmol) was dissolved in the minimum volume of water. An aqueous solution of ammonium hexafluorophosphate (16.3 g, 100 mmol, dissolved in the minimum volume of water) was then added, causing the precipitation of an off-white solid. This solid was collected via filtration, washed with additional water and then dried, yielding 1-methyl-[4,4'-bipyridin]-1-ium hexafluorophosphate **S3** as an off-white solid (6.24 g, 99%).

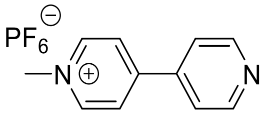

Figure S 55. The structure of **S3**.

^{1}H-NMR (400 MHz, DMSO-d$_6$): δ_H 9.16-9.09 (m, 2H), 8.90-8.83 (m, 2H), 8.64-8.56 (m, 2H), 8.06-8.00 (m, 2H), 4.38 (s, 3H).

^{13}C-NMR (101 MHz, DMSO-d$_6$): δ_C 151.9, 151.0, 146.2, 140.9, 125.0, 121.9, 47.5.

^{19}F-NMR (376 MHz, DMSO-d$_6$): δ_F -70.0 (d, J_{P-F} = 711 Hz).

FT-IR (ATR) (υmax/cm^{-1}): 3139 (C-H stretch), 1652 (C=C stretch, aromatic), 824 (P-F stretch), 555 (P-F bending).

(ESI)HRMS: Found 171.0917, $C_{11}H_{11}N_2^{2+}$ requires 171.0917; negative mode found 144.9646, PF_6^- requires 144.9647.

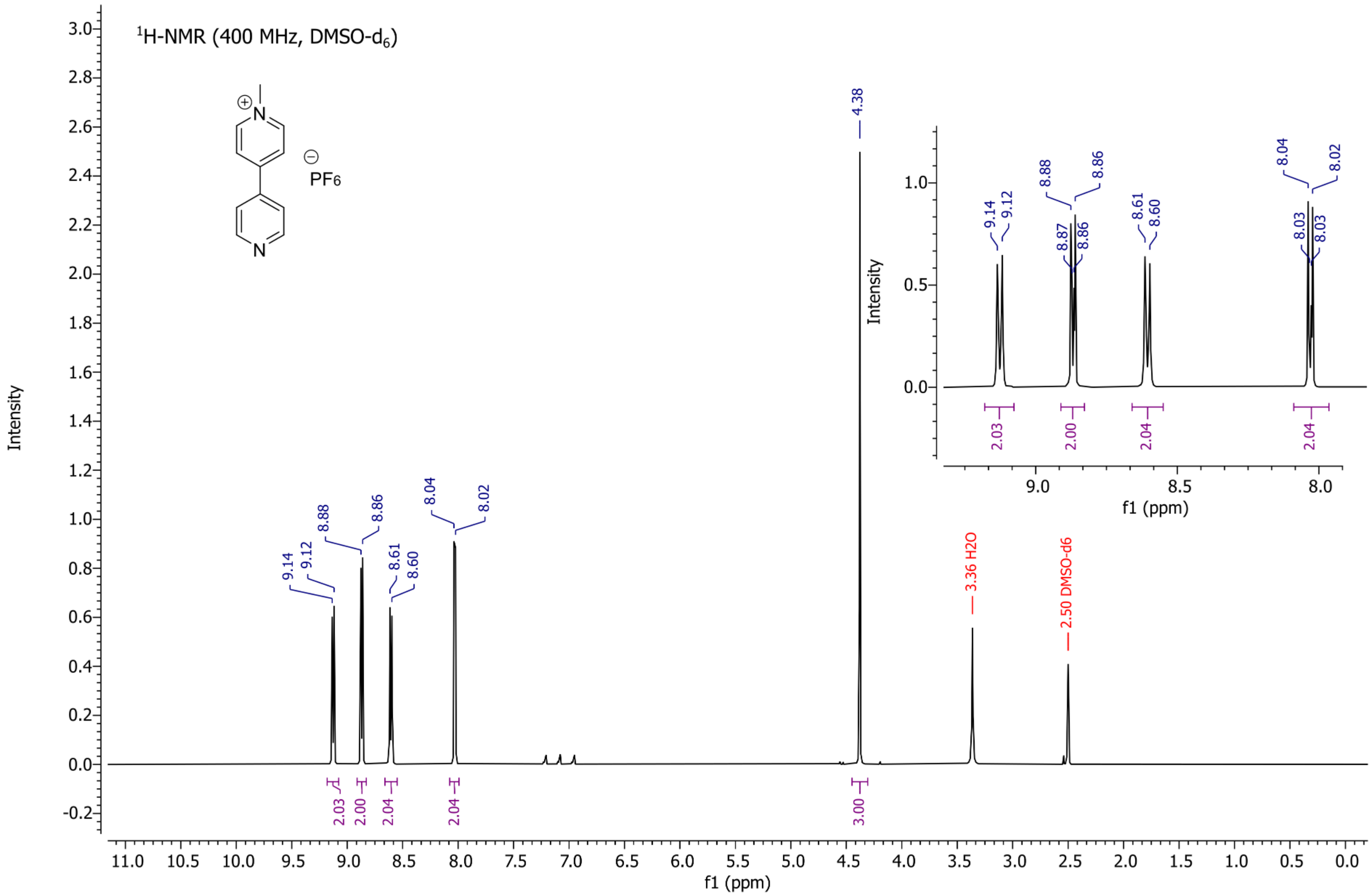

Figure S 56. ^{1}H-NMR spectrum of **S3**.

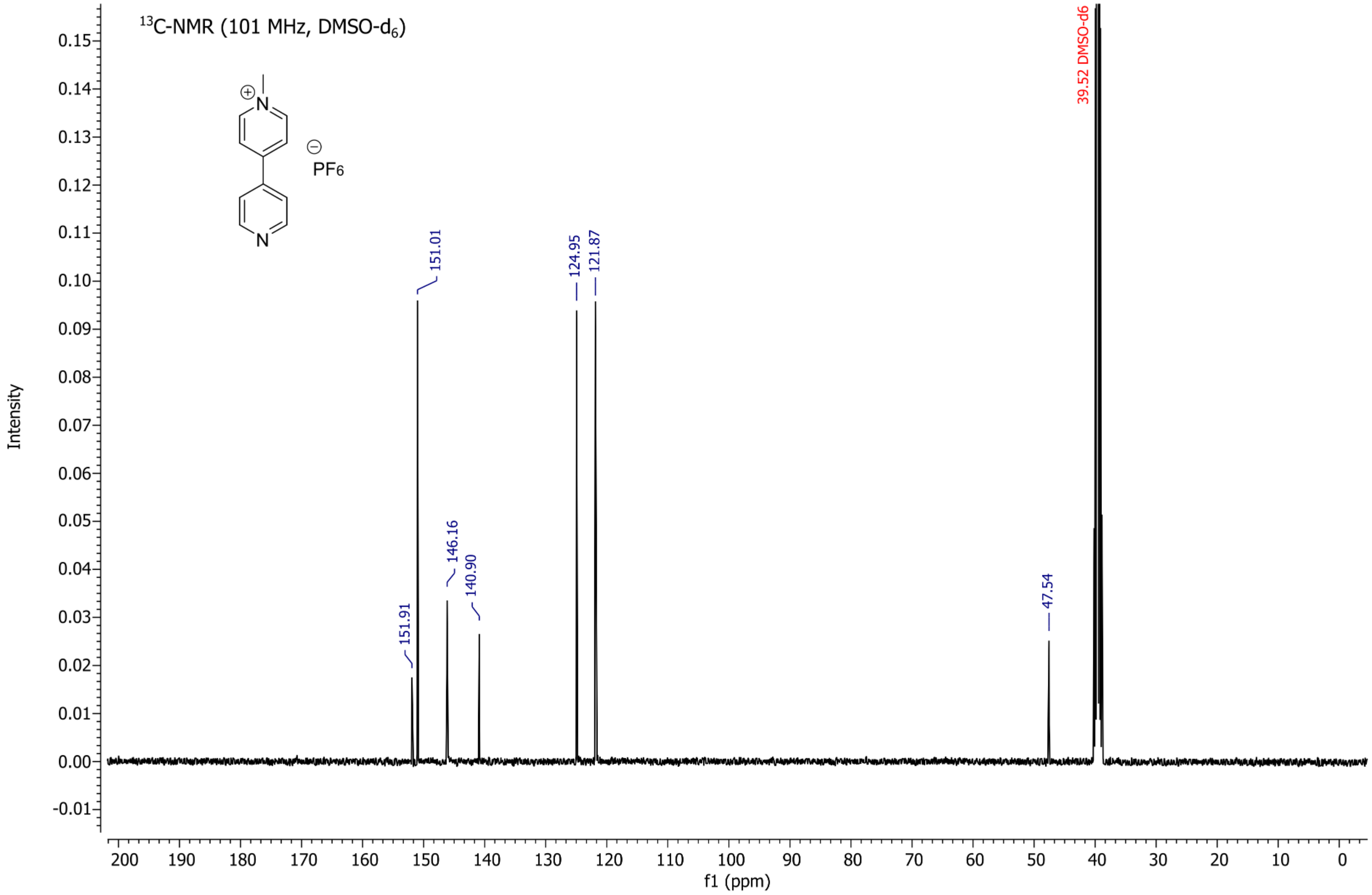

Figure S 57. ^{13}C-NMR spectrum of **S3**.

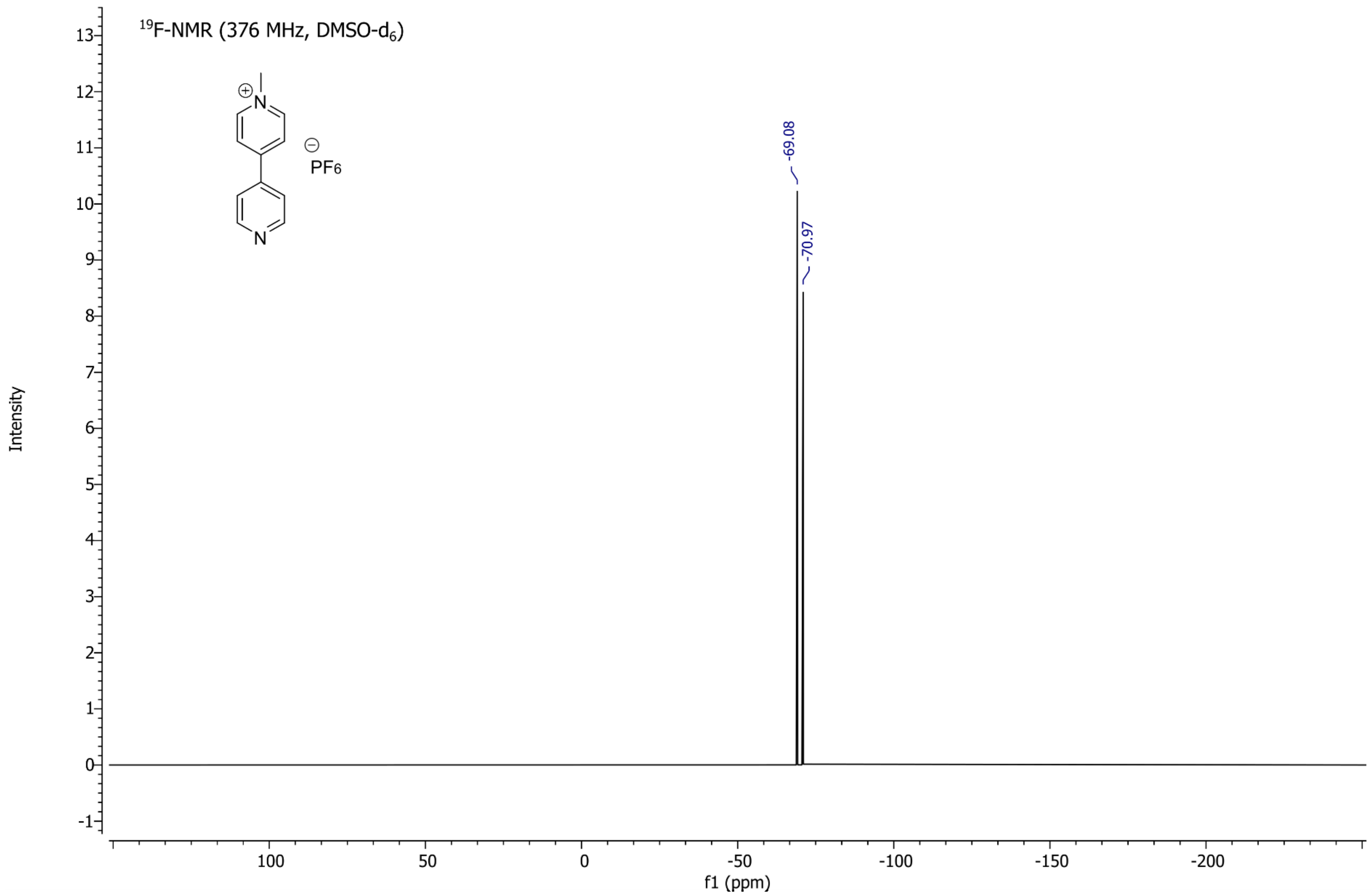

Figure S 58. ^{19}F-NMR spectrum of **S3**.

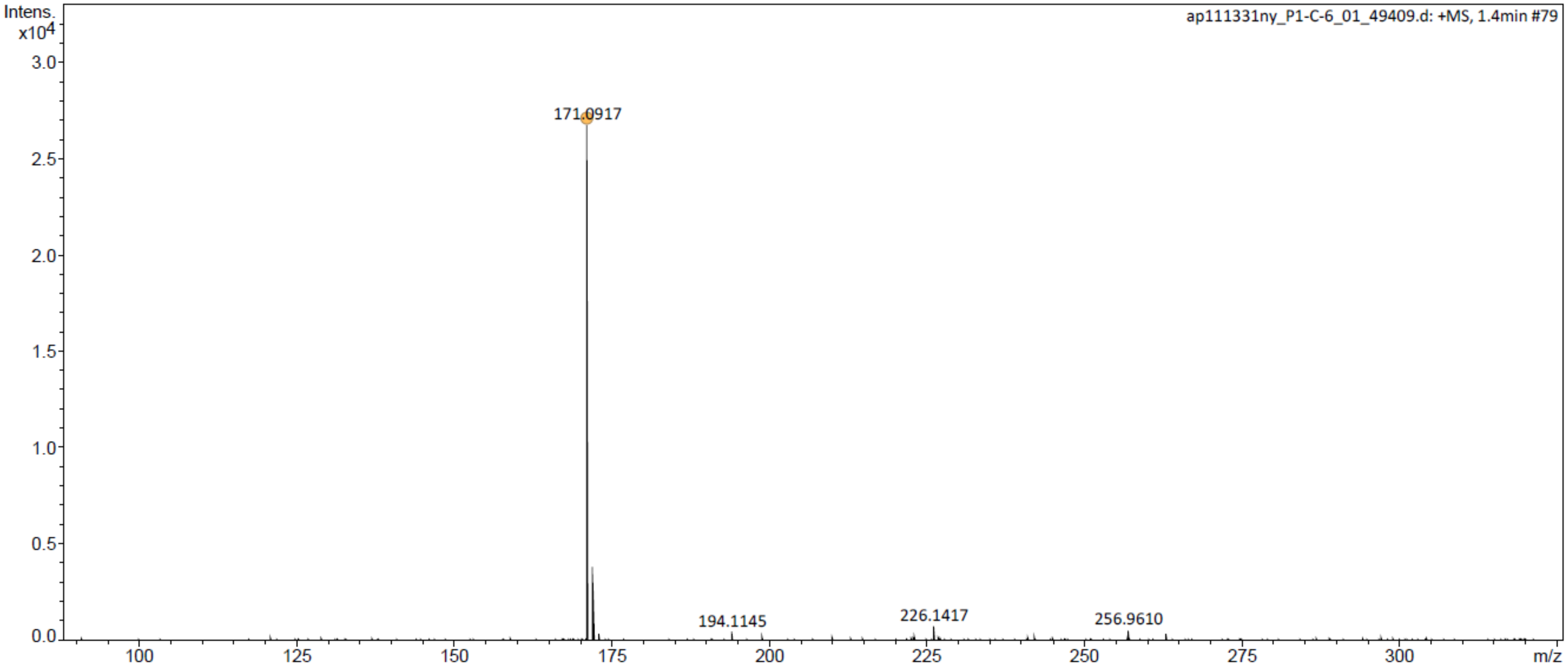

Meas. m/z	#	Ion Formula	m/z	err [ppm]	err [mDa]	mSigma	Mean err [ppm]
171.0917	1	C11H11N2	171.0917	0.0	0.0	9.2	2.4

Figure S 59. Positive mode (ESI)HRMS of **S3**.

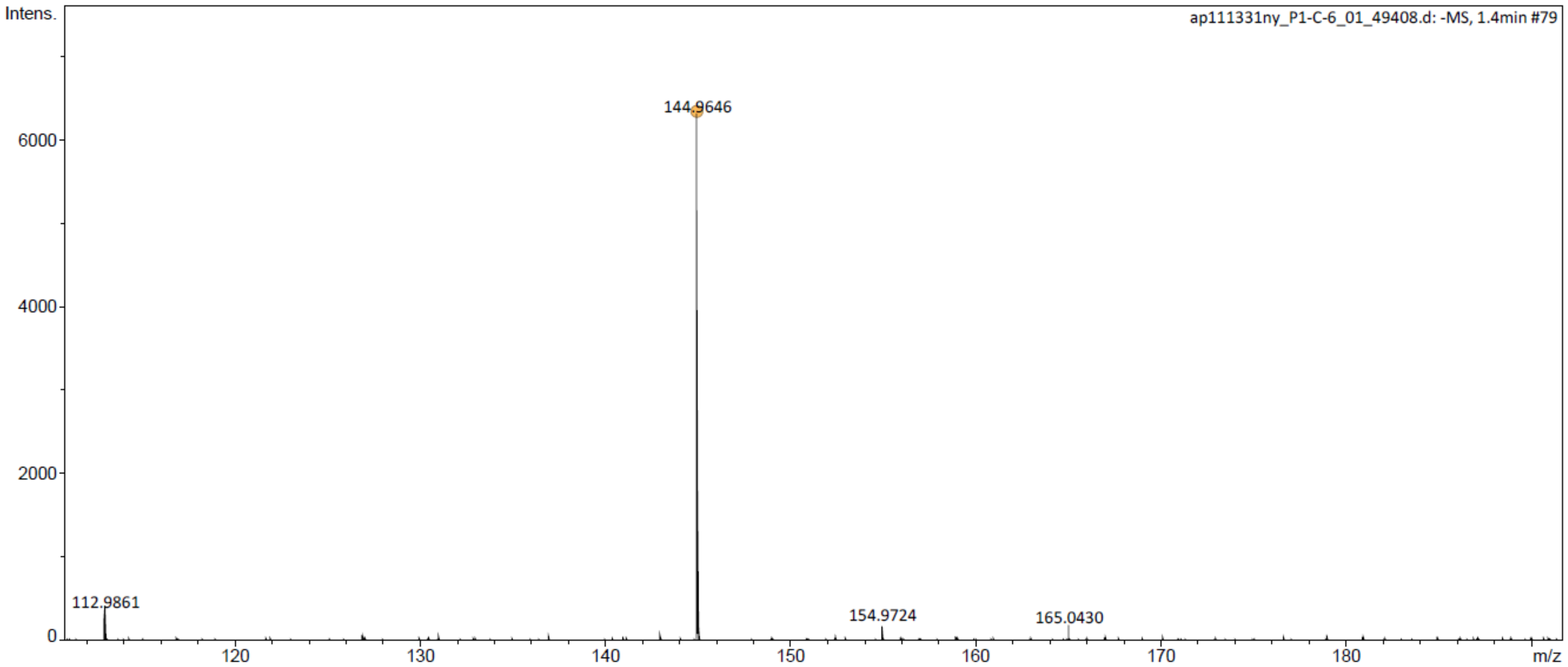

Meas. m/z	#	Ion Formula	m/z	err [ppm]	err [mDa]	mSigma	Mean err [ppm]
144.9646	1	F6P	144.9647	0.7	0.1	n.a.	0.7

Figure S 60. Negative mode (ESI)HRMS of **S3**.

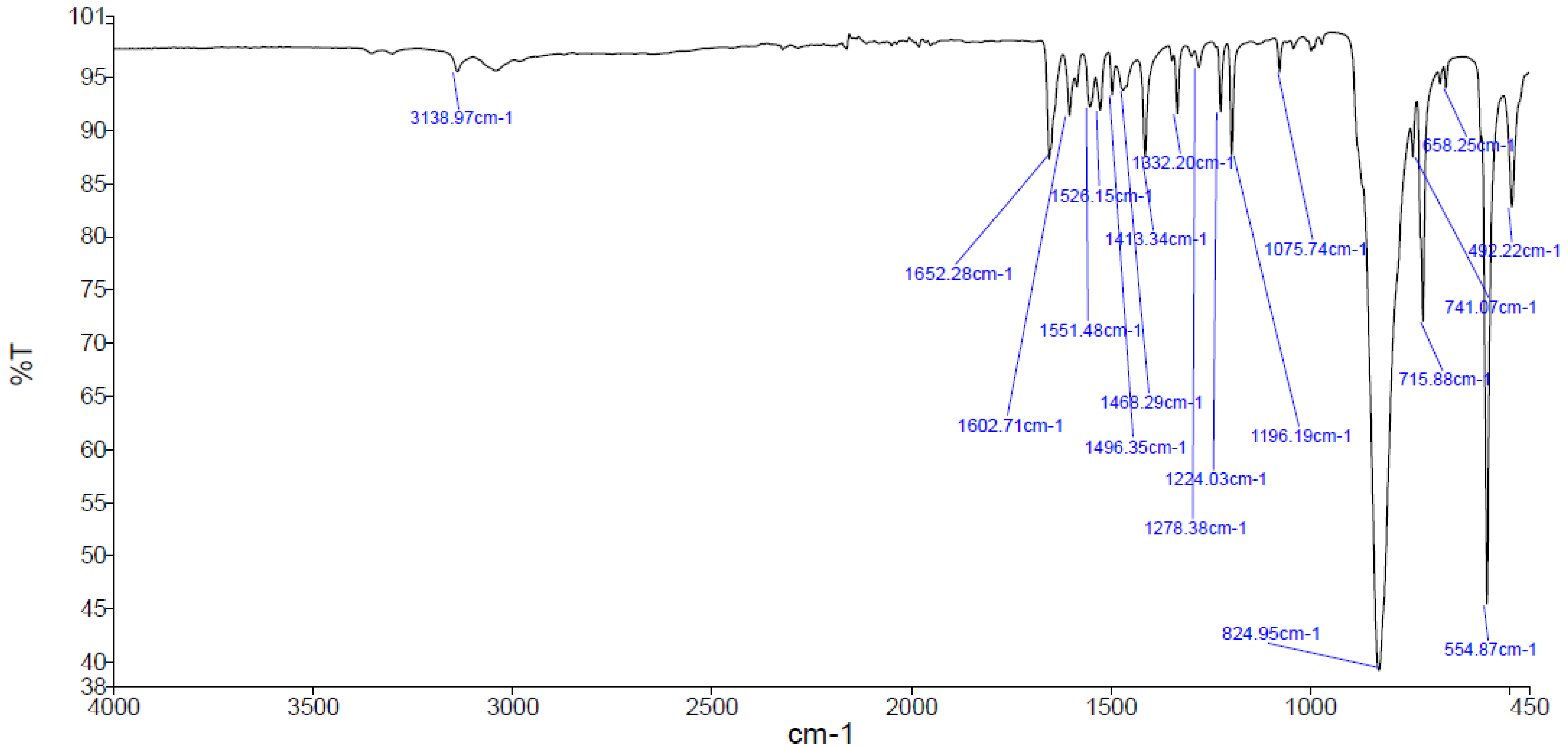

Figure S 61. FT-IR (ATR) spectrum of **S3**.

1-(2-ammonioethyl)-1'-methyl-[4,4'-bipyridine]-1,1'-diium hexafluorophosphate 9

To **S3** (0.316 g, 8.54 mmol) in MeCN (250 mL) was added 2-bromoethylamine hydrobromide (3.50 g, 17.1 mmol). The resultant mixture was stirred at reflux under N_2 for 18 h, after which time the solution was allowed to cool to rt and the solids removed via filtration. These solids were dissolved in the minimum volume of water. EtOH was then added, causing a product to precipitate as a fine off-white powder which was isolated by filtration. This product was then re-dissolved in the minimum volume of water, and a saturated aqueous solution of ammonium hexafluorophosphate was then added dropwise, with stirring, until no more precipitate was observed forming. This precipitate was then isolated by filtration and recrystalised from hot water to yield the desired product **9** as white microcrystals (0.684 g, 12%).

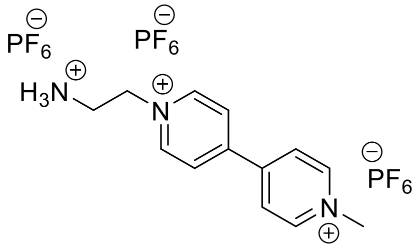

Figure S 62. The structure of **9**.

^{1}H-NMR (400 MHz, DMSO-d$_6$): δ_H 9.32-9.24 (m, 4H), 8.86-8.81 (m, 2H), 8.78-8.73 (m, 2H), 7.93 (br s, 3H), 4.89 (t, *J* = 5.55 Hz, 2H), 4.44 (s, 3H), 3.56 (t, *J* = 5.80 Hz, 2H).

^{13}C-NMR (101 MHz, DMSO-d$_6$): δ_c 149.2, 147.8, 146.8, 146.7, 126.4, 126.0, 58.2, 48.1, 39.0.

^{19}F-NMR (376 MHz, DMSO-d$_6$): δ_F -70.0 (d, $J_{P\text{-}F}$ = 711 Hz).

^{31}P-NMR (162 MHz, DMSO-d$_6$): δ_F -143.6 (sept, $J_{P\text{-}F}$ = 711 Hz).

FT-IR (ATR) (υmax/cm^{-1}): 3616 (N-H stretch), 3295 (C-H stretch), 2909 (C-H stretch), 1644 (C=C stretch, aromatic), 814 (P-F stretch), 553 (P-F bending).

(ESI)HRMS: Positive mode found 214.1338, $C_{13}H_{16}N_3^+$ requires 214.1339; negative mode found 144.9642, PF_6^- requires 144.9647.

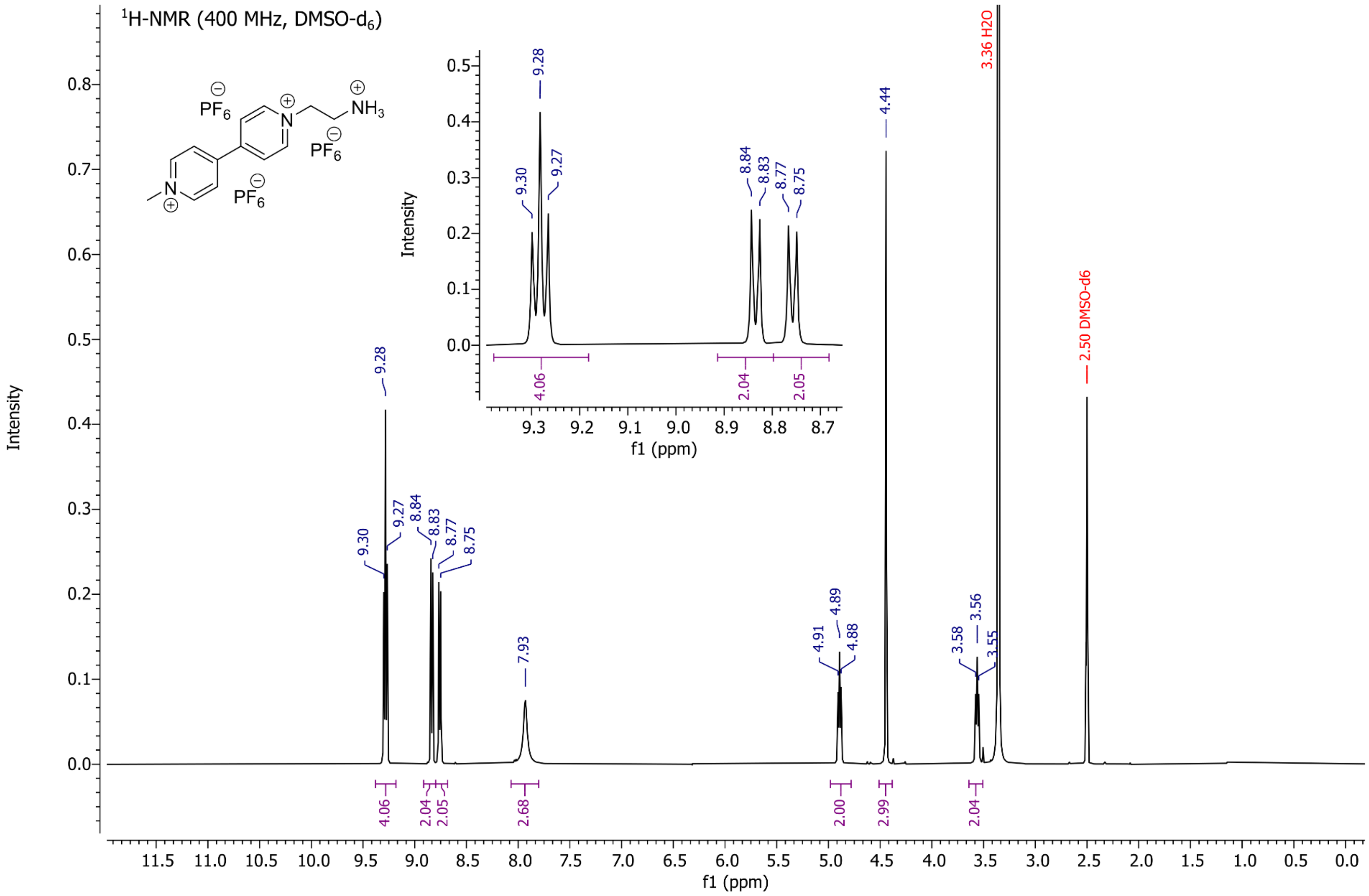

Figure S 63. ^{1}H-NMR spectrum of **9**.

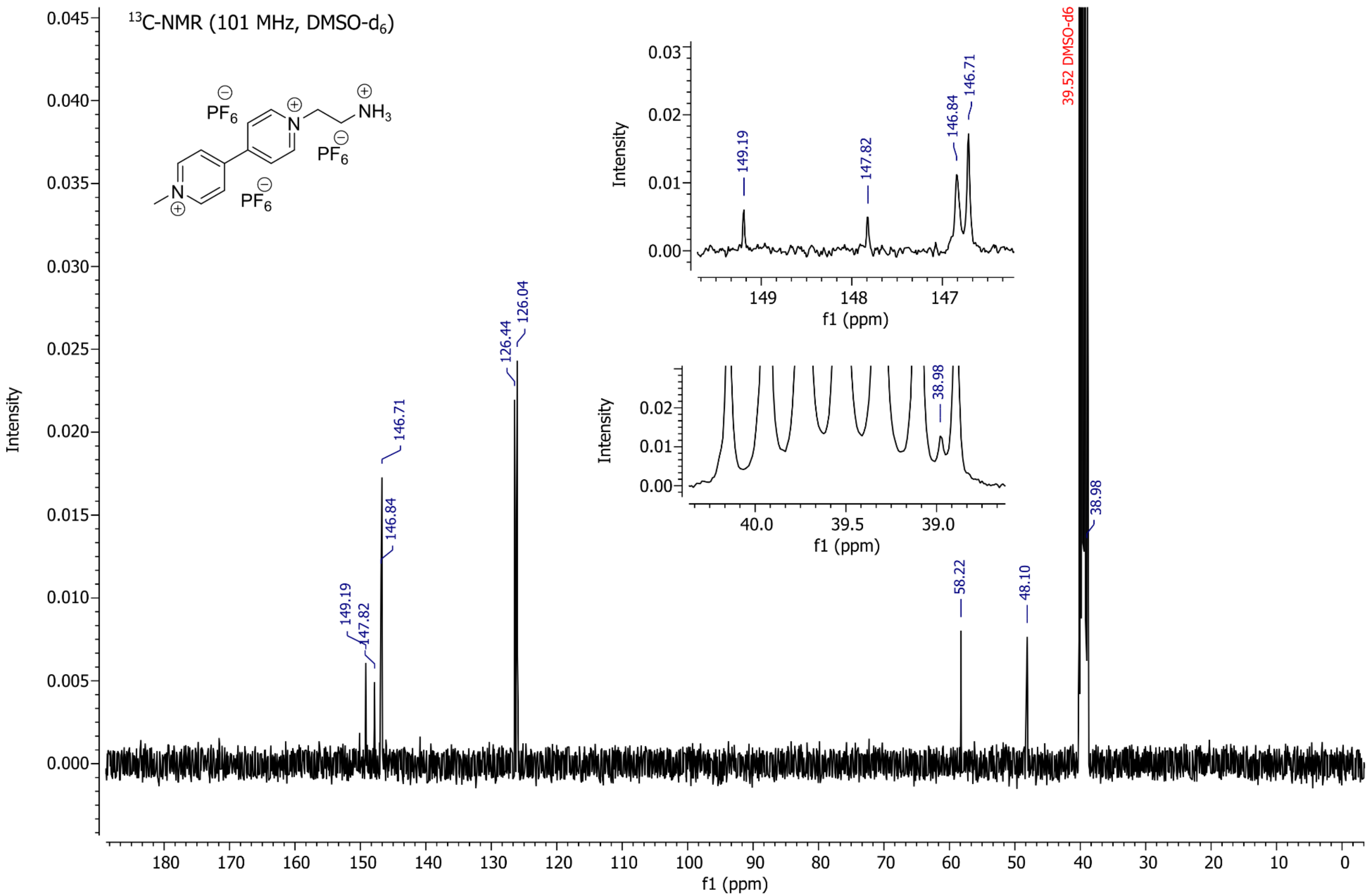

Figure S 64. ^{13}C-NMR spectrum of **9**.

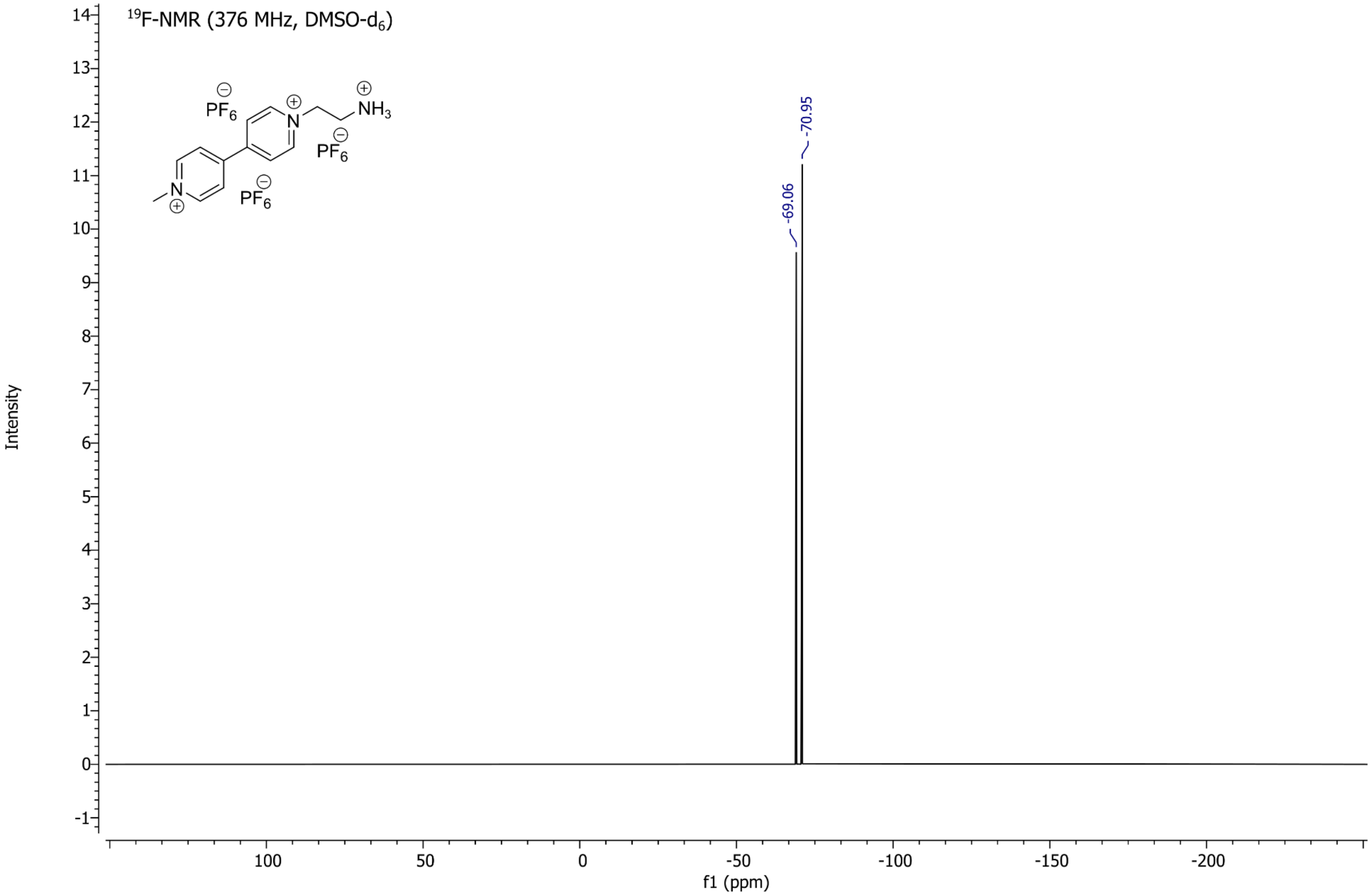

Figure S 65. ^{19}F-NMR spectrum of **9**.

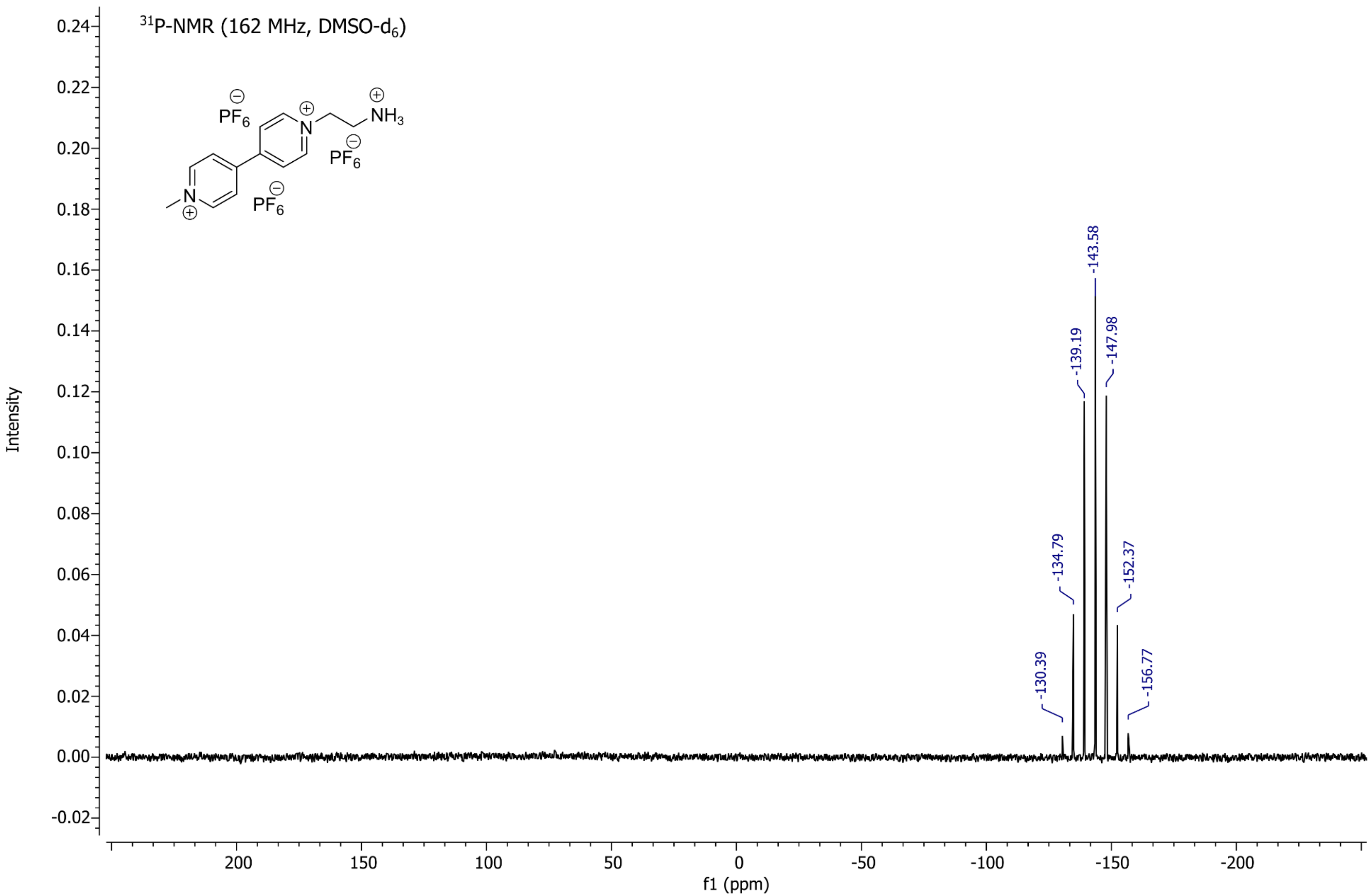

Figure S 66. ^{31}P-NMR spectrum of **9**.

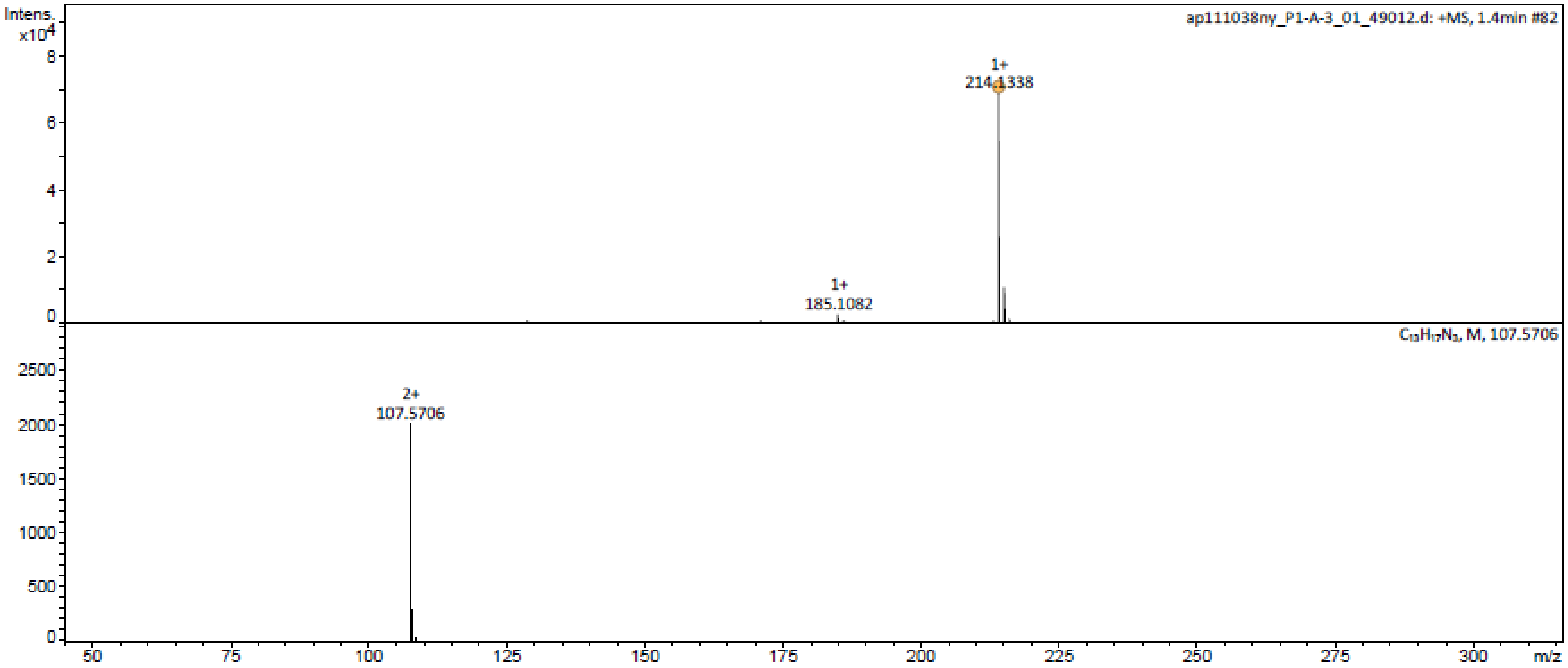

Meas. m/z	#	Ion Formula	m/z	err [ppm]	err [mDa]	mSigma	Mean err [ppm]
214.1338	1	C13H16N3	214.1339	0.4	0.1	2.6	1.5

Figure S 67. Positive mode (ESI)HRMS of **9**.

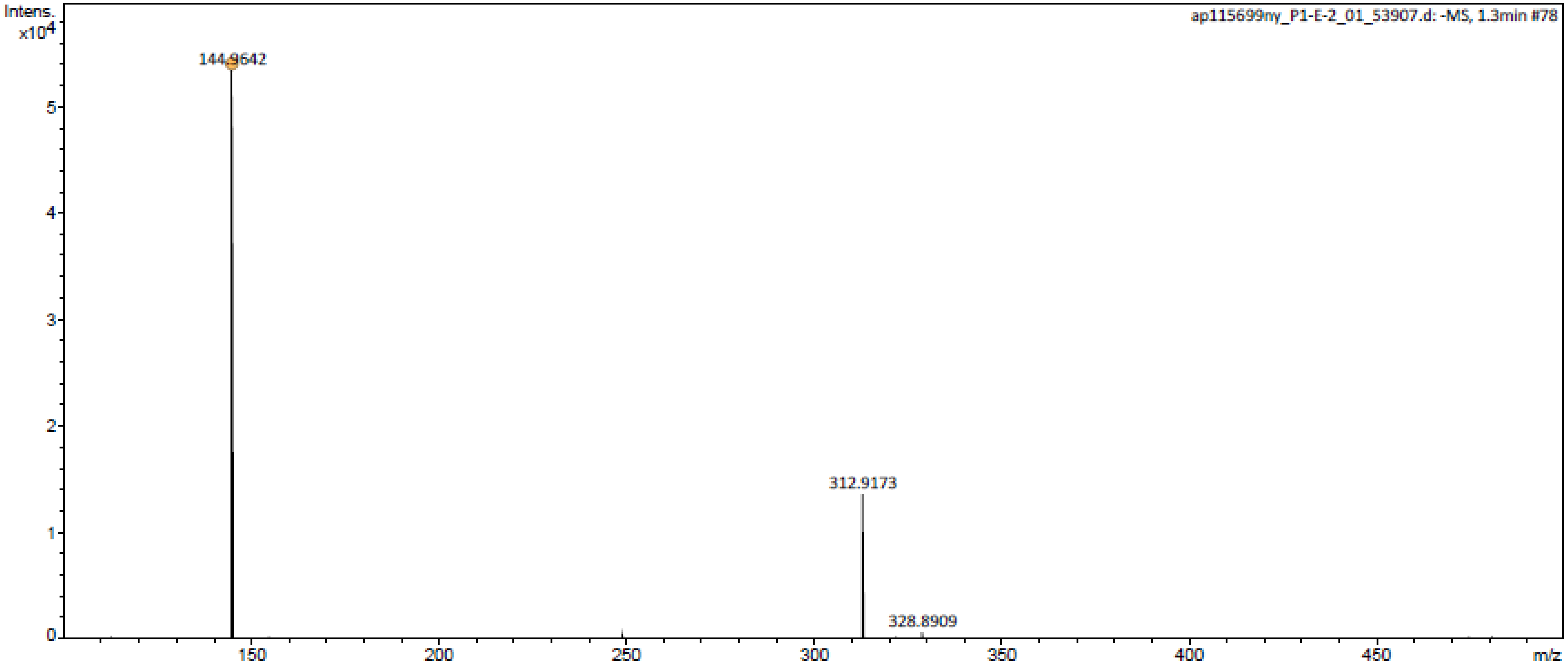

Meas. m/z	#	Ion Formula	m/z	err [ppm]	err [mDa]	mSigma	Mean err [ppm]
144.9642	1	F6P	144.9647	3.8	0.6	n.a.	3.8

Figure S 68. Negative mode (ESI)HRMS of **9**.

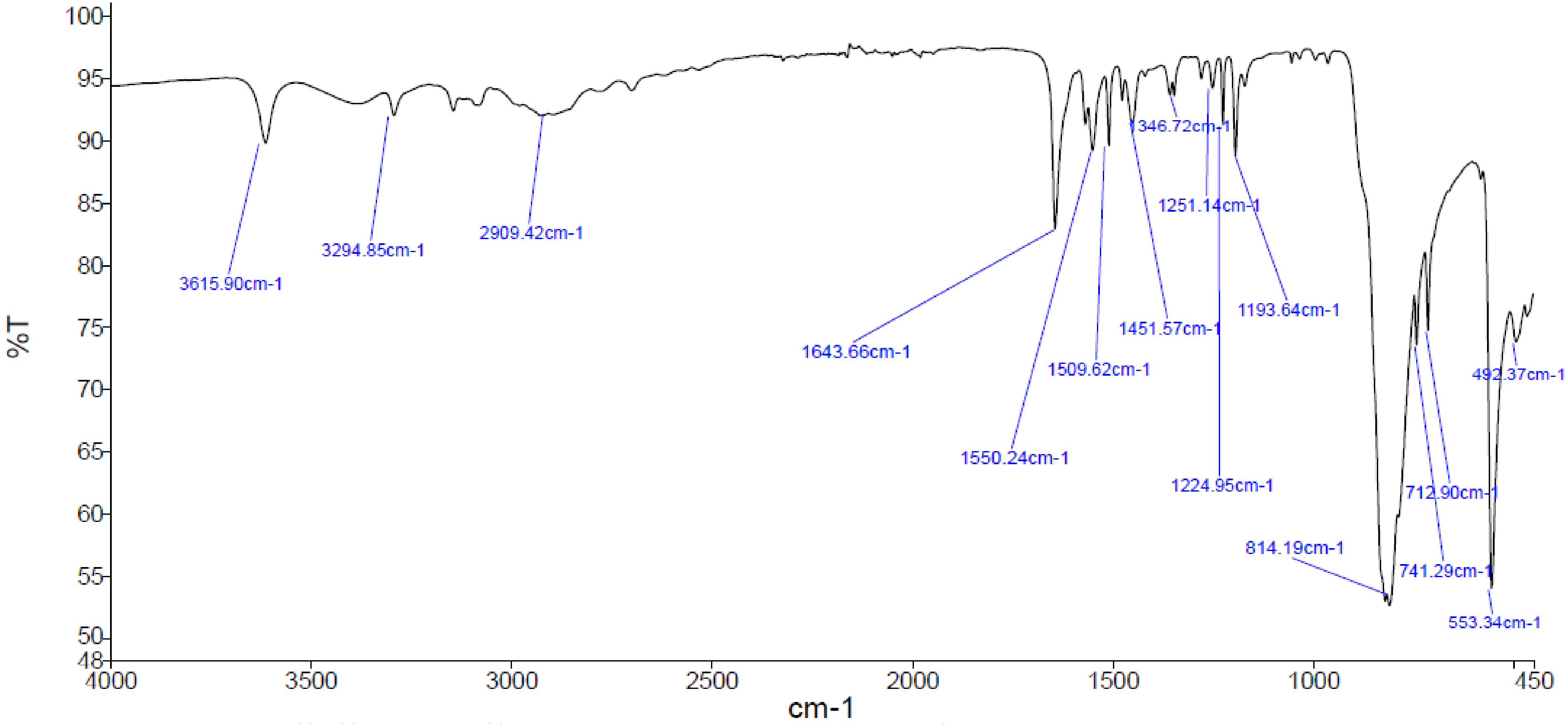

Figure S 69. FT-IR (ATR) spectrum of **9**.

3-((Z)-3-mesityl-2-((E)-(4-((2-(1'-methyl-[4,4'-bipyridin]-1,1'-diium-1-yl)ethyl)carbamoyl)phenyl)triaz-2-en-1-ylidene)-2,3-dihydro-1H-imidazol-1-yl)propane-1-sulfonate hexafluorophosphate 10

8 (55 mg, 0.1 mmol) was dissolved in DMSO (0.5 mL). HCTU (62 mg, 0.15 mmol) was then added, and the resultant solution stirred; a colour change from yellow to orange occurred almost instantly as the activated ester derivative of **8** formed. **9** (66 mg, 0.1 mmol) was then added to the stirred solution, whereafter a solution of DIPEA (35 µL, 0.2 mmol) in THF (1 mL) was added dropwise over the course of 2 min. The reaction solution was stirred for 2 h in darkness, after which time MeCN (3 mL) was added. Diethyl ether was then dropwise until a precipitate formed. This precipitate was pelleted via centrifugation and the supernatant discarded. The pelleted precipitate was then dissolved in water and purified via reverse phase flash column chromatography using a C18Aq RediSep® Rf Gold column over a gradient of water to MeCN. Fractions containing the desired product were flash-frozen using liquid nitrogen and lyophilized, yielding **10** as a fluffy intense yellow solid (40 mg, 49%).

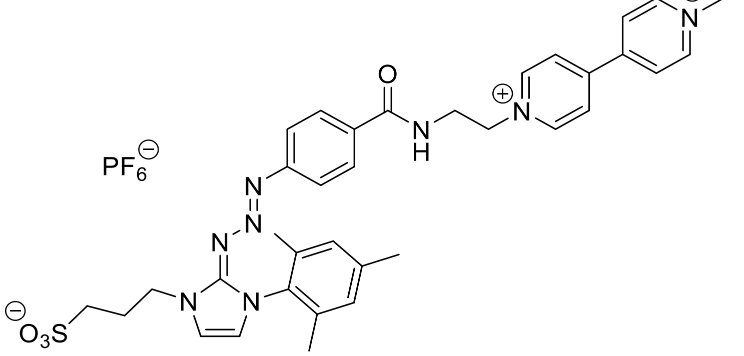

Figure S 70. The structure of **10**.

^{1}H-NMR (400 MHz, DMSO-d$_6$): δ_H 9.48-9.31 (m, 2H), 9.30-9.25 (m, 2H), 8.83-8.78 (m, 2H), 8.78-8.74 (m, 2H), 8.52 (t, *J* = 5.90 Hz, 1H), 7.47-7.41 (m, 2H), 7.37 (d, *J* = 2.45 Hz, 1H), 7.01 (s, 2H), 6.94 (d, *J* = 2.45 Hz, 1H), 6.60-6.56 (m, 2H), 4.84 (br t, *J* = 5.00 Hz, 2H), 4.43 (s, 3H), 4.10 (t, *J* = 7.00 Hz, 2H), 3.88 (br dt, *J* = 5.90, 5.00 Hz, 2H), 2.48-2.43 (m, 2H), 2.27 (s, 3H), 2.14-2.00 (2H, m), 1.93 (s, 6H).

^{13}C-NMR (101 MHz, DMSO-d$_6$): δ_C 166.8, 154.6, 150.4, 148.6, 147.9, 146.7, 146.4, 137.7, 135.0, 134.0, 129.3, 128.9, 127.6, 126.2, 126.0, 119.8, 117.9, 117.1, 61.0, 48.2, 48.0, 45.3, 25.6, 20.6, 17.4.

^{19}F-NMR (376 MHz, DMSO-d$_6$): δ_F -70.0 (d, $J_{P\text{-}F}$ = 711 Hz).

^{31}P-NMR (162 MHz, DMSO-d$_6$): δ_F -143.6 (sept, $J_{P\text{-}F}$ = 711 Hz).

FT-IR (ATR) (υmax/cm^{-1}): 3333 (C-H stretch), 2926 (C-H stretch), 1720 (C=O stretch), 1641 (C=C stretch, aromatic), 1536, 1181 (S=O stretch), 839 (P-F stretch), 557 (P-F bending).

(ESI)HRMS: Positive mode found 667.2821, $C_{35}H_{39}N_8O_4S^+$ requires 667.2809; negative mode found 144.9640, PF_6^- requires 144.9647.

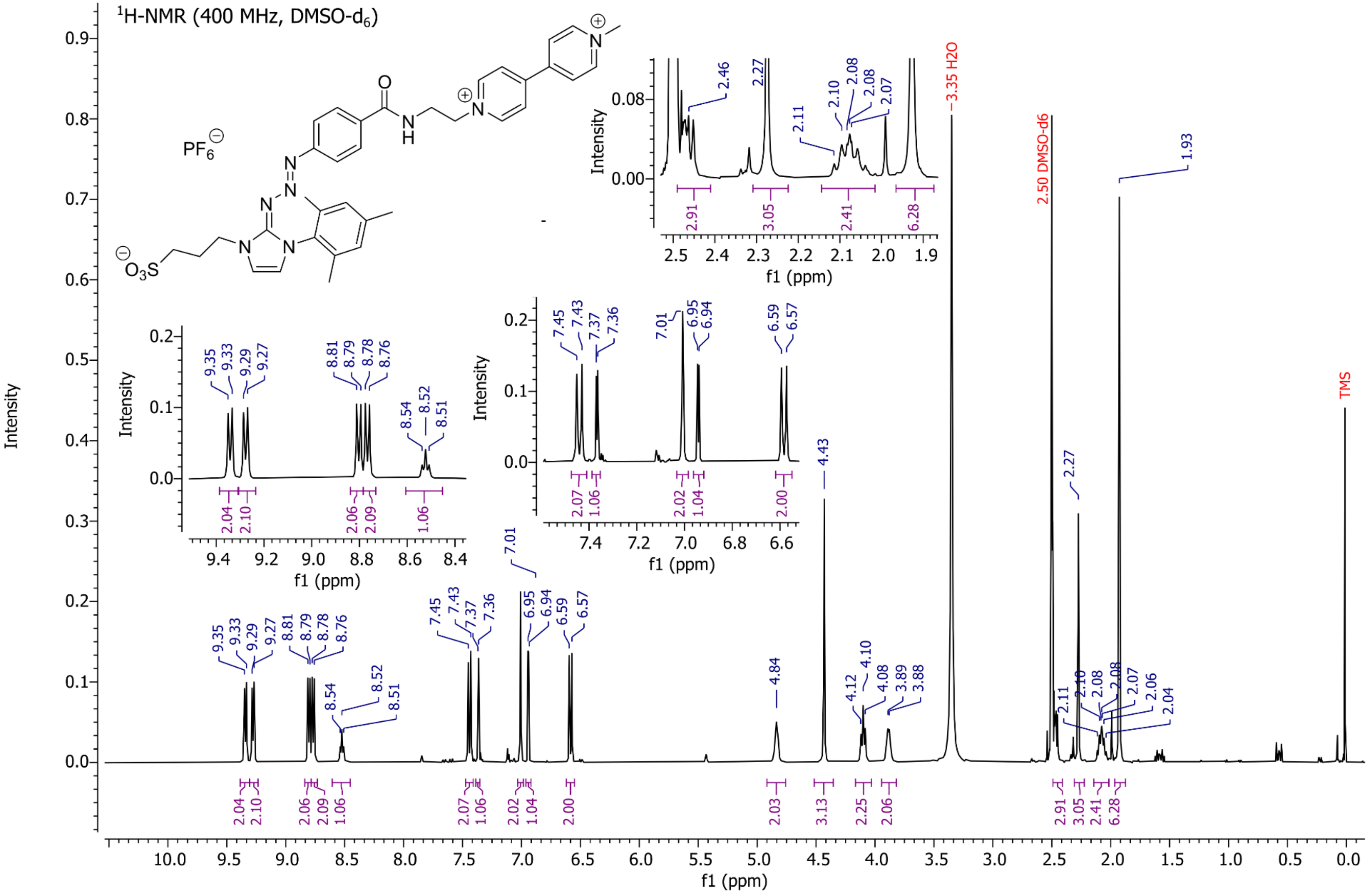

Figure S 71. ^{1}H-NMR spectrum of **10**.

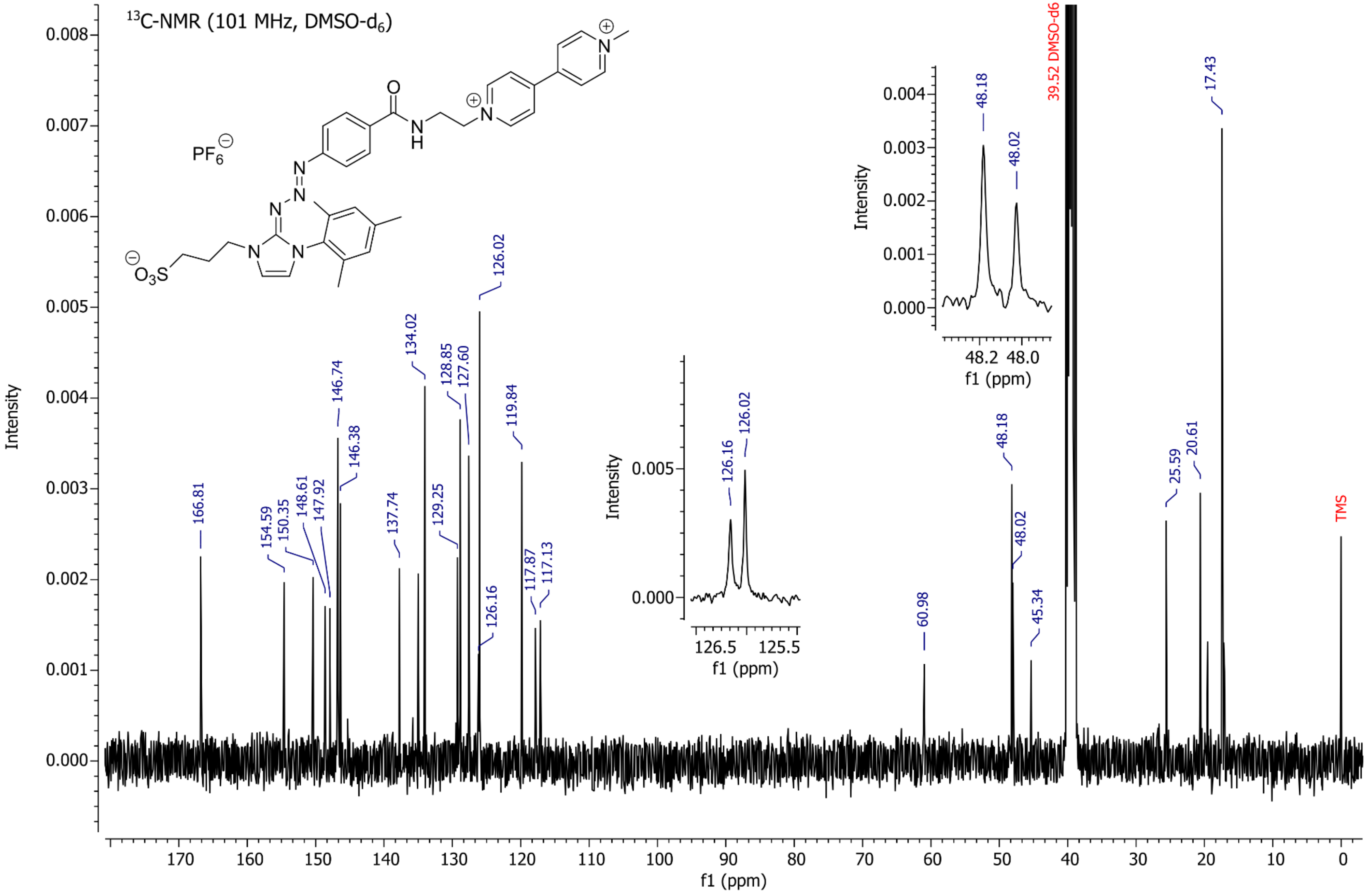

Figure S 72. ^{13}C-NMR spectrum of **10**.

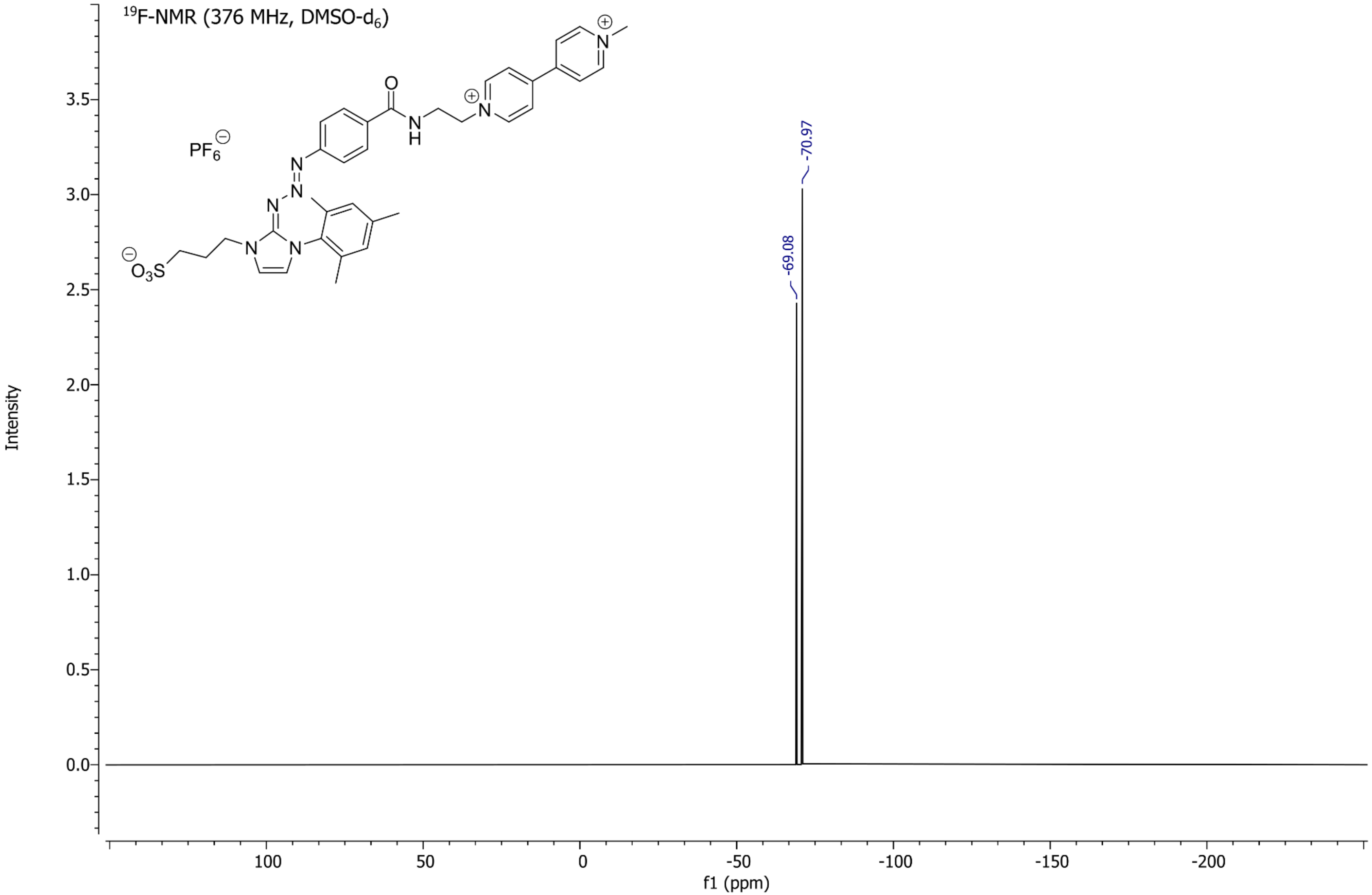

Figure S 73. ^{19}F-NMR spectrum of **10**.

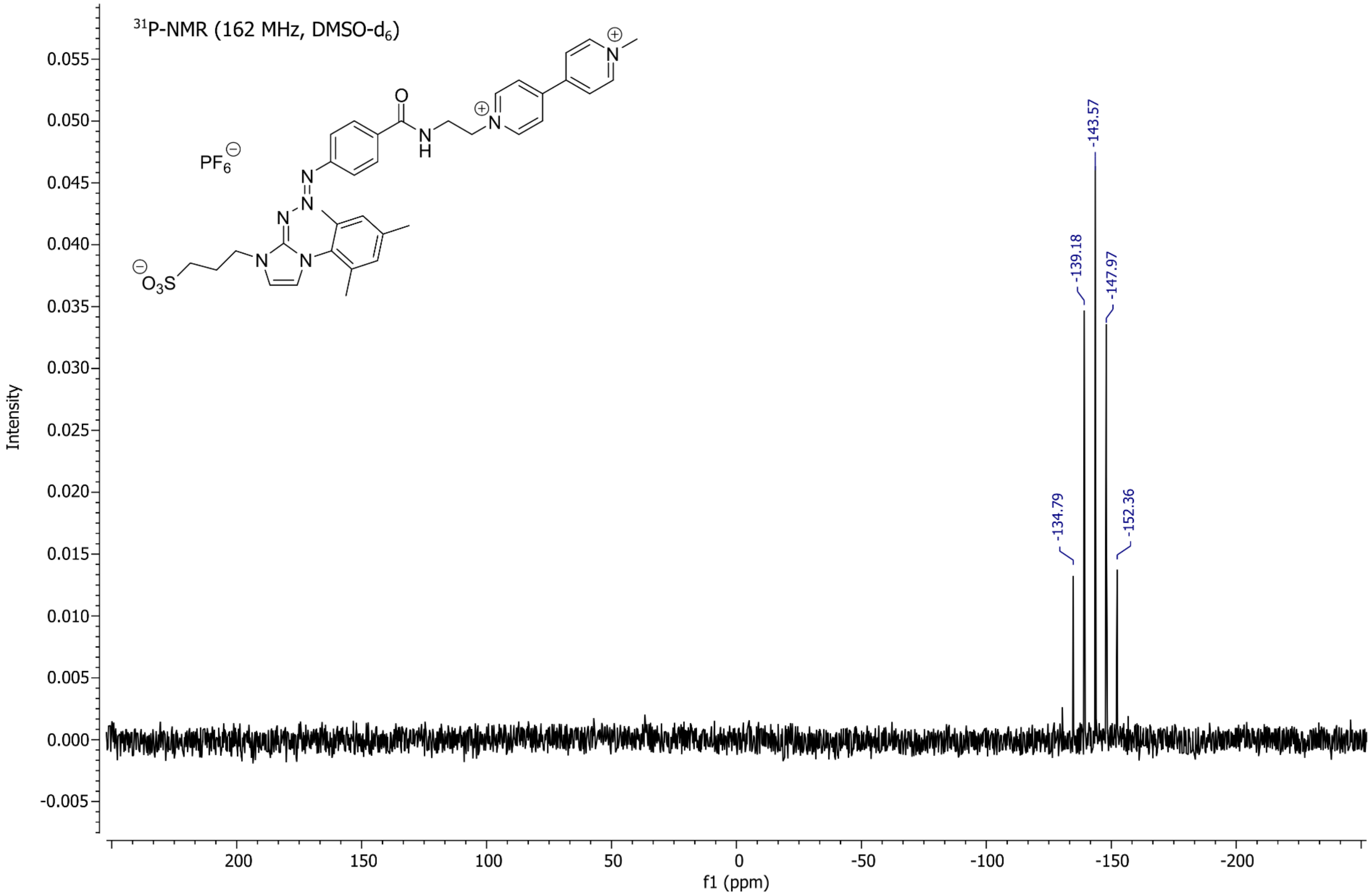

Figure S 74. ^{31}P-NMR spectrum of **10**.

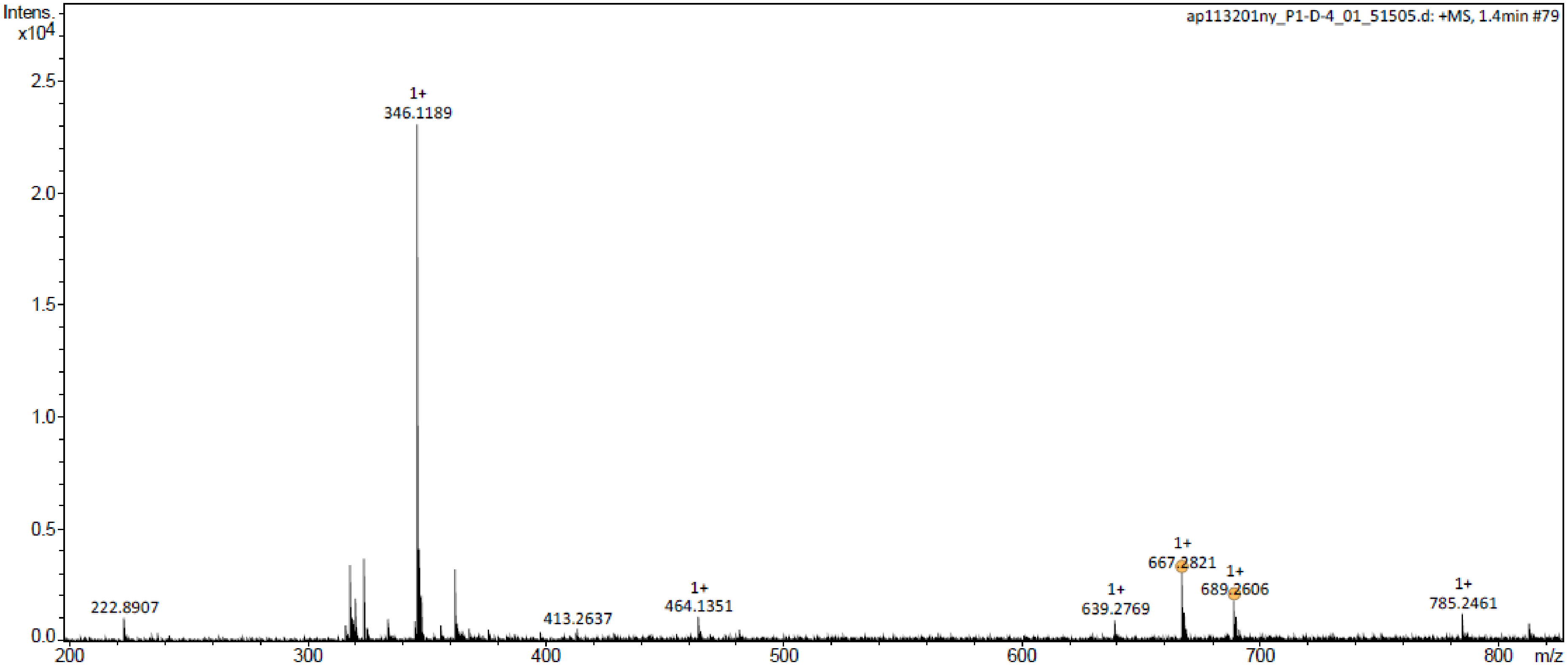

Meas. m/z	#	Ion Formula	m/z	err [ppm]	err [mDa]	mSigma	Mean err [ppm]
667.2821	1	C35H39N8O4S	667.2809	-1.7	-1.1	71.9	-0.7
689.2606	1	C35H38N8NaO4S	689.2629	3.4	2.3	102.6	-0.7

Figure S 75. Positive mode (ESI)HRMS of **10**.

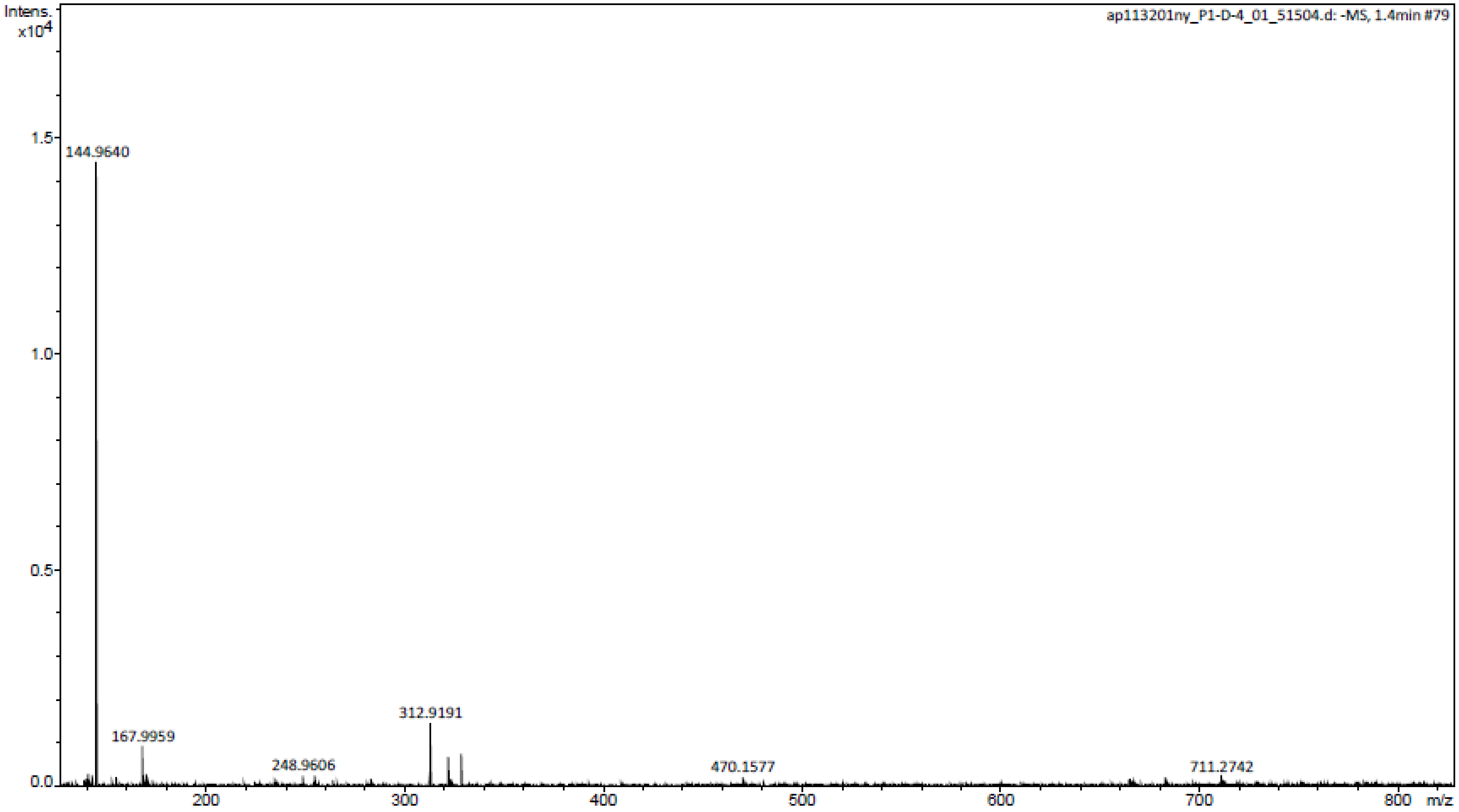

Figure S 76. Negative mode (ESI)HRMS of **10**.

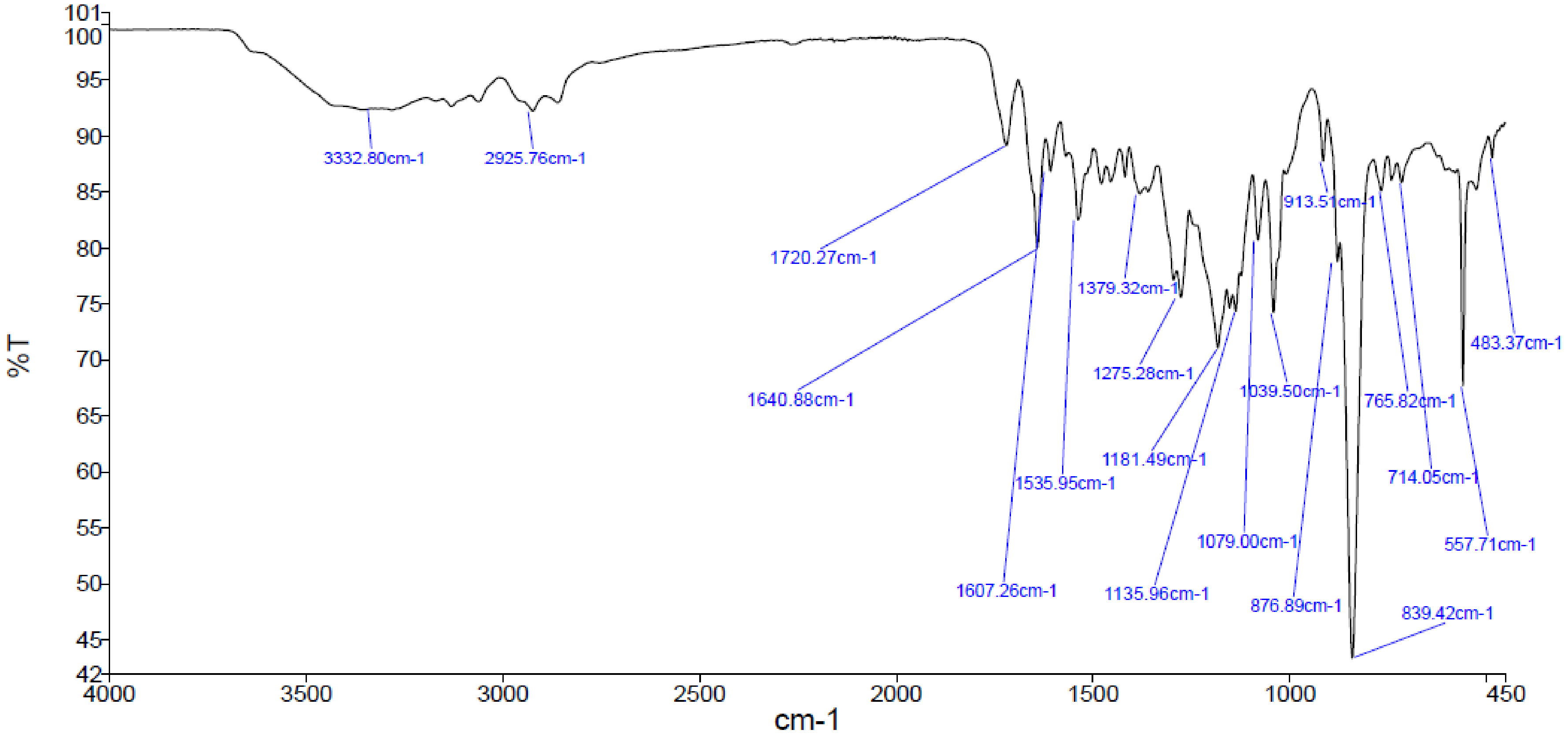

Figure S 77. FT-IR (ATR) spectrum of **10**.

2-azidoethan-1-aminium chloride 11

To a solution of 2-bromoethylamine hydrobromide (1.85 g, 9.0 mmol) in water (25 mL) was added NaN_3 (1.76 g, 27 mmol). The resultant solution was stirred at 75°C for 18 h. The pH of the reaction solution was then adjusted to 14 via the addition of NaOH, whereafter the 2-azidoethan-1-amine product was extracted into DCM (3 × 30 mL). The organic extraction was then added back into the separating funnel and 1 M HCl (aq) was added until the pH of the new aqueous layer remained acidic. The biphasic system was then drained into a round bottomed flask, and all solvents were removed *in vacuo* to yield 2-azidoethan-1-aminium chloride **11** as an off-white microcrystalline solid (1.01 g, 92%).

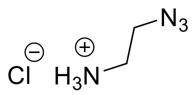

Figure S 78. The structure of **11**.

^{1}H-NMR (400 MHz, D_2O): δ_H 3.73 (t, *J* = 5.60 Hz, 2H), 3.19 (t, *J* = 5.60 Hz, 2H).

^{13}C-NMR (101 MHz, D_2O): δ_c 48.0, 38.7.

FT-IR (ATR) (υmax/cm^{-1}): 2886 (C-H stretch), 2096 (N=N=N stretch, azide).

(ESI)HRMS: Found 87.0666, $C_2H_7N_4^+$ requires 87.0665.

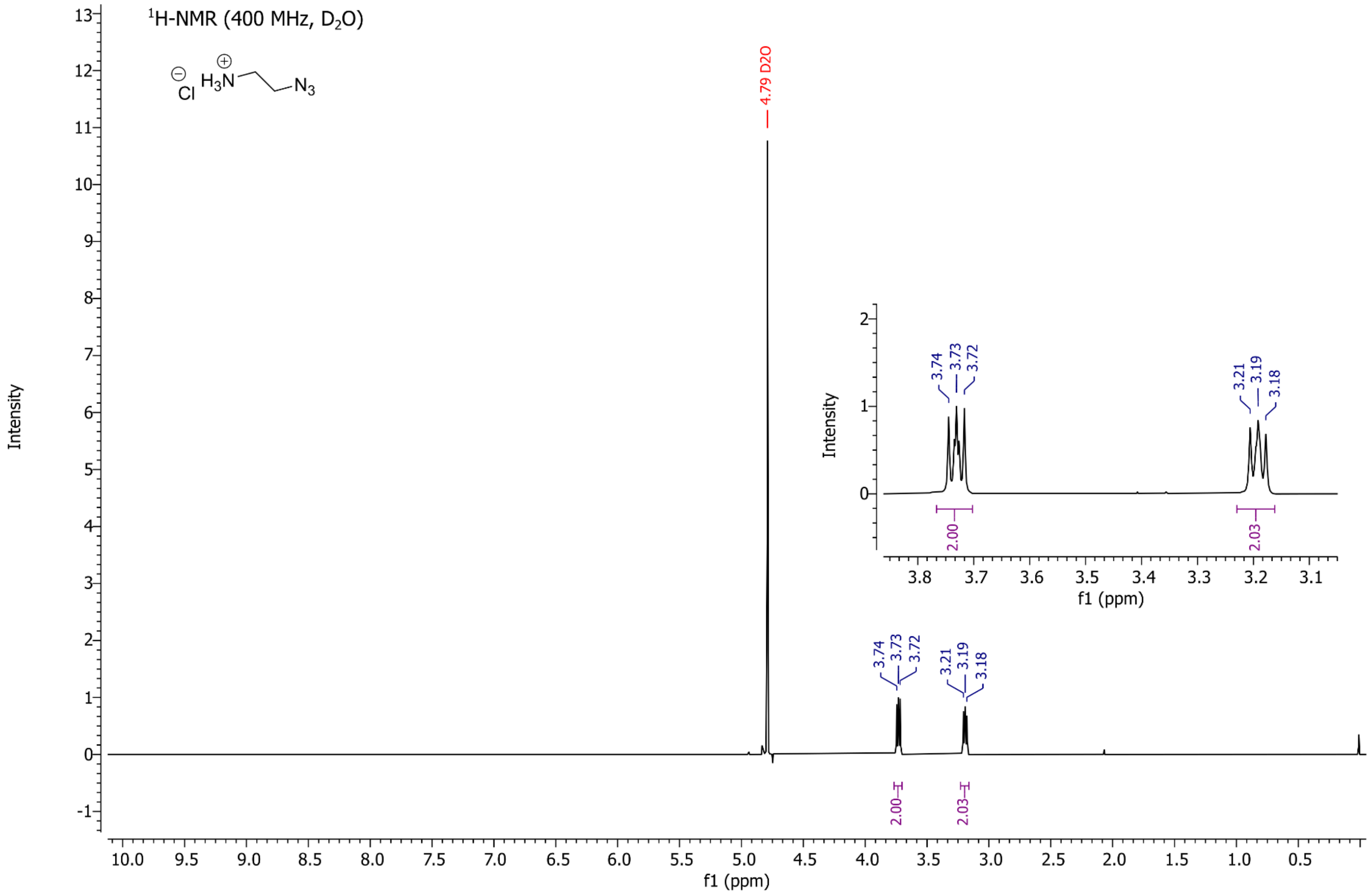

Figure S 79. ^{1}H-NMR spectrum of **11**.

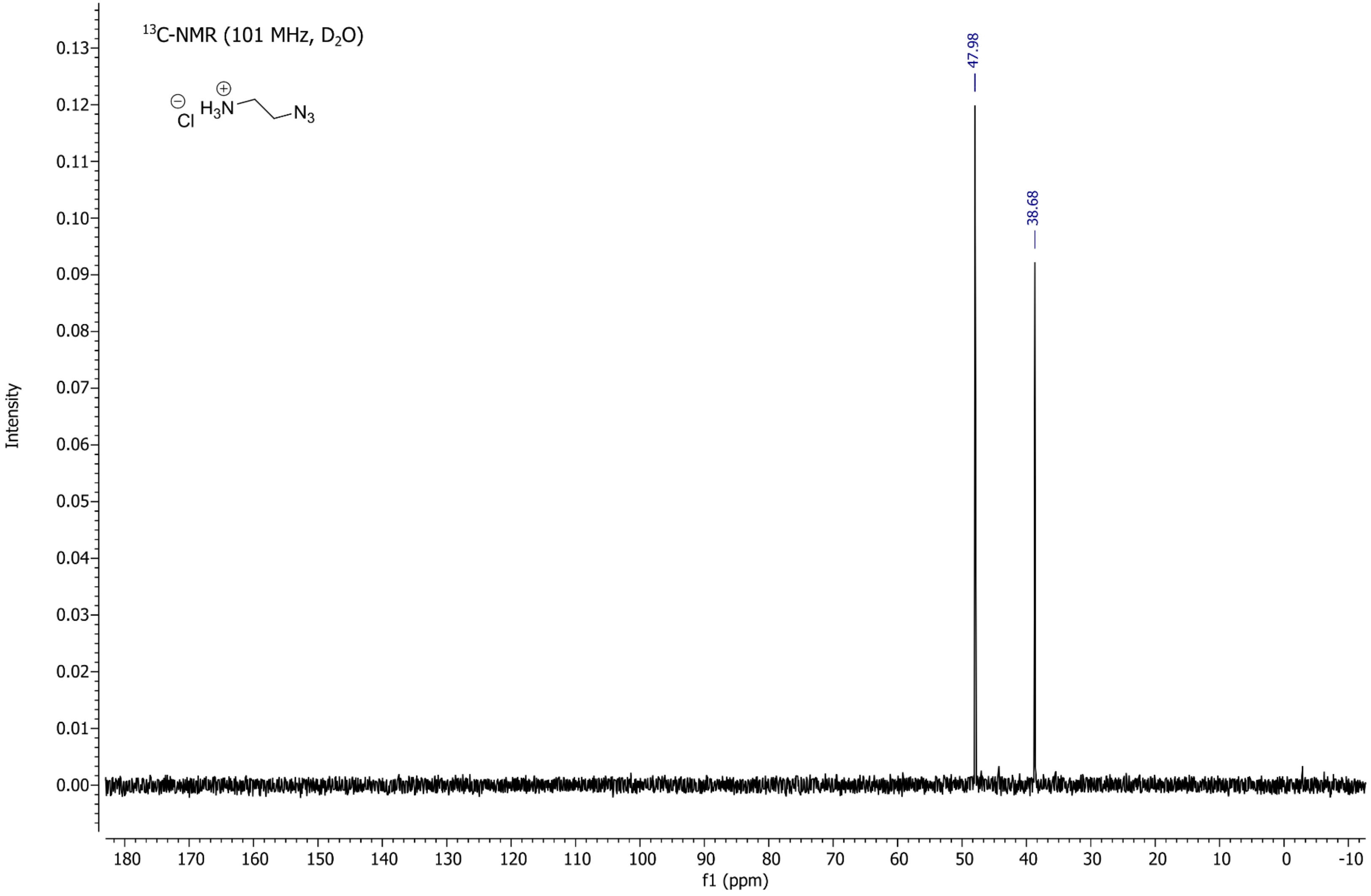

Figure S 80. ^{13}C-NMR spectrum of **11**.

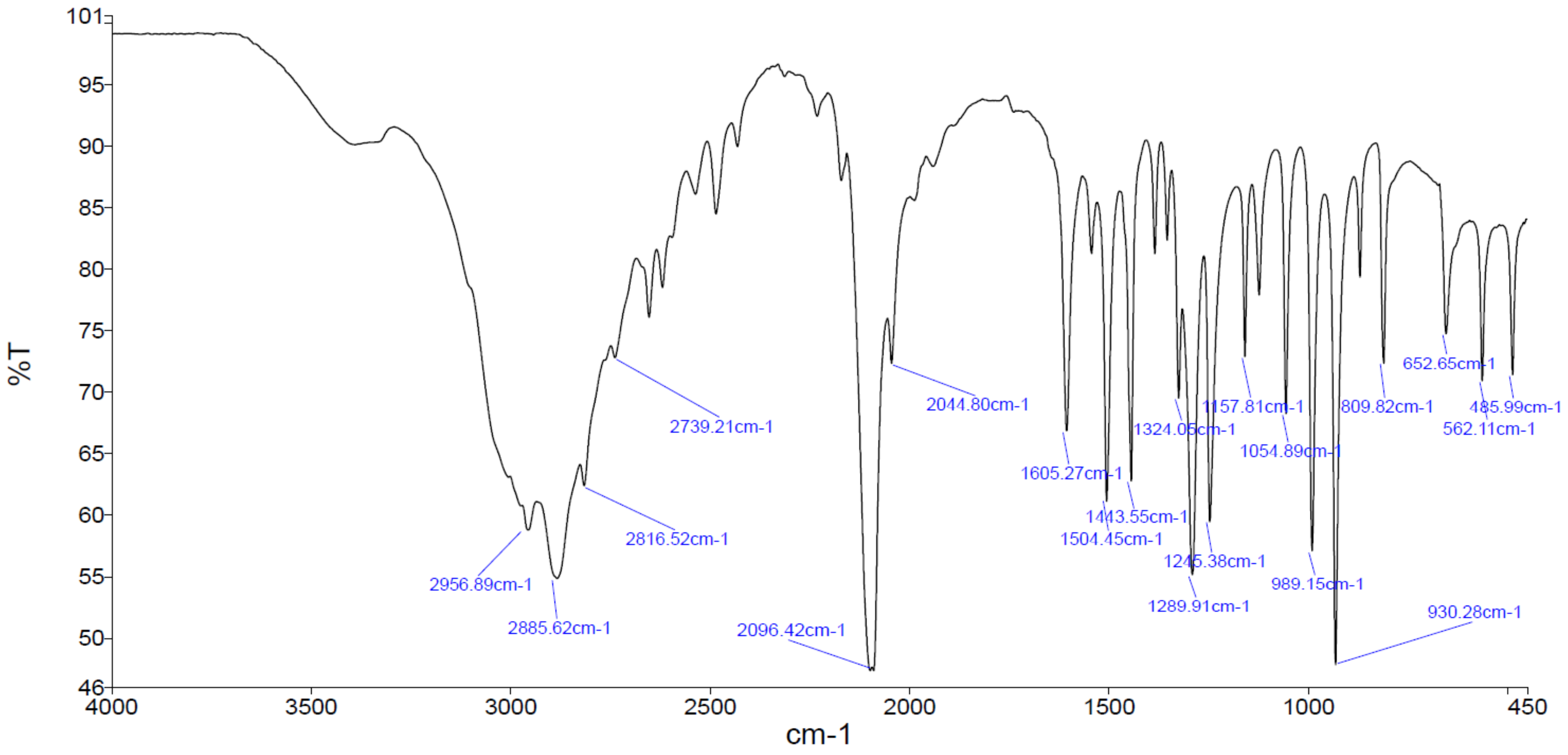

Figure S 81. FT-IR (ATR) spectrum of **11**

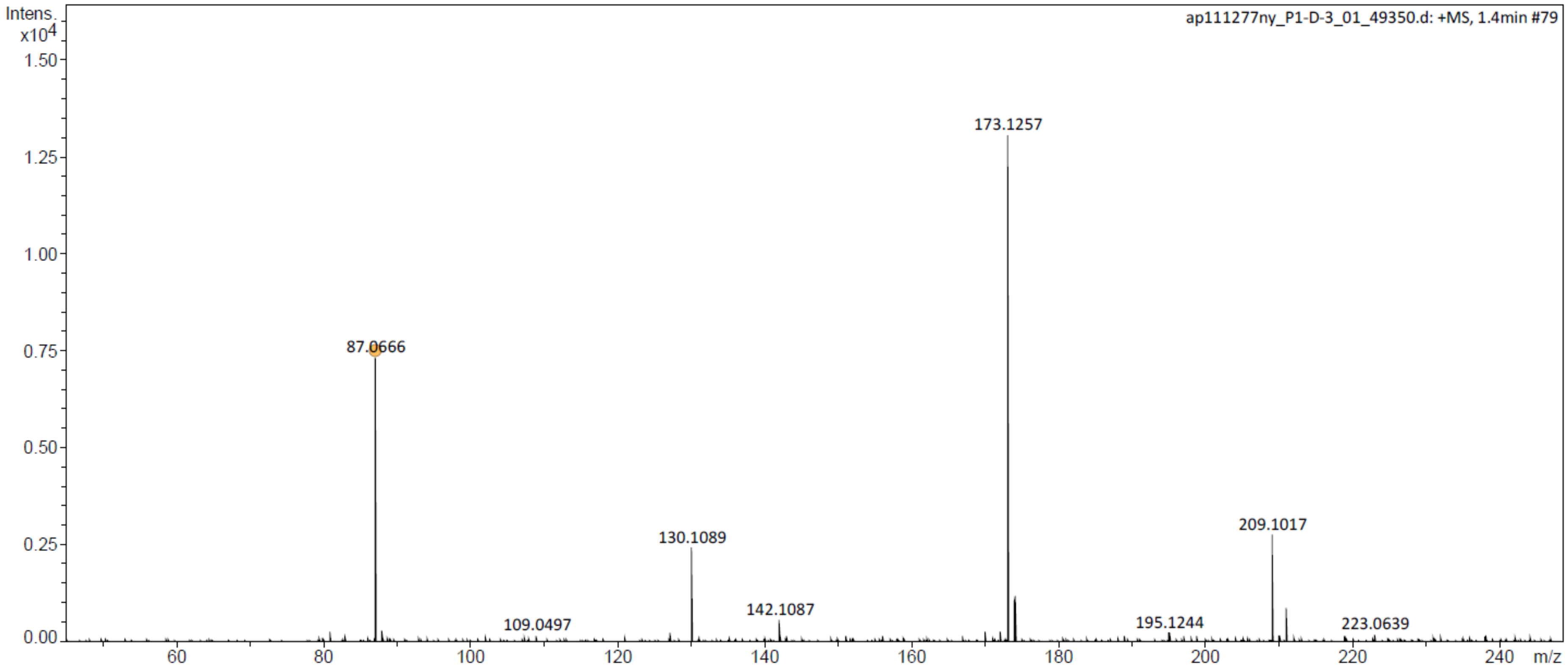

Meas. m/z	#	Ion Formula	m/z	err [ppm]	err [mDa]	mSigma	Mean err [ppm]
87.0666	1	C2H7N4	87.0665	-1.3	-0.1	1.3	-0.6

Figure S 82. (ESI)HRMS of **11**.

Preparation of potassium tert-butoxide treated silica S4

500 mL of dry silica gel was suspended in 20% methanol in DCM. To the resultant suspension was added 0.5 g of potassium tert-butoxide. The resultant suspension was stirred for 10 min, whereafter it was transferred into a glass column. Excess solvent was allowed to drain away, whereafter the potassium tert-butoxide treated silica **S4** was decanted into a beaker and allowed to dry prior to further use.

Potassium 3-((Z)-2-((E)-(4-((2-azidoethyl)carbamoyl)phenyl)triaz-2-en-1-ylidene)-3-mesityl-2,3-dihydro-1H-imidazol-1-yl)propane-1-sulfonate 12

8 (100 mg, 0.18 mmol) was dissolved in DMSO (2 mL), yielding a yellow solution. HCTU (114 mg, 0.27 mmol) was then added, which caused an instantaneous colour change from yellow to orange as the activated ester of **8** formed. The resultant mixture was then stirred in darkness for 1 min.

11 (90 mg, 0.73 mmol) was dissolved in DMSO (1 mL). To this solution was added KO^tBu (82 mg, 0.73 mmol) and the resultant mixture was briefly agitated. The resultant solution was then delivered to the solution containing the activated ester of **8**, causing an instantaneous colour change from orange to yellow. The reaction solution was then stirred for 3.5 h at rt in darkness, whereafter tetrabutylammonium bromide (60 mg, 0.19 mmol) was added. The resultant solution was then diluted with DCM (30 mL) and transferred to a separating funnel. Water (30 mL) was added to the separating funnel, forming the top layer, and 0.55 mL of 1 M HCl was then added. The biphasic mixture was then shaken and allowed to re-separate. The organic layer was then taken and dried over $MgSO_4$.

A 1 M aqueous solution of potassium hexafluorophosphate was prepared, and 0.18 mL of this solution was delivered to the dried organic solution. The resultant mixture was then concentrated under a stream of nitrogen. The resultant residue was triturated with ethyl acetate until insoluble material appeared, whereafter an equal volume of hexane was added. The crude product was then isolated by filtration and was further purified via flash column chromatography using a mobile phase of 20% methanol in DCM and a stationary phase of potassium tert-butoxide treated silica **S4**. This yielded potassium **12** as an intense yellow solid (50 mg, 48%).

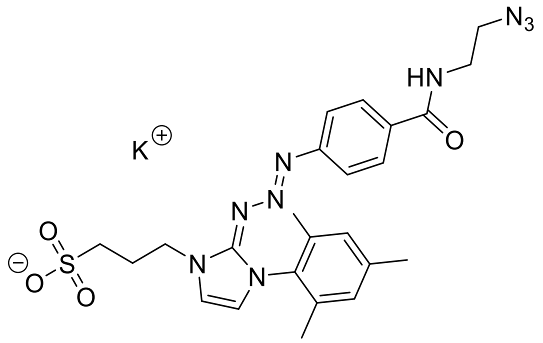

Figure S 83. The structure of **12**.

^{1}H-NMR (400 MHz, Methanol-d_4): δ_H 7.60-7.53 (m, 2H), 7.29 (d, *J* = 2.44 Hz, 1H), 7.05 (s, 2H), 6.77 (d, *J* = 2.44 Hz, 1H), 6.65-6.57 (m, 2H), 4.29 (t, *J* = 7.07 Hz, 2H), 3.59-3.51 (t, *J* = 5.61 Hz, 2H), 3.51-3.44 (m, 2H), 2.91 (t, *J* = 7.80 Hz, 2H), 2.40 (s, 3H), 2.39-2.31 (m, 2H), 2.03 (s, 6H).

^{13}C-NMR (101 MHz, Methanol-d_4): δ_c 170.3, 155.2, 152.0, 140.1, 136.8, 135.5, 131.5, 130.4, 128.8, 121.4, 119.1, 118.5, 51.5, 49.1, 45.8, 40.6, 26.4, 21.2, 18.0.

FT-IR (ATR) (υmax/cm^{-1}): 3356 (C-H stretch), 2100 (N=N=N stretch, azide), 1635 (C=O stretch), 1534, 1159 (S=O stretch).

(ESI)HRMS: Found 538.1989, $C_{24}H_{28}N_9O_4S^-$ requires Found 538.1990.

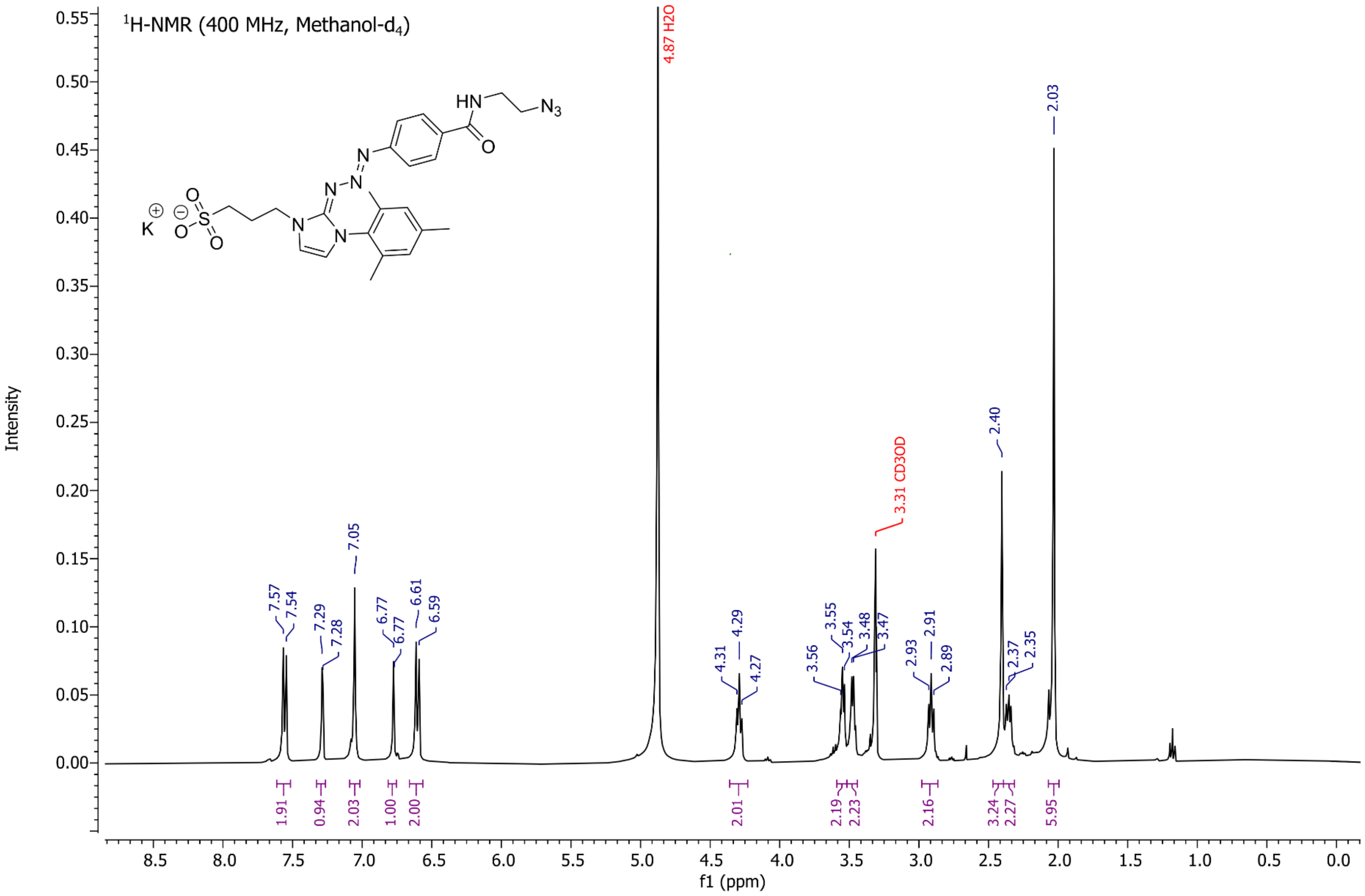

Figure S 84. ^{1}H-NMR spectrum of **12**.

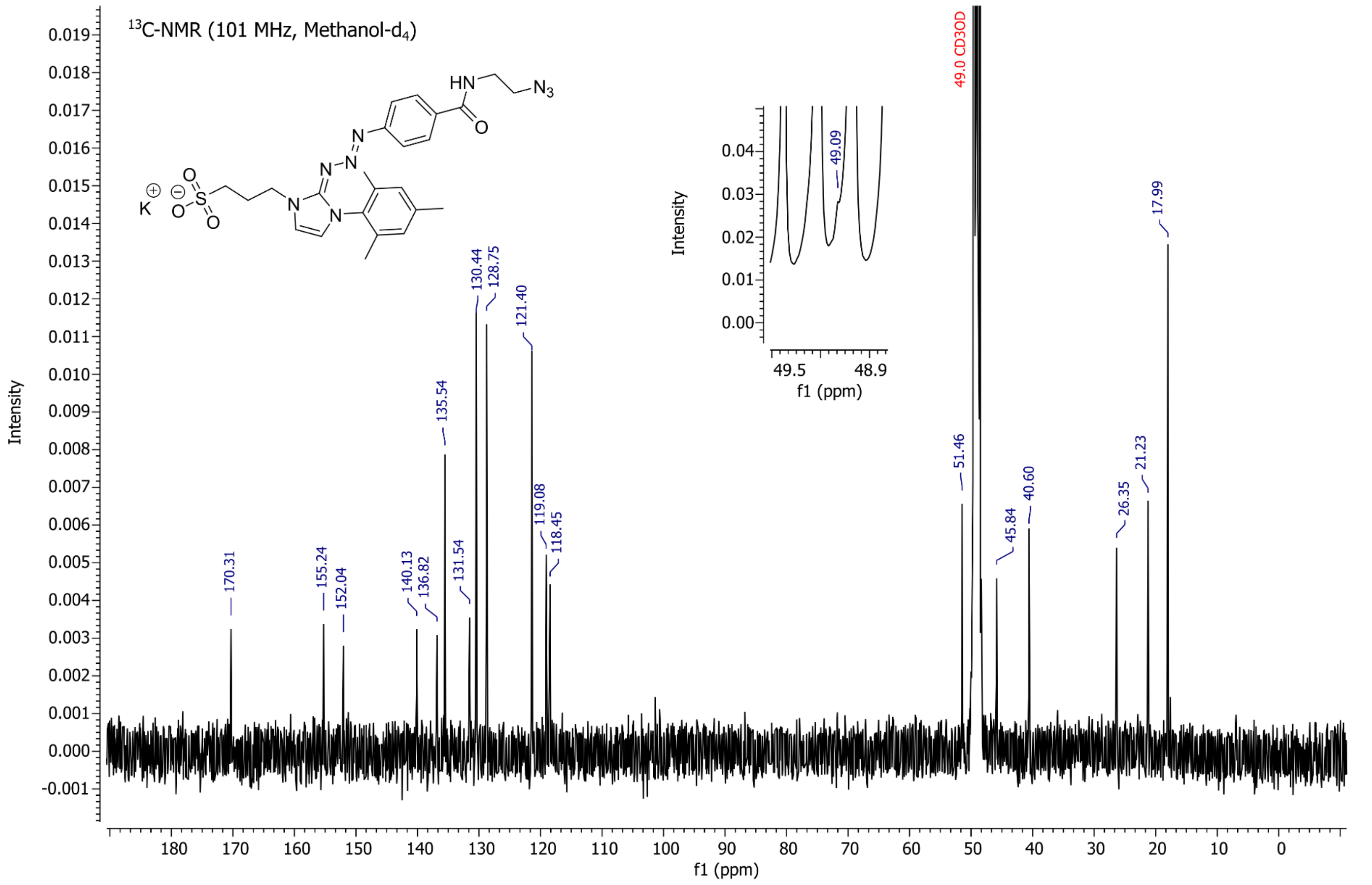

Figure S 85. ^{13}C-NMR spectrum of **12**.

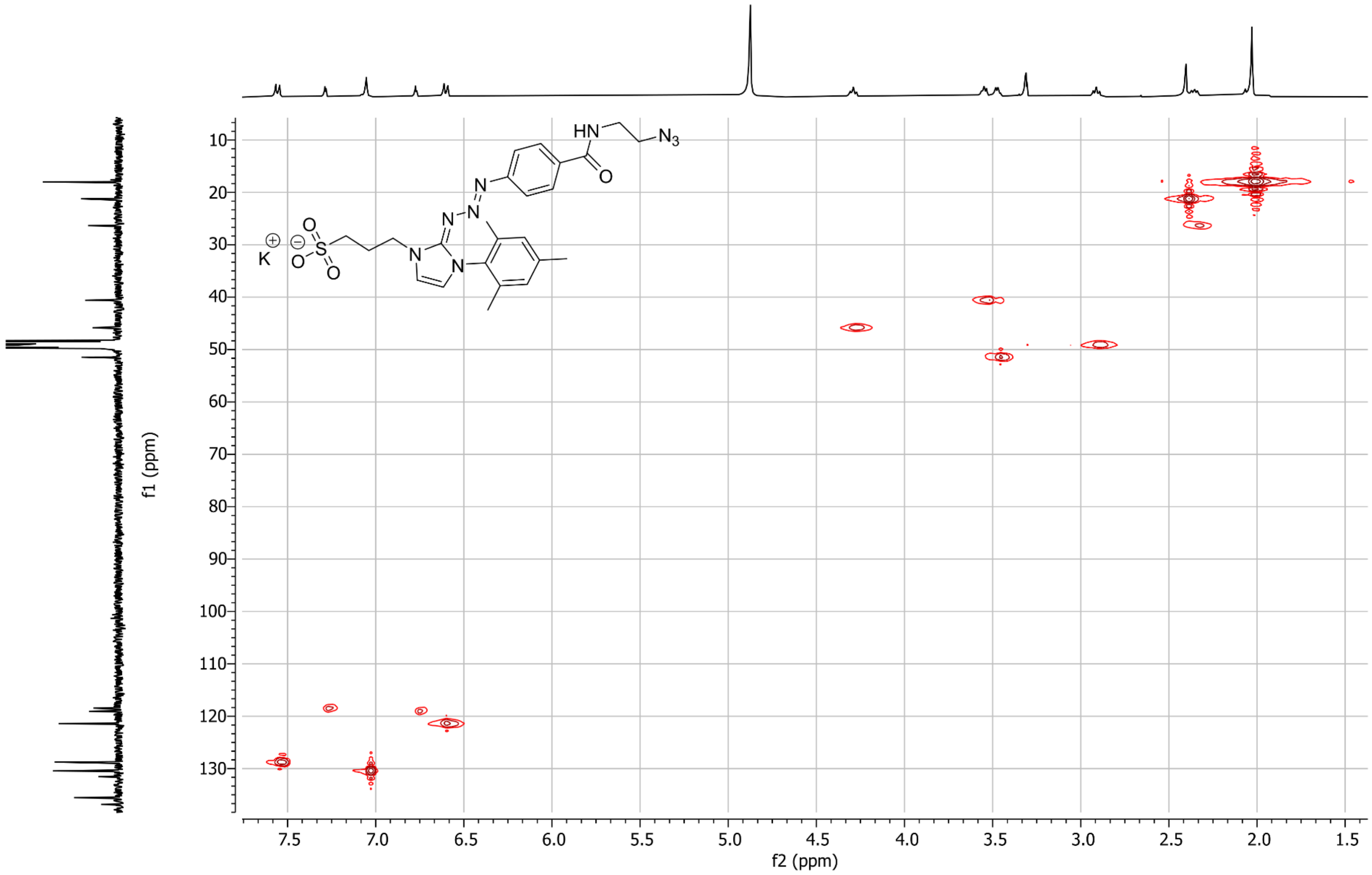

Figure S 86. ^{13}C-HMQC NMR spectrum of **12**.

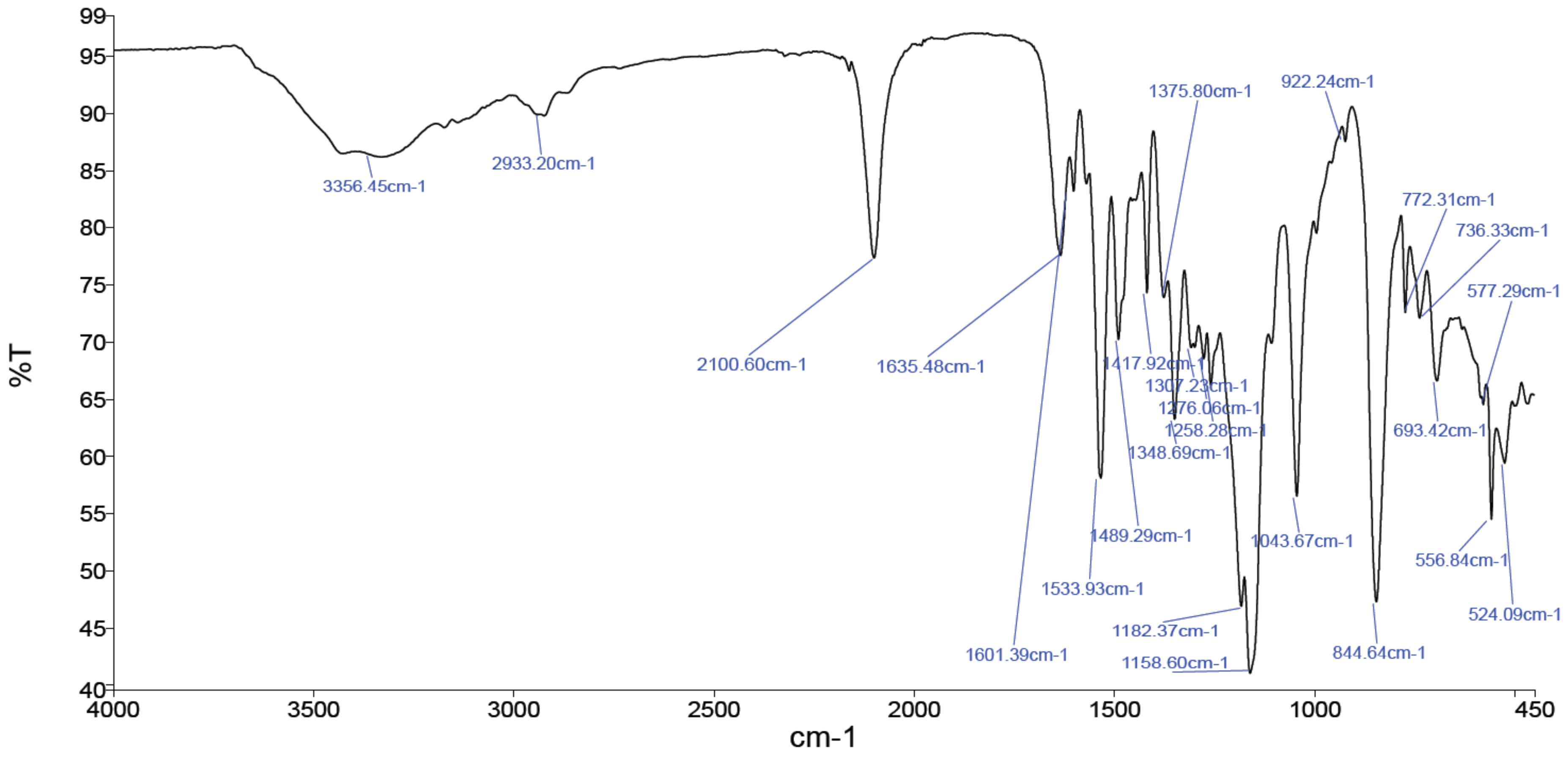

Figure S 87. FT-IR (ATR) spectrum of **12**.

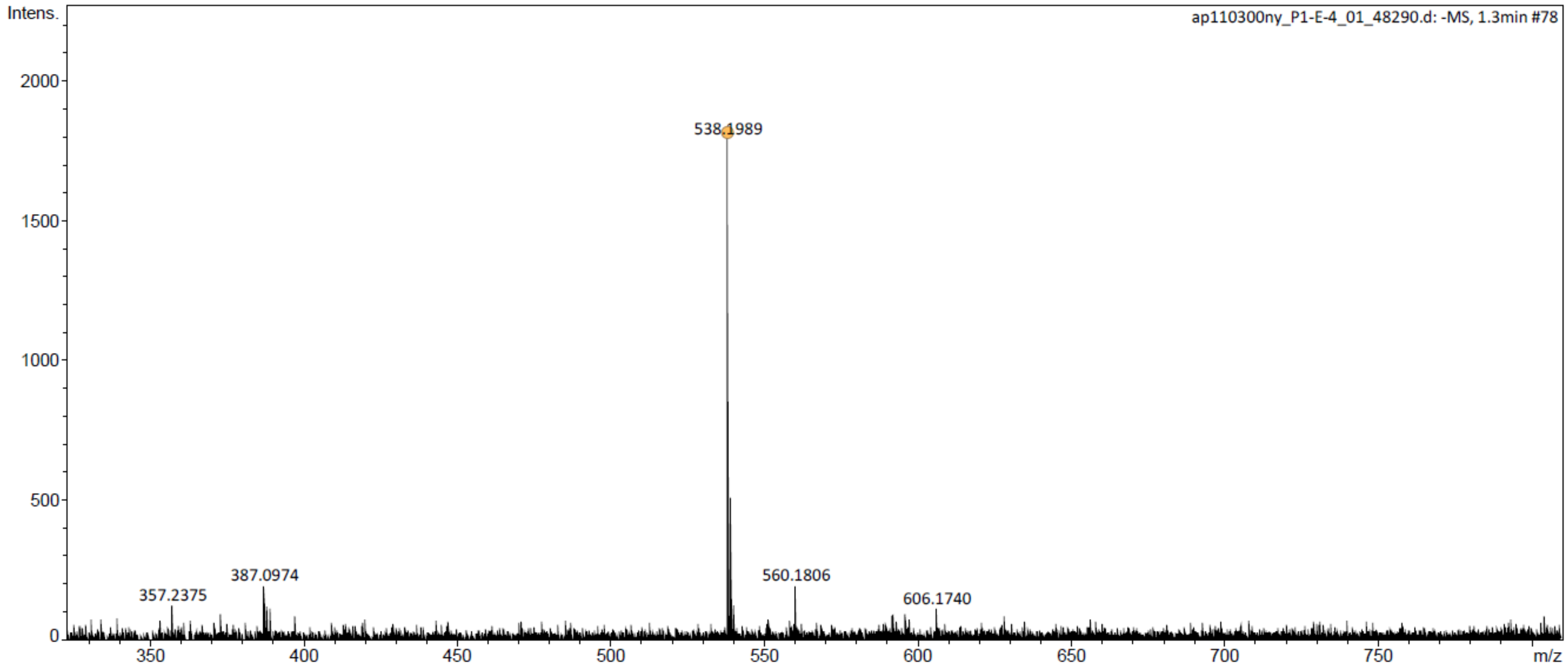

Meas. m/z	#	Ion Formula	m/z	err [ppm]	err [mDa]	mSigma	Mean err [ppm]
538.1989	1	C24H28N9O4S	538.1990	0.2	0.1	20.5	-2.0

Figure S 88. Negative mode (ESI)HRMS of **12**.

1-(but-3-yn-1-yl)-[4,4'-bipyridin]-1-ium hexafluorophosphate S5

To a solution of 3-Butyn-1-ol (2 g, 28.5 mmol) in DCM (50 mL) at 0°C was added DMAP (34 mg, 0.28 mmol). Tosyl chloride (6.52 g, 34.2 mmol) was then added, followed by triethylamine (3.48 g, 34.4 mmol, 4.8 mL) was then added and the resultant reaction mixture was stirred for 5 hours at rt under N_2. A precipitate formed, which was removed via Bucher filtration and rinsed washed with additional DCM (20 mL). The eluates were combined, and were washed sequentially with water (2 x 50 mL), saturated $NaHCO_3$(aq) (50 mL) and brine (50 mL). The washed solution was then dried over $MgSO_4$ and concentrated *in vacuo* to yield a crude sample of the tosylate derivative of 3-butyn-1-ol, which was then used immediately without further purification.

The crude tosylate was dissolved in the minimum volume of MeCN, and was then delivered dropwise to a stirred solution of 4,4'-dipyridyl (12 g, 76.8 mmol) in MeCN (60 mL). The resultant solution was then heated at reflux overnight under N_2. The reaction mixture was then concentrated in vacuo, and diethyl ether (150 mL) was added. The crude product mixture was sonicated vigorously, and the resultant milky coloured diethyl ether solution was decanted to leave a brown residue in the flask. Ethyl acetate (100 mL) was then added to the vessel that contained the brown residue, and the vessel was vigorously shaken. The ethyl acetate was then decanted, again leaving a brown residue in the flask. The brown residue was then dissolved in the minimum volume of water and the resultant solution was transferred to a separating funnel. The aqueous product solution was washed with DCM (2 x 30 mL) and was then decanted into a round bottom flask and stirred.

A saturated solution of NH_4PF_6 (aq) was added dropwise to the stirred aqueous product solution until no more precipitate formed. The resulting mixture was then sonicated (to break apart any clumps) and the precipitate was collected via Buchner filtration. The precipitate was then recrystalised from hot water, isolated by Bucher filtration and allowed to dry. This yielded 1-(but-3-yn-1-yl)-[4,4'-bipyridin]-1-ium hexafluorophosphate **S5** as a pale tan powder (4.58 g, 45%).

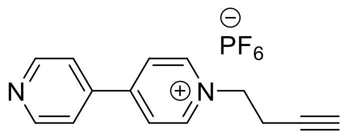

Figure S 89. The structure of **S5**.

^{1}H-NMR (400 MHz, DMSO-d$_6$): δ_H 9.28-9.22 (m, 2H), 8.90-8.85 (m, 2H), 8.72-8.75 (m, 2H), 8.08-8.02 (m, 2H), 4.80 (t, J = 6.60 Hz, 2H), 3.08 (t, J = 2.53 Hz, 2H), 3.02 (tt, J = 6.60, 2.53 Hz, 2H).

^{13}C-NMR (101 MHz, DMSO-d$_6$): δ_C 152.8, 150.9, 145.6, 140.9, 125.2, 122.0, 79.2, 75.2, 58.3, 20.4.

^{19}F-NMR (376 MHz, DMSO-d$_6$): δ_F -69.9 (d, J_{P-H} = 711 Hz).

FT-IR (ATR) (υmax/cm^{-1}): 3294 (C-H stretch, alkyne), 1643 (C=C stretch, aromatic), 826 (C-H bending, aromatic).

(ESI)HRMS: Positive mode found 209.1070, $C_{14}H_{13}N_2^+$ requires 209.1073; negative mode found 144.9646, PF_6^- requires 144.9647.

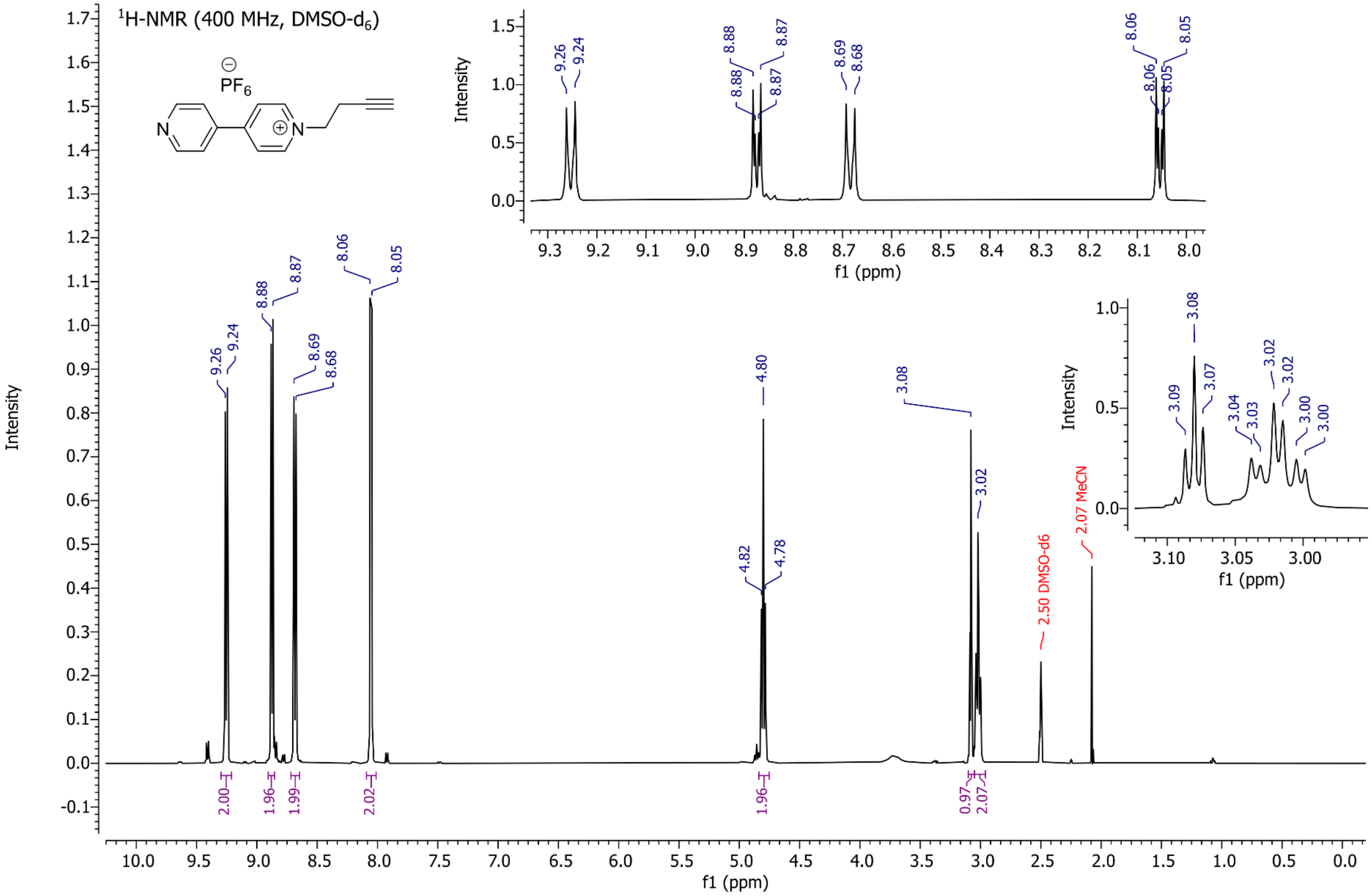

Figure S 90. ^{1}H-NMR spectrum of **S5**.

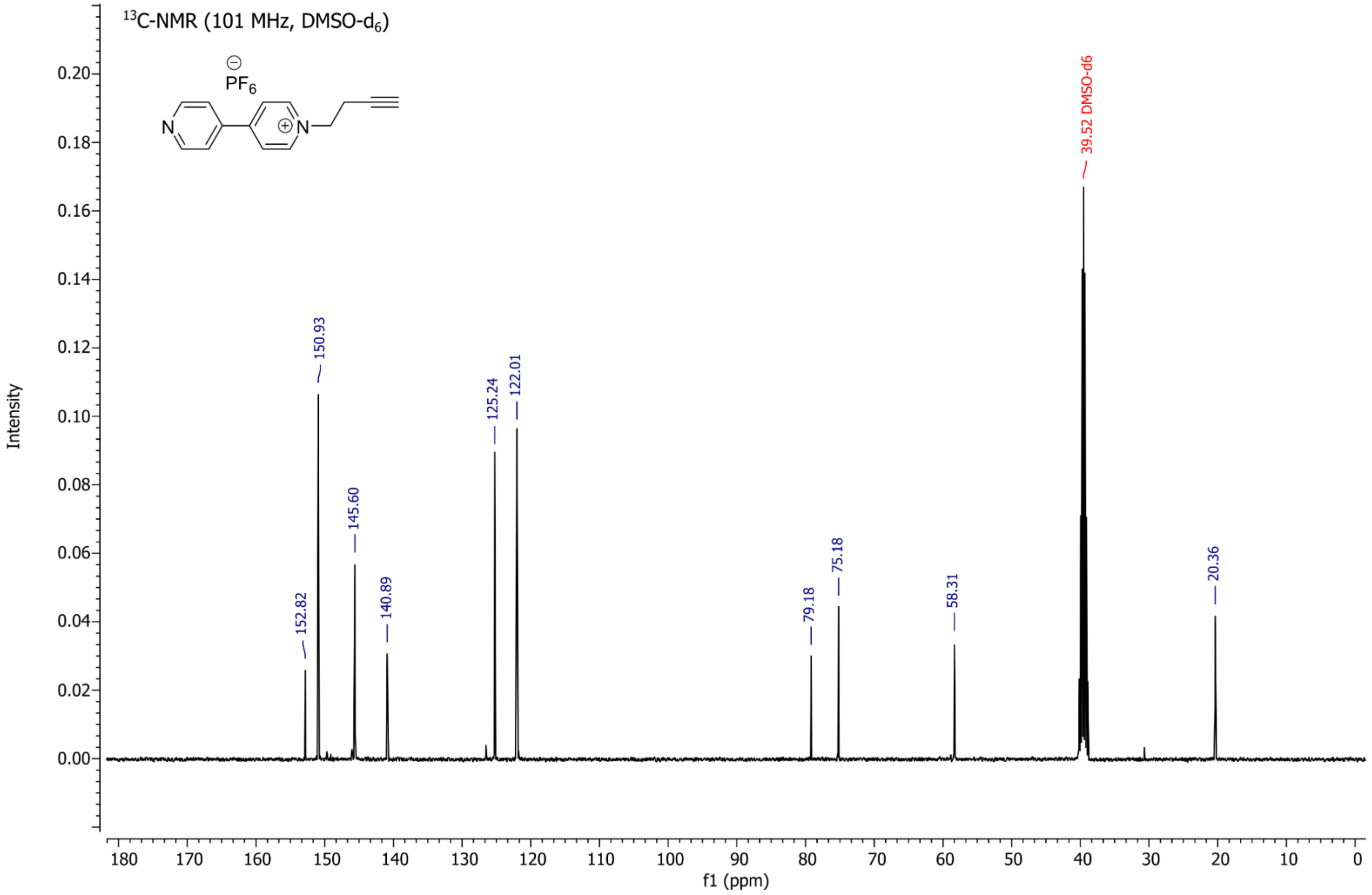

Figure S 91. ^{13}C-NMR spectrum of **S5**.

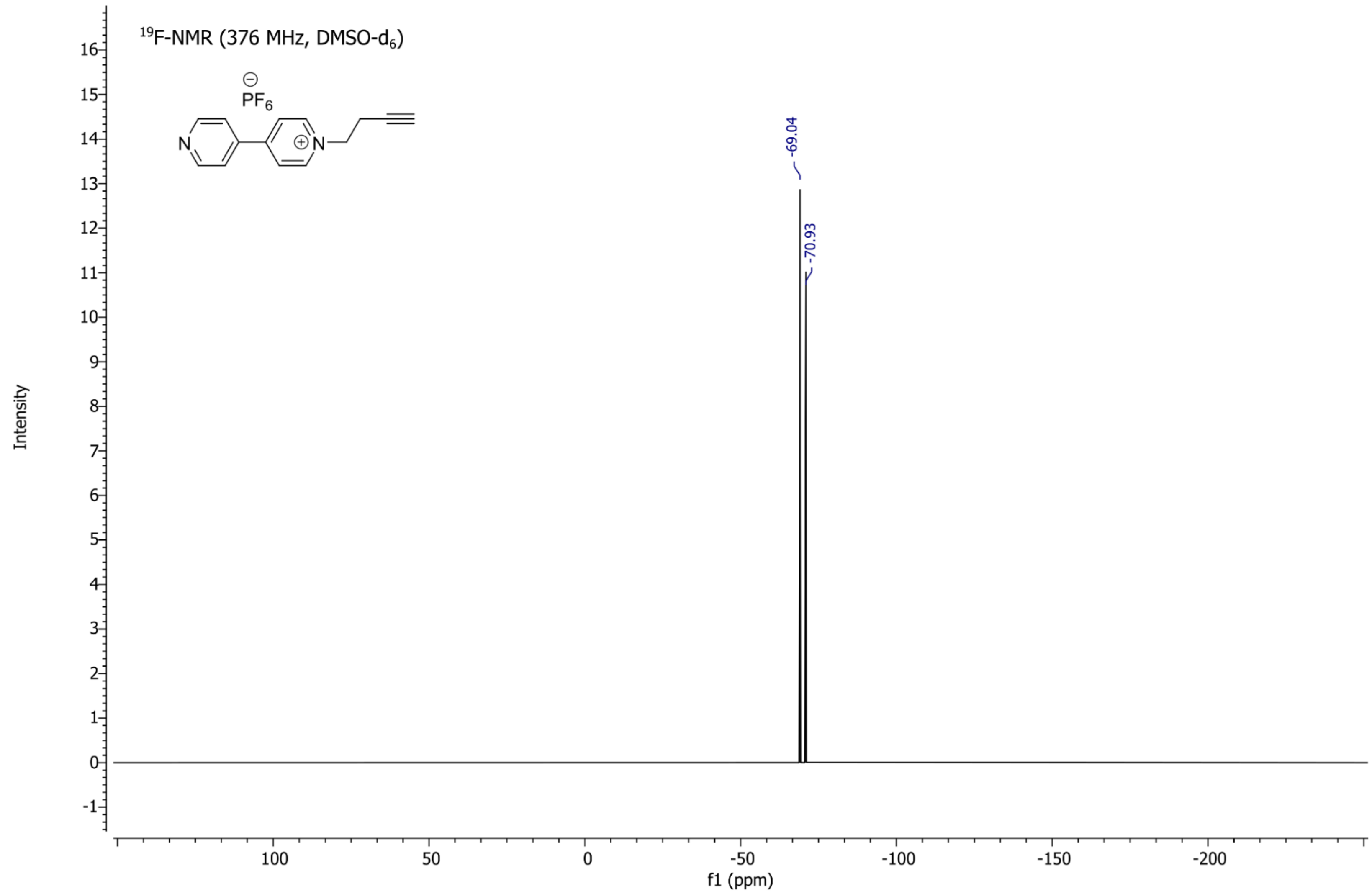

Figure S 92. ^{19}F-NMR spectrum of **S5**.

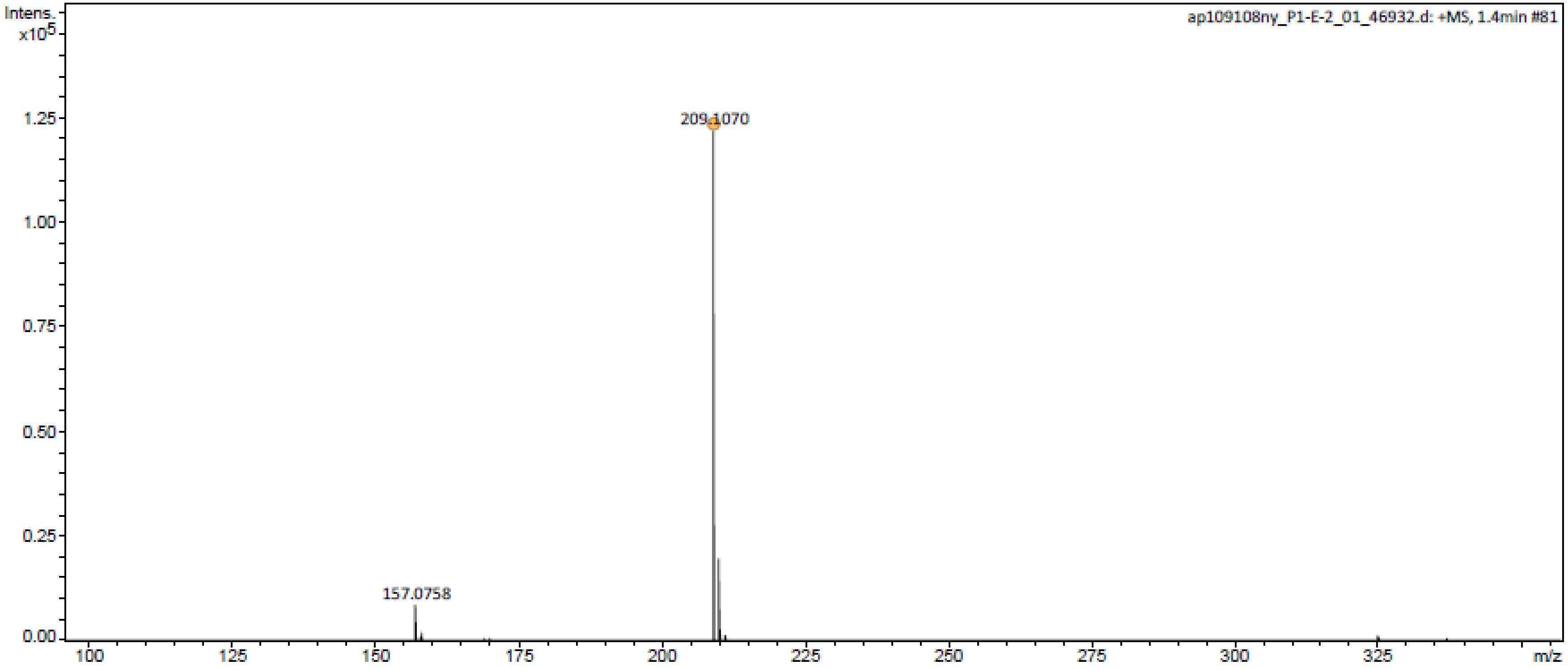

Meas. m/z	#	Ion Formula	m/z	err [ppm]	err [mDa]	mSigma	Mean err [ppm]
209.1070	1	C14H13N2	209.1073	1.5	0.3	0.8	2.3

Figure S 93. Positive mode (ESI)HRMS of **S5**.

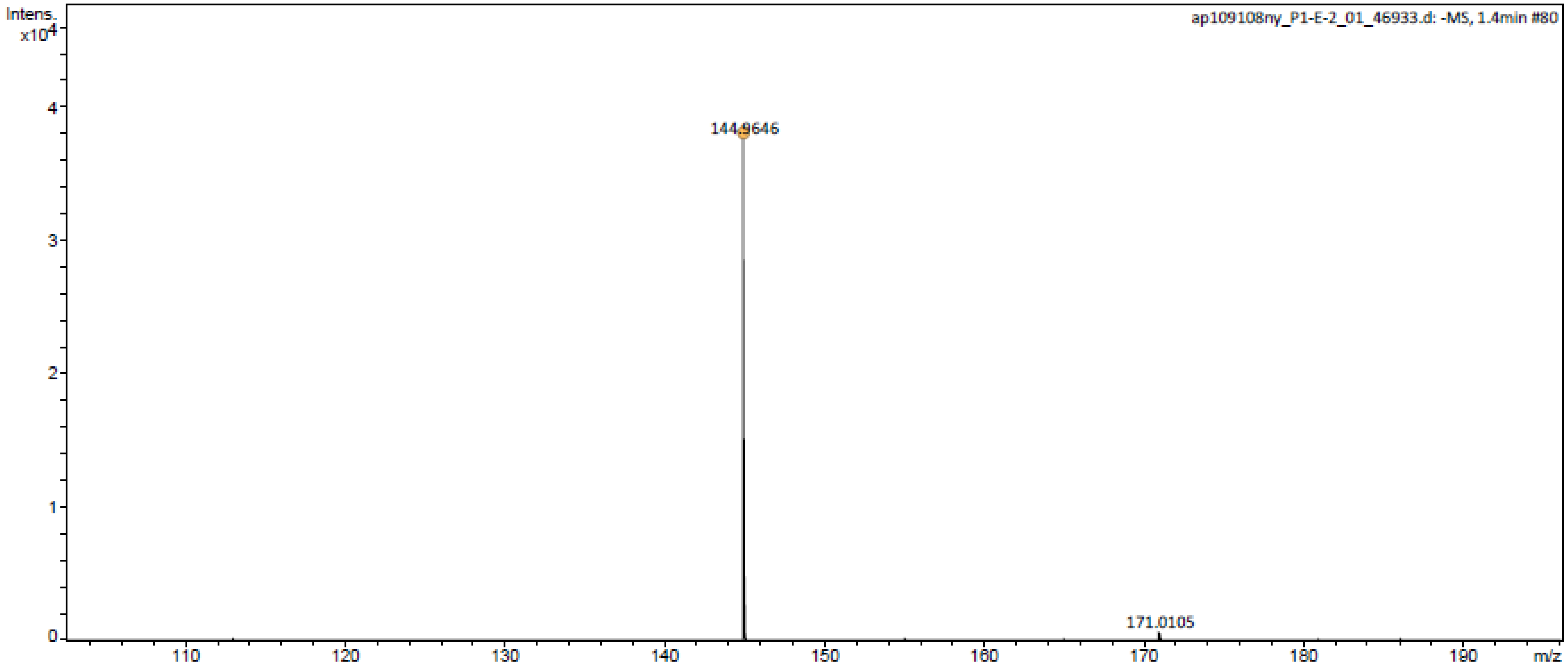

Meas. m/z	#	Ion Formula	m/z	err [ppm]	err [mDa]	mSigma	Mean err [ppm]
144.9646	1	F6P	144.9647	0.9	0.1	n.a.	0.9

Figure S 94. Negative mode (ESI)HRMS of **S5**.

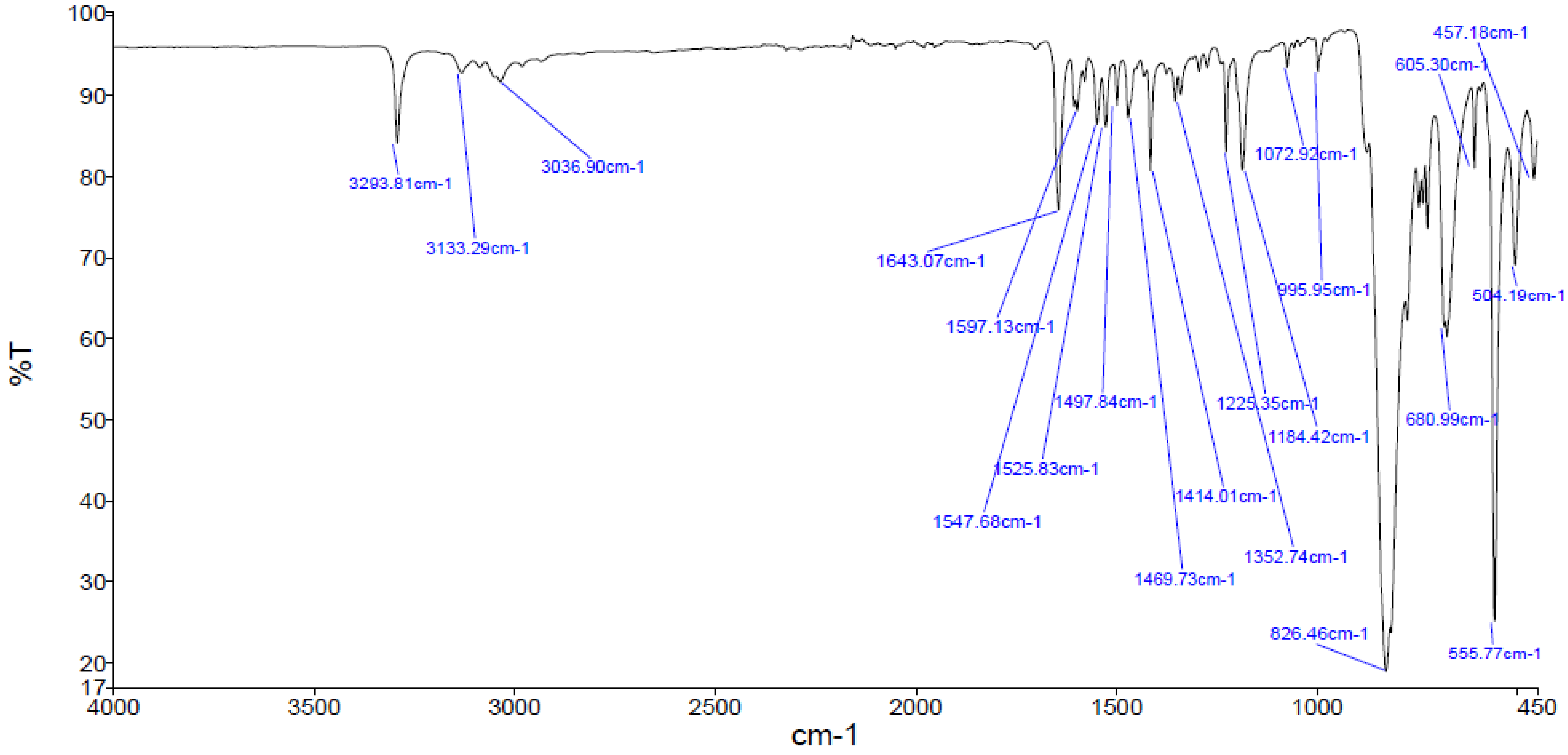

Figure S 95. FT-IR (ATR) spectrum of **S5**.

1-(but-3-yn-1-yl)-1'-methyl-[4,4'-bipyridine]-1,1'-diium hexafluorophosphate S6

To **S5** (0.50 g, 0.97 mmol) dissolved in the minimum volume of MeCN was added iodomethane (0.213 g, 1.5 mmol, 0.1 mL). The resultant solution was then stirred for 3 h at 50°C, whereafter additional MeCN was added if the reaction mixture ceased to be freely stirring (due to precipitate formation). The reaction mixture was then stirred at 50°C overnight. The precipitated orange product was thereafter isolated via Buchner filtration and then dissolved in the minimum volume of water with stirring. A saturated solution of NH_4PF_6 (aq) was then added dropwise to the stirred aqueous product solution until no more precipitate formed. The resulting mixture was then sonicated (to break apart any clumps) and the precipitate was collected via Buchner filtration. The precipitate was then re-dissolved in the minimum volume of hot water, which was then allowed to cool to yielded 1-(but-3-yn-1-yl)-1'-methyl-[4,4'-bipyridine]-1,1'-diium hexafluorophosphate **S6** as pale tan microcrystals which were collected via filtration and allowed to dry (0.26 g, 52%).

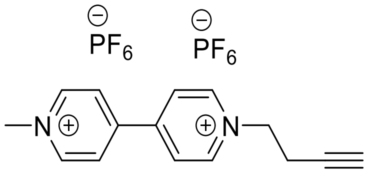

Figure S 96. The structure of **S6**.

^{1}H-NMR (400 MHz, DMSO-d$_6$): δ_H 9.43-9.36 (m, 2H), 9.32-9.25 (m, 2H), 8.86-8.80 (m, 2H), 8.78-8.72 (m, 2H), 4.84 (t, *J* = 6.65 Hz, 2H), 4.43 (s, 3H), 3.13 (t, *J* = 2.50 Hz, 2H), 3.02 (td, *J* = 6.65, 2.50 Hz, 2H).

^{13}C-NMR (101 MHz, DMSO-d$_6$): δ_C 149.2, 148.1, 146.6, 146.1, 126.4, 126.2, 79.1, 75.3, 58.8, 48.1, 20.4.

^{19}F-NMR (376 MHz, DMSO-d$_6$): δ_F -70.0 (d, J_{P-F} = 711 Hz).

FT-IR (ATR) (υmax/cm^{-1}): 3300 (C-H stretch, alkyne), 1643 (C=C stretch, aromatic), 813 (P-F stretch), 551 (P-F bending).

(ESI)HRMS: Positive mode found 112.0653, $C_{15}H_{16}N_2^{2+}$ requires 112.0651; positive mode found 223.1225, $C_{15}H_{15}N_2^+$ requires 223.1230; negative mode found 144.9641, PF_6^- requires 144.9647.

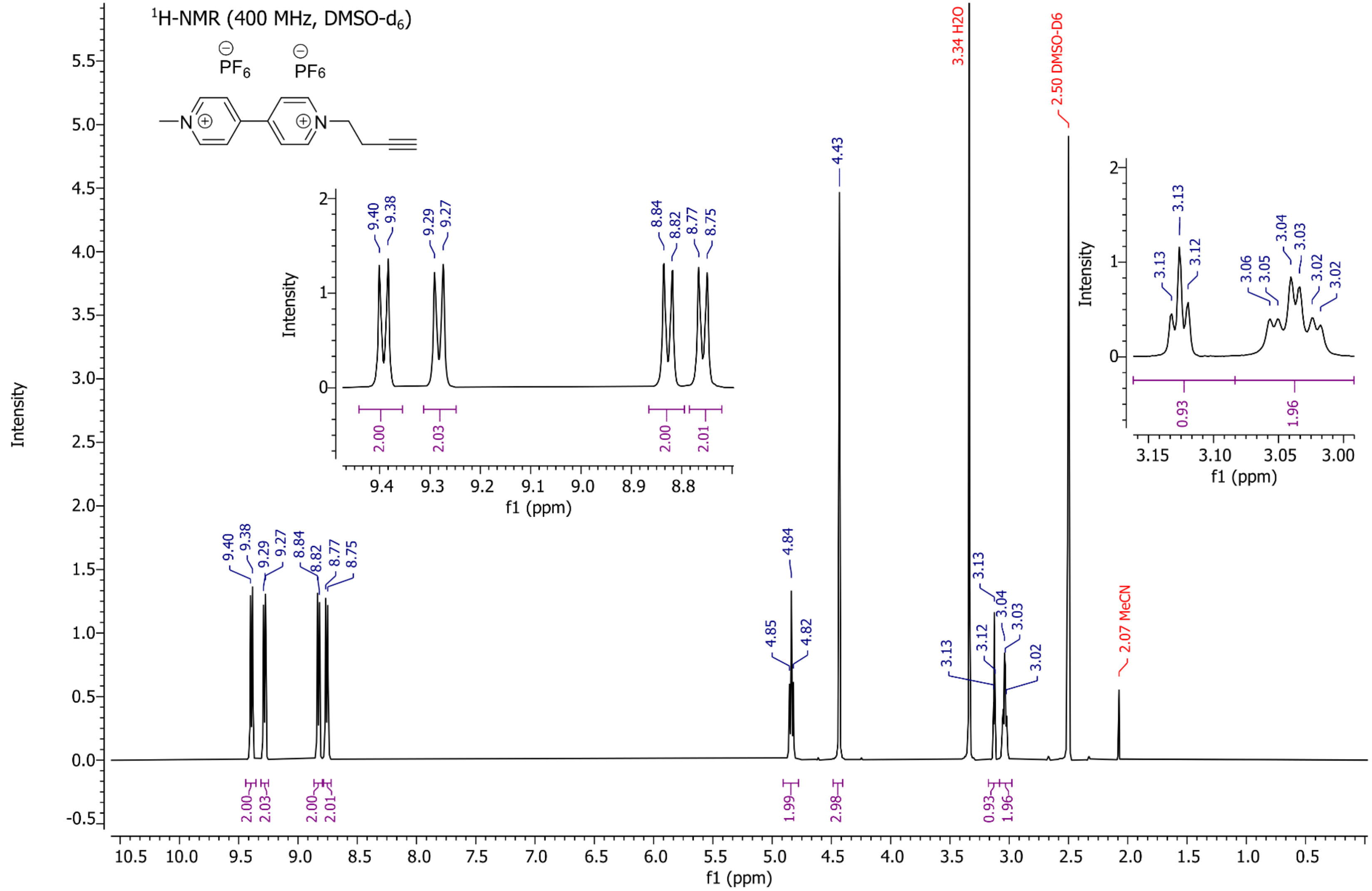

Figure S 97. ^{1}H-NMR spectrum of **S6**.

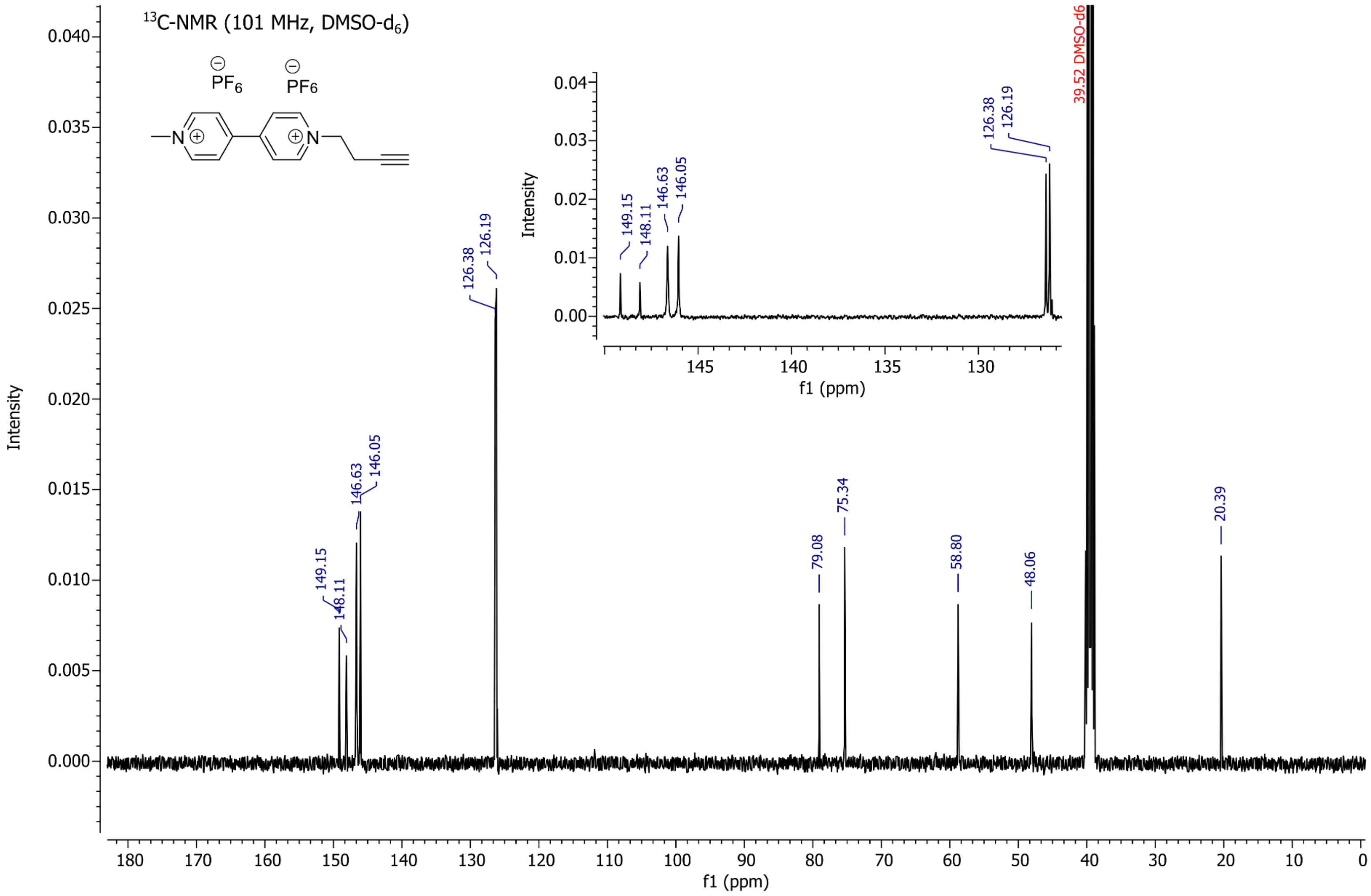

Figure S 98. ^{13}C-NMR spectrum of **S6**.

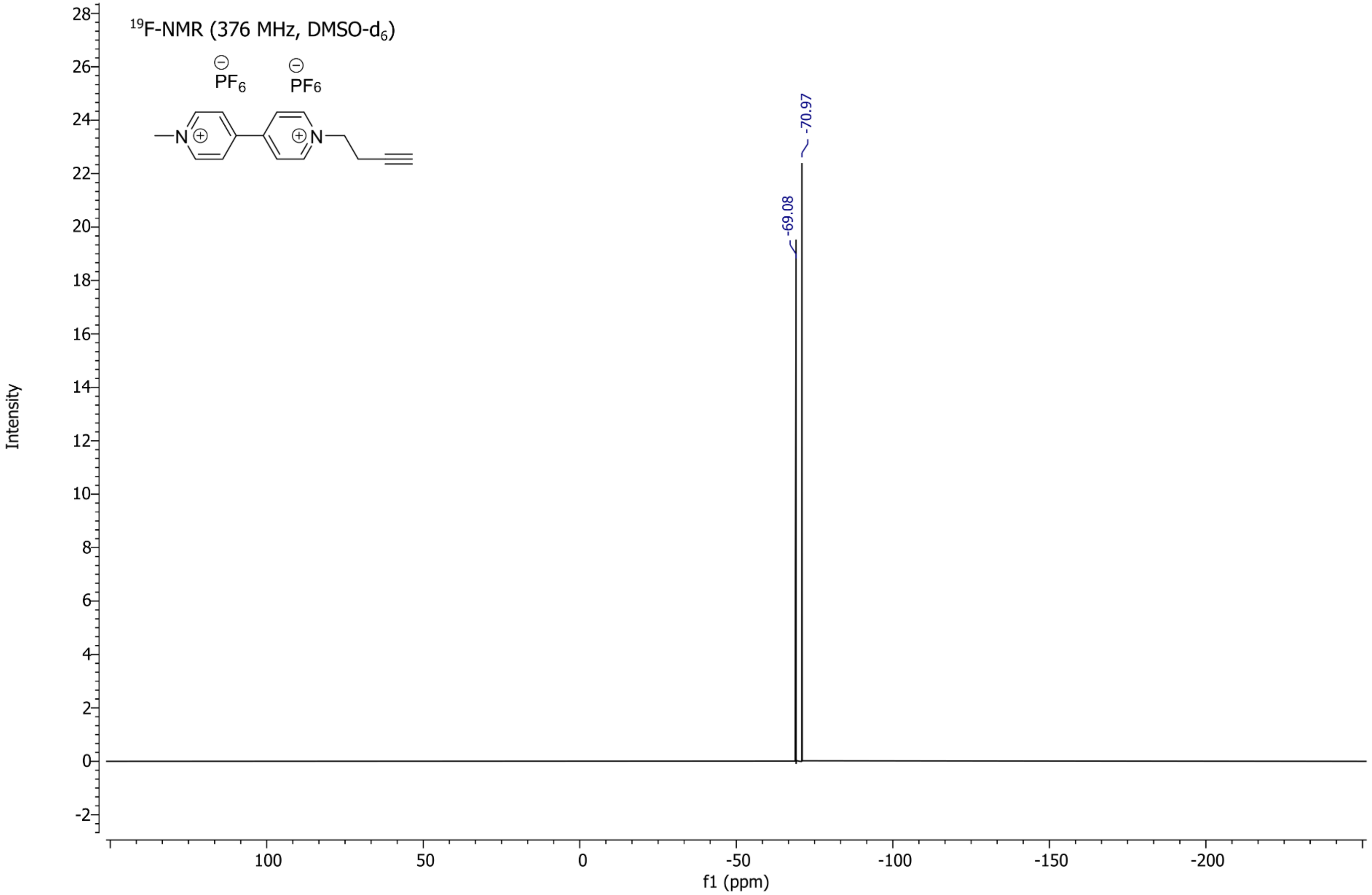

Figure S 99. ^{19}F-NMR spectrum of **S6**.

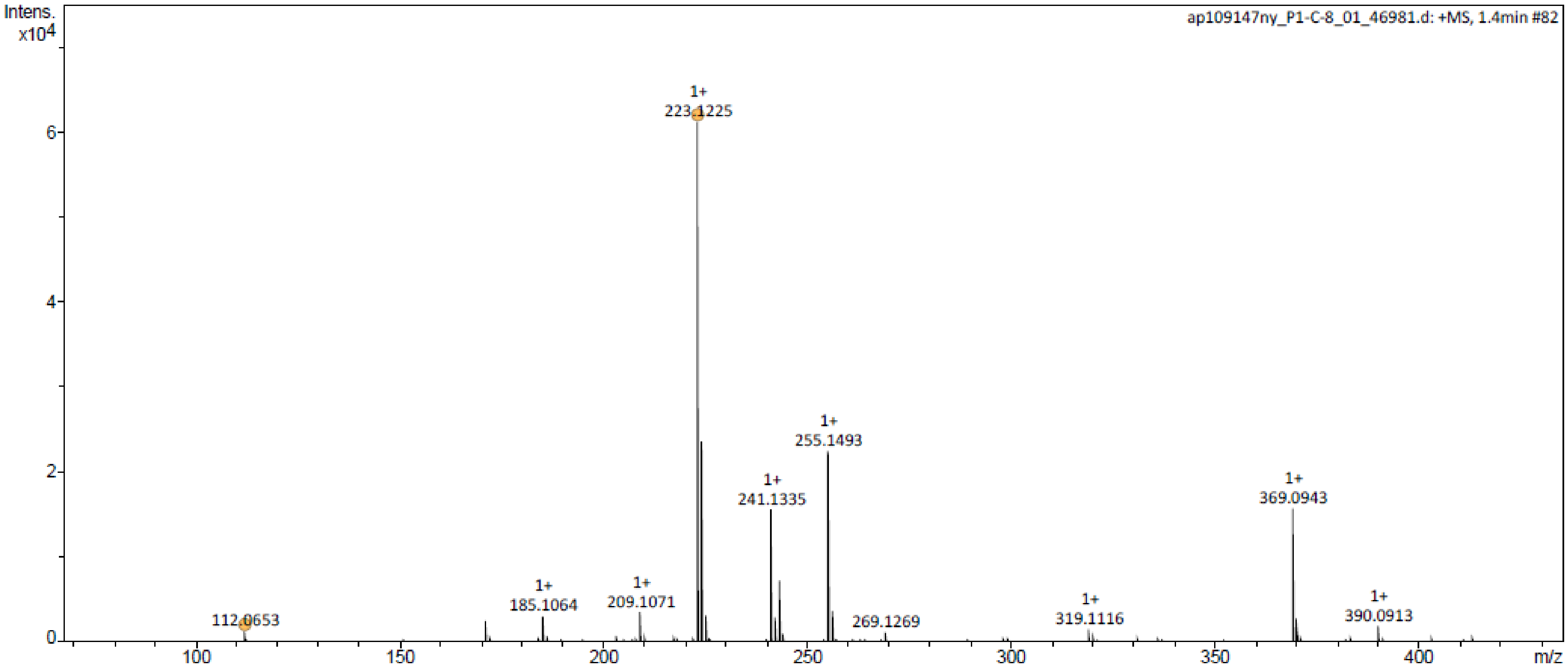

Meas. m/z	#	Ion Formula	m/z	err [ppm]	err [mDa]	mSigma	Mean err [ppm]
112.0653	1	C15H16N2	112.0651	-1.9	-0.2	15.0	0.7
223.1225	1	C15H15N2	223.1230	2.3	0.5	125.1	-4.3

Figure S 100. Positive mode (ESI)HRMS of **S6**.

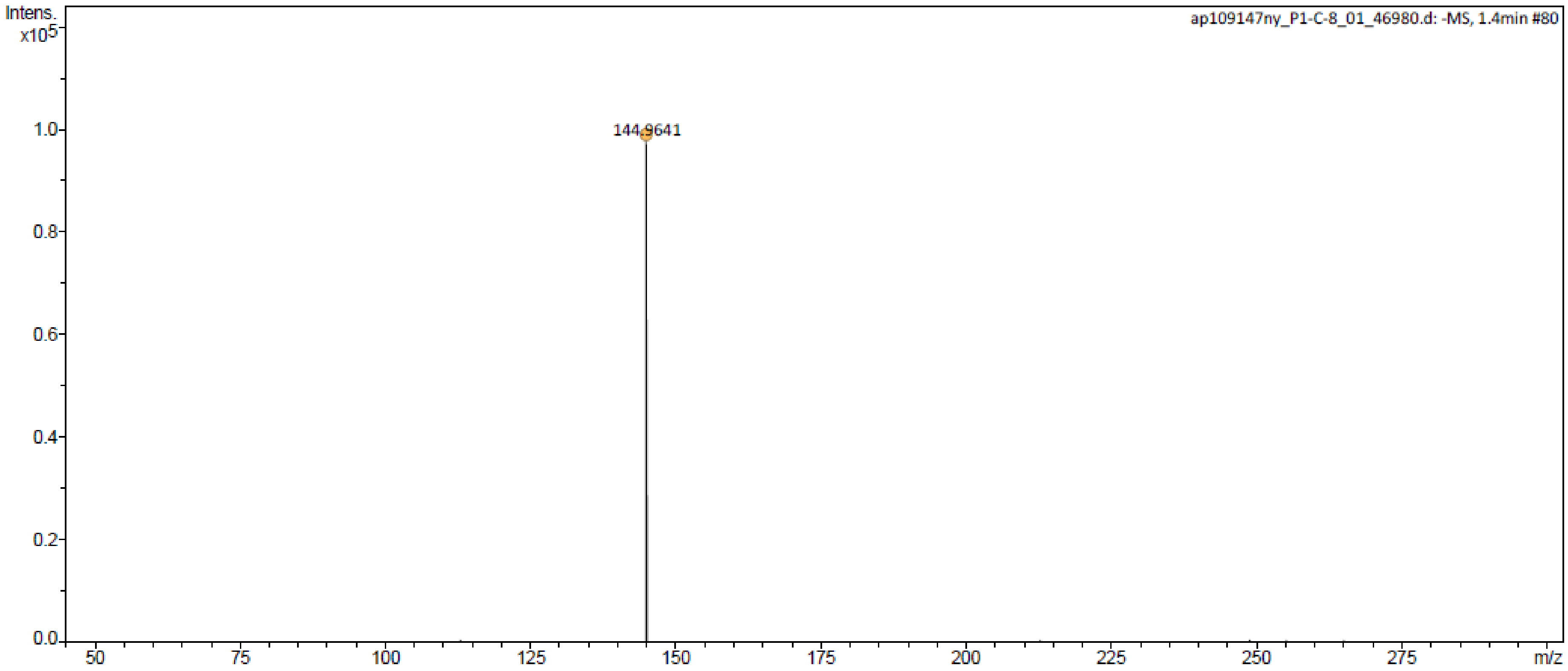

Meas. m/z	#	Ion Formula	m/z	err [ppm]	err [mDa]	mSigma	Mean err [ppm]
144.9641	1	F6P	144.9647	4.1	0.6	n.a.	4.1

Figure S 101. Negative mode (ESI)HRMS of **S6**.

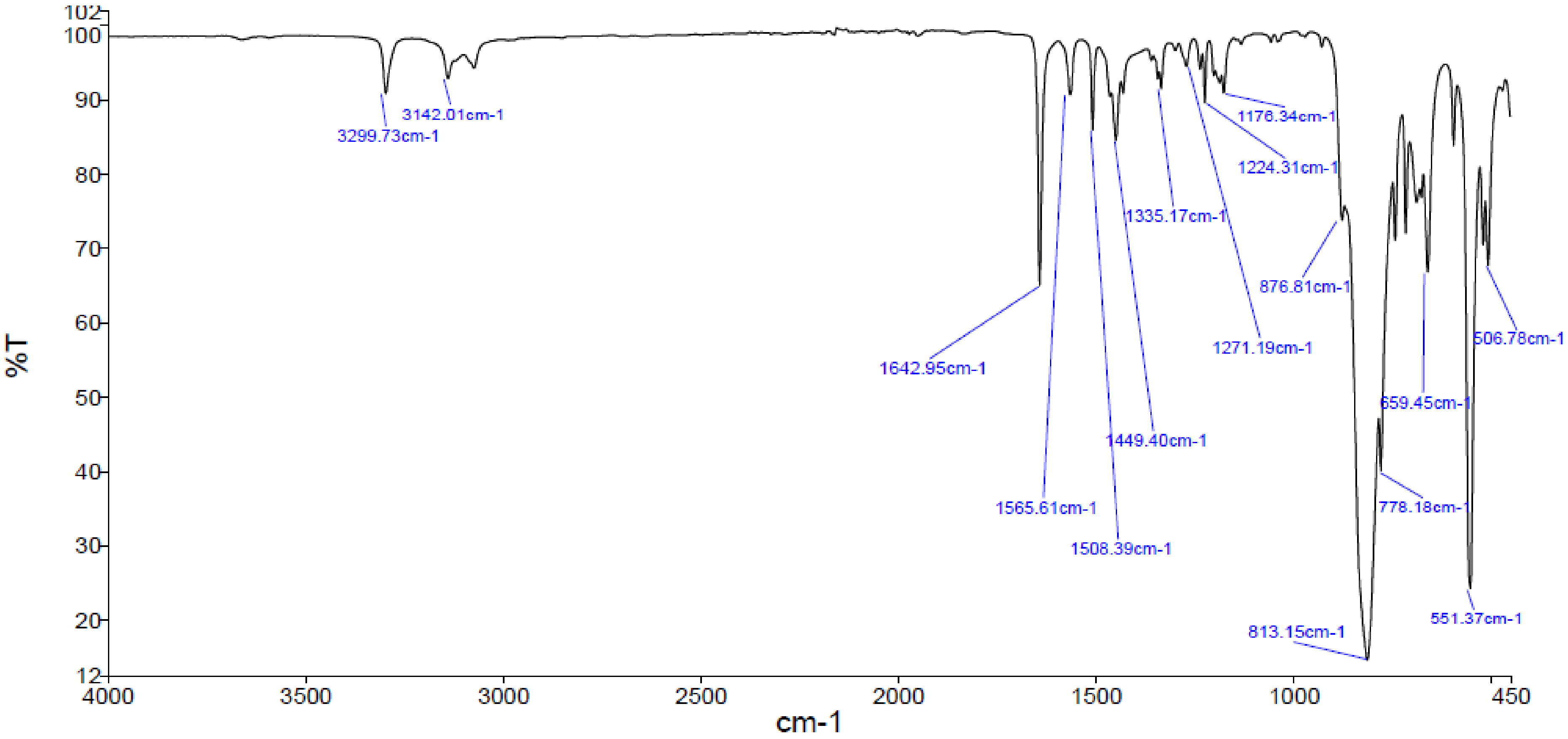

Figure S 102. FT-IR (ATR) spectrum of **S6**.

1-(but-3-yn-1-yl)-1'-methyl-[4,4'-bipyridine]-1,1'-diium chloride 13

To a stirred solution of **S6** (0.15 g, 0.29 mmol) dissolved in the minimum volume of MeCN was added a solution of tetrabutylammonium chloride (0.25 g, 0.9 mmol) dissolved in the minimum volume of MeCN. A pale off-white precipitate immediately formed. The resultant solution was briefly stirred and was then sonicated for 5 minutes to break apart any clumps. The product was then isolated by filtration, washed with additional MeCN and dried *in vacuo*. This yielded 1-(but-3-yn-1-yl)-1'-methyl-[4,4'-bipyridine]-1,1'-diium chloride **13** as an off-white powder (72 mg, 84%).

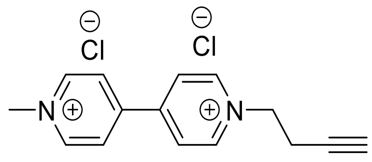

Figure S 103. The structure of **13**.

^{1}H-NMR (400 MHz, D_2O): δ_H 9.22-9.15 (m, 2H), 9.09-9.00 (m, 2H), 8.61-8.54 (m, 2H), 8.54-8.47 (m, 2H), 4.89 (t, *J* = 6.20 Hz, 2H), 4.50 (s, 3H), 3.04* (td, *J* = 6.20, 2.50 Hz, 2H), 2.52* (t, *J* = 2.50 Hz, 2H).

^{13}C-NMR (101 MHz, D_2O): δ_c 150.7, 149.8, 146.4, 145.8, 126.9, 126.7, 78.4, 74.1, 59.9, 48.4, 20.7.

FT-IR (ATR) (umax/cm^{-1}): 3167 (C-H stretch, alkyne), 2993 (C-H stretch), 2108 (C≡C stretch, terminal alkyne), 1640 (C=C stretch, aromatic), 823 (C-H bending).

(ESI)HRMS: Found 224.1296, $C_{15}H_{16}N_2^+$ requires 224.1308.

* The appearance of these peaks changes as a function of time when in D_2O. The signal at 3.04 ppm (attributable to the CH_2 environment nearest to the alkyne) retains the same integral yet becomes less triplet-of-doublet like and more triplet like in appearance, whereas the integral of the signal at 2.52 ppm (which is attributable to the proton of the terminal alkyne) reduces as a function of time. It seems likely that this is due to the exchange of the terminal alkyne proton for deuterium. This is further supported by HMQC evidence which struggles to find C-H coupling for the terminal alkyne environment when the sample has been incubated for 5 hours in D_2O.

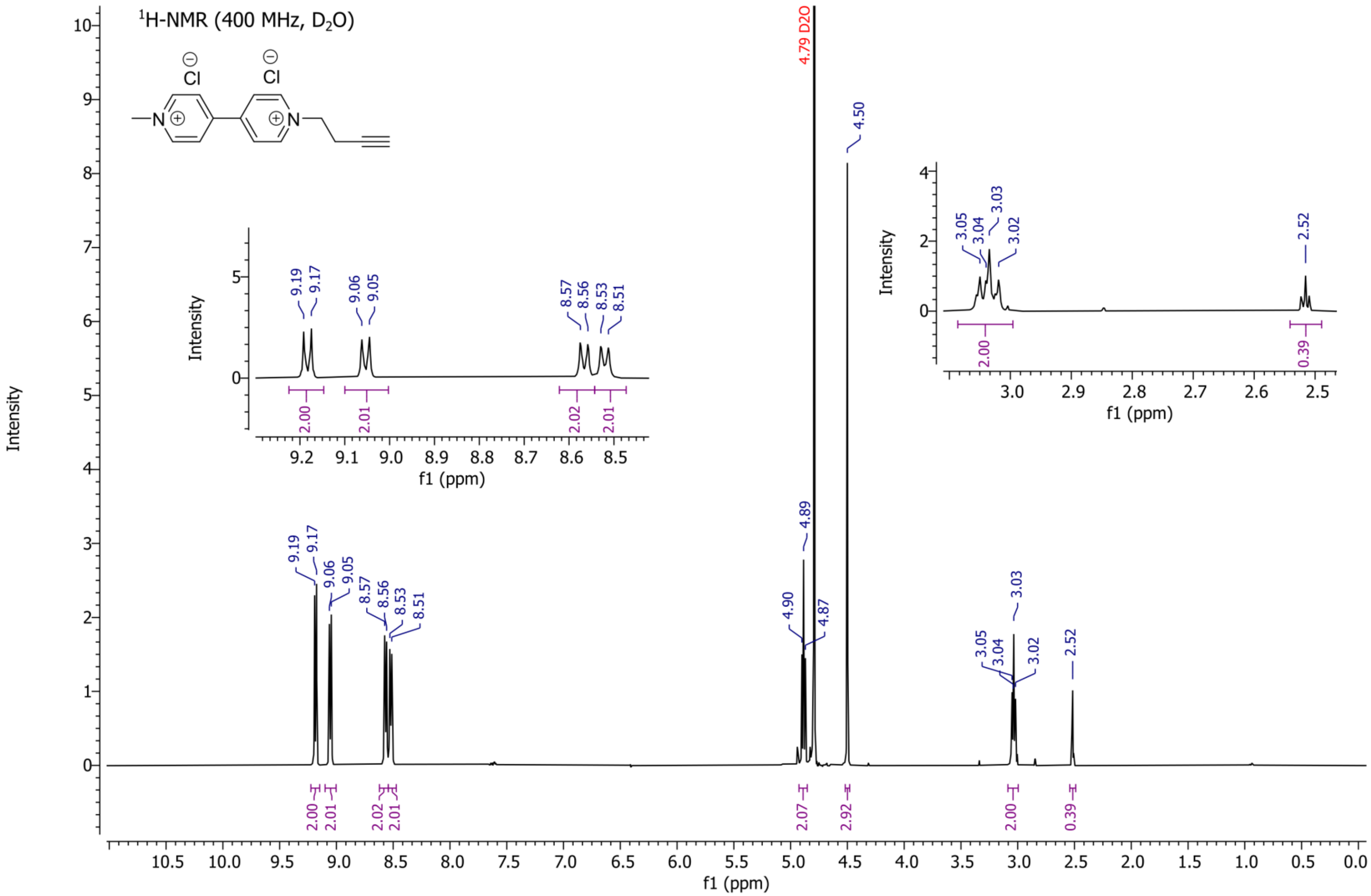

Figure S 104. ^{1}H-NMR spectrum of **13**.

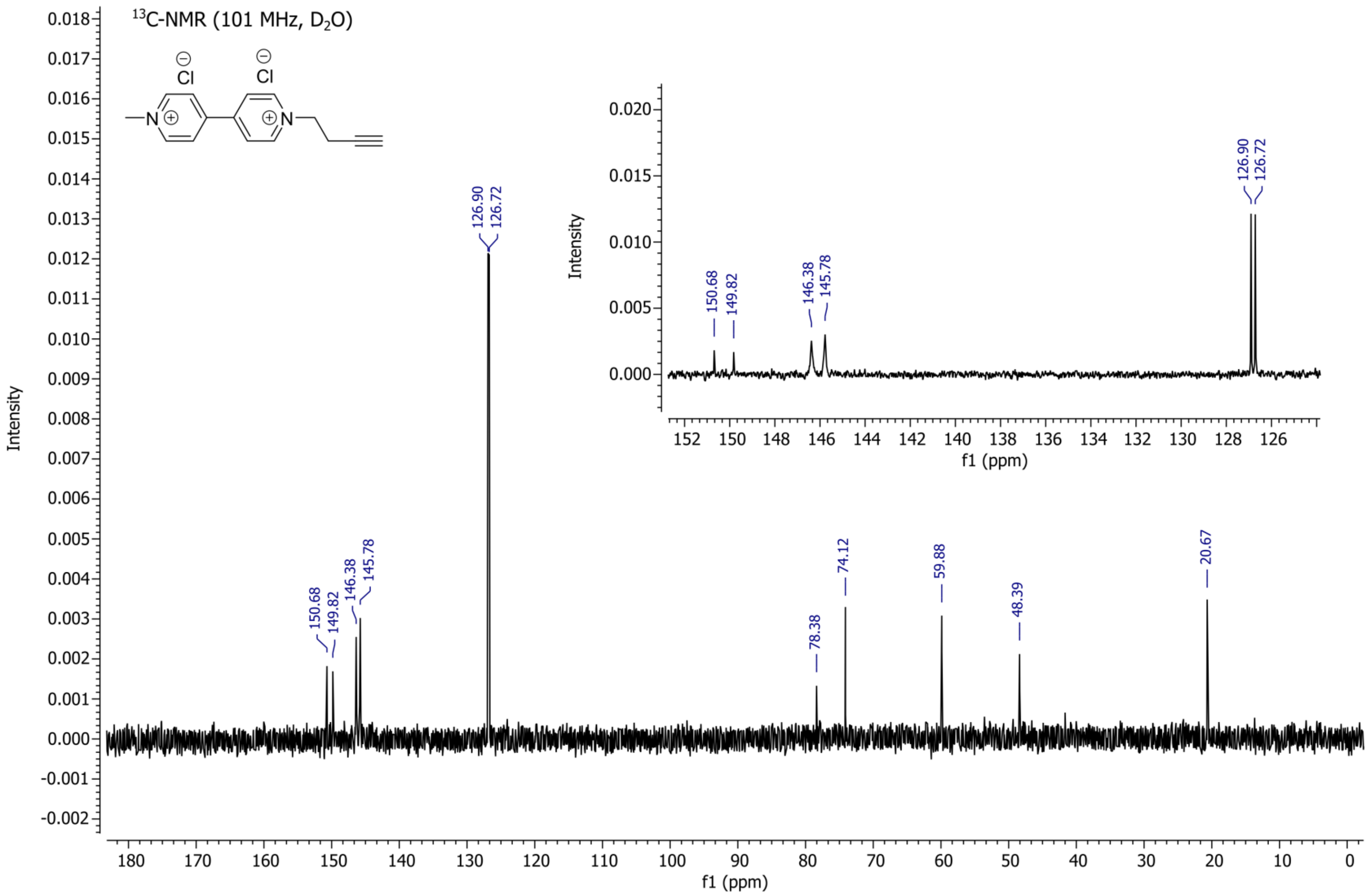

Figure S 105. ^{13}C-NMR spectrum of **13**.

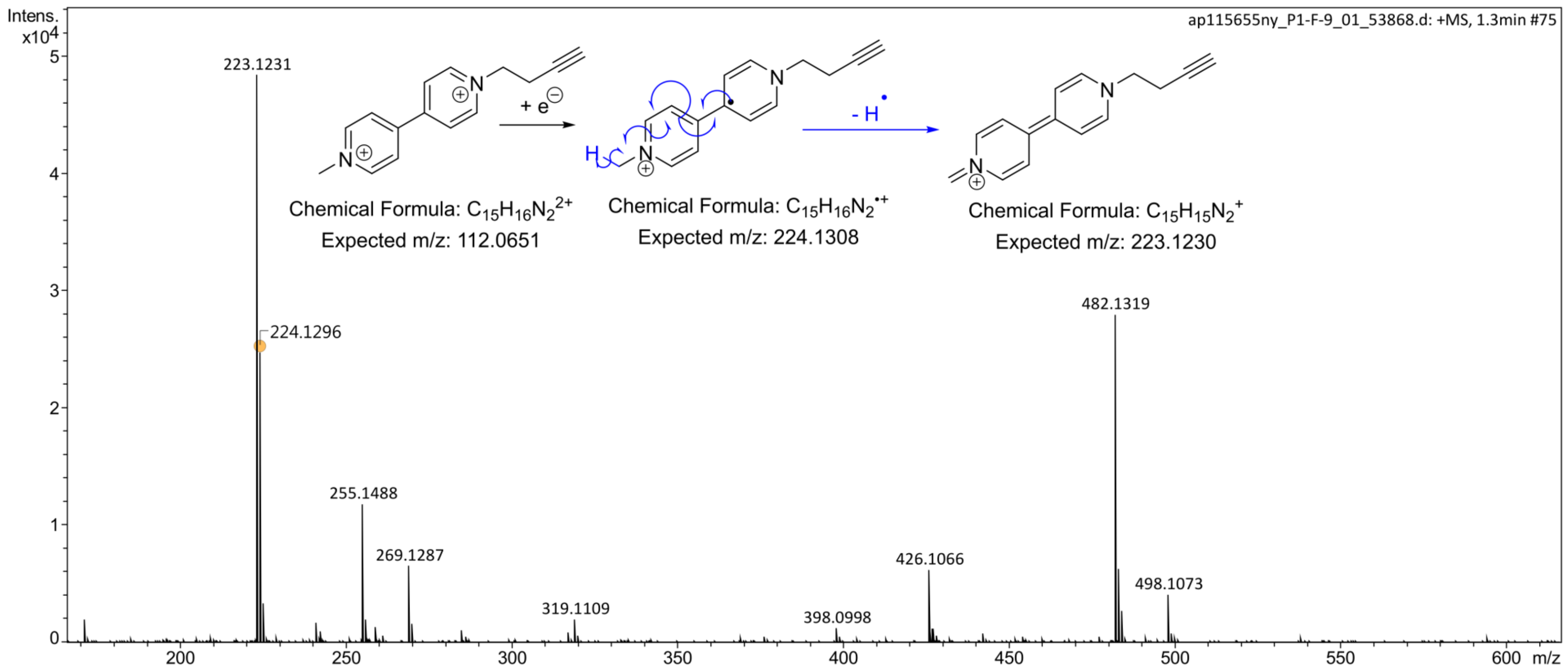

Meas. m/z	#	Ion Formula	m/z	err [ppm]	err [mDa]	mSigma	Mean err [ppm]
224.1296	1	C15H16N2	224.1308	5.4	1.2	21.5	1.2

Figure S 106. Positive mode (ESI)HRMS of **13**.

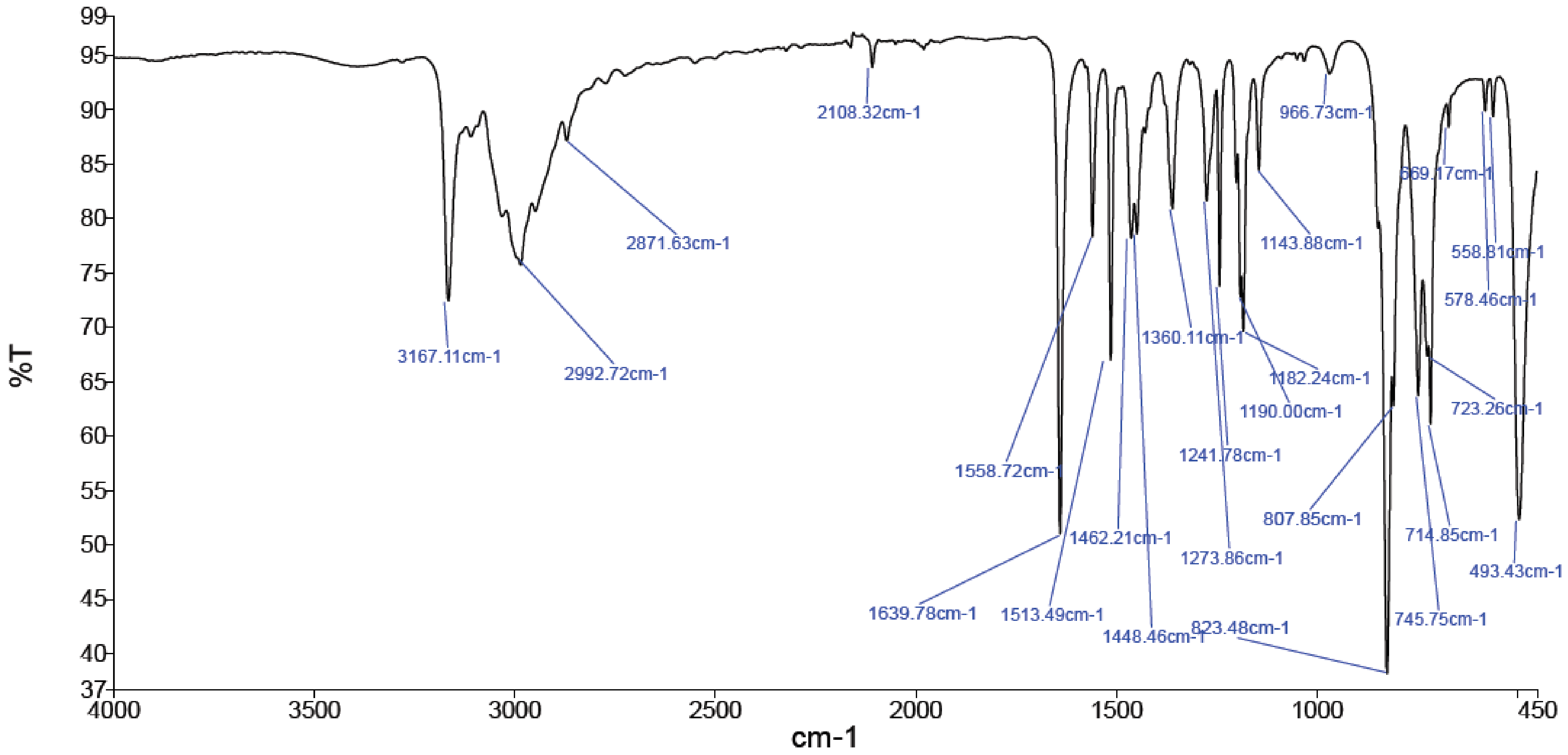

Figure S 107. FT-IR (ATR) spectrum of **13**.

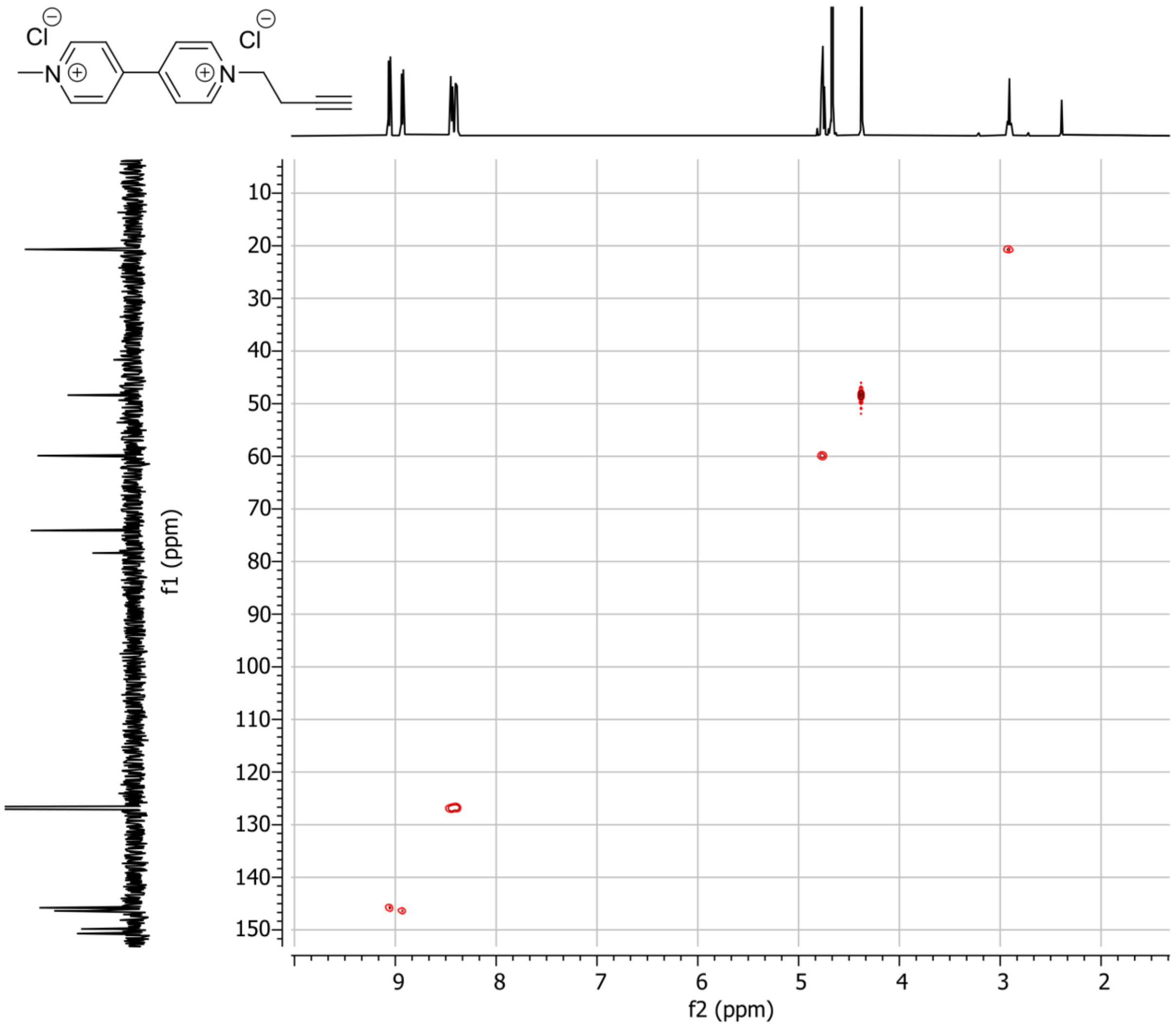

Figure S 108. ^{13}C-HMQC NMR spectrum of **13**.

3-(2-iminio-3-mesityl-2,3-dihydro-1H-imidazol-1-yl)propane-1-sulfonate S7

To a stirred solution of **4** (0.100 g, 0.13 mmol) dissolved in MeCN (10 mL) was added resorcinol (18 mg, 0.25 mmol). The solution was then stirred until all the resorcinol became dissolved, whereupon acetic acid (22 µL, 0.38 mmol) was added and the solution briefly stirred once again. The reaction vessel (a sample vial of 20 mL capacity) was then exposed to 365 nm UV irradiation from a commercially available UV nail curing product.* The solution changed from a vibrant yellow colour to an orange colour as the reaction progressed, and an orange precipitate appeared. After being subjected to UV irradiation for 1 hour, the reaction solution was filtered to remove as much orange precipitate as possible, and the eluate was concentrated *in vacuo*. The residue yielded was suspended in water (30 mL) and the aqueous suspension was washed with toluene. The resulting biphasic mixture was allowed to settle for 2 days to allow the emulsion to clear, and to allow any additional precipitates to leave solution. After this time the biphasic mixture was filtered to remove precipitated material, and was transferred to a separating funnel. The aqueous layer was isolated and was concentrated under a stream of nitrogen, and the resultant residue was re-dissolved in the minimum volume of water and further purified via reverse-phase flash chromatography on a Teledyne CombiFlash® NEXTGEN 300+ system using a C18 column (RediSep® Rf Gold C18 Reversed Phase Column, 5.5 gram media) which was pre-equilibrated with water, using a mobile phase gradient from water → MeCN over 16 minutes. Fractions containing **S7** were concentrated under a stream of nitrogen, and were then re-dissolved in the minimum volume of water, flash-frozen using liquid nitrogen and lyophilised to yield **S7** as an off-white fluffy solid (22 mg, 52%)

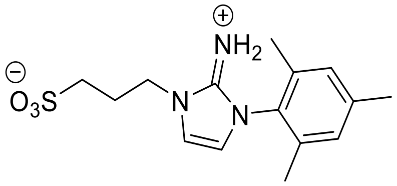

Figure S 109. The structure of **S7**.

^{1}H-NMR (400 MHz, D_2O): δ_H 7.13 (s, 2H), 7.10 (d, *J* = 2.50 Hz, 1H), 6.84 (d, *J* = 2.50 Hz, 1H), 4.11 (t, *J* = 7.05 Hz, 2H), 3.01-2.90 (m, 2H), 2.32 (s, 3H), 2.32-2.21 (m, 2H), 2.30 (s, 6H).

^{13}C-NMR (101 MHz, D_2O): δ_c 145.2, 141.5, 136.4, 129.5, 128.51, 116.7, 116.5, 47.3, 44.4, 23.7, 20.2, 16.4.

FT-IR (ATR) (υmax/cm^{-1}): 3099, 1660 (C=C stretch, aromatic), 1529, 1210 (S=O stretch), 1181 (S=O stretch), 1035.

(ESI)HRMS: Found 346.1203, $C_{15}H_{21}N_3Na_2O_3S^+$ requires 346.1196.

* This product was a *nailstar*® 36-Watt Professional UV Nail Lamp (Model: NS-01-UK&EU). This product is fitted with 4 replaceable 9 W U-shaped tube bulbs that emit at 365 nm and has a mirrored interior.

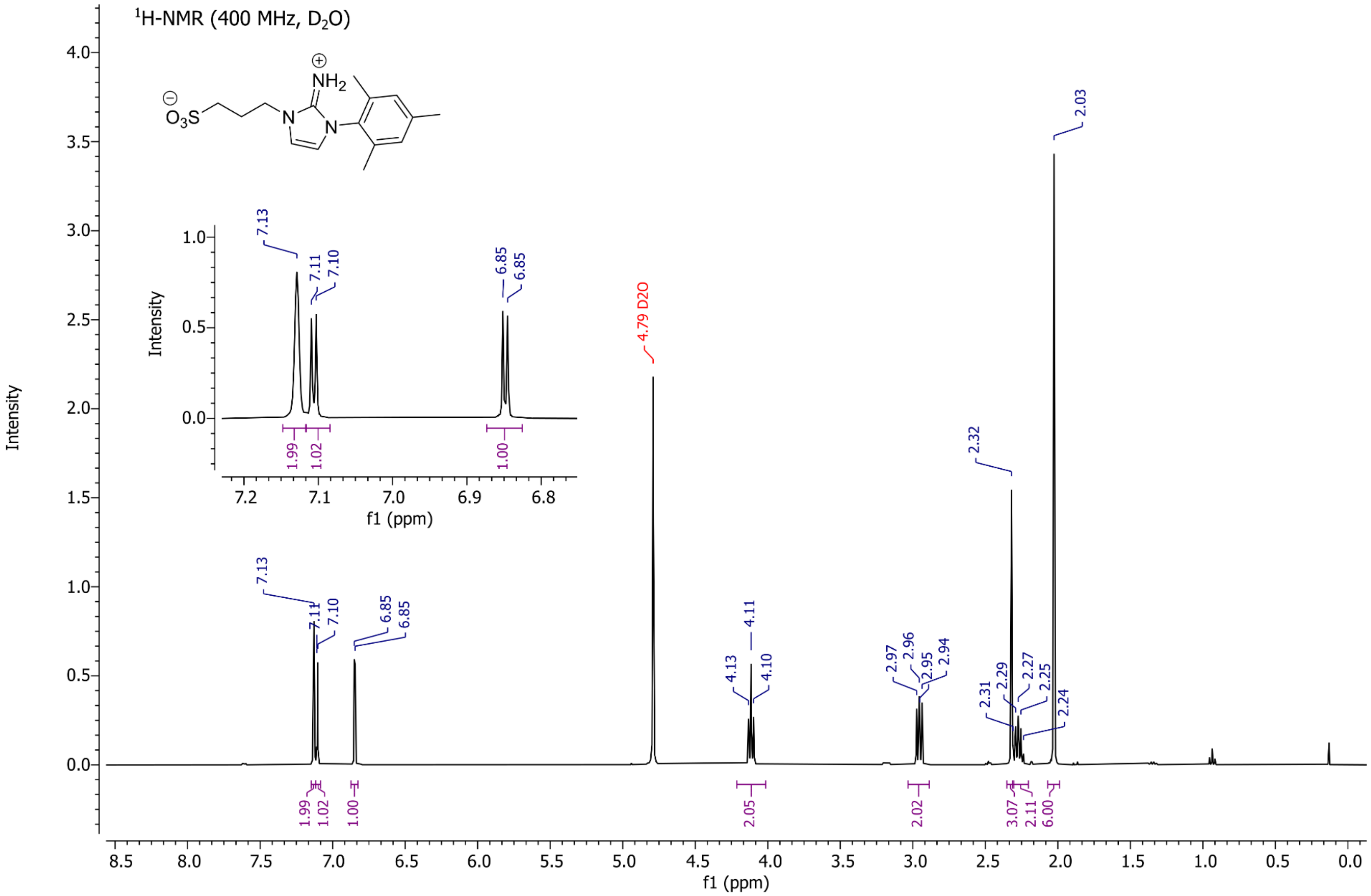

Figure S 110. ^{1}H-NMR spectrum of **S7**.

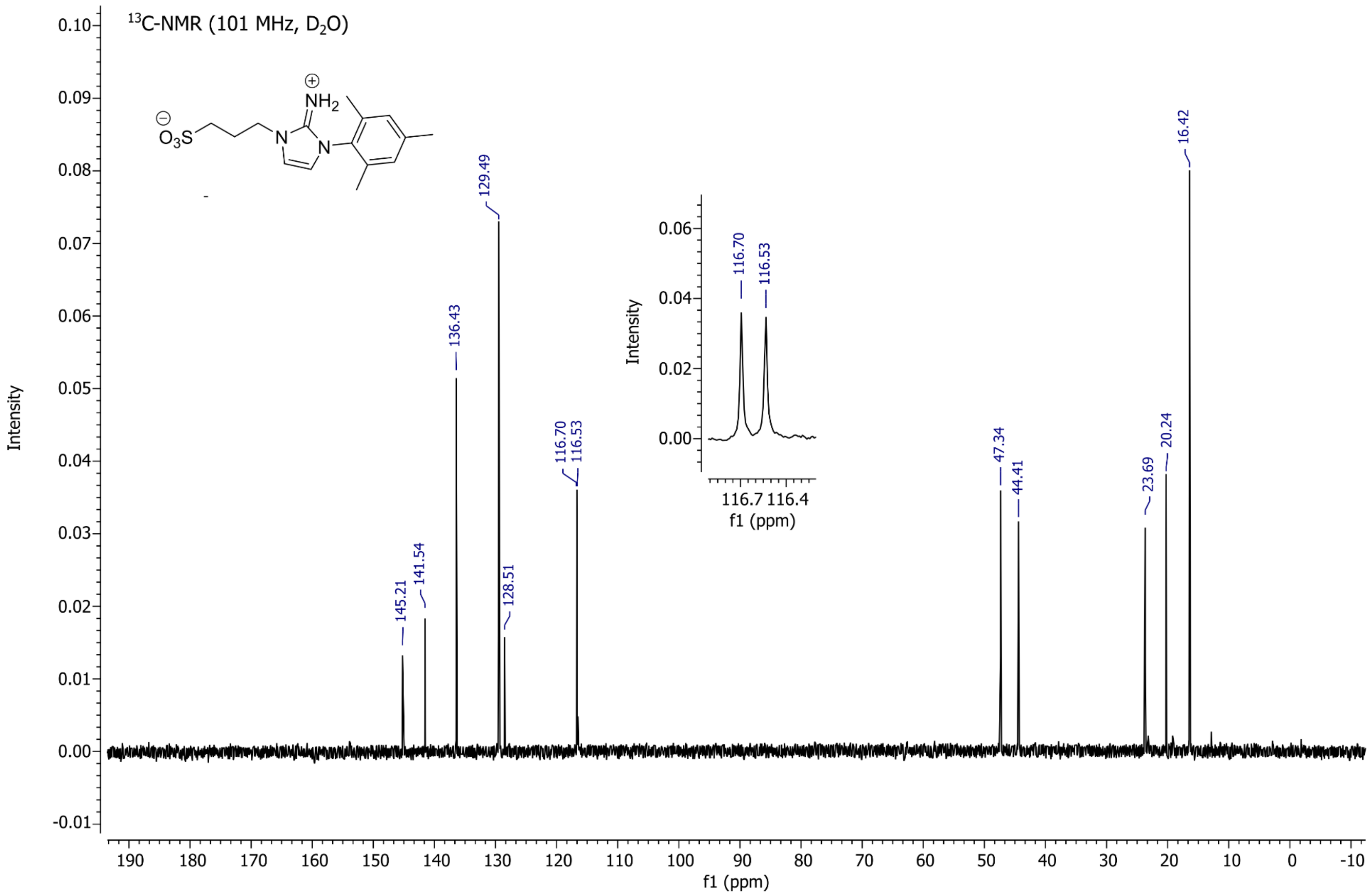

Figure S 111. ^{13}C-NMR spectrum of **S7**.

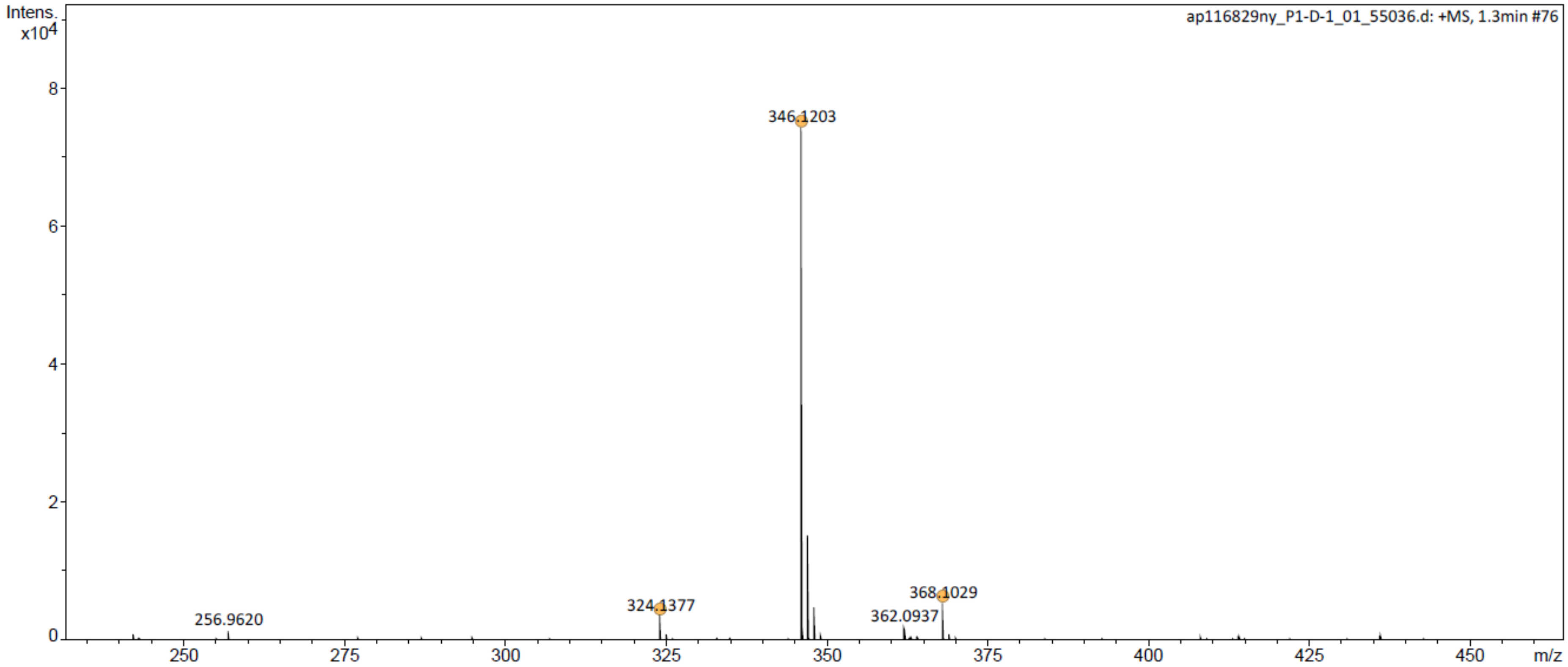

Meas. m/z	#	Ion Formula	m/z	err [ppm]	err [mDa]	mSigma	Mean err [ppm]
324.1377	1	C15H22N3O3S	324.1376	-0.1	-0.0	37.9	0.6
346.1203	1	C15H21N3NaO3S	346.1196	-2.0	-0.7	10.3	-2.0
368.1029	1	C15H20N3Na2O3S	368.1015	-3.7	-1.4	12.3	-5.2

Figure S 112. Positive mode (ESI)HRMS of **S7**.

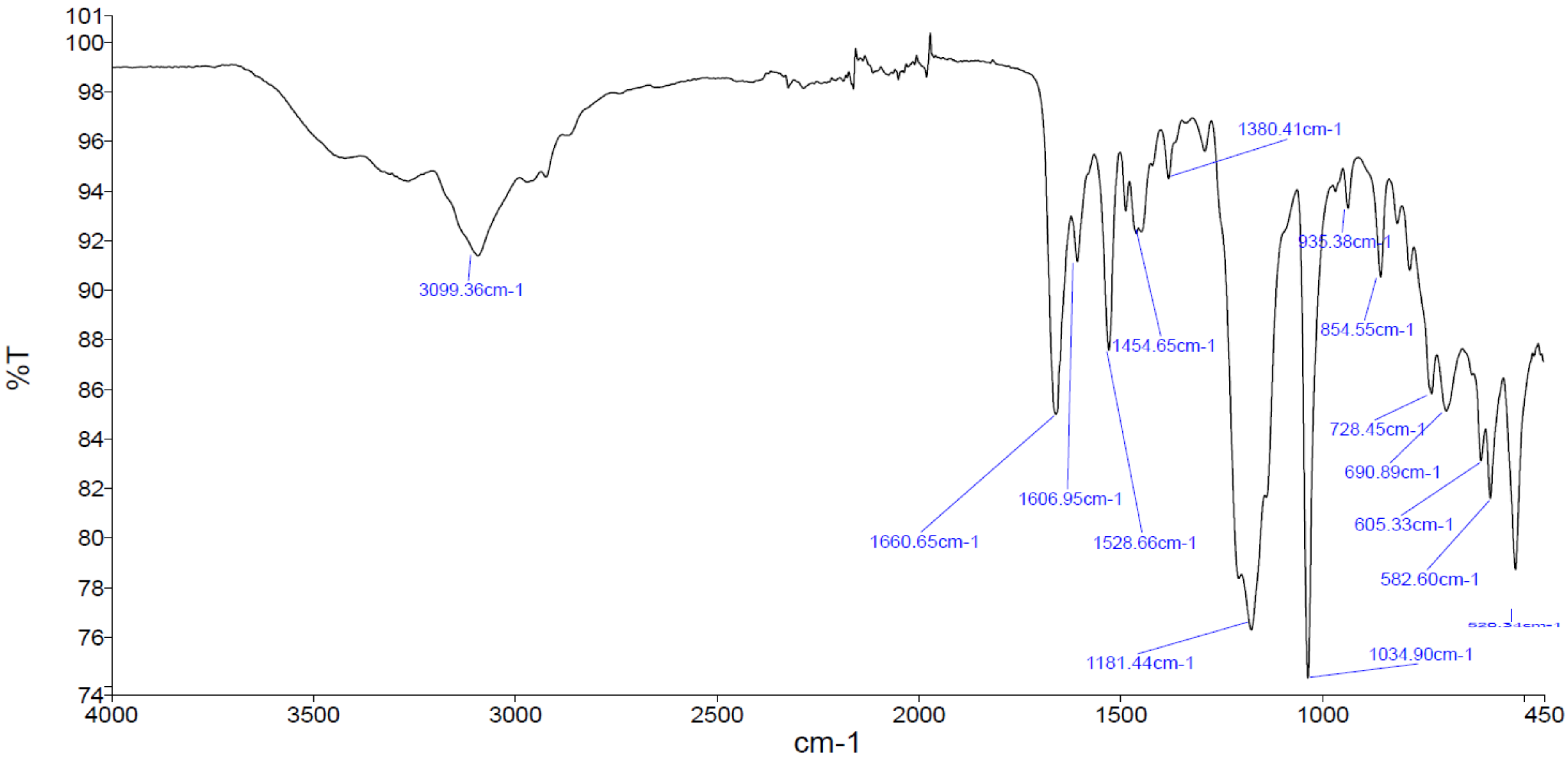

Figure S 113. FT-IR (ATR) spectrum of **S7**.

UV-vis analysis of 4

General buffers:

pH 8.5 UV-vis buffer: 50 mM sodium phosphate + 150 mM NaCl, pH 8.5.
pH 8.0 UV-vis buffer: 50 mM sodium phosphate + 150 mM NaCl, pH 8.0.
pH 7.0 UV-vis buffer: 50 mM sodium phosphate + 150 mM NaCl, pH 7.0.
pH 6.0 UV-vis buffer: 50 mM sodium phosphate + 150 mM NaCl, pH 6.0.

Half-life measurements of 4

In order to measure the half-life of triazabutadiene **4** in aqueous solution the absorbance at the λ_{max} of the triazabutadiene signal (~395 nm) was monitored at 24°C as a function of time for 30 µM solutions of **4** in UV-vis buffer (pH 7.0, 8.0, or 8.5). Quartz cuvettes (1 mL volume and 1 cm path length) were used, and data was recorded using a UV-1800 Shimadzu Spectrophotometer.

It can be assumed that, in buffered aqueous solution (i.e. where $[H^+]$ is effectively constant) in darkness, the rate at which dilute solutions of triazabutadienes breaks down due to protonation-triggered aryl diazonium release would follow first order kinetics. The collected data of Abs vs time was therefore fitted via non-linear regression to **Equation S1**, where Abs_0 is the initial absorbance reading recorded and k is the first order rate constant. Having determined values for k via this method, it was thereafter possible to calculate values for half-life using **Equation S2**:

$$Abs = Abs_0 e^{-kt}$$ **Equation S1**

$$t_{\frac{1}{2}} = \frac{\ln 2}{k}$$ **Equation S2**

UV-triggered aryl diazonium release from 4

50 µM solutions of **4** were prepared via the delivery of 100 µL of a 500 µM stock solution of **4** in DMSO to 900 µL of UV-vis buffer (pH 6.0, pH 7.0 or pH 8.0) in a quartz cuvette (1 mL volume and 1 cm path length). Initial UV-vis spectra were then recorded using a DeNovix Ds-11 FX+ spectrophotometer.

UV irradiation of samples was then performed using a commercially available UV nail curing product - the *nailstar*® 36-Watt Professional UV Nail Lamp (Model: NS-01-UK&EU). This product is fitted with 4 replaceable 9 W U-shaped tube bulbs that emit at 365 nm and has a mirrored interior. The quartz cuvettes were stood in a shallow dish of icy water inside the *nailstar*®. The samples were then exposed to 15 s periods of UV irradiation, with UV-vis spectra being recorded after each irradiation period.

Electrochemistry

General considerations

Experiments were conducted in a glovebox (in-house design and construction) under an N_2 atmosphere (O_2 ≤ 40 ppm). A PalmSens4 potentiostat (PalmSens) with PSTrace 5.9 for Windows software was used for all electrochemical experiments.

Potentials are reported versus the SHE reference electrode. These potentials were calculated using **Equation S3**, where C was calculated for each reference electrode used by recording a cyclic voltammogram in a standard ferricyanide solution and comparing the midpoint potential to the literature value.[29]

$$E(V\ vs\ SHE) = E(V\ vs\ REF) + \mathrm{C}\ (V) \qquad \textbf{Equation S3}$$

C was + 240 mV for the Ag/AgCl (3 M NaCl) reference electrode and was +310 mV for the Ag/AgCl reference electrodes integrated into the SPEs.

General buffers:

pH 8.0 EChem buffer: 50 mM sodium phosphate, 150 mM NaCl, pH 8.0.
pH 7.0 EChem buffer: 50 mM sodium phosphate, 150 mM NaCl, pH 7.0.

Electrodes and electrode preparation steps

3 mm Disk Working Electrodes:
Glassy carbon (product reference: ET051-3) and gold (product reference: ET053-3) 3 mm diameter disk working electrodes were manufactured by eDAQ and were purchased through Green Leaf Scientific. The structure of these electrodes is depicted in **Figure S114**.

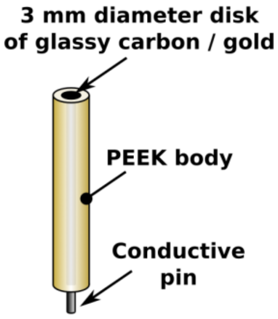

Figure S 114. Glassy carbon / gold 3 mm disk electrodes.

These electrodes were cleaned prior to each diazonium/triazabutadiene grafting experiment via mechanical polishing for 2 min using 1 µm alumina slurry impregnated onto a *WhiteFelt* polishing pad (Buehler). Electrodes were then rinsed with Milli-Q water and sonicated in Milli-Q water for 5 min.

Screen Printed Electrodes:
The screen printed electrodes (SPEs) utilised in this publication were manufactured by PalmSens and were purchased from Alvatek (product reference BVT-AC1.W4.R2 Dw2). As is depicted in **Figure S115**, these SPEs feature a carbon working electrode of a diameter of 2.4 ± 0.1 mm, a reference electrode comprised of Ag covered with AgCl, and a counter electrode made of platinum. 40 µL of electrolyte solution was sufficient to cover all three electrodes and thereby complete the electrochemical cell.

These SPEs were treated as single-use, and consequently no preparative/cleaning steps were employed prior to using these SPEs in experiments, they were simply used as supplied.

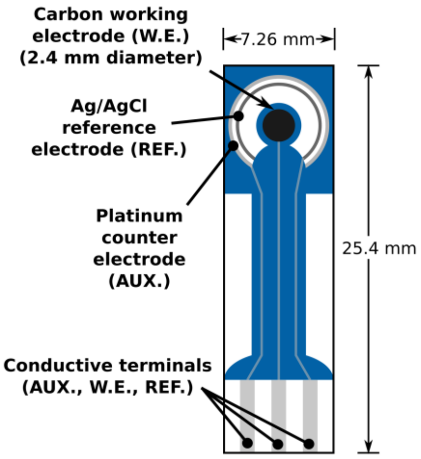

Figure S 115. Carbon SPE BVT-AC1.W4.R2 Dw2.

Gold-Coated Silicon Wafers:
Gold-coated silicon wafer (99.999% Au, Au layer thickness: 1000 Å, 99.99% Ti adhesion layer) was purchased from Sigma-Aldrich and cut into 6×12 mm rectangles.

Prior to performing electrochemical experiments using these slices (in which these slices were used as working electrodes), a solution of acidic piranha (**Caution**: Piranha solution reacts violently with organic matter and should be handled with extreme care!) was prepared by slowly adding 1 part of 30% hydrogen peroxide to 3 parts of concentrated sulfuric acid. The 6 mm × 12 mm gold-coated silicon wafer samples were immersed in 40 mL of acidic piranha solution and were removed after 1 h. The gold substrates were then rinsed with HPLC-grade water prior to being used for electrochemical grafting experiments.

ITO-Coated Glass Slides:
Indium tin oxide (ITO) coated glass slides (25 mm × 25 mm × 1.1 mm, surface resistivity 70-100 Ω/sq) were purchased from Sigma Aldrich. These ITO-coated glass slides were cut into 7 × 25 × 1.1 mm slices and were then rinsed with HPLC-grade water and allowed to dry prior to being used for electrochemical grafting experiments.

Ag/AgCl (3 M NaCl) Reference Electrode
Ag/AgCl (3 M NaCl) reference electrodes (product reference: MF-2052) were manufactured by BASi and were purchased through Alvatek. The structure of these electrodes is depicted in **Figure S116**.

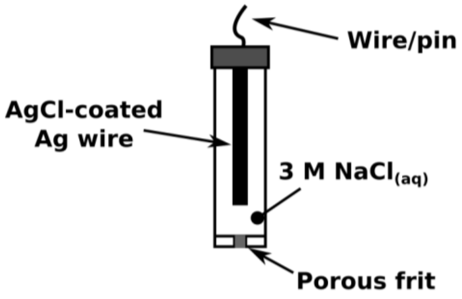

Figure S 116. Ag/AgCl (3 M NaCl) reference electrode.

Platinum Wire Counter Electrode
The Pt wire counter electrode was made in-house from wire of 1 mm diameter, purchased from Sigma-Aldrich.

Experimental procedures

Grafting 4/5 onto screen-printed electrodes (SPEs):
To prepare a 1 mM of **4** suitable for electrografting experiments, 50 µL of a 10 mM solution of **4** in acetonitrile was added to 450 µL of pH 7.0 EChem buffer at 0°C. This solution was then either kept in darkness or exposed to 365 nm UV irradiation from a *nailStar®* 36 Watt UV lamp (Model: NS-01-UK&EU) for 30 s at 0°C.

To prepare a 1 mM solution of **5** suitable for electrografting, 50 µL of a 10 mM solution of **4** in water (prepared at 0°C) was added to 450 µL of pH 7.0 EChem buffer at 0°C.

When performing electrografting experiments using **4**/**5**, 40 µL of the relevant electrografting solution was applied to a fresh SPE (i.e. an SPE freshly removed from its original packaging) such that all three electrodes present on the SPE were immersed.

Electrografting was performed via cyclic voltammetry using the parameters tabulated below:

**Equilibration time (s):**	10
**E begin (V vs REF):**	0.5
**E vertex1 (V vs REF):**	0.5
**E vertex2 (V vs REF):**	-0.5
**E step (V):**	0.01
**Scan rate (mV s^{-1}):**	100
**Number of scans**	5

Note that while the temperature of the electrografting solution applied to the SPE can be assumed to be 0°C at the point of application, it was not practical to provide additional cooling to the solution during electrografting.

Grafting 4/5 onto 3 mm disk glassy carbon electrodes:
1 mM solutions of **4** or **5** suitable for electrografting experiments were prepared using the stock solutions/buffers listed below and the recipes tabulated below. Each solution was prepared such that the acetonitrile content was 10% and the total volume was 2 mL.

Stock solutions/buffers:

- **4 stock:** 20 mM **4** in acetonitrile
- **5 stock:** 20 mM **5** in pH 7.0 EChem buffer (kept at 0°C).

Recipes:

**Condition**	**Volume of 4 stock (µL)**	**Volume of 5 stock (µL)**	**Volume of pH 7.0 Echem buffer (µL)**	**Volume of acetonitrile (µL)**
**4** (+ or - UV)	100	0	1800	100
**5**	0	100	1700	200
Blank	0	0	1800	200

Each solution was prepared at 0°C. Solutions of **4** were then either kept in darkness or exposed to 365 UV irradiation from a *nailStar*® 36 Watt UV lamp (Model: NS-01-UK&EU) for 30 s at 0°C. After being prepared the solutions were transferred into a glass vessel able to accommodate a 2 mL volume prior and three electrodes were inserted - a Ag/AgCl (3 M NaCl) reference electrode, a platinum wire counter electrode and a 3 mm diameter glassy carbon electrode, yielding a complete 3-electode electrochemical cell. Electrografting was performed via cyclic voltammetry using the parameters tabulated below:

**Equilibration time (s):**	5
**E begin (V vs REF):**	0.5
**E vertex1 (V vs REF):**	0.5
**E vertex2 (V vs REF):**	-0.5
**E step (V):**	0.005
**Scan rate (mV s^{-1}):**	20
**Number of scans**	3

Note that while the temperature of the electrografting solution can be assumed to be 0°C at the point of preparation, it was not practical to provide additional cooling to the solution during electrografting.

Grafting 4/5 onto gold-coated silicon wafer slices:

When using 6×12 mm slices of gold-coated silicon wafer to prepare samples for XPS analysis it was necessary to perform the electrografting experiments in small beaker using a 10 mL volume of electrografting solution (**Figure S117**). The need for a larger volume of electrografting solution was purely due to the practical constraints of attempting to immerse a slice of gold-coated silicon wafer (electronically connected as the working electrode via a crocodile-clip) into the solution alongside a platinum wire counter electrode and a Ag/AgCl (3 M NaCl) reference electrode. An ice bath was used to keep the electrografting solution at ~ 0°C.

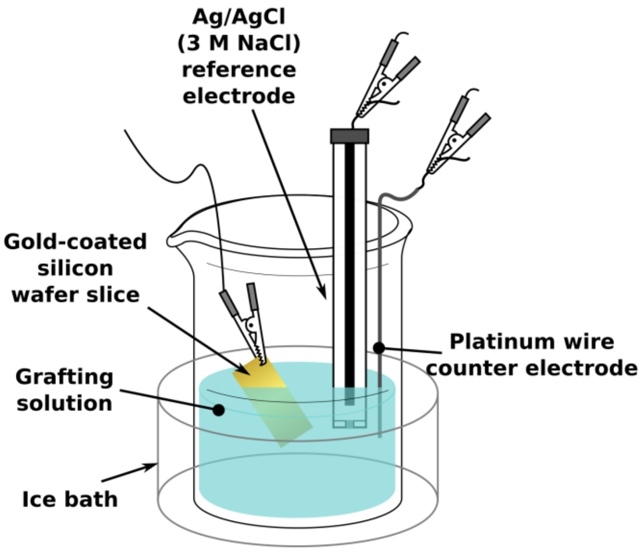

Figure S 117. The 3-electrode setup used to perform electrografting experiments onto gold-coated silicon wafer slices.

To prepare a 1 mM solution of **4** suitable for electrografting, 100 µL of a 100 mM solution of **4** in DMF was added to 9.9 mL of pH 8 EChem buffer at 0°C. This solution was then either kept in darkness or exposed to 365 nm UV irradiation from a *nailStar*® 36 Watt UV lamp (Model: NS-01-UK&EU) for 30 s at 0°C.

To prepare a 1 mM of **5** suitable for electrografting, 3.2 mg of **5** was dissolved in 9.9 mL of pH 8.0 EChem buffer at 0°C. 100 µL of DMF was then added prior to performing electrografting experiments.

To prepare a blank electrografting solution, 100 µL of DMF was added to 9.9 mL of pH 8.0 EChem buffer at 0°C.

Experiments were conducted on 6×12 mm slices using the permutations of conditions tabulated below:

**Sample name / conditions**
Blank
**4**, not UV exposed, not electrografted
**4**, not UV exposed, electrografted
**4**, UV exposed, not electrografted
**4**, UV exposed, electrografted
Diazonium salt **5**, electrografted

For the “not electrografted” samples, the slices of gold-coated silicon wafer were immersed in electrografting solution (as shown in **Figure S117**), but cyclic voltammetry was not performed.

For “electrografted” samples, the slices of gold-coated silicon wafer were immersed in electrografting solution (as shown in **Figure S117**) and cyclic voltammetry was performed using the parameters tabulated below.

**Equilibration time (s):**	10
**E begin (V vs REF):**	0.2
**E vertex1 (V vs REF):**	0.2
**E vertex2 (V vs REF):**	-0.5
**E step (V):**	0.01
**Scan rate (mV s^{-1}):**	20
**Number of scans**	5

After performing electrografting/sample immersion, the slices of gold-coated silicon wafer were removed from the electrografting solution and were rinsed thoroughly with HPLC-grade water. The slices were then placed inside sample vials filled with HPLC grade water and were sent for analysis via XPS.

Aliquoting of 10:

10 was dissolved in anhydrous DMSO and the resulting solution sub-aliquoted into Eppendorf tubes such that each Eppendorf tube contained 4 × 10^{-8} moles of **10**. Dioxane was then added, such that the DMSO concentration was below 5% v/v. The aliquots were then flash-frozen using liquid N_2 and lyophilized, removing both the DMSO and dioxane. The resulting samples of **10** were then stored in darkness at -20°C for future use.

Exploring the grafting behaviour of 10 using 3 mm disk working electrodes:

It was possible to perform electrografting experiments using as little as 40 µL of sample/electrolyte when using 3 mm diameter gold / glassy carbon disk electrodes. In order to do this, a universal clear blue plastic 1000 µL pipette tip was cut using a scalpel, such that the thinner portion was removed. The remaining part of the pipette tip was pushed over the tip of the disk electrode, forming a collar around the tip of the working electrode. The body of the working electrode was then clamped in a vertical orientation, such that the collar points upwards. A Ag/AgCl (3 M NaCl) reference electrode and a platinum wire counter electrode was then positioned within the collar, and a small volume of analyte/electrolyte solution (i.e. 40-100 µL) was pipetted into the collar. This yielded a complete 3-electode electrochemical cell. The porous frit of the reference electrode was always positioned proximal (i.e. within 3 mm) of the disk of the working electrode to minimise any uncompensated ohmic drop ("iR drop"). This setup is depicted in **Figure S118**.

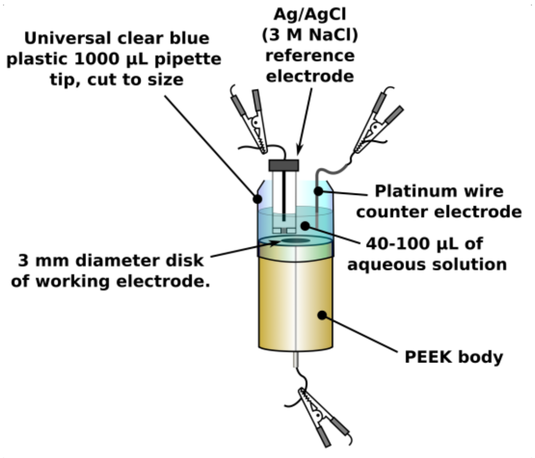

Figure S 118. 3-electrode setup used to perform low-volume electrochemical experiments using disk electrodes.

To prepare a 1 mM electrografting solution of **10**, 40 µL of pH 8.0 EChem buffer was added to an aliquot of **10** at 0°C. This solution was then either kept in darkness or exposed to 365 nm UV irradiation from a 12 W UV LED Helios® Nail lamp (Item Code: HGPK25) for 30 s at 0°C prior to being pipetted into the collar of the 3-electrode setup depicted above.

The blank electrografting solution was simply 40 µL of pure pH 8.0 EChem buffer.

For "-graft" samples, the tip of the working electrode was immersed in electrografting buffer but no cyclic voltammetry was performed. For "+graft" samples, cyclic voltammetry was performed using the parameters tabulated below.

**Equilibration time (s):**	5
**E begin (V vs REF):**	0.3
**E vertex1 (V vs REF):**	0.3

**E vertex2 (V vs REF):**	-0.35
**E step (V):**	0.05
**Scan rate (mV s^{-1}):**	20
**Number of scans**	4

After performing electrografting/electrode tip immersion, the working electrode was cleaned via rinsing thoroughly with HPLC-grade water, sonication in HPLC-grade water (1 minute), sonication in DMSO (1 minute) and then a final rinse in HPLC-grade water before being air dried prior to latter analysis.

The setup depicted in **Figure S118** (minus the working electrode) was thoroughly rinsed with HPLC-grade water. After this cleaning step was complete, the derivatised working electrode was then reconnected and the plastic collar filled with fresh pH 8.0 EChem buffer. In order to detect the presence of grafted viologen motifs, cyclic voltammograms were then recorded over a series of scan rates (10 mV s^{-1} → 60 V s^{-1}). All scans shared the following parameters:

**Equilibration time (s):**	5
**E begin (V vs REF):**	-0.25
**E vertex1 (V vs REF):**	-0.25
**E vertex2 (V vs REF):**	-0.8
**Number of scans**	3

Exploring the grafting behaviour of 10 using ITO-coated glass slides:

The 7 × 25 × 1.1 mm ITO-coated glass slides were inserted into a 1 mL chamber volume 10 mm pathlength cuvette and electrolyte/electrografting solution was added such that a 15 mm portion of the ITO-coated glass slide was immersed. By calculating the volume taken up by the immersed portion of the glass slide inside the cuvette, and by deducting this from the volume that would be expected to fill the cuvette to a depth of 15 mm, the volume of the solution can be calculated to be approximately 480 µL. The top of the ITO-coated glass side was then connected to the potentiostat using a crocodile clip, and a Ag/AgCl (3 M NaCl) reference electrode and a platinum wire counter electrode were then positioned in such a manner that they were in contact with the electrolyte solution but not in direct physical contact with the ITO-coated glass slide. This yielded a complete 3-electode electrochemical cell. The porous frit of the reference electrode was proximal (i.e. within 3 mm) of the immersed surface of the ITO-coated glass slide, thereby minimising any uncompensated ohmic drop ("iR drop"). This setup is depicted in **Figure S119**.

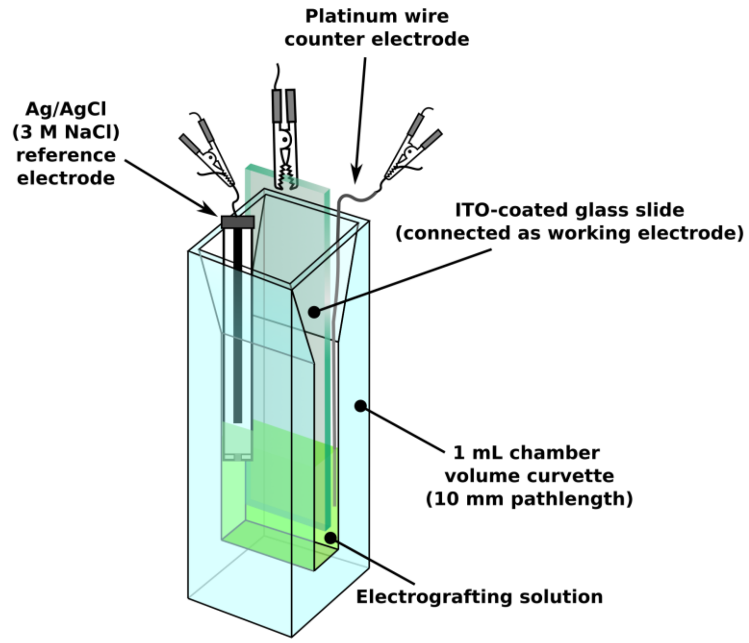

Figure S 119. 3-electrode setup used to perform electrochemical experiments using ITO-coated glass slides.

The blank electrografting solution was simply pure pH 8.0 EChem buffer. To record a blank over the electrografting range, cyclic voltammetry was performed using the parameters tabulated below.

**Equilibration time (s):**	5
**E begin (V vs REF):**	0.3
**E vertex1 (V vs REF):**	0.3
**E vertex2 (V vs REF):**	-0.35
**E step (V):**	0.05
**Scan rate (mV s^{-1}):**	20
**Number of scans**	4

After recording a blank over the electrografting range, a blank was recorded over a range appropriate for the detection of viologen motifs using the following parameters:

**Equilibration time (s):**	5
**E begin (V vs REF):**	-0.25
**E vertex1 (V vs REF):**	0.25
**E vertex2 (V vs REF):**	-0.8
**E step (V):**	0.005
**Scan rate (mV s^{-1}):**	400
**Number of scans**	3

After the blanks had been recorded 50 µL of the blank electrografting solution was pipetted from the 3-electrode setup and added to an aliquot of **10**. The resultant solution was then exposed to 365 nm UV irradiation from a 12 W UV LED Helios® Nail lamp (Item Code: HGPK25) for 30 s at 0°C and was then pipetted back into the 3-electrode setup. Assuming a final solution volume of approximately 480 µL, the concentration of diazonium cations derived from **10** in the resultant electrografting solution can be calculated as approximately 80 µM. Diazonium electrografting was then performed via cyclic voltammetry using the parameters tabulated below.

**Equilibration time (s):**	5
**E begin (V vs REF):**	0.3
**E vertex1 (V vs REF):**	0.3
**E vertex2 (V vs REF):**	-0.35
**E step (V):**	0.05
**Scan rate (mV s^{-1}):**	20
**Number of scans**	7

After performing electrografting with **10**, the setup depicted in **Figure S119** was thoroughly rinsed, first with HPLC-grade water, then with DMSO, then with HPLC-grade water, and then with fresh pH 8.0 EChem buffer. After this cleaning step was complete, the volume of fresh pH 8.0 EChem buffer in the 3-electrode setup was adjusted such that the ITO-coated glass slide was once again immersed to a depth of 15 mm. In order to detect the presence of grafted viologen motifs, cyclic voltammograms were then recorded over a series of scan rates (10 mV s^{-1} → 60 V s^{-1}). All scans shared the following parameters:

**Equilibration time (s):**	5
**E begin (V vs REF):**	-0.25
**E vertex1 (V vs REF):**	-0.25
**E vertex2 (V vs REF):**	-0.8
**Number of scans**	3

Grafting 12 onto a 3 disk working electrodes:

1 mM solutions of **12** in pH 8.0 EChem buffer were prepared, and was exposed to 365 nm UV irradiation from a 12 W UV LED Helios® Nail lamp (Item Code: HGPK25) for 30 s at 0°C prior to being pipetted into the collar of the 3-electrode setup depicted in **Figure S118**, using either 3 mm diameter glassy carbon or gold disk electrodes as working electrodes. Diazonium electrografting was then performed via cyclic voltammetry using the parameters tabulated below. Representative cyclic voltammograms of this diazonium electrografting are also included below (**Figure S120**).

**Equilibration time (s):**	5
**E begin (V vs REF):**	0.3
**E vertex1 (V vs REF):**	0.3
**E vertex2 (V vs REF):**	-0.35
**E step (V):**	0.05
**Scan rate (mV s^{-1}):**	20
**Number of scans**	4

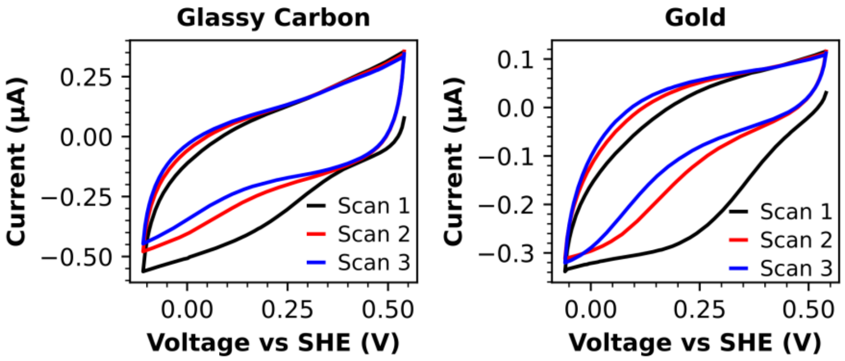

Figure S 120. Electrografting onto 3 mm diameter disk working electrodes using UV-exposed 1 mM solutions of **12**.

After performing electrografting/electrode tip immersion, the working electrode was cleaned via rinsing thoroughly with HPLC-grade water, sonication in HPLC-grade water (1 minute), sonication in DMSO (1 minute) and then a final rinse in HPLC-grade water.

A solution suitable for the Cu-catalysed azide alkyne click (CuAAC) reaction between surface-confined azide motifs and alkyne functionalised viologen **13** was then prepared. This CuAAC solution was prepared using pH 7.4 buffer (50 mM sodium phosphate + 150 mM NaCl) under nitrogen, with the individual solution components being delivered from appropriate stock solutions. The last component of the CuAAC solution to be added was **13**. The final composition of the solution was:

- 200 µM **13**
- 100 µM $CuSO_4$
- 500 µM tris(3-hydroxypropyltriazolylmethyl)amine
- 500 µM sodium ascorbate

The derivatised working electrodes were then immersed in the CuAAC solution and were incubated overnight under nitrogen at room temperature. After this time the electrodes were removed from the solution and were rinsed thoroughly with HPLC-grade water.

The derivatised working electrodes were then reconnected as depicted in **Figure S118**, and the plastic collars were filled with fresh pH 8.0 EChem buffer. In order to detect the presence of grafted viologen motifs, cyclic voltammograms were then recorded over a series of scan rates (10 mV s^{-1} → 60 V s^{-1}). All scans shared the following parameters:

**Equilibration time (s):**	5
**E begin (V vs REF):**	-0.25
**E vertex1 (V vs REF):**	-0.25
**E vertex2 (V vs REF):**	-0.8
**Number of scans**	3

Control experiments were likewise performed in which fresh pH 8.0 EChem buffer were used in place of 1 mM solutions of **12** during the electrografting step.

Isolation and analysis of viologen signals

Viologen-derived Faradaic signals were isolated via baseline subtraction, as is illustrated below in **Figure S121**.

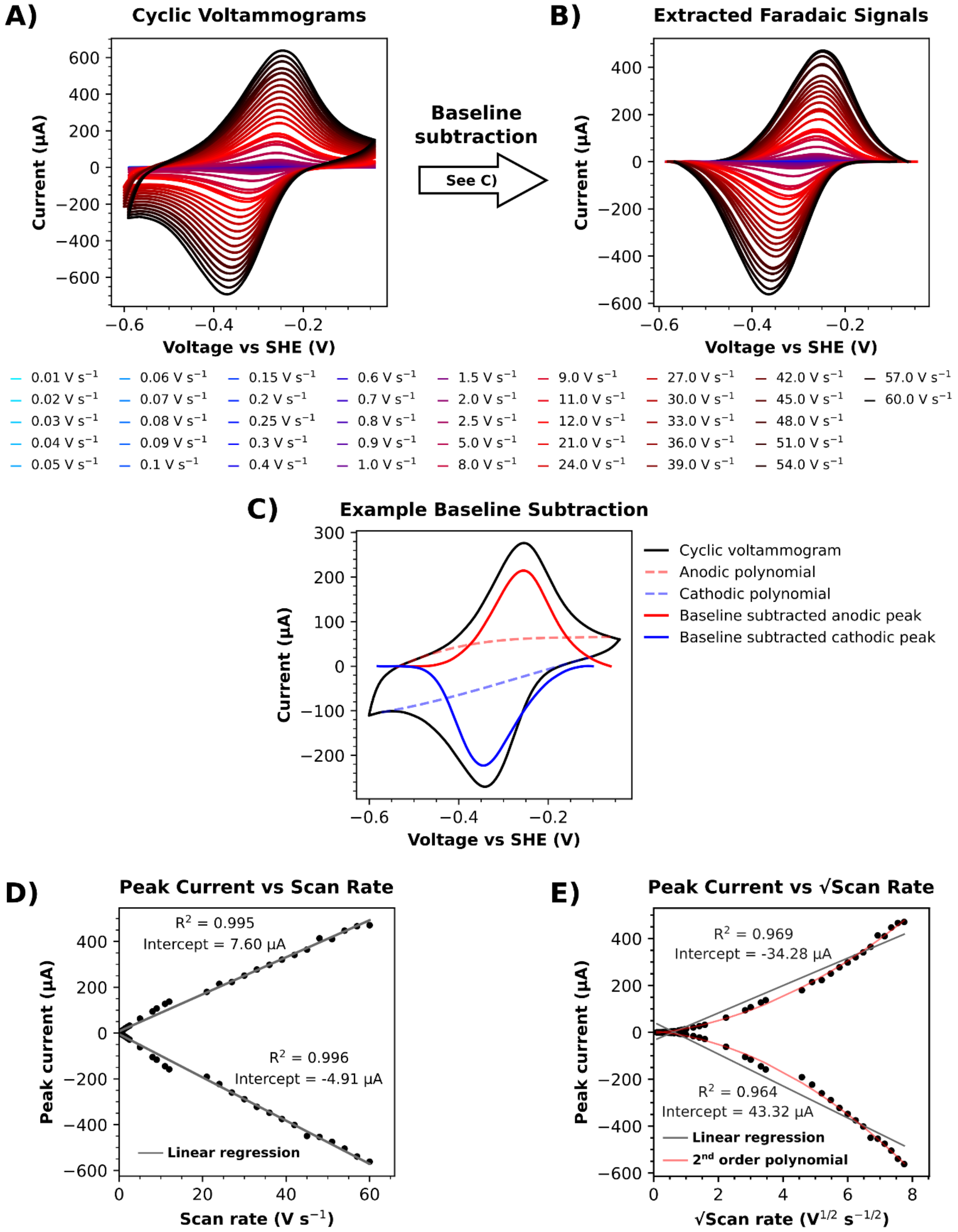

Figure S 121. Isolation and analysis of Faradaic signals from viologen motifs for glassy carbon electrodes electrografted with a UV-treated solution of **10**. **A)** Cyclic voltammograms recorded using a glassy carbon electrode subjected to electrografting with a UV-treated solution of **10** (1 mM of **10** in pH 8.0 50 mM sodium phosphate + 150 mM NaCl buffer). **B)** Faradaic signals isolated from cyclic voltammograms via baseline subtraction. **C)** Baseline subtraction is performed by using data from either side of a Faradaic signal to fit a polynomial that approximates the background capacitive (baseline) current. Subtraction of the experimental data from datapoints simulated using the polynomial can be used to approximately isolate the Faradaic current contribution from the total current. **D)** A plot of the peak current of the Faradaic signals as a function of scan rate. **E)** A plot of the peak current of the Faradaic signals as a function of √(scan rate).

Determination of surface-confined nature of redox couple

As can be seen in **Figure S121D**, a plot of the peak current of the Faradaic signals as a function of scan rate reveals linear relationships, the y-intercepts of which are the origin of the graph; this is characteristic of a surface-confined redox process. Furthermore, **Figure S121E** (a plot of the peak current of the Faradaic signals as a function of $\sqrt{scan\ rate}$) does not reveal a linear relationship, and the data is far better fitted to a second order polynomial; this again indicates that peak current is proportional to scan rate rather than to $\sqrt{scan\ rate}$, and thus demonstrates that the redox signals are not originating from solution-phase species.[59,60]

Calculation of surface coverage

The surface coverage (Γ) of the redox protein/enzyme on the electrode surface can be determined via the integration of isolated Faradaic signal (units = A = C s^{-1}) with respect to applied voltage (units = V = J C^{-1}), as described by **Equation S4**.[60]

$$\Gamma_{Total_{(Immob)}} = \frac{\int I_{Faradic}\, dE}{nvFA} \qquad \textbf{Equation S4}$$

$\boldsymbol{\Gamma_{Total_{(Immob)}}}$ = the surface coverage of the immobilised redox species / m^2.

$\int \boldsymbol{I_{Faradic}}\, \boldsymbol{dE}$ = the area enclosed by the Faradaic signal / J s^{-1}.

$\boldsymbol{n}$ = the number of electrons transferred in the redox couple.

$\boldsymbol{F}$ = the Faraday constant / C mol^{-1}.

$\boldsymbol{A}$ = the surface area of the electrode / m^2.

$\boldsymbol{v}$ = scan rate / J C^{-1} s^{-1}.

$\boldsymbol{E}$ = applied voltage rate / J C^{-1}.

Investigating potential origins of the attenuated diazonium electrografting behaviour of **4** relative to **5**

We sought to determine if any of the components present in UV-exposed solutions of **4** could explain their mildly attenuated electrografting performance compared to solutions of **5**. The most obvious differences between these two solutions were thought to be the presence or absence of 1 mM of the sterically bulky tetrabutylammonium cation, and the presence or absence up to 1 mM of the byproduct of **4**'s degradation (the conjugate base of **S7**). It was also considered that if incomplete conversion of **4** into diazonium cations was occurring, or if **S7** were capable of reacting with diazonium cations to reform triazabutadienes (**Figure S122**), that the concentration of diazonium cations would differ between UV-exposed solutions of **4** and solutions of **5**. A series of experiments were thus performed.

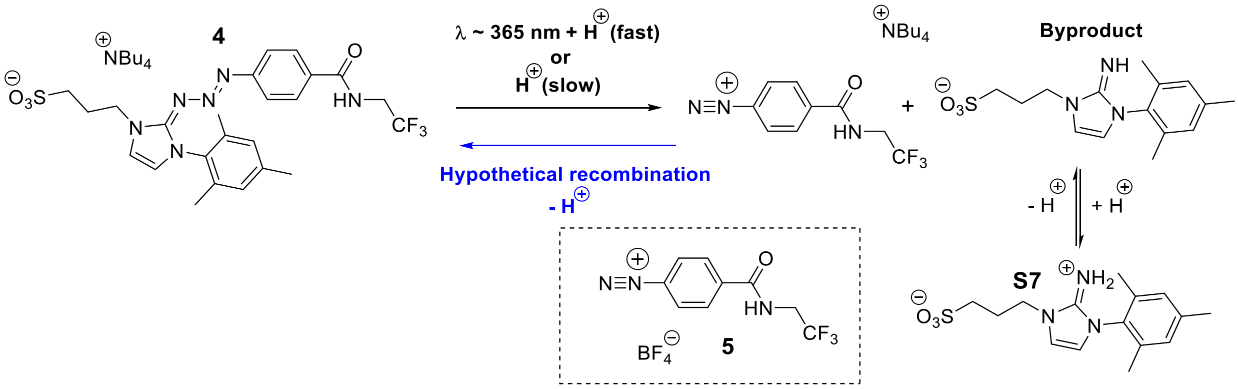

Figure S 122. The products of the protic degradation of **4** in comparison to **5,** and the possibility of reformation of **4** from its degradation products.

Experimental

Preparing various solutions of 4/5 of S7

A series of 1 mM solutions of **4** or **5** were prepared using the stock solutions/buffers listed below and the recipes tabulated below (**Table S1**). Each solution was prepared such that the acetonitrile content was 10% and the total volume was 2 mL. Each solution was prepared at 0°C. Solutions of **4** were then either kept in darkness or exposed to 365 nm UV irradiation from a *nailStar*® 36 Watt UV lamp (Model: NS-01-UK&EU) for 30 s at 0°C.

Stock solutions/buffers:

- **4 stock:** 20 mM **4** in acetonitrile
- **5 stock:** 20 mM **5** in pH 7.0 EChem buffer (kept at 0°C).
- **S7 stock:** 20 mM **S7** in water
- **TBAC buffer:** 2 mM tetrabutylammonium chloride (TBAC) in pH 7.0 EChem buffer.

Table S1: Recipes for making various solutions of **4**, **5** and **S7**.

Condition	Volume of 4 stock (µL)	Volume of S7 stock (µL)	Volume of TBAC buffer (µL)	Volume of pH 7.0 Echem buffer (µL)	Volume of acetonitrile (µL)
**4** (+ or - UV)	100	0	0	1800	100
**4** + UV + **S7**	100	100	0	1700	100
**4** + UV + TBAC	100	100	1000	700	100
Blank	0	0	1800	0	200

Condition	Volume of 5 stock (µL)	Volume of S7 stock (µL)	Volume of TBAC buffer (µL)	Volume of pH 7.0 Echem buffer (µL)	Volume of acetonitrile (µL)
**5**	100	0	0	1700	200
**5** + **S7**	100	100	0	1600	200
**5** + **S7** + TBAC	100	100	1000	600	200
**5** + TBAC	100	0	1000	700	200

Grafting various solutions of 4/5 and S7 onto 3 mm disk glassy carbon electrodes

After being freshly prepared solutions were immediately transferred into a glass vessel able to accommodate a 2 mL volume prior and three electrodes were inserted - a Ag/AgCl (3 M NaCl) reference electrode, a platinum wire counter electrode and a 3 mm diameter glassy carbon electrode, yielding a complete 3-electode electrochemical cell. Note that while the temperature of the electrografting solution could be assumed to be 0°C at the point of preparation, it was not practical to provide additional cooling to the solution during electrografting. Electrografting was performed via cyclic voltammetry using the parameters tabulated below.

**Equilibration time (s):**	5
**E begin (V vs REF):**	0.5
**E vertex1 (V vs REF):**	0.5
**E vertex2 (V vs REF):**	-0.5
**E step (V):**	0.005
**Scan rate (mV s^{-1}):**	20
**Number of scans**	3

UV-vis analysis of solutions of 4/5 and S7

After being freshly prepared, 100 µL of solution was added to 1900 µL of water in a BRAND™ UV-Cuvette Disposable Cuvette (Semi-Micro Cuvette, permissive to wavelengths in the range of 230 to 900 nm, 10 mm path length) and mixed thoroughly via pipetting several times. Data was then recorded using a DeNovix Ds-11 FX+ spectrophotometer.

NMR analysis of solution 5 + S7

After UV irradiation of a solution of **5** + **S7** solution the solution was incubated at rt in darkness for 5 minutes. After this time 900 µL of this solution was flash-frozen using liquid nitrogen and lyophilized. The material yielded was then re-dissolved in D_2O (500 µL) and subjected to analysis by ^{1}H, ^{19}F and COSY NMR.

Results

The cyclic voltammograms recorded are shown below (**Figure S123**). The presence or absence of tetrabutylammonium chloride (TBAC) had no notable or consistent effect on electrografting intensity. Addition of 1 mM of **S7** to electrografting solutions of **4** or **5** did however appear to mildly attenuate the electrografting intensity. We also noted that, upon the addition of **S7** to solutions of **5**, the solution changes from colourless to the characteristic green/yellow of triazabutadienes, with the colour developing in intensity over the course of several minutes. We also noticed that 1 mM solutions of **4** did not completely decolourise upon UV exposure, making us wonder if the flux of UV-irradiation the 1 mM samples of **4** were exposed to, coupled with a degree of recombination between the released aryl diazonium cations and **S7** (which will be more prevalent at 1 mM concentration than at 50 µM) was resulting in the aryl diazonium concentration in solution being lower than 1 mM, and thereby attenuating the intensity of electrografting reductive features observed. To further investigate this, we performed NMR experiments and UV-vis studies.

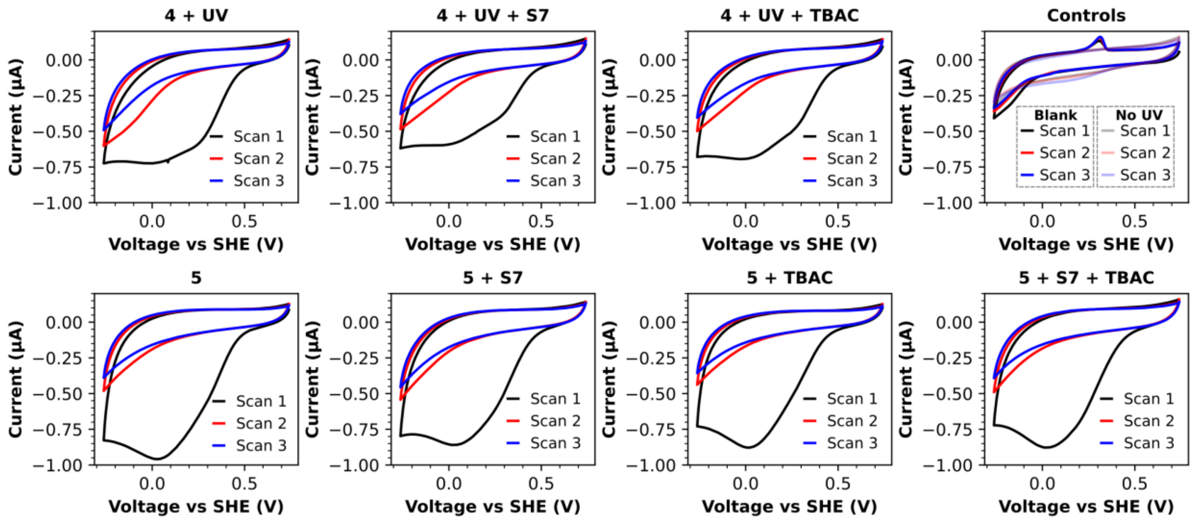

Figure S 123. Electrografting onto 3 mm diameter disk glassy carbon working electrodes using various 1 mM solutions of **4** and **5**.

UV-vis analysis (**Figure S124**) showed that approximately 60% of **4** had been converted to aryl diazonium cations after their brief UV-exposure. The UV-vis analysis also showed that the presence of an additional 1 mM of **S7** in samples of **4** further reduced this conversion to approximately 50%. The addition of 1 mM of **S7** to samples of **5** also resulted in a new absorption band developing over the course of several minutes at λ_{max} 393 nm, which is characteristic of triazabutadienes. The analysis of the **5** + **S7** solution via NMR (**Figure S125**) also showed that triazabutadiene had formed, and thus these data strongly show that a degree of recombination between **S7** and the aryl diazonium cations released from **4** is possible at 1 mM concentration. This, coupled with the incomplete conversion of 1 mM solutions of **4** into aryl diazonium cations during their brief UV-exposure explains the moderately attenuated electrografting intensity of UV-exposed 1 mM solutions of **4** relative to that observed when using **5**.

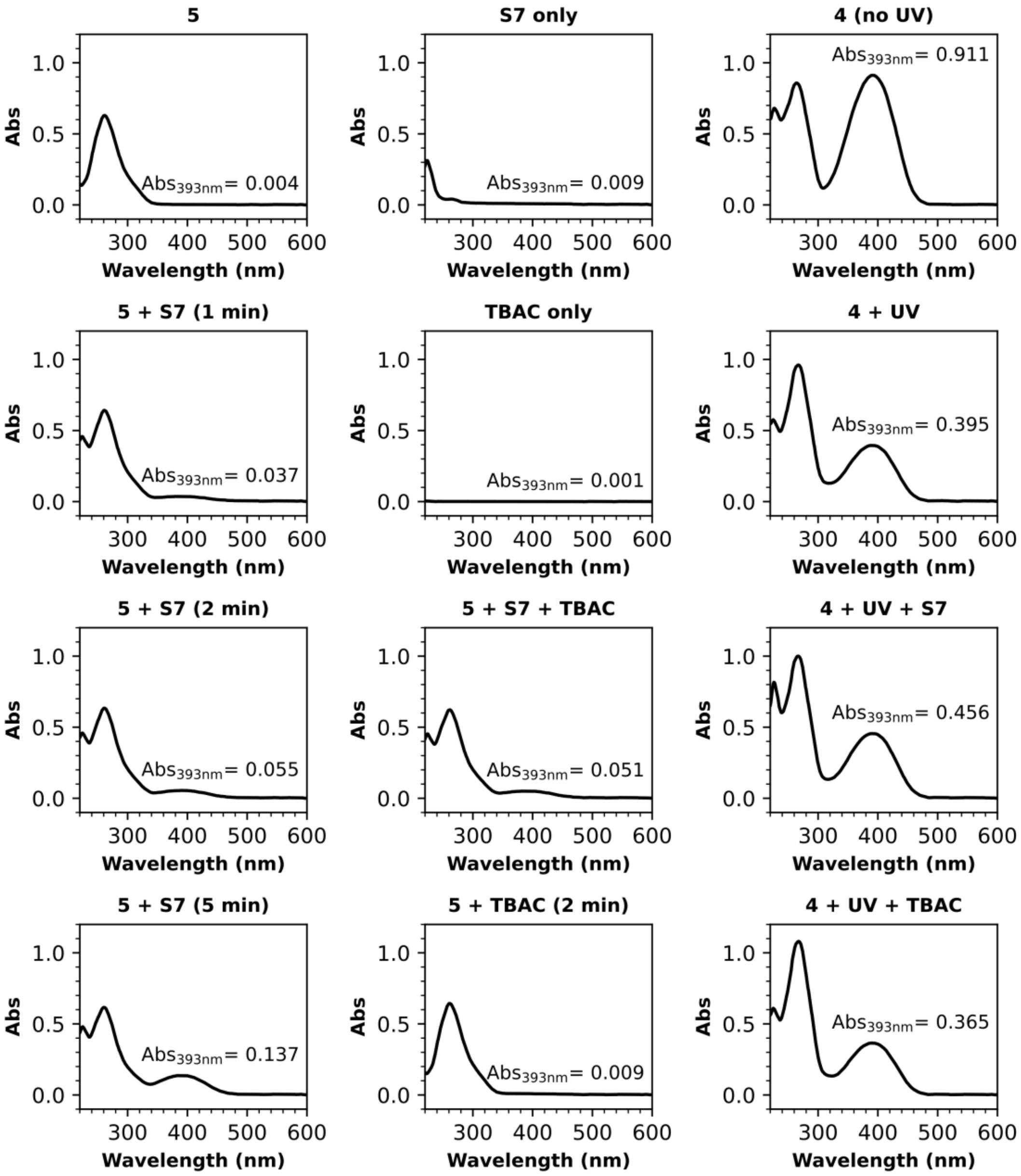

Figure S 124. UV-vis analysis of various solutions of **4** and **5**.

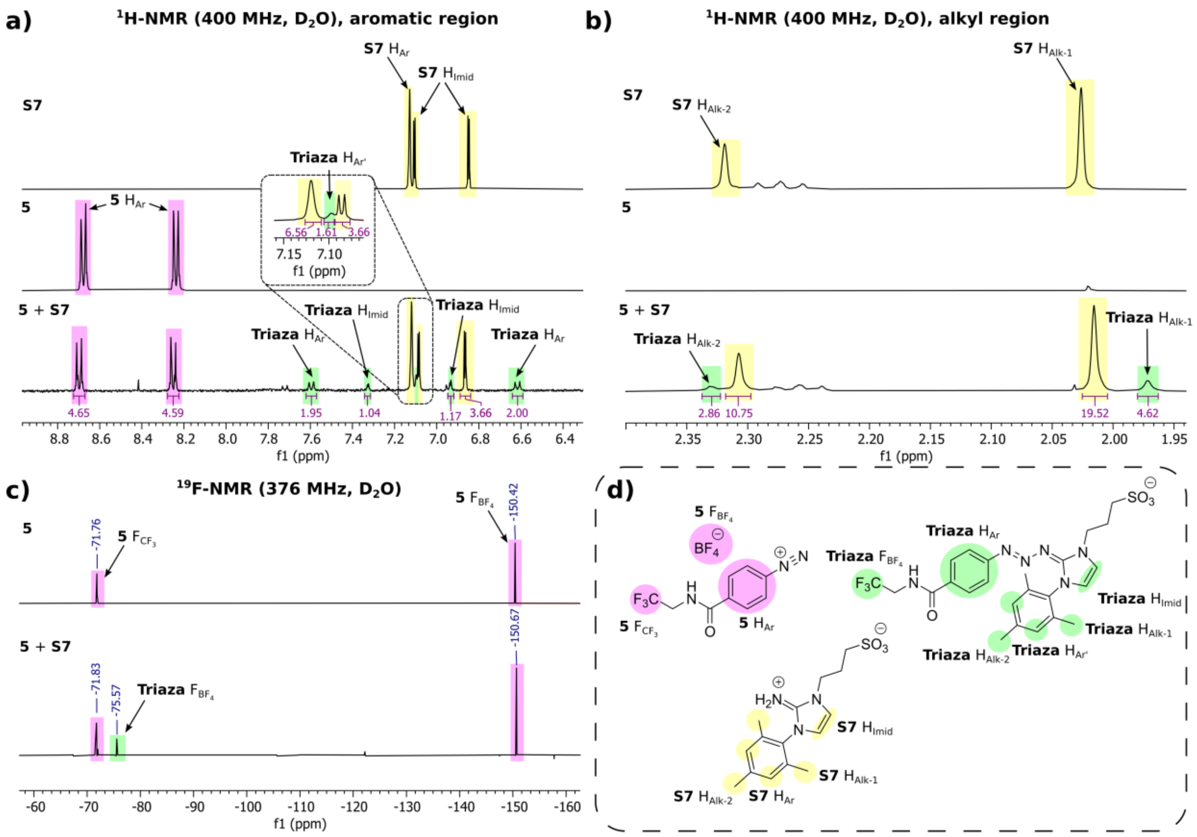

Figure S 125. NMR analyses of various solution **5** and **S7**. **a)** ^{1}H-NMR of the aromatic region of **S7**, **5** and a mixture of **5** and **S7**. **b)** ^{1}H-NMR of the alkyl region of **S7**, **5** and a mixture of **5** and **S7**. **c)** ^{19}F-NMR of **S7**, **5** and a mixture of **5** and **S7**. **d)** Annotation of characteristic nuclear environments. All NMR spectra were recorded using D_2O as the solvent.

X-ray Photoelectron Spectroscopy (XPS) data

Acknowledgments

The X-ray Photoelectron Spectroscopy (XPS) data collection was performed at the EPSRC National Facility for XPS ("HarwellXPS"), operated by Cardiff University and University College London (UCL), under Contract No. PR16195.

Experimental

The gold-coated silicon wafer slice samples, as described in the "**Grafting 4/5 onto gold-coated silicon wafer slices**" section, were analysed using XPS.

XPS data was acquired using a Kratos Axis SUPRA using monochromated Al kα (1486.69 eV) X-rays at 15 mA emission and 12 kV HT (180 W) and a spot size/analysis area of 700 x 300 µm. The instrument was calibrated to the binding energy (BE) of the gold metal Au $4f_{7/2}$ line (83.95 eV)[30,31] and dispersion adjusted give a BE of 932.6 eV for the Cu $2p_{3/2}$ line of metallic copper. The Ag $3d_{5/2}$ line full width at half maximum (FWHM) at 10 eV pass energy was 0.544 eV. The source resolution for monochromatic Al kα X-rays was ~0.3 eV. The instrumental resolution was determined to be 0.29 eV at 10 eV pass energy using the Fermi edge of the valence band for metallic silver. Resolution with charge compensation system on <1.33 eV FWHM on polytetrafluoroethylene (PTFE). High resolution spectra were obtained using a pass energy of 20 eV, step size of 0.1 eV and sweep time of 60 s, resulting in a line width of 0.696 eV for Au $4f_{7/2}$. Survey spectra were obtained using a pass energy of 160 eV. Samples were ground the instrument via conductive clips. The data was recorded at a base pressure of below 9 x 10^{-9} Torr, a room temperature of 294 K and an angle of 30°.

Analysis

The data was analysed using CasaXPS v2.3.19PR1.0.[32] Data were fit with a Shirley background prior to component analysis, and were calibrated by setting the Au $4f_{7/2}$ peak to be equal to the literature value of 83.95 eV.[30,31]

The binding energies of the assigned components used to model the C 1s XPS region were based on those reported for similar carbon chemical environments in the literature,[33-35] as were those used to model the O 1s[36,37] and F 1s[38] regions. The S 2p region[39] did not contain any readily identifiable signals.

XPS data

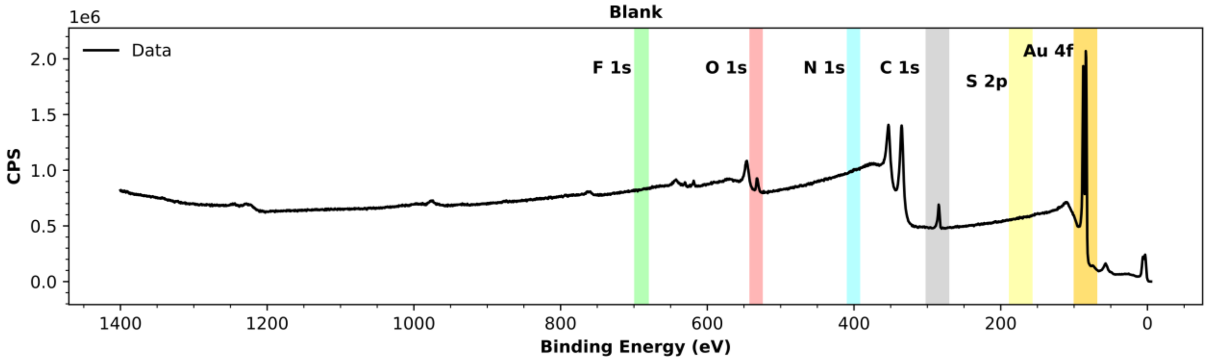

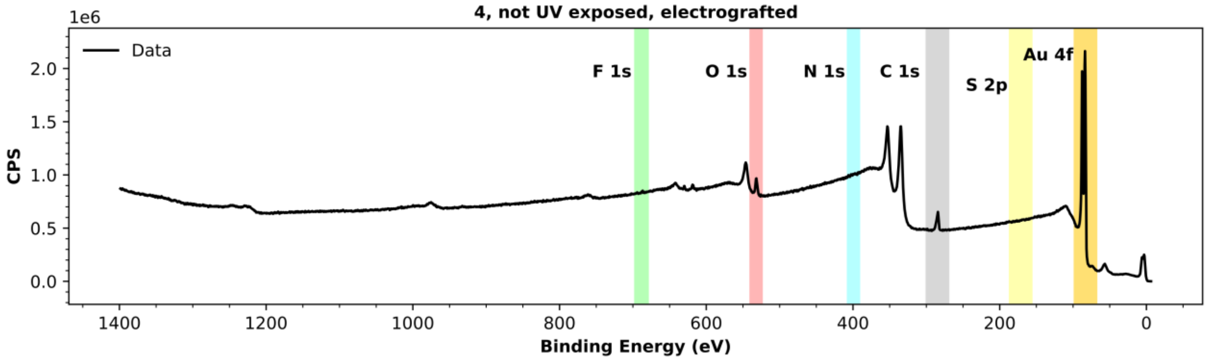

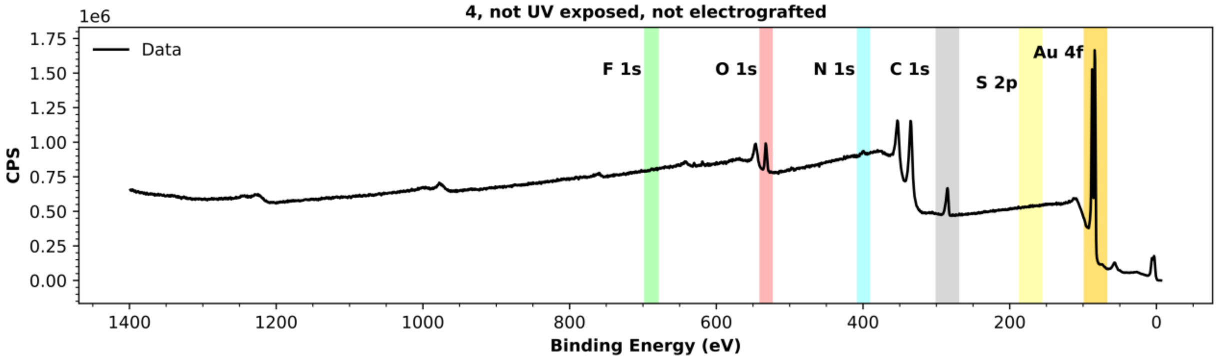

continued overleaf...

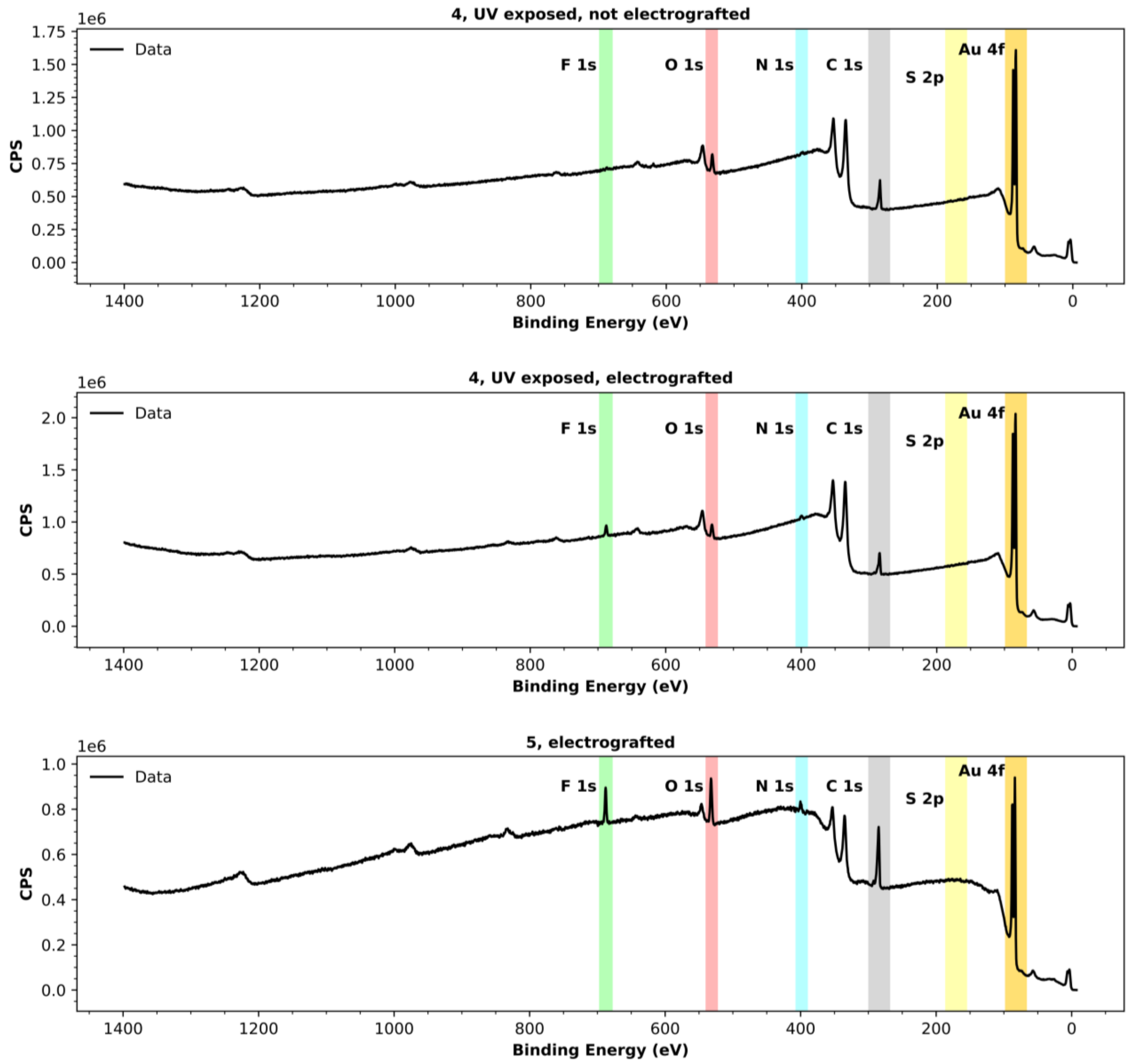

Figure S 126. Wide-scan XPS spectra of gold-coated silicon wafer slices subjected to various treatments with solutions of **4**/**5**.

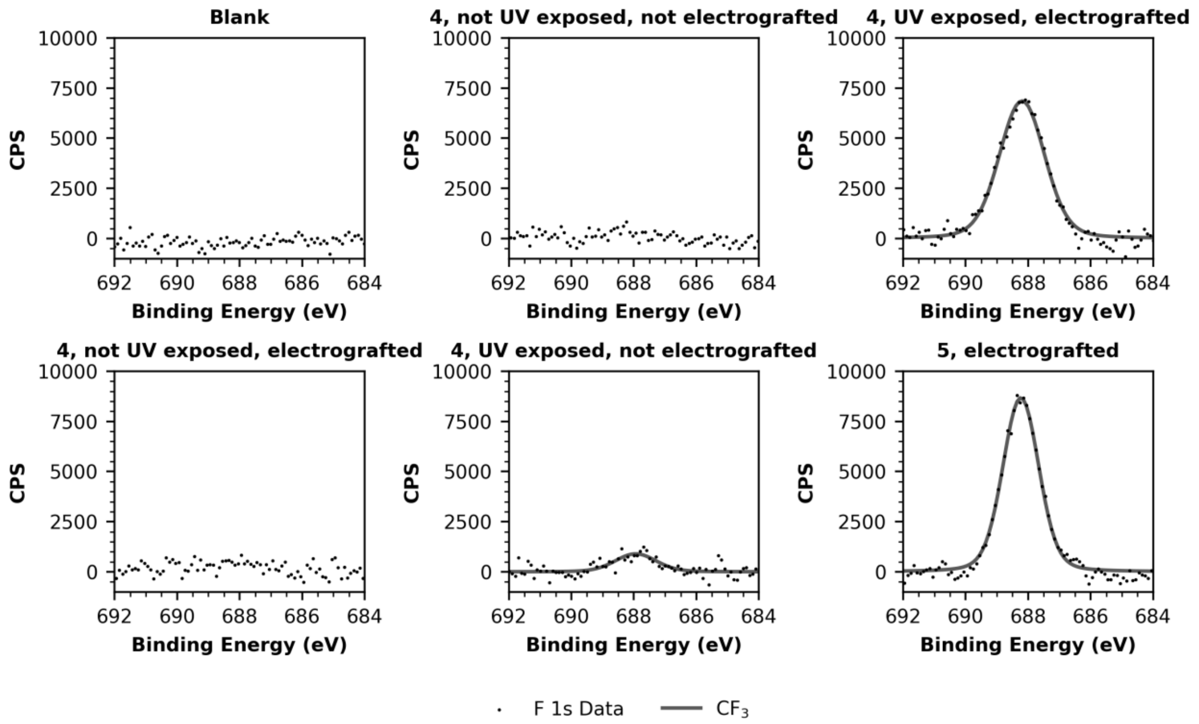

Figure S 127. Fluorine 1s XPS spectra of gold-coated silicon wafer slices subjected to various treatments with solutions of **4**/**5**.

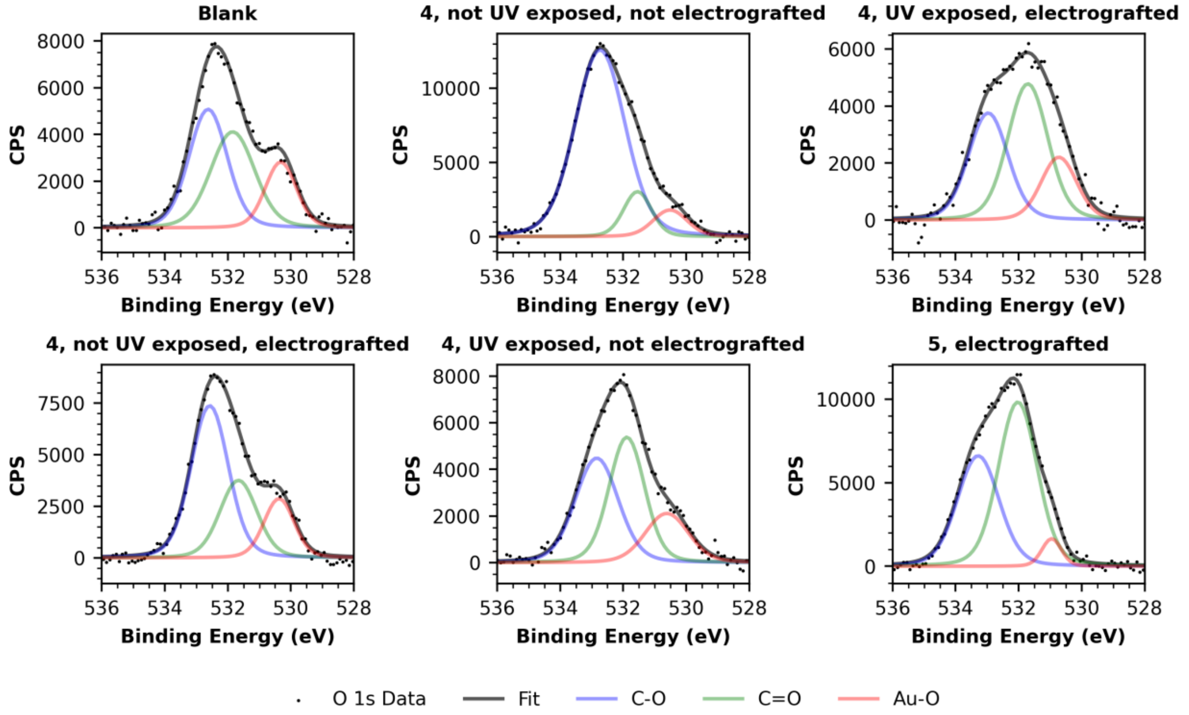

Figure S 128. Oxygen 1s XPS spectra of gold-coated silicon wafer slices subjected to various treatments with solutions of **4**/**5**.

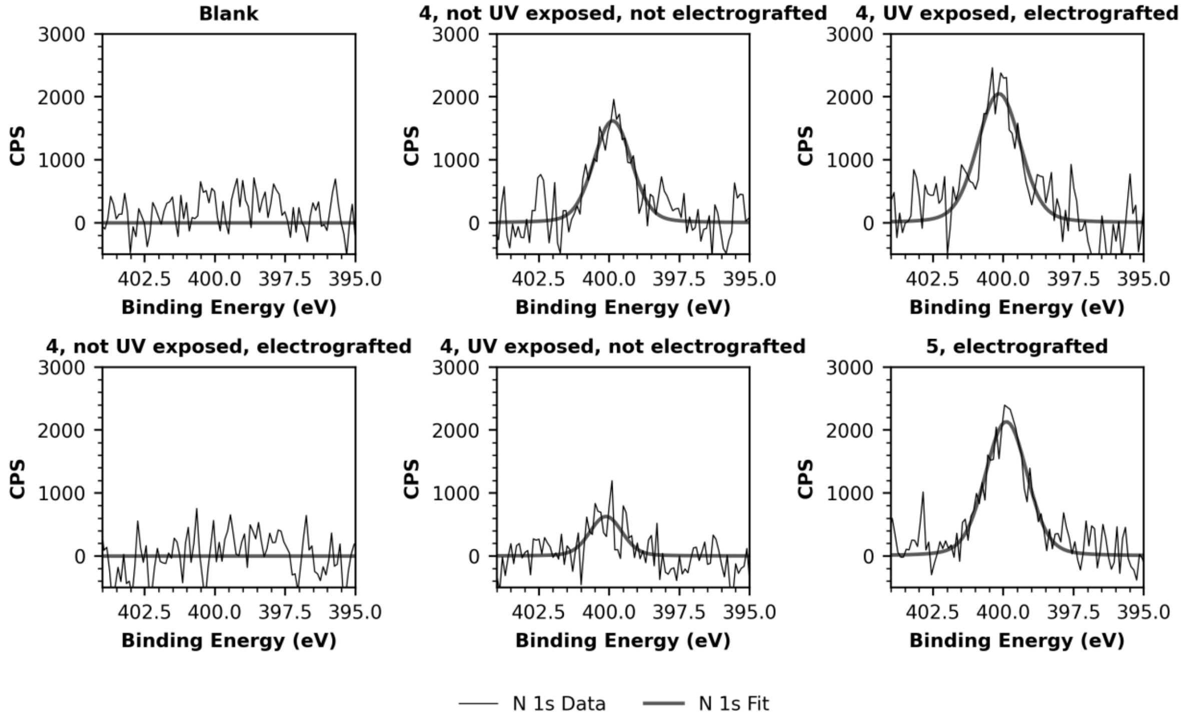

Figure S 129. Nitrogen 1s XPS spectra of gold-coated silicon wafer slices subjected to various treatments with solutions of **4**/**5**.

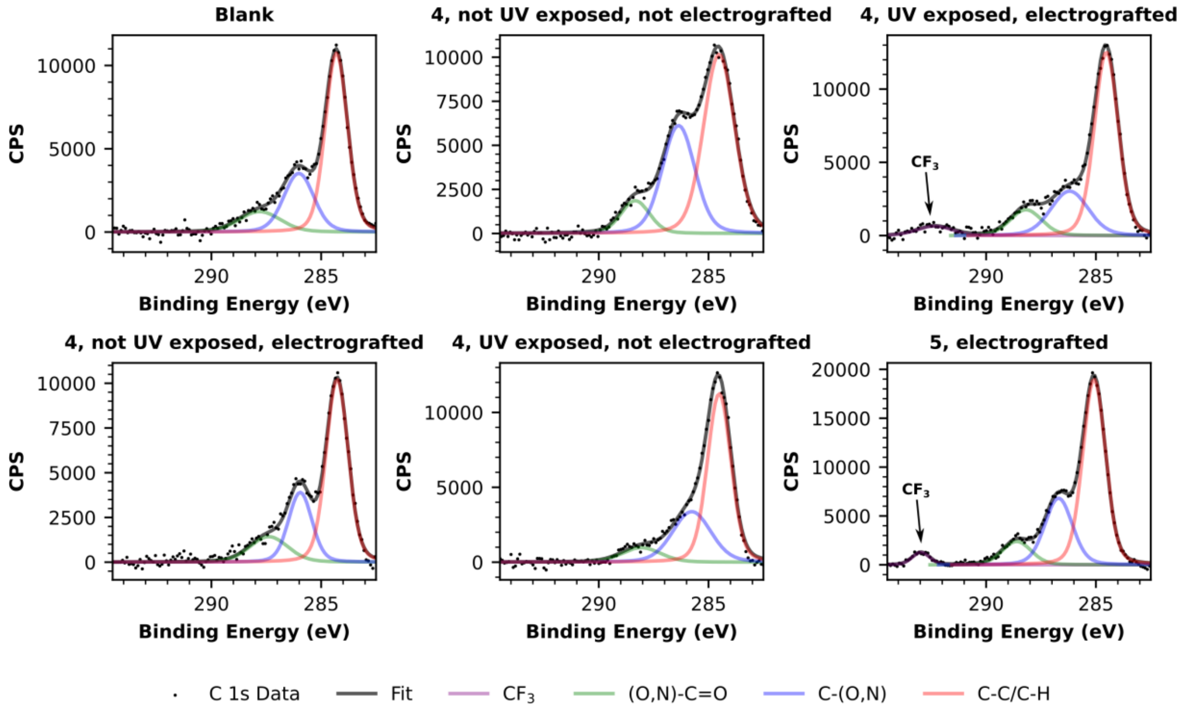

Figure S 130. Carbon 1s XPS spectra of gold-coated silicon wafer slices subjected to various treatments with solutions of **4**/**5**.

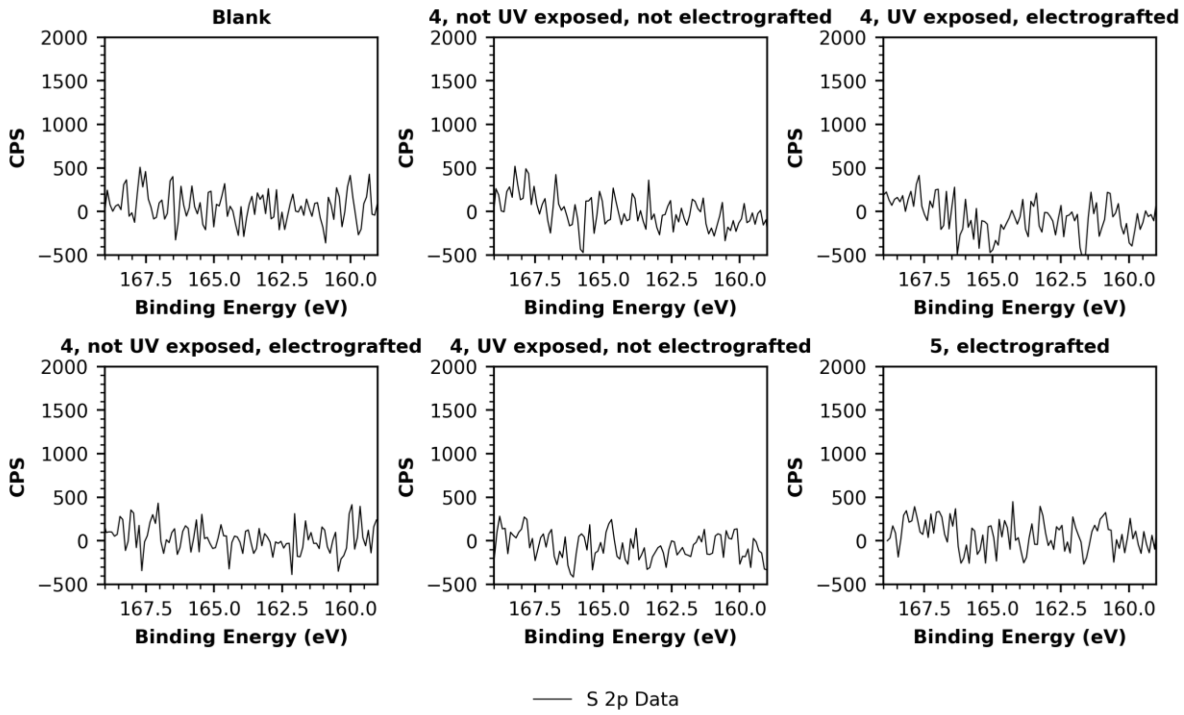

Figure S 131. Sulfur 2p XPS spectra of gold-coated silicon wafer slices subjected to various treatments with solutions of **4**/**5**.

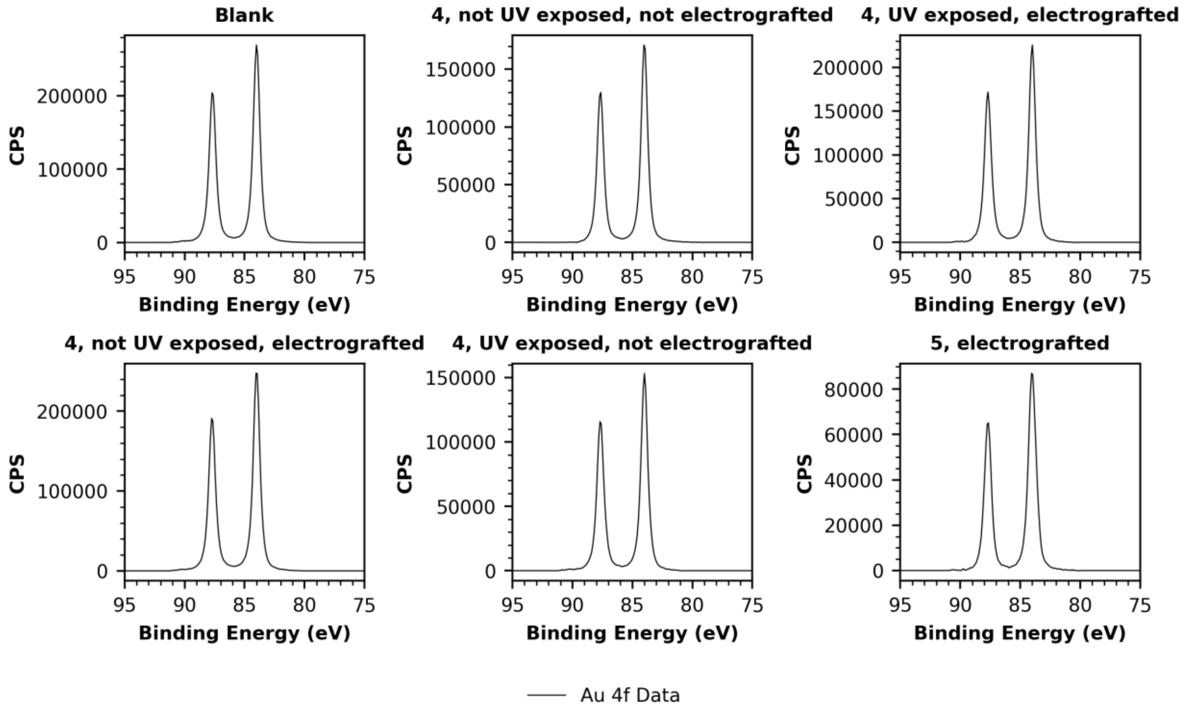

Figure S 132. Gold 4f XPS spectra of gold-coated silicon wafer slices subjected to various treatments with solutions of **4**/**5**.
